# Supplementary material for: Climate influence on the early human occupation of South America during the late Pleistocene
Source: Nat Commun. 2025 Mar 21;16:2780. doi: 10.1038/s41467-025-58134-5 (PMC11928665; doi:10.1038/s41467-025-58134-5)
Supplement: Supplementary file 1 — Supplementary Information [file 41467_2025_58134_MOESM1_ESM.pdf]

**Supplementary Information**

# Climate influence on the early human occupation of South America during the late Pleistocene

Becerra-Valdivia, L.[1,2]

[1]Department of Anthropology and Archaeology, University of Bristol, United Kingdom

[2]Linacre College, University of Oxford, United Kingdom

## Table of Contents


**Supplementary Figures** 1

**Supplementary Notes** 39

1. Lithic traditions 39
   - 1.2. Abriense/Tequendamiense 39
   - 1.3. El Jobo 39
   - 1.4. Fishtail/Fell 39
   - 1.5. Huentelauquén 39
   - 1.6. Itaparica 39
   - 1.7. Paiján 39
   - 1.8. Pay Paso and Tigre 40
2. Site reports 41
   - 2.1. Argentina 41
     - 2.1.1. Agua de la Cueva 41
     - 2.1.2. Arroyo Seco 2 (AS2) 42
     - 2.1.3. Campo Laborde 44
     - 2.1.4. Casa del Minero 1 46
     - 2.1.5. Cerro La China (1-3) 47
     - 2.1.6. Cerro Tres Tetas (C3T) 48
     - 2.1.7. El Alto 3 49
     - 2.1.8. Gruta del Indio 50
     - 2.1.9. Paso Otero 5 52
     - 2.1.9. Piedra Museo (AEP-1) 53
     - 2.1.10. La Moderna 55
   - 2.2. Brazil 55
     - 2.2.1. Abrigo do Sol 55
     - 2.2.2. Boqueirão do Sítio da Pedra Furada 56
     - 2.2.3. Caverna da Pedra Pintada 59
     - 2.2.4. Lapa do Boquete 63
     - 2.2.5. Lapa dos Bichos 66
     - 2.2.6. Lapa Vermelha IV 67
     - 2.2.7. Santa Elina 67
     - 2.2.8. RS-I-69 (Laranjito) 71
     - 2.2.9. Santana do Riacho 72
     - 2.2.10. Toca da Janela da Barra do Antonião 74
     - 2.2.11. Toca do Sítio do Meio 75
     - 2.2.12. Toca do Garrincho 77
     - 2.2.13. Vale da Pedra Furada 78

2.3. Bolivia 81
2.3.1. Cueva Bautista (AL03) 81
2.4. Chile 83
2.4.1. La Cueva 1 del Lago Sofía (or Lago Sofía 1) 83
2.4.2. Cueva de Fell 83
2.4.3. Cueva de la Vieja 84
2.4.4. Cueva del Medio 85
2.4.5. Los Rieles 87
2.4.6. Pampa Ramaditas 5 90
2.4.7. Monte Verde I & II 91
2.4.8. Pilauco 93
2.4.9. Punta Ñagué 95
2.4.10. Tagua Tagua 96
2.4.11. Tres Arroyos 1 (TA1) 98
2.4.12. Quebrada Maní 12, 32 and 35 100
2.4.13. Quebrada Santa Julia (LV. 221) 104
2.4.14. Quereo 104
2.5. Colombia 105
2.5.1. El Abra 105
2.5.2. La Palestina 2 (05YON002-02) 106
2.5.3. Nare (005PNA005) 107
2.5.4. San Juan de Bedout (05PBE014) 107
2.5.5. Serranía La Lindosa 108
2.5.6. Tequendama 108
2.5.7. Tibitó 1 & 2 110
2.6. Peru 111
2.6.1. Cuncaicha 111
2.6.2. Guitarrero Cave 114
2.6.3. Huaca Prieta 117
2.6.5. Pampa de los Fósiles and Ascope 120
2.6.6. Quebrada Jaguay (QJ-280) 121
2.6.7. Quebrada Tacahuay 122
2.6.8. The Ring Site 123
2.7. Uruguay 125
2.7.1. K87 (Arroyo del Tigre) 125
2.7.2. Pay Paso 126
2.8. Venezuela 129
2.8.1. El Vano 129
2.8.2. Taima-Taima 129

3. OxCal code (not already in previous sections; .prior files in Source data folder) 132
Figure 2 132
Figure 3 132
Figure 4 140
References **141**

**Supplementary Figures**

This section includes supplementary figures noted in the main text. All site-specific figures are included within each site report in the following sections, as well as those related to sensitivity testing. Where appropriate, OxCal code follows each figure.

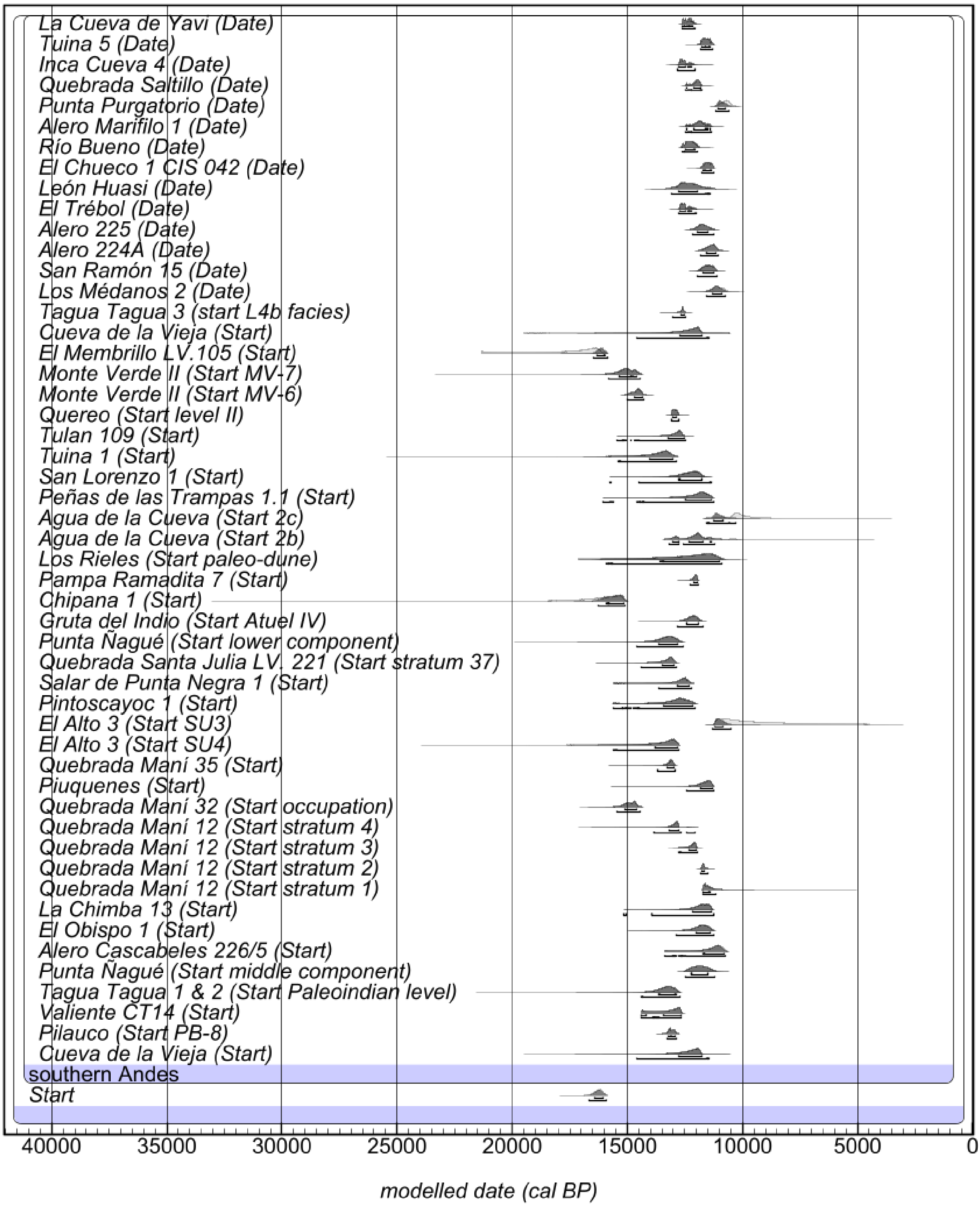


**Supplementary Figure 1.** Bayesian model showing the estimated start of cultural occupation in the southern Andes (16660-15905 cal BP), including all ACR/YD-aged cultural components within this province (entered as either start boundaries or single ages given available data, and noted next to each component name). Brackets beneath each age estimate show 68.3% and 95.4% CI.

```
Plot()
{
 Curve("SHCal20","shcal20.14c");
 Sequence()
 {
  Boundary("Start");
  Phase("southern Andes")
  {
   Date(Prior("Cueva de la Vieja (Start)","CdlV_S.prior"))
   {
    site="Cueva de la Vieja";
    latitude=-42.274167;
    longitude=-71.54;
    country="Chile";
    type="cave";
    high altitude="n";
    province="southern Andes";
    technology="uniface/biface";
    megafauna kill/scavenge="y";
   };
   Date(Prior("Pilauco (Start PB-8)","Pil_PB8_S.prior"))
   {
    site="Pilauco";
    latitude=-40.566667;
    longitude=-73.116667;
    country="Chile";
    type="open-air";
    high altitude="n";
    province="southern Andes";
    technology="uniface/biface";
    megafauna kill/scavenge="n";
   };
   Date(Prior("Valiente CT14 (Start)","Valiente_S.prior"))
   {
    site="Valiente CT14";
    latitude=-32.028333;
    longitude=-71.160833;
    country="Chile";
    type="open-air";
    high altitude="n";
    province="southern Andes";
    technology="Fishtail";
    megafauna kill/scavenge="n";
   };
   Date(Prior("Tagua Tagua 1 & 2 (Start Paleoindian level)","TT_S_Pleis.prior"))
   {
    site="Tagua Tagua 1 & 2";
    latitude=-34.4;
    longitude=-71.2;
    country="Chile";
    type="open-air";
    high altitude="n";
    province="southern Andes";
    technology="Fishtail";
    megafauna kill/scavenge="y";
   };
   Date(Prior("Punta Ñagué (Start middle component)","PN_S_middle.prior"))
   {
    site="Punta Ñagué";
    latitude=-31.85;
    longitude=-71.52;
    country="Chile";
    type="open-air";
    high altitude="n";
    province="southern Andes";
    technology="Huentelauquen";
    megafauna kill/scavenge="n";
   };
   Date(Prior("Alero Cascabeles 226/5 (Start)","AC_226_S.prior"))
   {
    site="Alero Cascabeles 226/5";
    latitude=-25.2;
    longitude=-70.4;
    country="Chile";
    type="rockshelter";
    high altitude="n";
    province="southern Andes";
    technology="Huentelauquen";
    megafauna kill/scavenge="n";
   };
   Date(Prior("El Obispo 1 (Start)","EO1_S.prior"))
   {
    site="El Obispo 1";
    latitude=-26.5;
    longitude=-70.6;
    country="Chile";
    type="open-air";
    high altitude="n";
    province="southern Andes";
    technology="Huentelauquen";
    megafauna kill/scavenge="n";
   };
   Date(Prior("La Chimba 13 (Start)","LC13_S.prior"))
   {
    site="La Chimba 13";
    latitude=-23.545556;
    longitude=-70.368889;
    country="Chile";
    type="open-air";
    high altitude="n";
    province="southern Andes";
    technology="Huentelauquen";
    megafauna kill/scavenge="n";
   };
   Date(Prior("Quebrada Maní 12 (Start stratum 1)","QM12_S_1.prior"))
   {
    site="Quebrada Maní 12";
    latitude=-21.2;
    longitude=-69.4;
    country="Chile";
    type="open-air";
    high altitude="n";
    province="southern Andes";
    technology="projectile point";
    megafauna kill/scavenge="n";
   };
   Date(Prior("Quebrada Maní 12 (Start stratum 2)","QM12_S_2.prior"))
   {
    site="Quebrada Maní 12";
    latitude=-21.2;
    longitude=-69.4;
    country="Chile";
    type="open-air";
    high altitude="n";
    province="southern Andes";
    technology="projectile point";
    megafauna kill/scavenge="n";
   };
   Date(Prior("Quebrada Maní 12 (Start stratum 3)","QM12_S_3.prior"))
   {
    site="Quebrada Maní 12";
    latitude=-21.2;
    longitude=-69.4;
    country="Chile";
    type="open-air";
    high altitude="n";
    province="southern Andes";
    technology="projectile point";
    megafauna kill/scavenge="n";
   };
   Date(Prior("Quebrada Maní 12 (Start stratum 4)","QM12_S_4.prior"))
   {
    site="Quebrada Maní 12";
    latitude=-21.2;
    longitude=-69.4;
    country="Chile";
    type="open-air";
    high altitude="n";
    province="southern Andes";
    technology="projectile point";
    megafauna kill/scavenge="n";
   };
   Date(Prior("Quebrada Maní 32 (Start occupation)","QM32_S.prior"))
   {
    site="Quebrada Maní 32";
    latitude=-21.2;
    longitude=-69.4;
    country="Chile";
    type="open-air";
    high altitude="n";
    province="southern Andes";
    technology="projectile point";
    megafauna kill/scavenge="n";
   };
   Date(Prior("Piuquenes (Start)","Piuquenes_S.prior"))
   {
    site="Piuquenes";
    latitude=-32.266667;
    longitude=-70.266667;
    country="Chile";
    type="rockshelter";
    high altitude="n";
    province="southern Andes";
    technology="projectile point";
    megafauna kill/scavenge="n";
   };
   Date(Prior("Quebrada Maní 35 (Start)","QM35_S.prior"))
   {
    site="Quebrada Maní 35";
    latitude=-21.2;
    longitude=-69.4;
    country="Chile";
    type="open-air";
    high altitude="n";
    province="southern Andes";
    technology="projectile point";
    megafauna kill/scavenge="n";
   };
   Date(Prior("El Alto 3 (Start SU4)","EA3_SU4_S.prior"))
   {
    site="El Alto 3";
    latitude=-31.3988;
    longitude=-64.7388;
    country="Argentina";
    type="rockshelter";
    high altitude="n";
    province="southern Andes";
    technology="projectile point";
    megafauna kill/scavenge="n";
   };
   Date(Prior("El Alto 3 (Start SU3)","EA3_SU3_S.prior"))
   {
    site="El Alto 3";
    latitude=-31.3988;
    longitude=-64.7388;
    country="Argentina";
    type="rockshelter";
    high altitude="n";
    province="southern Andes";
    technology="projectile point";
    megafauna kill/scavenge="n";
   };
   Date(Prior("Pintoscayoc 1 (Start)","Pinto1_S.prior"))
   {
    site="Pintoscayoc 1";
    latitude=-22.9444;
    longitude=-65.4182;
    country="Argentina";
    type="rockshelter";
    high altitude="y";
    province="southern Andes";
    technology="projectile point";
    megafauna kill/scavenge="n";
   };
   Date(Prior("Salar de Punta Negra 1 (Start)","SPN1_S.prior"))
   {
    site="Salar de Punta Negra 1";
    latitude=-24.466667;
    longitude=-68.883333;
    country="Chile";
    type="open-air";
    high altitude="y";
    province="southern Andes";
    technology="projectile point";
    megafauna kill/scavenge="n";
   };
   Date(Prior("Quebrada Santa Julia LV. 221 (Start stratum 37)","QSJ_S_37.prior"))
   {
    site="Quebrada Santa Julia LV. 221";
    latitude=-31.836917;
    longitude=-71.488278;
    country="Chile";
    type="open-air";
    high altitude="n";
    province="southern Andes";
    technology="projectile point";
    megafauna kill/scavenge="y";
   };
   Date(Prior("Punta Ñagué (Start lower component)","PN_S_lower.prior"))
   {
    site="Punta Ñagué";
    latitude=-31.85;
    longitude=-71.52;
```

```
 country="Chile";
 type="open-air";
 high altitude="n";
 province="southern Andes";
 technology="uniface/biface";
 megafauna kill/scavenge="n";
};
Date(Prior("Gruta del Indio (Start Atuel IV)","GdI_IV_S.prior"))
{
 site="Gruta del Indio";
 latitude=-34.75;
 longitude=-68.3666;
 country="Argentina";
 type="cave";
 high altitude="n";
 province="southern Andes";
 technology="uniface/biface";
 megafauna kill/scavenge="n";
};
Date(Prior("Chipana 1 (Start)","Chipana1_S.prior"))
{
 site="Chipana 1";
 latitude=-21.2;
 longitude=-69.4;
 country="Chile";
 type="open-air";
 high altitude="n";
 province="southern Andes";
 technology="uniface/biface";
 megafauna kill/scavenge="n";
};
Date(Prior("Pampa Ramadita 7 (Start)","PR7_S.prior"))
{
 site="Pampa Ramadita 7";
 latitude=-21.2;
 longitude=-69.4;
 country="Chile";
 type="open-air";
 high altitude="n";
 province="southern Andes";
 technology="uniface/biface";
 megafauna kill/scavenge="n";
};
Date(Prior("Los Rieles (Start paleo-dune)","LR_S_dune.prior"))
{
 site="Los Rieles";
 latitude=-31.93;
 longitude=-71.52;
 country="Chile";
 type="open-air";
 high altitude="n";
 province="southern Andes";
 technology="uniface/biface";
 megafauna kill/scavenge="n";
};
Date(Prior("Agua de la Cueva (Start 2b)","AdC_2b_S.prior"))
{
 site="Agua de la Cueva";
 latitude=-32.6169;
 longitude=-69.1636;
 country="Argentina";
 type="rockshelter";
 high altitude="y";
 province="southern Andes";
 technology="uniface/biface";
 megafauna kill/scavenge="n";
};
Date(Prior("Agua de la Cueva (Start 2c)","AdC_2c_S.prior"))
{
 site="Agua de la Cueva";
 latitude=-32.6169;
 longitude=-69.1636;
 country="Argentina";
 type="rockshelter";
 high altitude="y";
 province="southern Andes";
 technology="uniface/biface";
 megafauna kill/scavenge="n";
};
Date(Prior("Peñas de las Trampas 1.1 (Start)","PdlT1_S.prior"))
{
 site="Peñas de las Trampas 1.1";
 latitude=-26.0175;
 longitude=-67.35;
 country="Argentina";
 type="rockshelter";
 high altitude="y";
 province="southern Andes";
 technology="uniface/biface";
 megafauna kill/scavenge="n";
};
Date(Prior("San Lorenzo 1 (Start)","SLoren1_S.prior"))
{
 site="San Lorenzo 1";
 latitude=-23.4;
 longitude=-67.9;
 country="Chile";
 type="cave";
 high altitude="y";
 province="southern Andes";
 technology="uniface/biface";
 megafauna kill/scavenge="n";
};
Date(Prior("Tuina 1 (Start)","Tuina1_S.prior"))
{
 site="Tuina 1";
 latitude=-22.8;
 longitude=-68.3;
 country="Chile";
 type="rockshelter";
 high altitude="y";
 province="southern Andes";
 technology="uniface/biface";
 megafauna kill/scavenge="n";
};
Date(Prior("Tulan 109 (Start)","Tulan9_S.prior"))
{
 site="Tulan 109";
 latitude=-23.6;
 longitude=-67.9;
 country="Chile";
 type="cave";
 high altitude="y";
 province="southern Andes";
 technology="uniface/biface";
 megafauna kill/scavenge="n";
};
Date(Prior("Quereo (Start level II)","Q_S_QuereoII.prior"))
{
 site="Quereo";
 latitude=-31.93;
 longitude=-71.51;
 country="Chile";
 type="open-air";
 high altitude="n";
 province="southern Andes";
 technology="uniface/biface";
 megafauna kill/scavenge="y";
};
Date(Prior("Monte Verde II (Start MV-6)","MVII_S_MV6.prior"))
{
 site="Monte Verde II";
 latitude=-41.51;
 longitude=-73.2;
 country="Chile";
 type="open-air";
 high altitude="n";
 province="southern Andes";
 technology="uniface/biface";
 megafauna kill/scavenge="y";
};
Date(Prior("Monte Verde II (Start MV-7)","MVII_S_MV7.prior"))
{
 site="Monte Verde II";
 latitude=-41.51;
 longitude=-73.2;
 country="Chile";
 type="open-air";
 high altitude="n";
 province="southern Andes";
 technology="uniface/biface";
 megafauna kill/scavenge="y";
};
Date(Prior("El Membrillo LV.105 (Start)","Membrillo_S.prior"))
{
 site="El Membrillo LV.105";
 latitude=-31.928333;
 longitude=-71.488333;
 country="Chile";
 type="open-air";
 high altitude="n";
 province="southern Andes";
 technology="uniface/biface";
 megafauna kill/scavenge="y";
};
Date(Prior("Cueva de la Vieja (Start)","CdlV_S.prior"))
{
 site="Cueva de la Vieja";
 latitude=-45.2742;
 longitude=-71.54;
 country="Chile";
 type="cave";
 high altitude="n";
 province="southern Andes";
 technology="uniface/biface";
 megafauna kill/scavenge="n";
};
Date(Prior("Tagua Tagua 3 (start L4b facies)","TT4_S.prior"))
{
 site="Tagua Tagua 3";
 latitude=-34.4;
 longitude=-71.2;
 country="Chile";
 type="open-air";
 high altitude="n";
 province="southern Andes";
 technology="uniface/biface";
 megafauna kill/scavenge="y";
};
Date(Prior("Los Médanos 2 (Date)","LM2_D.prior"))
{
 site="Los Médanos 2";
 latitude=-26.3;
 longitude=-70.6;
 country="Chile";
 type="open-air";
 high altitude="n";
 province="southern Andes";
 technology="Huentelauquen";
 megafauna kill/scavenge="n";
};
Date(Prior("San Ramón 15 (Date)","SR15_D.prior"))
{
 site="San Ramón 15";
 latitude=-25.3;
 longitude=-70.4;
 country="Chile";
 type="open-air";
 high altitude="n";
 province="southern Andes";
 technology="Huentelauquen";
 megafauna kill/scavenge="n";
};
Date(Prior("Alero 224A (Date)","A224A_D.prior"))
{
 site="Alero 224A";
 latitude=-25.2;
 longitude=-70.4;
 country="Chile";
 type="rockshelter";
 high altitude="n";
 province="southern Andes";
 technology="Huentelauquen";
 megafauna kill/scavenge="n";
};
Date(Prior("Alero 225 (Date)","A225_D.prior"))
{
 site="Alero 225";
 latitude=-25.2;
 longitude=-70.4;
 country="Chile";
 type="rockshelter";
 high altitude="n";
 province="southern Andes";
 technology="Huentelauquen";
 megafauna kill/scavenge="n";
};
R_Date("El Trébol (Date)",10570,130)
{
 site="El Trébol";
 material="";
 latitude="-41.07";
 longitude="-71.49";
```

```
country="Argentina";
type="cave";
high altitude="n";
province="southern Andes";
technology="uniface/biface";
megafauna kill/scavenge="n";
code="AA-65707";
};
R_Date("León Huasi (Date)",10550,300)
{
site="León Huasi";
material="";
latitude="-26.3";
longitude="-70.6";
country="Chile";type="open-air";
high altitude="n";
province="southern Andes";
technology="Huentelauquén";
megafauna kill/scavenge="n";
code="GAK-13402";
};
R_Date("El Chueco 1 CIS 042 (Date)",10010,60)
{
site="El Chueco 1 CIS 042";
material="";
latitude="-44.4";
longitude="-71.30";
country="Chile";type="cave";
high altitude="n";
province="southern Andes";
technology="uniface/biface";
megafauna kill/scavenge="n";
code="Beta-227703";
};
R_Date("Río Bueno (Date)",10400,90)
{
site="Río Bueno";
material="";
latitude="-24.3";
longitude="-68.9";
country="Chile";type="open-air";
high altitude="y";
province="southern Andes";
technology="projectile point";
megafauna kill/scavenge="n";
code="No code Río Bueno";
};
R_Date("Alero Marifilo 1 (Date)",10190,120)
{
site="Alero Marifilo 1";
material="";
latitude="-39.51";
longitude="-72.06";
country="Chile";type="rockshelter";
high altitude="n";
province="southern Andes";
technology="uniface/biface";
megafauna kill/scavenge="n";
code="Beta-164475";
};
Date(Prior("Punta Purgatorio (Date)","PPurga_D.prior"))
{
site="Punta Purgatorio";
latitude=-31.963333;
longitude=-71.499444;
country="Chile";
type="open-air";
high altitude="n";
province="southern Andes";
province="southern Andes";
technology="uniface/biface";
megafauna kill/scavenge="n";
};
R_Date("Quebrada Saltillo (Date)",10260,60)
{
site="RS-I-66 Milton Almeida";
material="charcoal";
latitude="-24.3";
longitude="-68.9";
country="Chile";
type="open-air";
high altitude="y";
province=y;
technology="projectile point";
megafauna kill/scavenge="n";
code="Beta-191578";
};
R_Date("Inca Cueva 4 (Date)",10620,140)
{
site="Inca Cueva 4";
material="";
latitude="-7.06";
longitude="-79.40";
country="Peru";type="open-air";
high altitude="n";
province="southern Andes";
technology="Paiján";
megafauna kill/scavenge="n";
code="LP-137";
};
R_Date("Tuina 5 (Date)",10060,70)
{
site="Tuina 5";
material="";
latitude="-22.8";
longitude="-68.3";
country="Chile";type="cave";
high altitude="y";
province="southern Andes";
technology="uniface/biface";
megafauna kill/scavenge="n";
code="Beta-107120";
};
R_Date("La Cueva de Yavi (Date)",10450,55)
{
site="La Cueva de Yavi";
material="unreported";
latitude=-22.1;
longitude=-65.5;
country="Argentina";
type="cave";
high altitude="n";
province="southern Andes";
technology="uniface/biface";
megafauna kill/scavenge="n";
code="CSIC-1101";
};
};
Boundary("End");
};
};
```

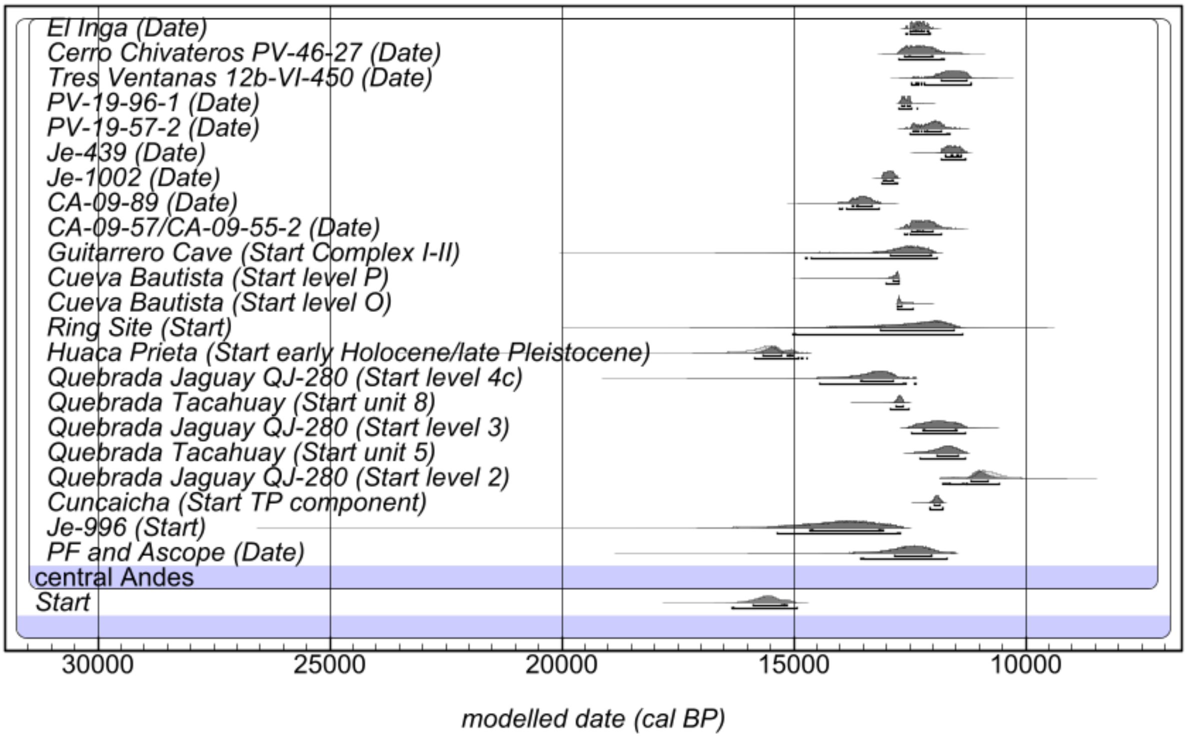


**Supplementary Figure 2.** Bayesian model showing the estimated start of cultural occupation in the central Andes (16320-14940 cal BP), including all ACR/YD-aged cultural components within this province (entered as either start boundaries or single ages given available data, and noted next to each component name). Brackets beneath each age estimate show 68.3% and 95.4% CI.

```
Plot()
{
Curve("SHCal20","shcal20.14c");
Sequence()
{
Boundary("Start");
Phase("central Andes")
{
Date(Prior("PF and Ascope (Date)","Paijan_S.prior"))
{
site="Pampa de los Fósiles and Ascope";
latitude=-7.71;
longitude=-79.1;
country="Peru";
type="open-air";
high altitude="n";
province="central Andes";
technology="Paiján";
megafauna kill/scavenge="n";
};
Date(Prior("Je-996 (Start)","Je_S.prior"))
{
site="Je-996";
latitude=-7.039;
longitude=-79.397;
country="Peru";
type="open-air";
high altitude="n";
province="central Andes";
technology="Paiján and Fishtail";
megafauna kill/scavenge="n";
};
Date(Prior("Cuncaicha (Start TP component)","Cun_S_TP.prior"))
{
site="Cuncaicha";
latitude=-15.373;
longitude=-72.618;
country="Peru";
type="rockshelter";
high altitude="y";
province="central Andes";
technology="projectile point";
megafauna kill/scavenge="n";
};
Date(Prior("Quebrada Jaguay QJ-280 (Start level 2)","QJ_S_2.prior"))
{
site="Quebrada Jaguay QJ-280";
latitude=-16.527;
longitude=-72.856;
country="Peru";
type="open-air";
high altitude="n";
province="central Andes";
technology="uniface/biface";
megafauna kill/scavenge="n";
};
Date(Prior("Quebrada Tacahuay (Start unit 5)","QT_S_5.prior"))
{
site="Quebrada Tacahuay";
latitude=-17.13;
longitude=-71.01;
country="Peru";
type="open-air";
high altitude="n";
province="central Andes";
technology="uniface/biface";
megafauna kill/scavenge="n";
};
Date(Prior("Quebrada Jaguay QJ-280 (Start level 3)","QJ_S_3.prior"))
{
site="Quebrada Jaguay QJ-280";
latitude=-16.527;
longitude=-72.856;
country="Peru";
type="open-air";
high altitude="n";
province="central Andes";
technology="uniface/biface";
megafauna kill/scavenge="n";
};
Date(Prior("Quebrada Tacahuay (Start unit 8)","QT_S_8.prior"))
{
site="Quebrada Tacahuay";
latitude=-17.13;
longitude=-71.01;
country="Peru";
type="open-air";
high altitude="n";
province="central Andes";
technology="uniface/biface";
megafauna kill/scavenge="n";
};
Date(Prior("Quebrada Jaguay QJ-280 (Start level 4c)","QJ_S_4.prior"))
{
site="Quebrada Jaguay QJ-280";
latitude=-16.527;
longitude=-72.856;
country="Peru";
type="open-air";
high altitude="n";
province="central Andes";
technology="uniface/biface";
megafauna kill/scavenge="n";
};
Date(Prior("Huaca Prieta (Start early Holocene/late Pleistocene)","HC_S_Pleis.prior"))
{
site="Huaca Prieta";
latitude=-7.9241;
longitude=-79.3069;
country="Peru";
type="open-air";
high altitude="n";
province="central Andes";
technology="uniface/biface";
megafauna kill/scavenge="n";
};
Date(Prior("Ring Site (Start)","TRS_S.prior"))
{
site="Ring Site";
latitude=-17.667;
longitude=-71.333;
country="Peru";
type="open-air";
high altitude="n";
province="central Andes";
technology="uniface/biface";
megafauna kill/scavenge="n";
};
Date(Prior("Cueva Bautista (Start level O)","CB_S_O.prior"))
{
site="Cueva Bautista";
latitude=-21.338;
longitude=-67.574;
country="Bolivia";
type="rockshelter";
high altitude="y";
province="central Andes";
technology="uniface/biface";
megafauna kill/scavenge="n";
};
Date(Prior("Cueva Bautista (Start level P)","CB_S_P.prior"))
{
site="Cueva Bautista";
latitude=-21.338;
longitude=-67.574;
country="Bolivia";
type="rockshelter";
high altitude="y";
province="central Andes";
technology="uniface/biface";
megafauna kill/scavenge="n";
};
Date(Prior("Guitarrero Cave (Start Complex I-II)","GC_S_I_II.prior"))
{
site="Guitarrero Cave";
latitude=-9.20069;
longitude=-77.7098;
country="Peru";
type="cave";
high altitude="y";
province="central Andes";
technology="uniface/biface";
megafauna kill/scavenge="n";
};
R_Date("CA-09-57/CA-09-55-2 (Date)",10360,100)
{
site="CA-09-57/CA-09-55-2";
material="";
latitude="-6.95";
longitude="-79.24";
country="Peru";type="open-air";
high altitude="n";
technology="Paiján";
megafauna kill/scavenge="n";
code="Beta-154141";
};
R_Date("CA-09-89 (Date)",11650,180)
{
site="CA-09-89";
material="";
latitude="-6.95";
longitude="-79.24";
country="Peru";type="open-air";
high altitude="n";
technology="Paiján";
megafauna kill/scavenge="n";
code="Beta-20887";
};
R_Date("Je-1002 (Date)",11014,64)
{
site="Je-1002";
material="";
latitude="-7.07";
longitude="-79.41";
country="Peru";type="open-air";
high altitude="n";
technology="Paiján";
megafauna kill/scavenge="n";
code="AA-57942";
};
R_Date("Je-439 (Date)",10056,67)
{
site="Je-439";
material="";
latitude="-22.12";
longitude="-65.47";
country="Argentina";type="cave";
high altitude="n";
technology="uniface/biface";
megafauna kill/scavenge="n";
code="AA-57950";
};
R_Date("PV-19-57-2 (Date)",10260,90)
{
site="PV-19-57-2";
material="";
latitude="-31.96";
longitude="-71.5";
country="Chile";type="open-air";
high altitude="n";
technology="uniface/biface";
megafauna kill/scavenge="n";
code="Beta-154128";
};
R_Date("PV-19-96-1 (Date)",10560,60)
{
site="PV-19-96-1";
material="";
latitude="-7.17";
longitude="-79.52";
country="Peru";type="open-air";
high altitude="n";
technology="Paiján";
megafauna kill/scavenge="n";
code="Beta-154123";
};
R_Date("Tres Ventanas 12b-VI-450 (Date)",10030,170)
{
site="Tres Ventanas 12b-VI-450";
material="";
latitude="-12.21";
longitude="-76.38";
country="Peru";type="open-air";
high altitude="n";
technology="Paiján";
megafauna kill/scavenge="n";
code="I-3091";
};
R_Date("Cerro Chivateros PV-46-27 (Date)",10430,160)
{
site="Cerro Chivateros PV-46-27";
material="";
latitude="-11.94";
```

```
  longitude="-77.13";
  country="Peru";type="open-air";
  high altitude="n";
  technology="uniface/biface";
  megafauna kill/scavenge="n";
  code="UCLA-683";
 };
 R_Date("El Inga (Date)",10410,35)
 {
  site="El Inga";
  material="charcoal";
  latitude="-0.05";
  longitude="-78.55";
  country="Ecuador";type="open-air";
  high altitude="y";
  technology="Fishtail";
  megafauna kill/scavenge="n";
  code="PRI-13-029";
 };
 };
 Boundary("End");
 };
};
```

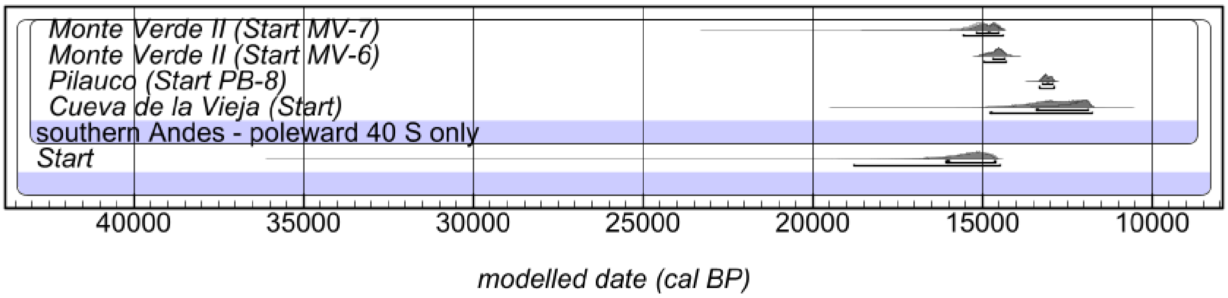


**Supplementary Figure 3.** Bayesian model showing the estimated start of cultural occupation in the southern Andes (poleward of 40° S, only; 18795-14460 cal BP), including all ACR/YD-aged cultural components within this province (entered as start boundaries). Brackets beneath each age estimate show 68.3% and 95.4% CI.

```
Plot()
{
 Sequence()
 {
  Boundary("Start");
  Phase("southern Andes - poleward 40 S only")
  {
   Date(Prior("Cueva de la Vieja (Start)","CdlV_S.prior"))
   {
    site="Cueva de la Vieja";
    latitude=-42.274167;
    longitude=-71.54;
    country="Chile";
    type="cave";
    high altitude="n";
    province="southern Andes";
    technology="uniface/biface";
    megafauna kill/scavenge="y";
   };
   Date(Prior("Pilauco (Start PB-8)","Pil_PB8_S.prior"))
   {
    site="Pilauco";
    latitude=-40.566667;
    longitude=-73.116667;
    country="Chile";
    type="open-air";
    high altitude="n";
    province="southern Andes";
    technology="uniface/biface";
    megafauna kill/scavenge="n";
   };
   Date(Prior("Monte Verde II (Start MV-6)","MVII_S_MV6.prior"))
   {
    site="Monte Verde II";
    latitude=-41.51;
    longitude=-73.2;
    country="Chile";
    type="open-air";
    high altitude="n";
    province="southern Andes";
    technology="uniface/biface";
    megafauna kill/scavenge="y";
   };
   Date(Prior("Monte Verde II (Start MV-7)","MVII_S_MV7.prior"))
   {
    site="Monte Verde II";
    latitude=-41.51;
    longitude=-73.2;
    country="Chile";
    type="open-air";
    high altitude="n";
    province="southern Andes";
    technology="uniface/biface";
    megafauna kill/scavenge="y";
   };
  };
  Boundary("End");
 };
};
```

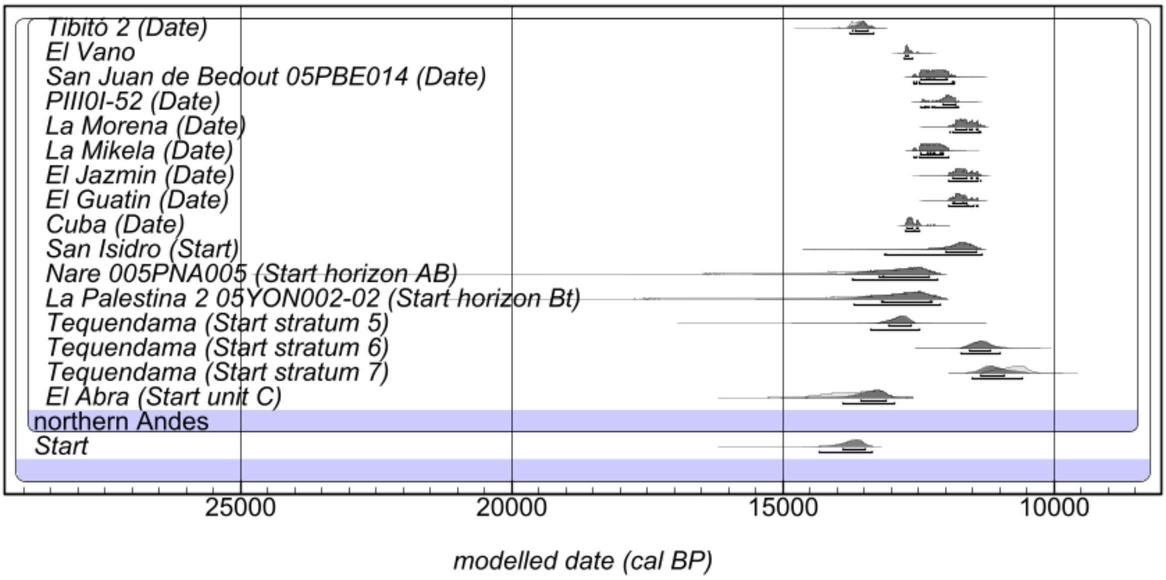


**Supplementary Figure 4.** Bayesian model showing the estimated start of cultural occupation in the northern Andes (14325-13350 cal BP), including all ACR/YD-aged cultural components within this

province (entered as either start boundaries or single ages given available data, and noted next to each component name). Brackets beneath each age estimate show 68.3% and 95.4% CI.

```
Plot()
{
 Sequence()
 {
  Boundary("Start");
  Phase("northern Andes")
  {
   Date(Prior("El Abra (Start unit C)","EA_S_C.prior"))
   {
    site="El Abra";
    latitude=5.02;
    longitude=-73.937;
    country="Colombia";
    type="rockshelter";
    high altitude="n";
    province="northern Andes";
    technology="Abriense";
    megafauna kill/scavenge="n";
   };
   Date(Prior("Tequendama (Start stratum 7)","Teq_S_7.prior"))
   {
    site="Tequendama";
    latitude=4.533;
    longitude=-74.275;
    country="Colombia";
    type="rockshelter";
    high altitude="n";
    province="northern Andes";
    technology="Tequendamiense";
    megafauna kill/scavenge="y";
   };
   Date(Prior("Tequendama (Start stratum 6)","Teq_S_6.prior"))
   {
    site="Tequendama";
    latitude=4.533;
    longitude=-74.275;
    country="Colombia";
    type="rockshelter";
    high altitude="n";
    province="northern Andes";
    technology="Tequendamiense";
    megafauna kill/scavenge="y";
   };
   Date(Prior("Tequendama (Start stratum 5)","Teq_S_5.prior"))
   {
    site="Tequendama";
    latitude=4.533;
    longitude=-74.275;
    country="Colombia";
    type="rockshelter";
    high altitude="n";
    province="northern Andes";
    technology="Tequendamiense";
    megafauna kill/scavenge="y";
   };
   Date(Prior("La Palestina 2 05YON002-02 (Start horizon Bt)","LP2_S_Bt.prior"))
   {
    site="La Palestina 2 05YON002-02";
    latitude=6.716;
    longitude=-74.383;
    country="Colombia";
    type="open-air";
    high altitude="n";
    province="northern Andes";
    technology="uniface/biface";
    megafauna kill/scavenge="n";
   };
   Date(Prior("Nare 005PNA005 (Start horizon AB)","Nare_S.prior"))
   {
    site="Nare 005PNA005";
    latitude=6.35;
    longitude=-74.1;
    country="Colombia";
    type="open-air";
    high altitude="n";
    province="northern Andes";
    technology="uniface/biface";
    megafauna kill/scavenge="n";
   };
   Date(Prior("San Isidro (Start)","SIsi_S.prior"))
   {
    site="San Isidro";
    latitude=2.4;
    longitude=-76.5;
    country="Colombia";
    type="open-air";
    high altitude="n";
    province="northern Andes";
    technology="uniface/biface";
    megafauna kill/scavenge="n";
   };
   R_Date("Cuba (Date)",10619,66)
   {
    site="Cuba ";
    material="charcoal";
    latitude="5.197776";
    longitude="-75.676397";
    country="Colombia";type="open-air";
    high altitude="n";
    technology="uniface/biface";
    megafauna kill/scavenge="n";
    code="AA-102510";
   };
   R_Date("El Guatin (Date)",10130,50)
   {
    site="El Guatin";
    material="charcoal";
    latitude="4.75";
    longitude="-75.7";
    country="Colombia";type="open-air";
    high altitude="n";
    technology="uniface/biface";
    megafauna kill/scavenge="n";
    code="Beta-325213";
   };
   R_Date("El Jazmin (Date)",10120,70)
   {
    site="El Jazmin";
    material="charcoal";
    latitude="3.8";
    longitude="-76.2";
    country="Colombia";type="open-air";
    high altitude="n";
    technology="uniface/biface";
    megafauna kill/scavenge="n";
    code="unreported";
   };
   R_Date("La Mikela (Date)",10376,70)
   {
    site="La Mikela";
    material="charcoal";
    latitude="4.781956";
    longitude="-75.68447";
    country="Colombia";type="open-air";
    high altitude="n";
    technology="uniface/biface";
    megafauna kill/scavenge="n";
    code="AA-98939";
   };
   R_Date("La Morena (Date)",10090,60)
   {
    site="La Morena";
    material="unreported";
    latitude="6.2";
    longitude="-75.5";
    country="Colombia";type="open-air";
    high altitude="n";
    technology="uniface/biface";
    megafauna kill/scavenge="n";
    code="unreported";
   };
   R_Date("PIII0I-52 (Date)",10260,50)
   {
    site="PIII0I-52";
    material="unreported";
    latitude="6.8";
    longitude="-75.1";
    country="Colombia";type="open-air";
    high altitude="n";
    technology="uniface/biface";
    megafauna kill/scavenge="n";
    code="unreported";
   };
   R_Date("San Juan de Bedout 05PBE014 (Date)",10350,90)
   {
    site="San Juan de Bedout 05PBE014";
    material="";
    latitude="6.5";
    longitude="-74.5";
    country="Colombia";type="open-air";
    high altitude="n";
    technology="uniface/biface";
    megafauna kill/scavenge="n";
    code="no code San Juan";
   };
   R_Date("El Vano",10710,60)
   {
    site="El Vano";
    material="unreported";
    latitude="9.62";
    longitude="-70";
    country="Venezuela";type="open-air";
    high altitude="n";
    technology="El Jobo";
    megafauna kill/scavenge="y";
    code="Beta-95602";
   };
   R_Date("Tibitó 2 (Date)",11740,110)
   {
    site="Tibitó 2";
    material="";
    latitude="4.98";
    longitude="-73.98";
    country="Colombia";type="open-air";
    high altitude="n";
    technology="Abriense";
    megafauna kill/scavenge="y";
    code="GrN-9375";
   };
  };
  Boundary("End");
 };
};
```

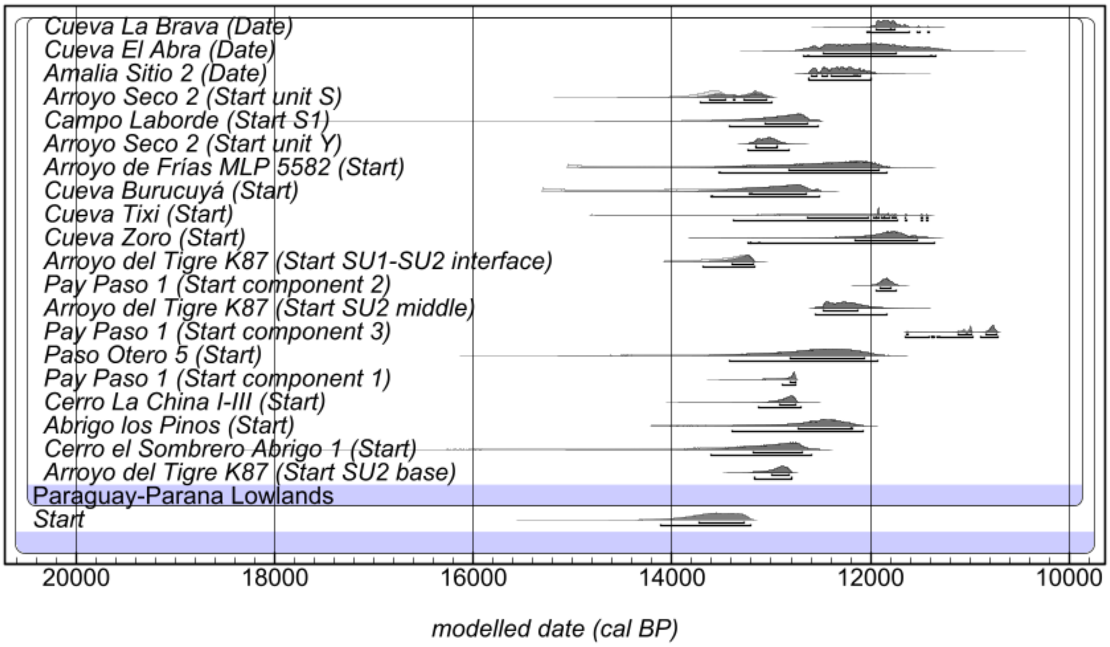


**Supplementary Figure 5.** Bayesian model showing the estimated start of cultural occupation in the Paraguay-Parana Lowlands (14115-13200 cal BP), including all ACR/YD-aged cultural components within this province (entered as either start boundaries or single ages given available data, and noted next to each component name). Brackets beneath each age estimate show 68.3% and 95.4% CI.

```
Plot()
{
 Curve("SHCal20","shcal20.14c");
 Sequence()
 {
  Boundary("Start");
  Phase("Paraguay-Parana Lowlands")
  {
   Date(Prior("Arroyo del Tigre K87 (Start SU2 base)","K87_S_base.prior"))
   {
    site="Arroyo del Tigre K87";
    latitude=-30.4;
    longitude=-57.8;
    country="Uruguay";
    type="open-air";
    high altitude="n";
    province="Paraguay-Parana Lowlands";
    technology="Fishtail";
    megafauna kill/scavenge="n";
   };
   Date(Prior("Cerro el Sombrero Abrigo 1 (Start)","CSA1_S.prior"))
   {
    site="Cerro el Sombrero Abrigo 1";
    latitude=-37.8166;
    longitude=-58.5666;
    country="Argentina";
    type="rockshelter";
    high altitude="n";
    province="Paraguay-Parana Lowlands";
    technology="Fishtail";
    megafauna kill/scavenge="n";
   };
   Date(Prior("Abrigo los Pinos (Start)","AlP_S.prior"))
   {
    site="Abrigo los Pinos";
    latitude=-37.4;
    longitude=-58.9;
    country="Argentina";
    type="rockshelter";
    high altitude="n";
    province="Paraguay-Parana Lowlands";
    technology="Fishtail";
    megafauna kill/scavenge="n";
   };
   Date(Prior("Cerro La China I-III (Start)","CLC_S.prior"))
   {
    site="Cerro La China I-III";
    latitude=-37.95;
    longitude=-58.6166;
    country="Argentina";
    type="rockshelter";
    high altitude="n";
    province="Paraguay-Parana Lowlands";
    technology="Fishtail";
    megafauna kill/scavenge="n";
   };
   Date(Prior("Pay Paso 1 (Start component 1)","PP_S_1.prior"))
   {
    site="Pay Paso 1";
    latitude=-30.2685;
    longitude=-57.460639;
    country="Uruguay";
    type="open-air";
    high altitude="n";
    province="Paraguay-Parana Lowlands";
    technology="Fishtail";
    megafauna kill/scavenge="y";
   };
   Date(Prior("Paso Otero 5 (Start)","PO5_S.prior"))
   {
    site="Paso Otero 5";
    latitude=-38.2022;
    longitude=-59.1327;
    country="Argentina";
    type="open-air";
    high altitude="n";
    province="Paraguay-Parana Lowlands";
    technology="Fishtail";
    megafauna kill/scavenge="y";
   };
   Date(Prior("Pay Paso 1 (Start component 3)","PP_S_3.prior"))
   {
    site="Pay Paso 1";
    latitude=-30.2685;
    longitude=-57.460639;
    country="Uruguay";
    type="open-air";
    high altitude="n";
    province="Paraguay-Parana Lowlands";
    technology="Pay Paso";
    megafauna kill/scavenge="y";
   };
   Date(Prior("Arroyo del Tigre K87 (Start SU2 middle)","K87_S_mid.prior"))
   {
    site="Arroyo del Tigre K87";
    latitude=-30.4;
    longitude=-57.8;
    country="Uruguay";
    type="open-air";
    high altitude="n";
    province="Paraguay-Parana Lowlands";
    technology="Tigre";
    megafauna kill/scavenge="n";
   };
   Date(Prior("Pay Paso 1 (Start component 2)","PP_S_2.prior"))
   {
    site="Pay Paso 1";
    latitude=-30.2685;
    longitude=-57.460639;
    country="Uruguay";
    type="open-air";
    high altitude="n";
    province="Paraguay-Parana Lowlands";
    technology="Tigre";
    megafauna kill/scavenge="y";
   };
   Date(Prior("Arroyo del Tigre K87 (Start SU1-SU2 interface)","K87_S_1_2.prior"))
   {
    site="Arroyo del Tigre K87";
    latitude=-30.4;
    longitude=-57.8;
    country="Uruguay";
    type="open-air";
    high altitude="n";
    province="Paraguay-Parana Lowlands";
    technology="uniface/biface";
    megafauna kill/scavenge="n";
   };
   Date(Prior("Cueva Zoro (Start)","CZ_S.prior"))
   {
    site="Cueva Zoro";
    latitude=-37.8166;
    longitude=-58.6333;
    country="Argentina";
    type="rockshelter";
    high altitude="n";
    province="Paraguay-Parana Lowlands";
    technology="uniface/biface";
    megafauna kill/scavenge="n";
   };
   Date(Prior("Cueva Tixi (Start)","CTixi_S.prior"))
   {
    site="Cueva Tixi";
    latitude=-37.4;
    longitude=-58.9;
    country="Argentina";
    type="cave";
    high altitude="n";
    province="Paraguay-Parana Lowlands";
    technology="uniface/biface";
    megafauna kill/scavenge="n";
   };
   Date(Prior("Cueva Burucuyá (Start)","CBuru_S.prior"))
   {
    site="Cueva Burucuyá";
    latitude=-37.8;
    longitude=-58.1;
    country="Argentina";
    type="cave";
    high altitude="n";
    province="Paraguay-Parana Lowlands";
    technology="uniface/biface";
    megafauna kill/scavenge="n";
   };
   Date(Prior("Arroyo de Frías MLP 5582 (Start)","AdF_S.prior"))
   {
    site="Arroyo de Frías MLP 5582";
    latitude=-34.65;
    longitude=-59.4307;
    country="Argentina";
    type="open-air";
    high altitude="n";
    province="Paraguay-Parana Lowlands";
    technology="uniface/biface";
    megafauna kill/scavenge="n";
   };
   Date(Prior("Arroyo Seco 2 (Start unit Y)","AS2_S_unitY.prior"))
   {
    site="Arroyo Seco 2";
    latitude=-38.360556;
    longitude=-60.244167;
    country="Argentina";
    type="open-air";
    high altitude="n";
    province="Paraguay-Parana Lowlands";
    technology="uniface/biface";
    megafauna kill/scavenge="y";
   };
   Date(Prior("Campo Laborde (Start S1)","CL_S_1.prior"))
   {
    site="Campo Laborde";
    latitude=-37.1;
    longitude=-60.38;
    country="Argentina";
    type="open-air";
    high altitude="n";
    province="Paraguay-Parana Lowlands";
    technology="uniface/biface";
    megafauna kill/scavenge="y";
   };
   Date(Prior("Arroyo Seco 2 (Start unit S)","AS2_S_unitS.prior"))
   {
    site="Arroyo Seco 2";
    latitude=-38.360556;
    longitude=-60.244167;
    country="Argentina";
    type="open-air";
    high altitude="n";
    province="Paraguay-Parana Lowlands";
    technology="uniface/biface";
    megafauna kill/scavenge="y";
   };
   R_Date("Amalia Sitio 2 (Date)",10425,75)
   {
    site="Amalia Sitio 2";
    material="";
    latitude="-37.8";
    longitude="-58.10";
    country="Argentina";type="rockshelter";
    high altitude="n";
    technology="Fishtail";
    megafauna kill/scavenge="n";
    code="AA-35499";
   };
   R_Date("Cueva El Abra (Date)",10270,200)
   {
    site="Cueva El Abra";
    material="";
    latitude="-37.4";
    longitude="-58.90";
    country="Argentina";type="cave";
    high altitude="n";
    technology="uniface/biface";
    megafauna kill/scavenge="n";
    code="AA-94641";
   };
   R_Date("Cueva La Brava (Date)",10178,54)
   {
    site="Cueva La Brava";
    material="";
    latitude="-37.8";
    longitude="-58.1";
    country="Argentina";type="cave";
    high altitude="n";
    technology="uniface/biface";
    megafauna kill/scavenge="n";
    code="AA-9463";
   };
  };
  Boundary("End");
 };
};
```

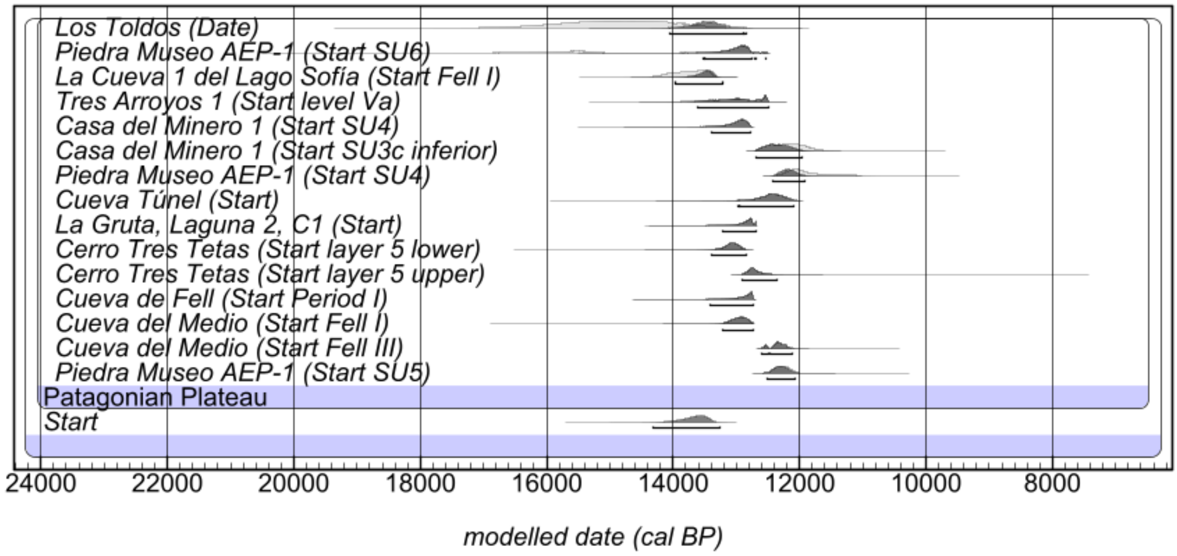


**Supplementary Figure 6.** Bayesian model showing the estimated start of cultural occupation in the Patagonian Plateau (14310-13265 cal BP), including all ACR/YD-aged cultural components within this province (entered as either start boundaries or single ages given available data, and noted next to each component name). Brackets beneath each age estimate show 68.3% and 95.4% CI.

```
Plot()
{
 Curve("SHCal20","shcal20.14c");
 Sequence()
 {
  Boundary("Start");
  Phase("Patagonian Plateau")
  {
   Date(Prior("Piedra Museo AEP-1 (Start SU5)","PM_S_SU5.prior"))
   {
    site="Piedra Museo AEP-1";
    latitude=-47.895;
    longitude=-67.867778;
    country="Argentina";
    type="rockshelter";
    high altitude="n";
    province="Patagonian Plateau";
    technology="Fishtail";
    megafauna kill/scavenge="y";
   };
   Date(Prior("Cueva del Medio (Start Fell III)","CM_S_Fell_III.prior"))
   {
    site="Cueva del Medio";
    latitude=-51.576;
    longitude=-72.583;
    country="Chile";
    type="cave";
    high altitude="n";
    province="Patagonian Plateau";
    technology="Fishtail";
    megafauna kill/scavenge="y";
   };
   Date(Prior("Cueva del Medio (Start Fell I)","CM_S_Fell_I.prior"))
   {
    site="Cueva del Medio";
    latitude=-51.576;
    longitude=-72.583;
    country="Chile";
    type="cave";
    high altitude="n";
    province="Patagonian Plateau";
    technology="Fishtail";
    megafauna kill/scavenge="y";
   };
   Date(Prior("Cueva de Fell (Start Period I)","CF_S_Fell.prior"))
   {
    site="Cueva de Fell";
    latitude=-52.044444;
    longitude=-70.056389;
    country="Chile";
    type="rockshelter";
    high altitude="n";
    province="Patagonian Plateau";
    technology="Fishtail";
    megafauna kill/scavenge="y";
   };
   Date(Prior("Cerro Tres Tetas (Start layer 5 upper)","C3T_S_layer5upper.prior"))
   {
    site="Cerro Tres Tetas";
    latitude=-48.149444;
    longitude=-68.933333;
    country="Argentina";
    type="open-air";
    high altitude="n";
    province="Patagonian Plateau";
    technology="uniface/biface";
    megafauna kill/scavenge="n";
   };
   Date(Prior("Cerro Tres Tetas (Start layer 5 lower)","C3T_S_layer5lower.prior"))
   {
    site="Cerro Tres Tetas";
    latitude=-48.149444;
    longitude=-68.933333;
    country="Argentina";
    type="open-air";
    high altitude="n";
    province="Patagonian Plateau";
    technology="uniface/biface";
    megafauna kill/scavenge="n";
   };
   Date(Prior("La Gruta, Laguna 2, C1 (Start)","LG_S.prior"))
   {
    site="La Gruta, Laguna 2, C1";
    latitude=-48.8261;
    longitude=-69.3961;
    country="Argentina";
    type="cave";
    high altitude="n";
    province="Patagonian Plateau";
    technology="uniface/biface";
    megafauna kill/scavenge="n";
   };
   Date(Prior("Cueva Túnel (Start)","CTunel_S.prior"))
   {
    site="Cueva Túnel";
    latitude=-48.4575;
    longitude=-68.8725;
    country="Argentina";
    type="cave";
    high altitude="n";
    province="Patagonian Plateau";
    technology="uniface/biface";
    megafauna kill/scavenge="n";
   };
   Date(Prior("Piedra Museo AEP-1 (Start SU4)","PM_S_SU4.prior"))
   {
    site="Piedra Museo AEP-1";
    latitude=-47.895;
    longitude=-67.867778;
    country="Argentina";
    type="rockshelter";
    high altitude="n";
    province="Patagonian Plateau";
    technology="uniface/biface";
    megafauna kill/scavenge="y";
   };
   Date(Prior("Casa del Minero 1 (Start SU3c inferior)","CM1_S_SU3c.prior"))
   {
    site="Casa del Minero 1";
    latitude=-48.575833;
    longitude=-68.9125;
    country="Argentina";
    type="cave";
    high altitude="n";
    province="Patagonian Plateau";
    technology="uniface/biface";
    megafauna kill/scavenge="y";
   };
   Date(Prior("Casa del Minero 1 (Start SU4)","CM1_S_SU4.prior"))
   {
    site="Casa del Minero 1";
    latitude=-48.575833;
    longitude=-68.9125;
    country="Argentina";
    type="cave";
    high altitude="n";
    province="Patagonian Plateau";
    technology="uniface/biface";
    megafauna kill/scavenge="y";
   };
   Date(Prior("Tres Arroyos 1 (Start level Va)","TA1_S_Va.prior"))
   {
    site="Tres Arroyos 1";
    latitude=-53.383;
    longitude=-68.783;
    country="Chile";
    type="rockshelter";
    high altitude="n";
    province="Patagonian Plateau";
    technology="uniface/biface";
    megafauna kill/scavenge="y";
   };
   Date(Prior("La Cueva 1 del Lago Sofía (Start Fell I)","CLS_S_Fell_I.prior"))
   {
    site="La Cueva 1 del Lago Sofía";
    latitude=-51.53;
    longitude=-72.53;
    country="Chile";
    type="cave";
    high altitude="n";
    province="Patagonian Plateau";
    technology="uniface/biface";
    megafauna kill/scavenge="y";
   };
   Date(Prior("Piedra Museo AEP-1 (Start SU6)","PM_S_SU6.prior"))
   {
    site="Piedra Museo AEP-1";
    latitude=-47.895;
    longitude=-67.867778;
    country="Argentina";
    type="rockshelter";
    high altitude="n";
    province="Patagonian Plateau";
    technology="uniface/biface";
    megafauna kill/scavenge="y";
   };
   R_Date("Los Toldos (Date)",12600,600)
   {
    site="Los Toldos";
    material="";
    latitude="-47.9";
    longitude="-67.87";
    country="Argentina";type="cave";
    high altitude="n";
    technology="uniface/biface";
    megafauna kill/scavenge="n";
    code="no code Los Toldos";
   };
  };
  Boundary("End");
 };
};
```

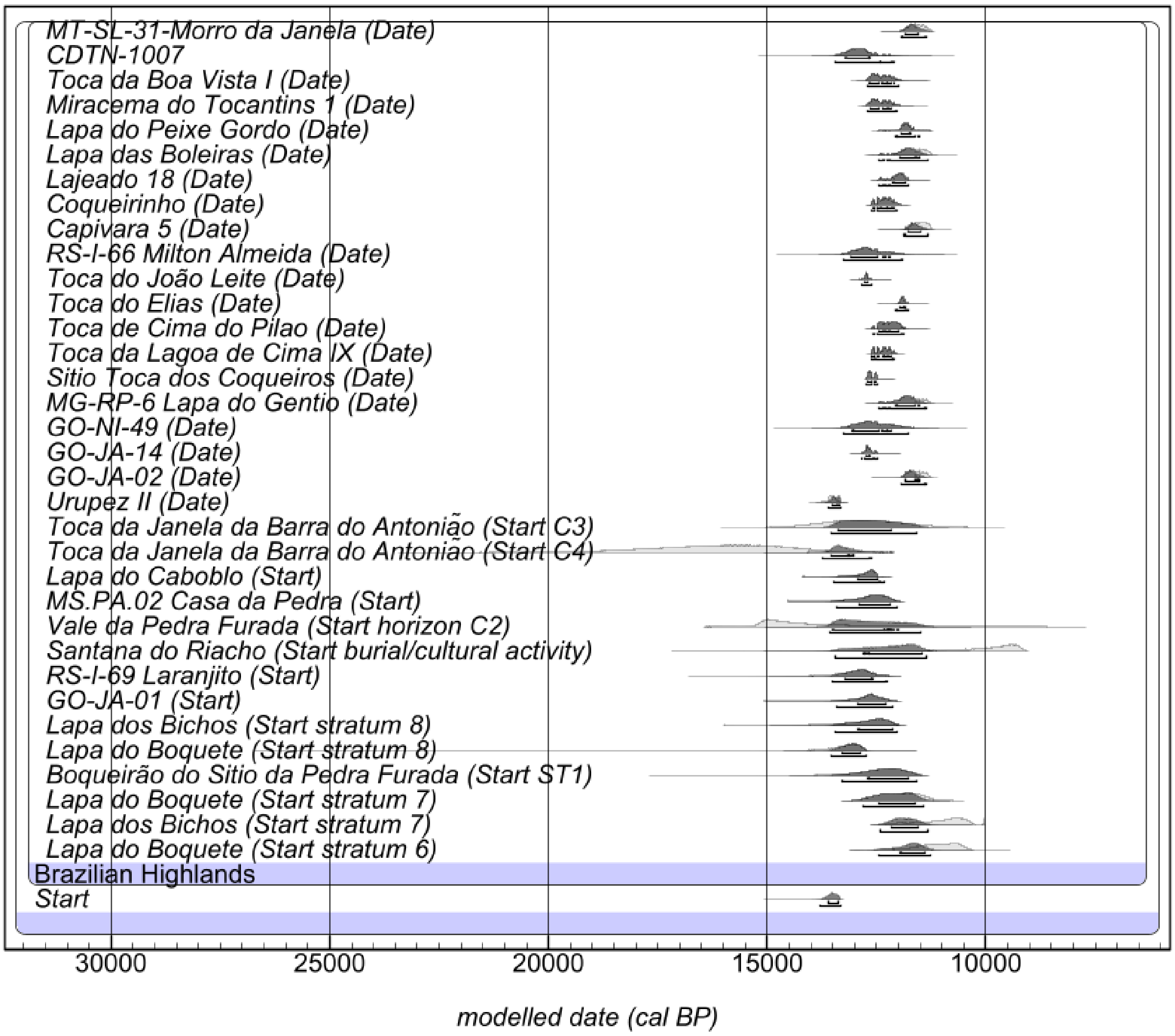


**Supplementary Figure 7.** Bayesian model showing the estimated start of cultural occupation in the Brazilian Highlands (13800-13315 cal BP), including all ACR/YD-aged cultural components within this province (entered as either start boundaries or single ages given available data, and noted next to each component name). Brackets beneath each age estimate show 68.3% and 95.4% CI.

```
Plot()
{
 Curve("SHCal20","shcal20.14c");
 Sequence()
 {
  Boundary("Start");
  Phase("Brazilian Highlands")
  {
   Date(Prior("Lapa do Boquete (Start stratum 6)","LBo_S_6.prior"))
   {
    site="Lapa do Boquete";
    latitude=-14.98;
    longitude=-44.44;
    country="Brazil";
    type="cave";
    high altitude="n";
    province="Brazilian Highlands";
    technology="Itaparica";
    megafauna kill/scavenge="n";
   };
   Date(Prior("Lapa dos Bichos (Start stratum 7)","LBi_S_7.prior"))
   {
    site="Lapa dos Bichos";
    latitude=-14.98;
    longitude=-44.44;
    country="Brazil";
    type="cave";
    high altitude="n";
    province="Brazilian Highlands";
    technology="Itaparica";
    megafauna kill/scavenge="n";
   };
   Date(Prior("Lapa do Boquete (Start stratum 7)","LBo_S_7.prior"))
   {
    site="Lapa do Boquete";
    latitude=-14.98;
    longitude=-44.44;
    country="Brazil";
    type="cave";
    high altitude="n";
    province="Brazilian Highlands";
    technology="Itaparica";
    megafauna kill/scavenge="n";
   };
   Date(Prior("Boqueirão do Sitio da Pedra Furada (Start ST1)","PF_S_ST1.prior"))
   {
    site="Boqueirão do Sitio da Pedra Furada";
    latitude=-8.85;
    longitude=-42.55;
    country="Brazil";
    type="rockshelter";
    high altitude="n";
    province="Brazilian Highlands";
    technology="Itaparica";
    megafauna kill/scavenge="n";
   };
   Date(Prior("Lapa do Boquete (Start stratum 8)","LBo_S_8.prior"))
   {
    site="Lapa do Boquete";
    latitude=-14.98;
    longitude=-44.44;
    country="Brazil";
    type="cave";
    high altitude="n";
    province="Brazilian Highlands";
    technology="Itaparica";
    megafauna kill/scavenge="n";
   };
   Date(Prior("Lapa dos Bichos (Start stratum 8)","LBi_S_8.prior"))
   {
    site="Lapa dos Bichos";
    latitude=-14.98;
    longitude=-44.44;
    country="Brazil";
    type="cave";
    high altitude="n";
    province="Brazilian Highlands";
    technology="Itaparica";
    megafauna kill/scavenge="n";
   };
   Date(Prior("GO-JA-01 (Start)","GOJA01_S.prior"))
   {
    site="GO-JA-01";
    latitude=-18.28638889;
    longitude=-52.04777778;
    country="Brazil";
    type="rockshelter";
    high altitude="n";
    province="Brazilian Highlands";
    technology="Itaparica";
    megafauna kill/scavenge="n";
   };
   Date(Prior("RS-I-69 Laranjito (Start)","RSI_S.prior"))
   {
    site="RS-I-69 Laranjito";
    latitude=-29.61333333;
    longitude=-56.93;
    country="Brazil";
    type="open-air";
    high altitude="n";
    province="Brazilian Highlands";
    technology="projectile point";
    megafauna kill/scavenge="n";
   };
   Date(Prior("Santana do Riacho (Start burial/cultural activity)","SR_S_humans.prior"))
   {
    site="Santana do Riacho";
    latitude=-19.2;
    longitude=-43.7;
    country="Brazil";
    type="rockshelter";
    high altitude="n";
    province="Brazilian Highlands";
    technology="uniface/biface";
    megafauna kill/scavenge="n";
   };
   Date(Prior("Vale da Pedra Furada (Start horizon C2)","VPF_S_C2.prior"))
   {
    site="Vale da Pedra Furada";
    latitude=-8.85;
    longitude=-42.55;
    country="Brazil";
    type="open-air";
    high altitude="n";
    province="Brazilian Highlands";
    technology="uniface/biface";
    megafauna kill/scavenge="n";
   };
   Date(Prior("MS.PA.02 Casa da Pedra (Start)","MSPA_S.prior"))
   {
    site="MS.PA.02 Casa da Pedra";
    latitude=-19.58333333;
    longitude=-52.66666667;
    country="Brazil";
    type="cave";
    high altitude="n";
    province="Brazilian Highlands";
    technology="uniface/biface";
    megafauna kill/scavenge="n";
   };
   Date(Prior("Lapa do Caboblo (Start)","LdCabo_S.prior"))
   {
    site="Lapa do Caboblo";
    latitude=-18.28527778;
    longitude=-43.85388889;
    country="Brazil";
    type="rockshelter/cave";
    high altitude="n";
    province="Brazilian Highlands";
    technology="uniface/biface";
    megafauna kill/scavenge="n";
   };
   Date(Prior("Toca da Janela da Barra do Antonião (Start C4)","TDJ_S_C4.prior"))
   {
    site="Toca da Janela da Barra do Antonião";
    latitude=-8.802222;
    longitude=-42.433056;
    country="Brazil";
    type="rockshelter";
    high altitude="n";
    province="Brazilian Highlands";
    technology="uniface/biface";
    megafauna kill/scavenge="n";
   };
   Date(Prior("Toca da Janela da Barra do Antonião (Start C3)","TDJ_S_C3.prior"))
   {
    site="Toca da Janela da Barra do Antonião";
    latitude=-8.802222;
    longitude=-42.433056;
    country="Brazil";
    type="rockshelter";
    high altitude="n";
    province="Brazilian Highlands";
    technology="Itaparica";
    megafauna kill/scavenge="n";
   };
   R_Date("Urupez II (Date)",11690,80)
   {
    site="Urupez II";
    material="";
    latitude="-34.83";
    longitude="-55.32";
    country="Uruguay";type="open-air";
    high altitude="n";
    technology="Fishtail";
    megafauna kill/scavenge="n";
    code="Beta-211398";
   };
   R_Date("GO-JA-02 (Date)",10120,80)
   {
    site="GO-JA-02";
    material="";
    latitude="-18.45";
    longitude="-52.01";
    country="Brazil";type="rockshelter";
    high altitude="n";
    technology="Itaparica";
    megafauna kill/scavenge="n";
    code="SI-3108";
   };
   R_Date("GO-JA-14 (Date)",10740,85)
   {
    site="GO-JA-14";
    material="";
    latitude="-14.48";
    longitude="-49.47";
    country="Brazil";type="rockshelter";
    high altitude="n";
    technology="Itaparica";
    megafauna kill/scavenge="n";
    code="SI-3111";
   };
   R_Date("GO-NI-49 (Date)",10750,300)
   {
    site="GO-NI-49";
    material="";
    latitude="-23.00";
    longitude="-65.45";
    country="Argentina";type="rockshelter";
    high altitude="y";
    technology="uniface/biface";
    megafauna kill/scavenge="n";
    code="SI-2769";
   };
   R_Date("MG-RP-6 Lapa do Gentio (Date)",10190,120)
   {
    site="MG-RP-6 Lapa do Gentio";
    material="";
    latitude="-16.26";
    longitude="-46.05";
    country="Brazil";type="rockshelter";
    high altitude="n";
    technology="Itaparica";
    megafauna kill/scavenge="n";
    code="SI-6837";
   };
   R_Date("Sitio Toca dos Coqueiros (Date)",10640,50)
   {
    site="Sitio Toca dos Coqueiros";
    material="";
    latitude="-29.72";
    longitude="-56.66";
    country="Brazil";type="open-air";
    high altitude="n";
    technology="projectile point";
    megafauna kill/scavenge="n";
    code="Beta-104571";
   };
   R_Date("Toca da Lagoa de Cima IX (Date)",10480,50)
   {
    site="Toca da Lagoa de Cima IX";
    material="";
    latitude="-8.7";
```

```
  longitude="-42.6";
  country="Brazil";type="rockshelter/cave";
  high altitude="n";
  technology="Itaparica";
  megafauna kill/scavenge="n";
  code="Beta-233909";
 };
 R_Date("Toca de Cima do Pilao (Date)",10390,80)
 {
  site="Toca de Cima do Pilao";
  material="";
  latitude="-8.87";
  longitude="-42.59";
  country="Brazil";type="rockshelter/cave";
  high altitude="n";
  technology="Itaparica";
  megafauna kill/scavenge="n";
  code="Beta-27345";
 };
 R_Date("Toca do Elias (Date)",10270,35)
 {
  site="Toca do Elias";
  material="";
  latitude="-8.84";
  longitude="-42.56";
  country="Brazil";type="rockshelter/cave";
  high altitude="n";
  technology="Itaparica";
  megafauna kill/scavenge="n";
  code="CAMS-95865";
 };
 R_Date("Toca do João Leite (Date)",10800,70)
 {
  site="Toca do João Leite";
  material="";
  latitude="-8.74";
  longitude="-42.74";
  country="Brazil";type="rockshelter/cave";
  high altitude="n";
  technology="Itaparica";
  megafauna kill/scavenge="n";
  code="Beta-220088";
 };
 R_Date("RS-I-66 Milton Almeida (Date)",10810,275)
 {
  site="RS-I-66 Milton Almeida";
  material="unreported";
  latitude="-29.71805556";
  longitude="-56.66388889";
  country="Brazil";
  type="open-air";
  high altitude="n";
  province=Brazilian Highlands;
  technology="projectile point";
  megafauna kill/scavenge="n";
  code="SI-2722";
 };
 R_Date("Capivara 5 (Date)",10050,80)
 {
  site="Capivara 5";
  material="";
  latitude="-10.14";
  longitude="-48.44";
  country="Brazil";type="open-air";
  high altitude="n";
  technology="uniface/biface";
  megafauna kill/scavenge="n";
  code="Beta-179196";
 };
 R_Date("Coqueirinho (Date)",10460,60)
 {
  site="Coqueirinho";
  material="";
  latitude="-19.54";
  longitude="-43.95";
  country="Brazil";type="open-air";
  high altitude="n";
  technology="uniface/biface";
  megafauna kill/scavenge="n";
  code="Beta-237346";
 };
 R_Date("Lajeado 18 (Date)",10300,60)
 {
  site="Lajeado 18";
  material="";
  latitude="-19.52";
  longitude="-44.07";
  country="Brazil";type="rockshelter/cave";
  high altitude="n";
  technology="uniface/biface";
  megafauna kill/scavenge="n";
  code="Beta-179198";
 };
 R_Date("Lapa das Boleiras (Date)",10150,130)
 {
  site="Lapa das Boleiras";
  material="";
  latitude="-18.3";
  longitude="-43.74";
  country="Brazil";type="rockshelter/cave";
  high altitude="n";
  technology="uniface/biface";
  megafauna kill/scavenge="n";
  code="Beta-168451";
 };
 R_Date("Lapa do Peixe Gordo (Date)",10210,60)
 {
  site="Lapa do Peixe Gordo";
  material="";
  latitude="-28.6";
  longitude="-65.6";
  country="Argentina";type="rockshelter";
  high altitude="n";
  technology="uniface/biface";
  megafauna kill/scavenge="n";
  code="Beta-233762";
 };
 R_Date("Miracema do Tocantins 1 (Date)",10530,90)
 {
  site="Miracema do Tocantins 1";
  material="";
  latitude="-9.64";
  longitude="-48.41";
  country="Brazil";type="open-air";
  high altitude="n";
  technology="uniface/biface";
  megafauna kill/scavenge="n";
  code="Beta-190080";
 };
 R_Date("Toca da Boa Vista I (Date)",10530,110)
 {
  site="Toca da Boa Vista I";
  material="";
  latitude="-8.63";
  longitude="-42.36";
  country="Brazil";type="rockshelter/cave";
  high altitude="n";
  technology="uniface/biface";
  megafauna kill/scavenge="n";
  code="Beta-32971";
 };
R_Date("Lapa do Dragao (Date)",11000,300)
{
 site="Lapa do Dragao";
 material="";
 latitude="-14.4167";
 longitude="-44.4";
 country="Brazil";type="rockshelter";
 high altitude="n";
 province=Brazilian Highlands;
 technology="uniface/biface";
 megafauna kill/scavenge="n";
 code="CDTN-1007";
};
 Date(Prior("MT-SL-31-Morro da Janela (Date)","MTLS21_D.prior"))
 {
  site="MT-SL-31-Morro da Janela";
  latitude=-15.92166667;
  longitude=-54.37833333;
  country="Brazil";
  type="rockshelter";
  high altitude="n";
  province="Brazilian Highlands";
  technology="uniface/biface";
  megafauna kill/scavenge="n";
 };
 };
 Boundary("End");
 };
};
```

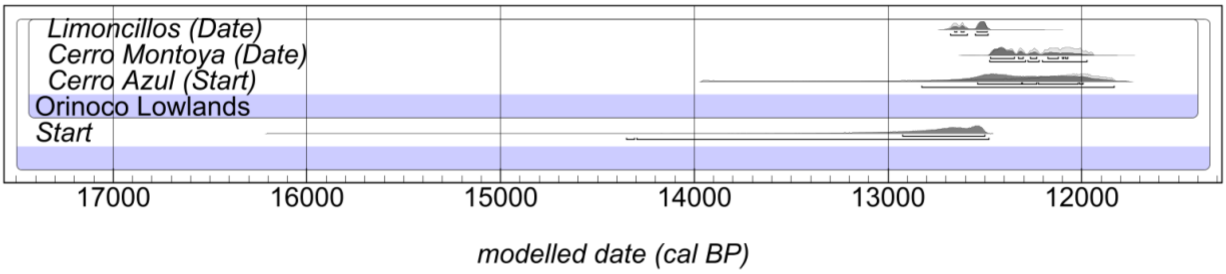


**Supplementary Figure 8.** Bayesian model showing the estimated start of cultural occupation in the Orinico Lowlands (14350-12480 cal BP), including all ACR/YD-aged cultural components within this province (entered as either start boundaries or single ages given available data, and noted next to each component name). Brackets beneath each age estimate show 68.3% and 95.4% CI.

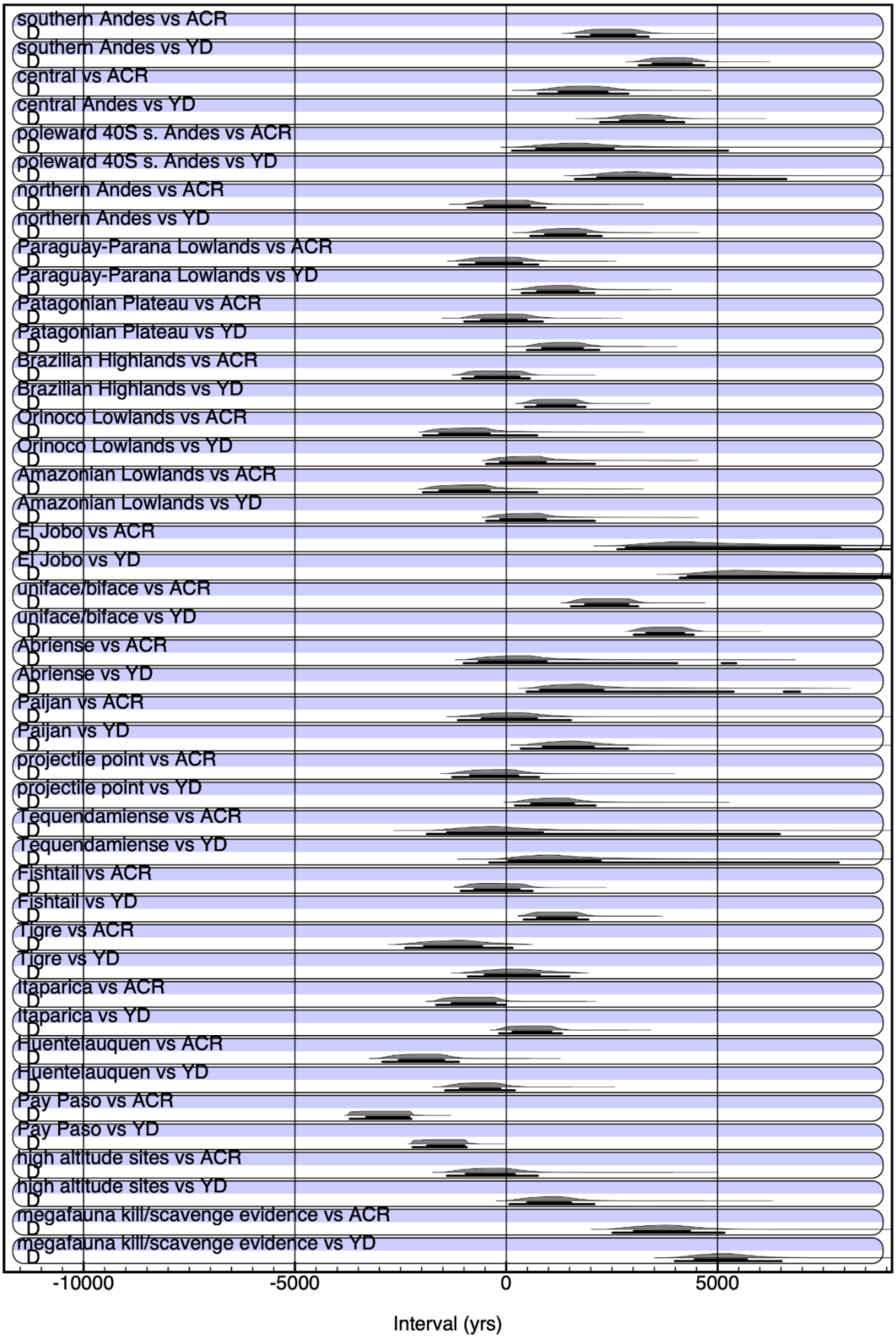


**Supplementary Figure 9.** Probability density function for the difference ('D') between the start of cultural activity for all start estimates noted in Figure 2 vs the temporal span of the ACR and YD. The latter were entered as uniform distributions 14,500-13,000 cal BP [ACR;

ACR=Date(U(calBP(14500),calBP(13000)))] and 13,000-11,700 cal BP [YD; YD=Date(U(calBP(13000),calBP(11700)))]. See Methods for further details. If the distribution does not include 0 (x-axis) at 95.5% CI (larger black bracket underneath each, with 68.2% CI represented by the smaller bracket), there is no significant difference between the modelled outputs and, thus, no overlap. If the distribution is to the right of 0, then it is considered non-overlapping and significantly older. Conversely, if the distribution is to the left of 0, then it is considered non-overlapping and significantly younger.

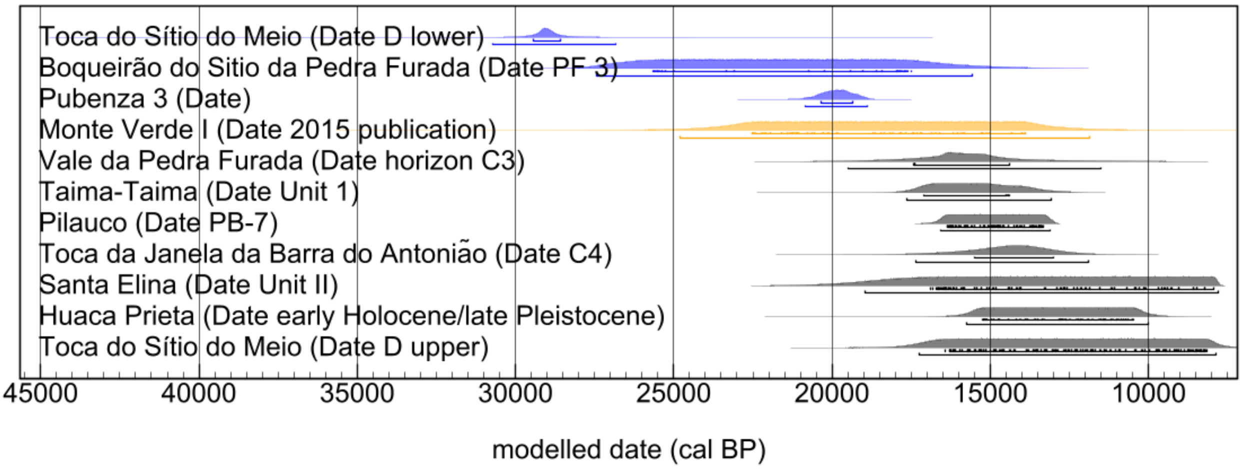


**Supplementary Figure 10.** Bayesian model showing the age range (Date) of cultural components with reported evidence that predates the ACR (not exhaustive, see Boëda et al.[1]). Distributions in blue were not included in the KDE_Model of ACR/YD-aged cultural components shown in Figure 3, as they do not overlap with the ACR. The distribution in orange was also not included as the site of Monte Verde was modelled as MV-II (versus the earlier MV-I), which has an overlapping ACR/YD occupation, to avoid repetition. See details for each site in the report below and note that pre-ACR archaeological evidence is generally contested (e.g., see reference[2]). Brackets beneath each age estimate show 68.3% and 95.4% CI.

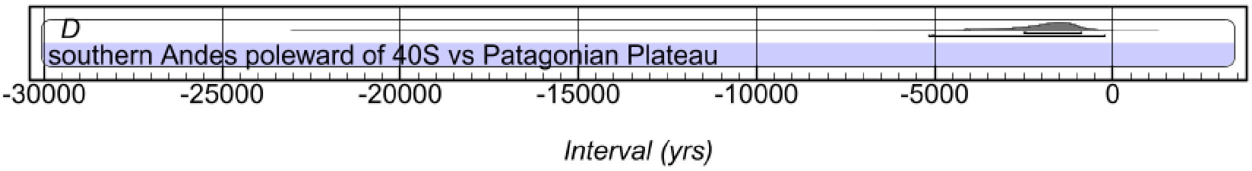


**Supplementary Figure 11.** Probability density function for the difference ('D') between the estimated start of human activity at the southern Andes (poleward of 40° S, only) and the Patagonian Plateau. These results suggest that there is a significant difference between the modelled outputs, as the distributions do not include zero at 95.4% or 68.3% CI (black brackets beneath).

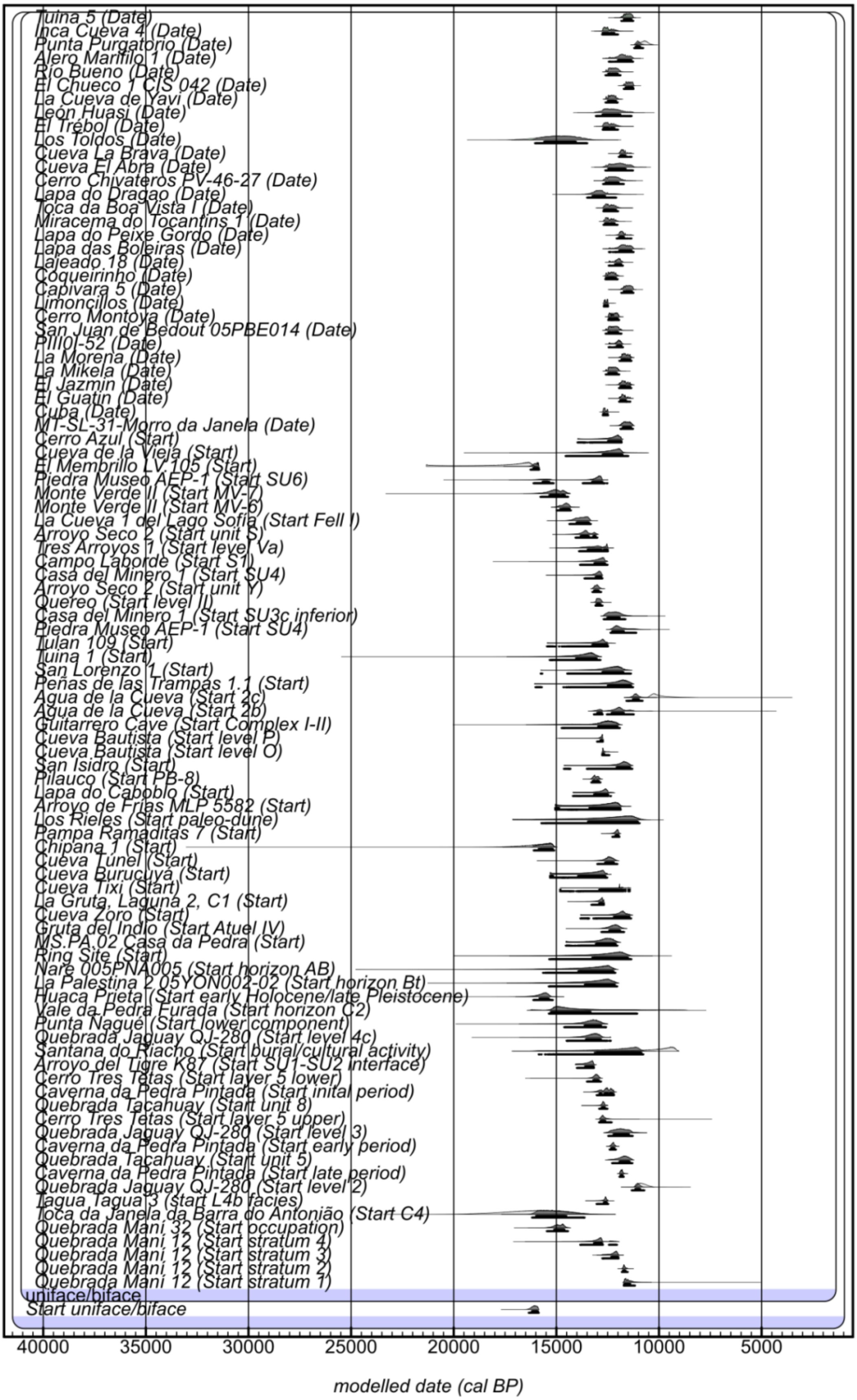


**Supplementary Figure 12.** Bayesian model showing the estimated start of uniface/biface technology (16340-15865 cal BP), including all pertaining ACR/YD-aged cultural components. The latter were entered as either start boundaries or single ages given available data (noted next to each component name). Brackets beneath each age estimate show 68.3% and 95.4% CI.

```
Plot()
{
 Sequence()
 {
  Boundary("Start uniface/biface");
  Phase("uniface/biface")
  {
   Date(Prior("Quebrada Maní 12 (Start stratum 1)","QM12_S_1.prior"))
   {
    site="Quebrada Maní 12";
    latitude=-21.2;
    longitude=-69.4;
    country="Chile";
    type="open-air";
    high altitude="n";
    province="southern Andes";
    technology="uniface/biface";
    megafauna kill/scavenge="n";
   };
   Date(Prior("Quebrada Maní 12 (Start stratum 2)","QM12_S_2.prior"))
   {
    site="Quebrada Maní 12";
    latitude=-21.2;
    longitude=-69.4;
    country="Chile";
    type="open-air";
    high altitude="n";
    province="southern Andes";
    technology="uniface/biface";
    megafauna kill/scavenge="n";
   };
   Date(Prior("Quebrada Maní 12 (Start stratum 3)","QM12_S_3.prior"))
   {
    site="Quebrada Maní 12";
    latitude=-21.2;
    longitude=-69.4;
    country="Chile";
    type="open-air";
    high altitude="n";
    province="southern Andes";
    technology="uniface/biface";
    megafauna kill/scavenge="n";
   };
   Date(Prior("Quebrada Maní 12 (Start stratum 4)","QM12_S_4.prior"))
   {
    site="Quebrada Maní 12";
    latitude=-21.2;
    longitude=-69.4;
    country="Chile";
    type="open-air";
    high altitude="n";
    province="southern Andes";
    technology="uniface/biface";
    megafauna kill/scavenge="n";
   };
   Date(Prior("Quebrada Maní 32 (Start occupation)","QM32_S.prior"))
   {
    site="Quebrada Maní 32";
    latitude=-21.2;
    longitude=-69.4;
    country="Chile";
    type="open-air";
    high altitude="n";
    province="southern Andes";
    technology="uniface/biface";
    megafauna kill/scavenge="n";
   };
   Date(Prior("Toca da Janela da Barra do Antonião (Start C4)","TDJ_S_C4.prior"))
   {
    site="Toca da Janela da Barra do Antonião";
    latitude=-8.802222;
    longitude=-42.433056;
    country="Brazil";
    type="rockshelter";
    high altitude="n";
    province="Brazilian Highlands";
    technology="uniface/biface";
    megafauna kill/scavenge="n";
   };
   Date(Prior("Tagua Tagua 3 (start L4b facies)","TT4_S.prior"))
   {
    site="Tagua Tagua 3";
    latitude=-34.4;
    longitude=-71.2;
    country="Chile";
    type="open-air";
    high altitude="n";
    province="southern Andes";
    technology="uniface/biface";
    megafauna kill/scavenge="y";
   };
   Date(Prior("Quebrada Jaguay QJ-280 (Start level 2)","QJ_S_2.prior"))
   {
    site="Quebrada Jaguay QJ-280";
    latitude=-16.527;
    longitude=-72.856;
    country="Peru";
    type="open-air";
    high altitude="n";
    province="central Andes";
    technology="uniface/biface";
    megafauna kill/scavenge="n";
   };
   Date(Prior("Caverna da Pedra Pintada (Start late period)","CPP_S_late.prior"))
   {
    site="Caverna da Pedra Pintada";
    latitude=-1.1;
    longitude=-54.06;
    country="Brazil";
    type="cave";
    high altitude="n";
    province="Amazonian Lowlands";
    technology="uniface/biface";
    megafauna kill/scavenge="n";
   };
   Date(Prior("Quebrada Tacahuay (Start unit 5)","QT_S_5.prior"))
   {
    site="Quebrada Tacahuay";
    latitude=-17.13;
    longitude=-71.01;
    country="Peru";
    type="open-air";
    high altitude="n";
    province="central Andes";
    technology="uniface/biface";
    megafauna kill/scavenge="n";
   };
   Date(Prior("Caverna da Pedra Pintada (Start early period)","CPP_S_early.prior"))
   {
    site="Caverna da Pedra Pintada";
    latitude=-1.1;
    longitude=-54.06;
    country="Brazil";
    type="cave";
    high altitude="n";
    province="Amazonian Lowlands";
    technology="uniface/biface";
    megafauna kill/scavenge="n";
   };
   Date(Prior("Quebrada Jaguay QJ-280 (Start level 3)","QJ_S_3.prior"))
   {
    site="Quebrada Jaguay QJ-280";
    latitude=-16.527;
    longitude=-72.856;
    country="Peru";
    type="open-air";
    high altitude="n";
    province="central Andes";
    technology="uniface/biface";
    megafauna kill/scavenge="n";
   };
   Date(Prior("Cerro Tres Tetas (Start layer 5 upper)","C3T_S_layer5upper.prior"))
   {
    site="Cerro Tres Tetas";
    latitude=-48.149444;
    longitude=-68.933333;
    country="Argentina";
    type="open-air";
    high altitude="n";
    province="Patagonian Plateau";
    technology="uniface/biface";
    megafauna kill/scavenge="n";
   };
   Date(Prior("Quebrada Tacahuay (Start unit 8)","QT_S_8.prior"))
   {
    site="Quebrada Tacahuay";
    latitude=-17.13;
    longitude=-71.01;
    country="Peru";
    type="open-air";
    high altitude="n";
    province="central Andes";
    technology="uniface/biface";
    megafauna kill/scavenge="n";
   };
   Date(Prior("Caverna da Pedra Pintada (Start inital period)","CPP_S_initial.prior"))
   {
    site="Caverna da Pedra Pintada";
    latitude=-1.1;
    longitude=-54.06;
    country="Brazil";
    type="cave";
    high altitude="n";
    province="Amazonian Lowlands";
    technology="uniface/biface";
    megafauna kill/scavenge="n";
   };
   Date(Prior("Cerro Tres Tetas (Start layer 5 lower)","C3T_S_layer5lower.prior"))
   {
    site="Cerro Tres Tetas";
    latitude=-48.149444;
    longitude=-68.933333;
    country="Argentina";
    type="open-air";
    high altitude="n";
    province="Patagonian Plateau";
    technology="uniface/biface";
    megafauna kill/scavenge="n";
   };
   Date(Prior("Arroyo del Tigre K87 (Start SU1-SU2 interface)","K87_S_1_2.prior"))
   {
    site="Arroyo del Tigre K87";
    latitude=-30.4;
    longitude=-57.8;
    country="Uruguay";
    type="open-air";
    high altitude="n";
    province="Paraguay-Parana Lowlands";
    technology="uniface/biface";
    megafauna kill/scavenge="n";
   };
   Date(Prior("Santana do Riacho (Start burial/cultural activity)","SR_S_humans.prior"))
   {
    site="Santana do Riacho";
    latitude=-19.2;
    longitude=-43.7;
    country="Brazil";
    type="rockshelter";
    high altitude="n";
    province="Brazilian Highlands";
    technology="uniface/biface";
    megafauna kill/scavenge="n";
   };
   Date(Prior("Quebrada Jaguay QJ-280 (Start level 4c)","QJ_S_4.prior"))
   {
    site="Quebrada Jaguay QJ-280";
    latitude=-16.527;
    longitude=-72.856;
    country="Peru";
    type="open-air";
    high altitude="n";
    province="central Andes";
    technology="uniface/biface";
    megafauna kill/scavenge="n";
   };
   Date(Prior("Punta Ñagué (Start lower component)","PN_S_lower.prior"))
   {
    site="Punta Ñagué";
    latitude=-31.85;
    longitude=-71.52;
    country="Chile";
    type="open-air";
    high altitude="n";
    province="southern Andes";
    technology="uniface/biface";
    megafauna kill/scavenge="n";
   };
   Date(Prior("Vale da Pedra Furada (Start horizon C2)","VPF_S_C2.prior"))
   {
    site="Vale da Pedra Furada";
    latitude=-8.85;
```

```
 longitude=-42.55;
 country="Brazil";
 type="open-air";
 high altitude="n";
 province="Brazilian Highlands";
 technology="uniface/biface";
 megafauna kill/scavenge="n";
};
Date(Prior("Huaca Prieta (Start early Holocene/late Pleistocene)","HC_S_Pleis.prior"))
{
 site="Huaca Prieta";
 latitude=-7.9241;
 longitude=-79.3069;
 country="Peru";
 type="open-air";
 high altitude="n";
 province="central Andes";
 technology="uniface/biface";
 megafauna kill/scavenge="n";
};
Date(Prior("La Palestina 2 05YON002-02 (Start horizon Bt)","LP2_S_Bt.prior"))
{
 site="La Palestina 2 05YON002-02";
 latitude=6.716;
 longitude=-74.383;
 country="Colombia";
 type="open-air";
 high altitude="n";
 province="northern Andes";
 technology="uniface/biface";
 megafauna kill/scavenge="n";
};
Date(Prior("Nare 005PNA005 (Start horizon AB)","Nare_S.prior"))
{
 site="Nare 005PNA005";
 latitude=6.35;
 longitude=-74.1;
 country="Colombia";
 type="open-air";
 high altitude="n";
 province="northern Andes";
 technology="uniface/biface";
 megafauna kill/scavenge="n";
};
Date(Prior("Ring Site (Start)","TRS_S.prior"))
{
 site="Ring Site";
 latitude=-17.667;
 longitude=-71.333;
 country="Peru";
 type="open-air";
 high altitude="n";
 province="central Andes";
 technology="uniface/biface";
 megafauna kill/scavenge="n";
};
Date(Prior("MS.PA.02 Casa da Pedra (Start)","MSPA_S.prior"))
{
 site="MS.PA.02 Casa da Pedra";
 latitude=-19.58333333;
 longitude=-52.66666667;
 country="Brazil";
 type="cave";
 high altitude="n";
 province="Brazilian Highlands";
 technology="uniface/biface";
 megafauna kill/scavenge="n";
};
Date(Prior("Gruta del Indio (Start Atuel IV)","GdI_IV_S.prior"))
{
 site="Gruta del Indio";
 latitude=-34.75;
 longitude=-68.3666;
 country="Argentina";
 type="cave";
 high altitude="n";
 province="southern Andes";
 technology="uniface/biface";
 megafauna kill/scavenge="n";
};
Date(Prior("Cueva Zoro (Start)","CZ_S.prior"))
{
 site="Cueva Zoro";
 latitude=-37.8166;
 longitude=-58.6333;
 country="Argentina";
 type="rockshelter";
 high altitude="n";
 province="Paraguay-Parana Lowlands";
 technology="uniface/biface";
 megafauna kill/scavenge="n";
};
Date(Prior("La Gruta, Laguna 2, C1 (Start)","LG_S.prior"))
{
 site="La Gruta, Laguna 2, C1";
 latitude=-48.8261;
 longitude=-69.3961;
 country="Argentina";
 type="cave";
 high altitude="n";
 province="Patagonian Plateau";
 technology="uniface/biface";
 megafauna kill/scavenge="n";
};
Date(Prior("Cueva Tixi (Start)","CTixi_S.prior"))
{
 site="Cueva Tixi";
 latitude=-37.4;
 longitude=-58.9;
 country="Argentina";
 type="cave";
 high altitude="n";
 province="Paraguay-Parana Lowlands";
 technology="uniface/biface";
 megafauna kill/scavenge="n";
};
Date(Prior("Cueva Burucuyá (Start)","CBuru_S.prior"))
{
 site="Cueva Burucuyá";
 latitude=-37.8;
 longitude=-58.1;
 country="Argentina";
 type="cave";
 high altitude="n";
 province="Paraguay-Parana Lowlands";
 technology="uniface/biface";
 megafauna kill/scavenge="n";
};
Date(Prior("Cueva Túnel (Start)","CTunel_S.prior"))
{
 site="Cueva Túnel";
 latitude=-48.4575;
 longitude=-68.8725;
 country="Argentina";
 type="cave";
 high altitude="n";
 province="Patagonian Plateau";
 technology="uniface/biface";
 megafauna kill/scavenge="n";
};
Date(Prior("Chipana 1 (Start)","Chipana1_S.prior"))
{
 site="Chipana 1";
 latitude=-21.2;
 longitude=-69.4;
 country="Chile";
 type="open-air";
 high altitude="n";
 province="southern Andes";
 technology="uniface/biface";
 megafauna kill/scavenge="n";
};
Date(Prior("Pampa Ramaditas 7 (Start)","PR7_S.prior"))
{
 site="Pampa Ramaditas 7";
 latitude=-21.2;
 longitude=-69.4;
 country="Chile";
 type="open-air";
 high altitude="n";
 province="southern Andes";
 technology="uniface/biface";
 megafauna kill/scavenge="n";
};
Date(Prior("Los Rieles (Start paleo-dune)","LR_S_dune.prior"))
{
 site="Los Rieles";
 latitude=-31.93;
 longitude=-71.52;
 country="Chile";
 type="open-air";
 high altitude="n";
 province="southern Andes";
 technology="uniface/biface";
 megafauna kill/scavenge="n";
};
Date(Prior("Arroyo de Frías MLP 5582 (Start)","AdF_S.prior"))
{
 site="Arroyo de Frías MLP 5582";
 latitude=-34.65;
 longitude=-59.4307;
 country="Argentina";
 type="open-air";
 high altitude="n";
 province="Paraguay-Parana Lowlands";
 technology="uniface/biface";
 megafauna kill/scavenge="n";
};
Date(Prior("Lapa do Caboblo (Start)","LdCabo_S.prior"))
{
 site="Lapa do Caboblo";
 latitude=-18.28527778;
 longitude=-43.85388889;
 country="Brazil";
 type="rockshelter/cave";
 high altitude="n";
 province="Brazilian Highlands";
 technology="uniface/biface";
 megafauna kill/scavenge="n";
};
Date(Prior("Pilauco (Start PB-8)","Pil_PB8_S.prior"))
{
 site="Pilauco";
 latitude=-40.566667;
 longitude=-73.116667;
 country="Chile";
 type="open-air";
 high altitude="n";
 province="northern Andes";
 technology="uniface/biface";
 megafauna kill/scavenge="n";
};
Date(Prior("San Isidro (Start)","SIsi_S.prior"))
{
 site="San Isidro";
 latitude=2.4;
 longitude=-76.5;
 country="Colombia";
 type="open-air";
 high altitude="n";
 province="northern Andes";
 technology="uniface/biface";
 megafauna kill/scavenge="n";
};
Date(Prior("Cueva Bautista (Start level O)","CB_S_O.prior"))
{
 site="Cueva Bautista";
 latitude=-21.338;
 longitude=-67.574;
 country="Bolivia";
 type="rockshelter";
 high altitude="y";
 province="central Andes";
 technology="uniface/biface";
 megafauna kill/scavenge="n";
};
Date(Prior("Cueva Bautista (Start level P)","CB_S_P.prior"))
{
 site="Cueva Bautista";
 latitude=-21.338;
 longitude=-67.574;
 country="Bolivia";
 type="rockshelter";
 high altitude="y";
 province="central Andes";
 technology="uniface/biface";
 megafauna kill/scavenge="n";
};
Date(Prior("Guitarrero Cave (Start Complex I-II)","GC_S_I_II.prior"))
{
 site="Guitarrero Cave";
 latitude=-9.20069;
 longitude=-77.7098;
```

```
 country="Peru";
 type="cave";
 high altitude="y";
 province="central Andes";
 technology="uniface/biface";
 megafauna kill/scavenge="n";
};
Date(Prior("Agua de la Cueva (Start 2b)","AdC_2b_S.prior"))
{
 site="Agua de la Cueva";
 latitude=-32.6169;
 longitude=-69.1636;
 country="Argentina";
 type="rockshelter";
 high altitude="y";
 province="southern Andes";
 technology="uniface/biface";
 megafauna kill/scavenge="n";
};
Date(Prior("Agua de la Cueva (Start 2c)","AdC_2c_S.prior"))
{
 site="Agua de la Cueva";
 latitude=-32.6169;
 longitude=-69.1636;
 country="Argentina";
 type="rockshelter";
 high altitude="y";
 province="southern Andes";
 technology="uniface/biface";
 megafauna kill/scavenge="n";
};
Date(Prior("Peñas de las Trampas 1.1 (Start)","PdlT1_S.prior"))
{
 site="Peñas de las Trampas 1.1";
 latitude=-26.0175;
 longitude=-67.35;
 country="Argentina";
 type="rockshelter";
 high altitude="y";
 province="southern Andes";
 technology="uniface/biface";
 megafauna kill/scavenge="n";
};
Date(Prior("San Lorenzo 1 (Start)","SLoren1_S.prior"))
{
 site="San Lorenzo 1";
 latitude=-23.4;
 longitude=-67.9;
 country="Chile";
 type="cave";
 high altitude="y";
 province="southern Andes";
 technology="uniface/biface";
 megafauna kill/scavenge="n";
};
Date(Prior("Tuina 1 (Start)","Tuina1_S.prior"))
{
 site="Tuina 1";
 latitude=-22.8;
 longitude=-68.3;
 country="Chile";
 type="rockshelter";
 high altitude="y";
 province="southern Andes";
 technology="uniface/biface";
 megafauna kill/scavenge="n";
};
Date(Prior("Tulan 109 (Start)","Tulan9_S.prior"))
{
 site="Tulan 109";
 latitude=-23.6;
 longitude=-67.9;
 country="Chile";
 type="cave";
 high altitude="y";
 province="southern Andes";
 technology="uniface/biface";
 megafauna kill/scavenge="n";
};
Date(Prior("Piedra Museo AEP-1 (Start SU4)","PM_S_SU4.prior"))
{
 site="Piedra Museo AEP-1";
 latitude=-47.895;
 longitude=-67.867778;
 country="Argentina";
 type="rockshelter";
 high altitude="n";
 province="Patagonian Plateau";
 technology="uniface/biface";
 megafauna kill/scavenge="y";
};
Date(Prior("Casa del Minero 1 (Start SU3c inferior)","CM1_S_SU3c.prior"))
{
 site="Casa del Minero 1";
 latitude=-48.575833;
 longitude=-68.9125;
 country="Argentina";
 type="cave";
 high altitude="n";
 province="Patagonian Plateau";
 technology="uniface/biface";
 megafauna kill/scavenge="y";
};
Date(Prior("Quereo (Start level II)","Q_S_QuereoII.prior"))
{
 site="Quereo";
 latitude=-31.93;
 longitude=-71.51;
 country="Chile";
 type="open-air";
 high altitude="n";
 province="southern Andes";
 technology="uniface/biface";
 megafauna kill/scavenge="y";
};
Date(Prior("Arroyo Seco 2 (Start unit Y)","AS2_S_unitY.prior"))
{
 site="Arroyo Seco 2";
 latitude=-38.360556;
 longitude=-60.244167;
 country="Argentina";
 type="open-air";
 high altitude="n";
 province="Paraguay-Parana Lowlands";
 technology="uniface/biface";
 megafauna kill/scavenge="y";
};
Date(Prior("Casa del Minero 1 (Start SU4)","CM1_S_SU4.prior"))
{
 site="Casa del Minero 1";
 latitude=-48.575833;
 longitude=-68.9125;
 country="Argentina";
 type="cave";
 high altitude="n";
 province="Patagonian Plateau";
 technology="uniface/biface";
 megafauna kill/scavenge="y";
};
Date(Prior("Campo Laborde (Start S1)","CL_S_1.prior"))
{
 site="Campo Laborde";
 latitude=-37.1;
 longitude=-60.38;
 country="Argentina";
 type="open-air";
 high altitude="n";
 province="Paraguay-Parana Lowlands";
 technology="uniface/biface";
 megafauna kill/scavenge="y";
};
Date(Prior("Tres Arroyos 1 (Start level Va)","TA1_S_Va.prior"))
{
 site="Tres Arroyos 1";
 latitude=-53.383;
 longitude=-68.783;
 country="Chile";
 type="rockshelter";
 high altitude="n";
 province="Patagonian Plateau";
 technology="uniface/biface";
 megafauna kill/scavenge="y";
};
Date(Prior("Arroyo Seco 2 (Start unit S)","AS2_S_unitS.prior"))
{
 site="Arroyo Seco 2";
 latitude=-38.360556;
 longitude=-60.244167;
 country="Argentina";
 type="open-air";
 high altitude="n";
 province="Paraguay-Parana Lowlands";
 technology="uniface/biface";
 megafauna kill/scavenge="y";
};
Date(Prior("La Cueva 1 del Lago Sofía (Start Fell I)","CLS_S_Fell_I.prior"))
{
 site="La Cueva 1 del Lago Sofía";
 latitude=-51.53;
 longitude=-72.53;
 country="Chile";
 type="cave";
 high altitude="n";
 province="Patagonian Plateau";
 technology="uniface/biface";
 megafauna kill/scavenge="y";
};
Date(Prior("Monte Verde II (Start MV-6)","MVII_S_MV6.prior"))
{
 site="Monte Verde II";
 latitude=-41.51;
 longitude=-73.2;
 country="Chile";
 type="open-air";
 high altitude="n";
 province="southern Andes";
 technology="uniface/biface";
 megafauna kill/scavenge="y";
};
Date(Prior("Monte Verde II (Start MV-7)","MVII_S_MV7.prior"))
{
 site="Monte Verde II";
 latitude=-41.51;
 longitude=-73.2;
 country="Chile";
 type="open-air";
 high altitude="n";
 province="southern Andes";
 technology="uniface/biface";
 megafauna kill/scavenge="y";
};
Date(Prior("Piedra Museo AEP-1 (Start SU6)","PM_S_SU6.prior"))
{
 site="Piedra Museo AEP-1";
 latitude=-47.895;
 longitude=-67.867778;
 country="Argentina";
 type="rockshelter";
 high altitude="n";
 province="Patagonian Plateau";
 technology="uniface/biface";
 megafauna kill/scavenge="y";
};
Date(Prior("El Membrillo LV.105 (Start)","Membrillo_S.prior"))
{
 site="El Membrillo LV.105";
 latitude=-31.928333;
 longitude=-71.488333;
 country="Chile";
 type="open-air";
 high altitude="n";
 province="southern Andes";
 technology="uniface/biface";
 megafauna kill/scavenge="y";
};
Date(Prior("Cueva de la Vieja (Start)","CdlV_S.prior"))
{
 site="Cueva de la Vieja";
 latitude=-42.274167;
 longitude=-71.54;
 country="Chile";
 type="cave";
 high altitude="n";
 province="Patagonian Plateau";
 technology="uniface/biface";
 megafauna kill/scavenge="y";
};
Date(Prior("Cerro Azul (Start)","CA_S.prior"))
{
 site="Cerro Azul";
 latitude=2.5298;
 longitude=-72.8664;
 country="Colombia";
```

```
 type="rockshelter";
 high altitude="n";
 province="Orinoco Lowlands";
 technology="uniface/biface";
 megafauna kill/scavenge="n";
};
Date(Prior("MT-SL-31-Morro da Janela (Date)","MTLS21_D.prior"))
{
 site="MT-SL-31-Morro da Janela";
 latitude=-15.92166667;
 longitude=-54.37833333;
 country="Brazil";
 type="rockshelter";
 high altitude="n";
 province="Brazilian Highlands";
 technology="uniface/biface";
 megafauna kill/scavenge="n";
};
R_Date("Cuba (Date)",10619,66)
{
 site="Cuba ";
 material="charcoal";
 latitude="5.197776";
 longitude="-75.676397";
 country="Colombia"type="open-air";
 high altitude="n";
 technology="uniface/biface";
 megafauna kill/scavenge="n";
 code="AA-102510";
};
R_Date("El Guatin (Date)",10130,50)
{
 site="El Guatin";
 material="charcoal";
 latitude="4.75";
 longitude="-75.7";
 country="Colombia"type="open-air";
 high altitude="n";
 technology="uniface/biface";
 megafauna kill/scavenge="n";
 code="Beta-325213";
};
R_Date("El Jazmin (Date)",10120,70)
{
 site="El Jazmin";
 material="charcoal";
 latitude="3.8";
 longitude="-76.2";
 country="Colombia"type="open-air";
 high altitude="n";
 technology="uniface/biface";
 megafauna kill/scavenge="n";
 code="unreported";
};
R_Date("La Mikela (Date)",10376,70)
{
 site="La Mikela";
 material="charcoal";
 latitude="4.781956";
 longitude="-75.68447";
 country="Colombia"type="open-air";
 high altitude="n";
 technology="uniface/biface";
 megafauna kill/scavenge="n";
 code="AA-98939";
};
R_Date("La Morena (Date)",10090,60)
{
 site="La Morena";
 material="unreported";
 latitude="6.2";
 longitude="-75.5";
 country="Colombia"type="open-air";
 high altitude="n";
 technology="uniface/biface";
 megafauna kill/scavenge="n";
 code="unreported";
};
R_Date("PIII0I-52 (Date)",10260,50)
{
 site="PIII0I-52";
 material="unreported";
 latitude="6.8";
 longitude="-75.1";
 country="Colombia"type="open-air";
 high altitude="n";
 technology="uniface/biface";
 megafauna kill/scavenge="n";
 code="unreported";
};
R_Date("San Juan de Bedout 05PBE014 (Date)",10350,90)
{
 site="San Juan de Bedout 05PBE014";
 material="";
 latitude="6.5";
 longitude="-74.5";
 country="Colombia"type="open-air";
 high altitude="n";
 technology="uniface/biface";
 megafauna kill/scavenge="n";
 code="no code San Juan";
};
R_Date("Cerro Montoya (Date)",10340,40)
{
 site="Cerro Montoya";
 material="seed";
 latitude="2.5644";
 longitude="-72.874";
 country="Colombia"type="rockshelter";
 high altitude="n";
 province=Orinoco Lowlands;
 technology="uniface/biface";
 megafauna kill/scavenge="n";
 code="Beta-509123";
};
R_Date("Limoncillos (Date)",10560,30)
{
 site="Limoncillos";
 material="seed";
 latitude="2.5644";
 longitude="-72.874";
 country="Colombia"type="rockshelter";
 high altitude="n";
 province=Orinoco Lowlands;
 technology="uniface/biface";
 megafauna kill/scavenge="n";
 code="Beta-509123 dup";
};
Curve("SHCal20","shcal20.14c");
R_Date("Capivara 5 (Date)",10050,80)
{
 site="Capivara 5";
 material="";
 latitude="-10.14";
 longitude="-48.44";
 country="Brazil"type="open-air";
 high altitude="n";
 technology="uniface/biface";
 megafauna kill/scavenge="n";
 code="Beta-179196";
};
R_Date("Coqueirinho (Date)",10460,60)
{
 site="Coqueirinho";
 material="";
 latitude="-19.54";
 longitude="-43.95";
 country="Brazil"type="open-air";
 high altitude="n";
 technology="uniface/biface";
 megafauna kill/scavenge="n";
 code="Beta-237346";
};
R_Date("Lajeado 18 (Date)",10300,60)
{
 site="Lajeado 18";
 material="";
 latitude="-19.52";
 longitude="-44.07";
 country="Brazil"type="rockshelter/cave";
 high altitude="n";
 technology="uniface/biface";
 megafauna kill/scavenge="n";
 code="Beta-179198";
};
R_Date("Lapa das Boleiras (Date)",10150,130)
{
 site="Lapa das Boleiras";
 material="";
 latitude="-18.3";
 longitude="-43.74";
 country="Brazil"type="rockshelter/cave";
 high altitude="n";
 technology="uniface/biface";
 megafauna kill/scavenge="n";
 code="Beta-168451";
};
R_Date("Lapa do Peixe Gordo (Date)",10210,60)
{
 site="Lapa do Peixe Gordo";
 material="";
 latitude="-28.6";
 longitude="-65.6";
 country="Argentina"type="rockshelter";
 high altitude="n";
 technology="uniface/biface";
 megafauna kill/scavenge="n";
 code="Beta-233762";
};
R_Date("Miracema do Tocantins 1 (Date)",10530,90)
{
 site="Miracema do Tocantins 1";
 material="";
 latitude="-9.64";
 longitude="-48.41";
 country="Brazil"type="open-air";
 high altitude="n";
 technology="uniface/biface";
 megafauna kill/scavenge="n";
 code="Beta-190080";
};
R_Date("Toca da Boa Vista I (Date)",10530,110)
{
 site="Toca da Boa Vista I";
 material="";
 latitude="-8.63";
 longitude="-42.36";
 country="Brazil"type="rockshelter/cave";
 high altitude="n";
 technology="uniface/biface";
 megafauna kill/scavenge="n";
 code="Beta-32971";
};
R_Date("Lapa do Dragao (Date)",11000,300)
{
 site="Lapa do Dragao";
 material="";
 latitude="-14.4167";
 longitude="-44.4";
 country="Brazil"type="rockshelter";
 high altitude="n";
 province=Brazilian Highlands;
 technology="uniface/biface";
 megafauna kill/scavenge="n";
 code="CDTN-1007";
};
R_Date("Cerro Chivateros PV-46-27 (Date)",10430,160)
{
 site="Cerro Chivateros PV-46-27";
 material="";
 latitude="-11.94";
 longitude="-77.13";
 country="Peru"type="open-air";
 high altitude="n";
 technology="uniface/biface";
 megafauna kill/scavenge="n";
 code="UCLA-683";
};
R_Date("Cueva El Abra (Date)",10270,200)
{
 site="Cueva El Abra";
 material="";
 latitude="-37.4";
 longitude="-58.90";
 country="Argentina"type="cave";
 high altitude="n";
 technology="uniface/biface";
 megafauna kill/scavenge="n";
 code="AA-94641";
};
R_Date("Cueva La Brava (Date)",10178,54)
{
 site="Cueva La Brava";
```

```
  material="";
  latitude="-37.8";
  longitude="-58.1";
  country="Argentina"type="cave";
  high altitude="n";
  technology="uniface/biface";
  megafauna kill/scavenge="n";
  code="AA-9463";
 };
 R_Date("Los Toldos (Date)",12600,600)
 {
  site="Los Toldos";
  material="";
  latitude="-47.9";
  longitude="-67.87";
  country="Argentina"type="cave";
  high altitude="n";
  technology="uniface/biface";
  megafauna kill/scavenge="n";
  code="no code Los Toldos";
 };
 R_Date("El Trébol (Date)",10570,130)
 {
  site="El Trébol";
  latitude=-41.0733;
  longitude=-71.4866;
  country="Argentina";
  type="cave";
  high altitude="n";
  province="southern Andes";
  technology="uniface/biface";
  megafauna kill/scavenge="n";
  code="AA-65707";
 };
 R_Date("León Huasi (Date)",10550,300)
 {
  site="León Huasi";
  material="";
  latitude="-26.3";
  longitude="-70.6";
  country="Chile"type="open-air";
  high altitude="n";
  technology="Huentelauquén";
  megafauna kill/scavenge="n";
  code="GAK-13402";
 };
 R_Date("La Cueva de Yavi (Date)",10450,55)
 {
  site="La Cueva de Yavi";
  material="unreported";
  latitude=-22.1;
  longitude=-65.5;
  country="Argentina";
  type="cave";
  high altitude="n";
  technology="uniface/biface";
  megafauna kill/scavenge="n";
  code="CSIC-1101";
 };
 R_Date("El Chueco 1 CIS 042 (Date)",10010,60)
 {
  site="El Chueco 1 CIS 042";
  material="";
  latitude="-44.4";
  longitude="-71.30";
  country="Chile"type="cave";
  high altitude="n";
  technology="uniface/biface";
  megafauna kill/scavenge="n";
  code="Beta-227703";
 };
 R_Date("Río Bueno (Date)",10400,90)
 {
  site="Río Bueno";
  material="";
  latitude="-24.3";
  longitude="-68.9";
  country="Chile"type="open-air";
  high altitude="y";
  technology="projectile point";
  megafauna kill/scavenge="n";
  code="No code Rio Bueno";
 };
 R_Date("Alero Marifilo 1 (Date)",10190,120)
 {
  site="Alero Marifilo 1";
  material="";
  latitude="-39.51";
  longitude="-72.06";
  country="Chile"type="rockshelter";
  high altitude="n";
  technology="uniface/biface";
  megafauna kill/scavenge="n";
  code="Beta-164475";
 };
 Date(Prior("Punta Purgatorio (Date)","PPurga_D.prior"))
 {
  site="Punta Purgatorio";
  latitude=-31.963333;
  longitude=-71.499444;
  country="Chile";
  type="open-air";
  high altitude="n";
  province="southern Andes";
  technology="uniface/biface";
  megafauna kill/scavenge="n";
 };
 R_Date("Inca Cueva 4 (Date)",10620,140)
 {
  site="Inca Cueva 4";
  material="";
  latitude="-7.06";
  longitude="-79.40";
  country="Peru"type="open-air";
  high altitude="n";
  technology="Paiján";
  megafauna kill/scavenge="n";
  code="LP-137";
 };
 R_Date("Tuina 5 (Date)",10060,70)
 {
  site="Tuina 5";
  material="";
  latitude="-22.8";
  longitude="-68.3";
  country="Chile"type="cave";
  high altitude="y";
  technology="uniface/biface";
  megafauna kill/scavenge="n";
  code="Beta-107120";
 };
};
Boundary("End uniface/biface");
};
};
```

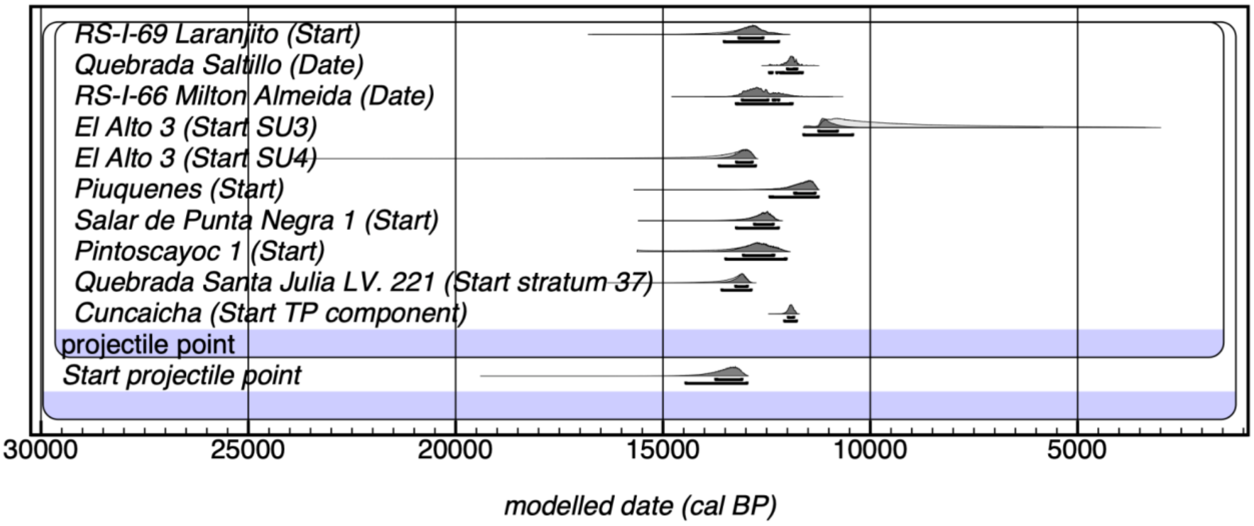


**Supplementary Figure 13.** Bayesian model showing the estimated start of projectile point technology (14220-13045 cal BP), including all pertaining ACR/YD-aged cultural components. The latter were entered as either start boundaries or single ages given available data (noted next to each component name). Brackets beneath each age estimate show 68.3% and 95.4% CI. Note, although there are projectile points in Pampa del Tamarugal (Chile) sites, e.g., Pampa Ramadita 5 and Quebrada Maní 35[3], cultural components from that region have been excluded as either surface finds or samples without clear/close association with dated samples (see details in Supplementary Notes 2).

```
Plot()
{
 Curve("SHCal20","shcal20.14c");
 Sequence()
 {
  Boundary("Start projectile point");
  Phase("projectile point")
  {
   Date(Prior("Cuncaicha (Start TP component)","Cun_S_TP.prior"))
   {
    site="Cuncaicha";
    latitude=-15.373;
    longitude=-72.618;
    country="Peru";
    type="rockshelter";
    high altitude="y";
    province="Andes";
    technology="projectile point";
    megafauna kill/scavenge="n";
   };
   Date(Prior("Quebrada Santa Julia LV. 221 (Start stratum 37)","QSJ_S_37.prior"))
   {
    site="Quebrada Santa Julia LV. 221";
    latitude=-31.836917;
    longitude=-71.488278;
    country="Chile";
    type="open-air";
    high altitude="n";
    province="Andes";
    technology="projectile point";
    megafauna kill/scavenge="y";
   };
   Date(Prior("Pintoscayoc 1 (Start)","Pinto1_S.prior"))
   {
    site="Pintoscayoc 1";
    latitude=-22.9444;
    longitude=-65.4182;
    country="Argentina";
    type="rockshelter";
    high altitude="y";
    province="Andes";
    technology="projectile point";
    megafauna kill/scavenge="n";
   };
   Date(Prior("Salar de Punta Negra 1 (Start)","SPN1_S.prior"))
   {
    site="Salar de Punta Negra 1";
    latitude=-24.466667;
    longitude=-68.883333;
    country="Chile";
    type="open-air";
    high altitude="y";
    province="Andes";
    technology="projectile point";
    megafauna kill/scavenge="n";
   };
   Date(Prior("Piuquenes (Start)","Piuquenes_S.prior"))
   {
    site="Piuquenes";
    latitude=-32.266667;
    longitude=-70.266667;
    country="Chile";
    type="rockshelter";
    high altitude="n";
    province="Andes";
    technology="projectile point";
    megafauna kill/scavenge="n";
   };
   Date(Prior("El Alto 3 (Start SU4)","EA3_SU4_S.prior"))
   {
    site="El Alto 3";
    latitude=-31.3988;
    longitude=-64.7388;
    country="Argentina";
    type="rockshelter";
    high altitude="n";
    province="Andes";
    technology="projectile point";
    megafauna kill/scavenge="n";
   };
   Date(Prior("El Alto 3 (Start SU3)","EA3_SU3_S.prior"))
   {
    site="El Alto 3";
    latitude=-31.3988;
    longitude=-64.7388;
    country="Argentina";
    type="rockshelter";
    high altitude="n";
    province="Andes";
    technology="projectile point";
    megafauna kill/scavenge="n";
   };
   R_Date("RS-I-66 Milton Almeida (Date)",10810,275)
   {
    site="RS-I-66 Milton Almeida";
    material="";
    latitude="-29.72";
    longitude="-56.7";
    country="Brazil";
    type="open-air";
    high altitude="n";
    technology="uniface/biface";
    megafauna kill/scavenge="n";
    code="SI-2722";
   };
   R_Date("Quebrada Saltillo (Date)",10260,60)
   {
    site="Quebrada Saltillo";
    material="";
    latitude="-6.87";
    longitude="-79.42";
    country="Peru";
    type="open-air";
    high altitude="n";
    technology="Paiján";
    megafauna kill/scavenge="n";
    code="Beta-191578";
   };
   Date(Prior("RS-I-69 Laranjito (Start)","RSI_S.prior"))
   {
    site="RS-I-69 Laranjito";
    latitude=-29.61333333;
    longitude=-56.93;
    country="Brazil";
    type="open-air";
    high altitude="n";
    province="Brazilian Highlands";
    technology="projectile point";
    megafauna kill/scavenge="n";
   };
  };
  Boundary("End projectile point");
 };
};
```

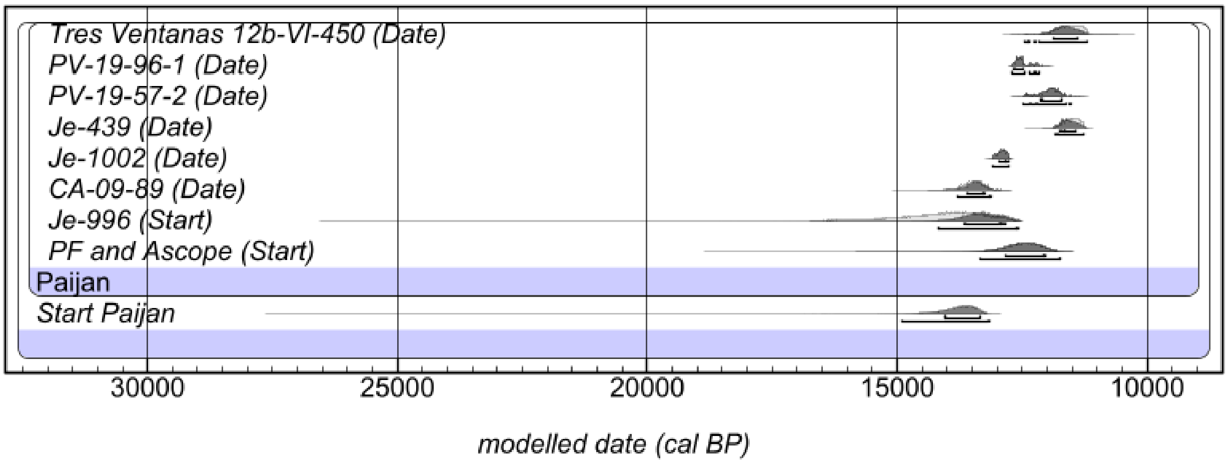


**Supplementary Figure 14.** Bayesian model showing the estimated start of Paiján technology (14960-13175 cal BP), including all pertaining ACR/YD-aged cultural components. The latter were entered as either start boundaries or single ages given available data (noted next to each component name). Brackets beneath each age estimate show 68.3% and 95.4% CI.

```
Plot()
{
 Curve("SHCal20","shcal20.14c");
 Sequence()
 {
  Boundary("Start Paijan");
  Phase("Paijan")
  {
   Date(Prior("PF and Ascope (Start)","Paijan_S.prior"))
   {
    site="Pampa de los Fósiles and Ascope";
    latitude=-7.71;
    longitude=-79.1;
    country="Peru";
    type="open-air";
    high altitude="n";
    technology="Paiján";
    megafauna kill/scavenge="n";
   };
   R_Date("CA-09-57/CA-09-55-2 (Date)",10360,100)
   {
    province="Andes";
    site="CA-09-57/CA-09-55-2";
    material="";
    latitude="-6.95";
    longitude="-79.24";
    country="Peru";type="open-air";
    high altitude="n";
    technology="Paiján";
    megafauna kill/scavenge="n";
    code="Beta-154141";
   };
   Date(Prior("Je-996 (Start)","Je_S.prior"))
   {
    site="Je-996";
    latitude=-7.039;
    longitude=-79.397;
    country="Peru";
    type="open-air";
    high altitude="n";
    province="Andes";
    technology="Paiján and Fishtail";
    megafauna kill/scavenge="n";
   };
   R_Date("CA-09-89 (Date)",11650,180)
   {
    site="CA-09-89";
    material="";
    latitude="-6.95";
    longitude="-79.24";
    country="Peru";type="open-air";
    high altitude="n";
    technology="Paiján";
    megafauna kill/scavenge="n";
    code="Beta-20887";
   };
   R_Date("Je-1002 (Date)",11014,64)
   {
    site="Je-1002";
    material="";
    latitude="-7.07";
    longitude="-79.41";
    country="Peru";type="open-air";
    high altitude="n";
    technology="Paiján";
    megafauna kill/scavenge="n";
    code="AA-57942";
   };
   R_Date("Je-439 (Date)",10056,67)
   {
    site="Je-439";
    material="";
    latitude="-22.12";
    longitude="-65.47";
    country="Argentina";type="cave";
    high altitude="n";
    technology="uniface/biface";
    megafauna kill/scavenge="n";
    code="AA-57950";
   };
   R_Date("PV-19-57-2 (Date)",10260,90)
   {
    site="PV-19-57-2";
    material="";
    latitude="-31.96";
    longitude="-71.5";
    country="Chile";type="open-air";
    high altitude="n";
    technology="uniface/biface";
    megafauna kill/scavenge="n";
    code="Beta-154128";
   };
   R_Date("PV-19-96-1 (Date)",10560,60)
   {
    site="PV-19-96-1";
    material="";
    latitude="-7.17";
    longitude="-79.52";
    country="Peru";type="open-air";
    high altitude="n";
    technology="Paiján";
    megafauna kill/scavenge="n";
    code="Beta-154123";
   };
   R_Date("Tres Ventanas 12b-VI-450 (Date)",10030,170)
   {
    site="Tres Ventanas 12b-VI-450";
    material="";
    latitude="-12.21";
    longitude="-76.38";
    country="Peru";type="open-air";
    high altitude="n";
    technology="Paiján";
    megafauna kill/scavenge="n";
    code="I-3091";
   };
  };
  Boundary("End Paijan");
 };
};
```

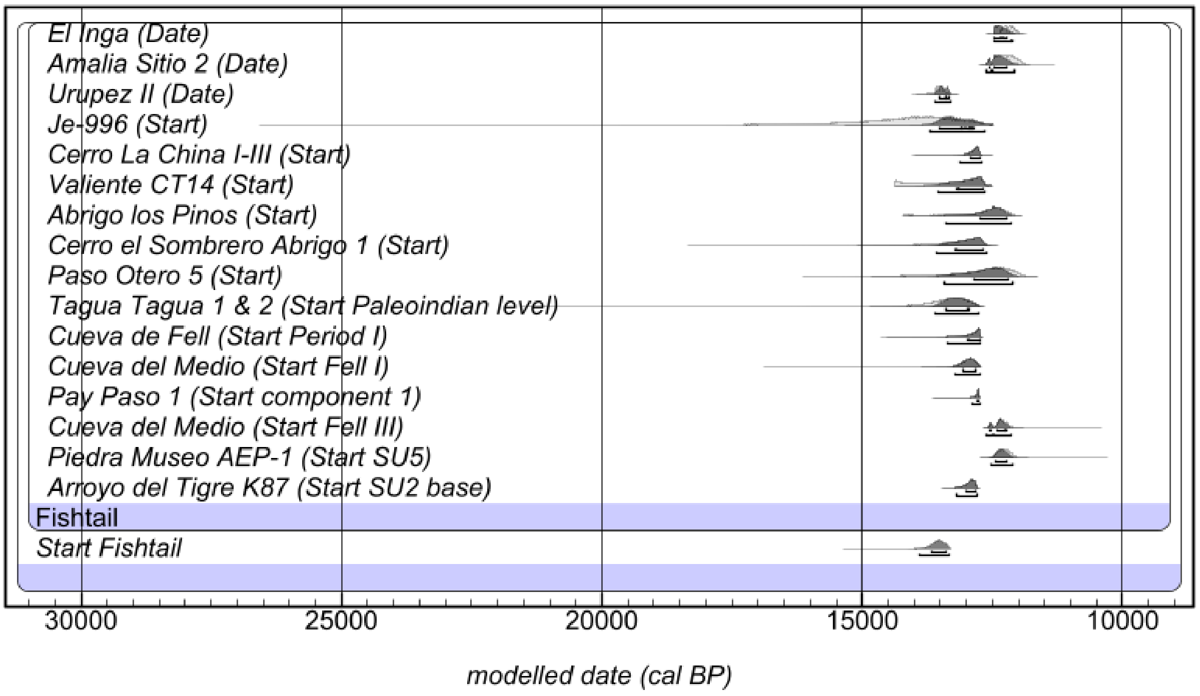


**Supplementary Figure 15.** Bayesian model showing the estimated start of Fishtail technology (13900-13320 cal BP), including all pertaining ACR/YD-aged cultural components. The latter were entered as either start boundaries or single ages given available data (noted next to each component name). Brackets beneath each age estimate show 68.3% and 95.4% CI.

```
Plot()
{
 Curve("SHCal20","shcal20.14c");
 Sequence()
 {
  Boundary("Start Fishtail");
  Phase("Fishtail")
  {
   Date(Prior("Arroyo del Tigre K87 (Start SU2 base)","K87_S_base.prior"))
   {
    site="Arroyo del Tigre K87";
    latitude=-30.4;
    longitude=-57.8;
    country="Uruguay";
    type="open-air";
    high altitude="n";
    province="Paraguay-Parana Lowlands";
    technology="Fishtail";
    megafauna kill/scavenge="n";
   };
   Date(Prior("Piedra Museo AEP-1 (Start SU5)","PM_S_SU5.prior"))
   {
    site="Piedra Museo AEP-1";
    latitude=-47.895;
    longitude=-67.867778;
    country="Argentina";
    type="rockshelter";
    high altitude="n";
    province="Patagonian Plateau";
    technology="Fishtail";
    megafauna kill/scavenge="y";
   };
   Date(Prior("Cueva del Medio (Start Fell III)","CM_S_Fell_III.prior"))
   {
    site="Cueva del Medio";
    latitude=-51.576;
    longitude=-72.583;
    country="Chile";
    type="cave";
    high altitude="n";
    province="Patagonian Plateau";
    technology="Fishtail";
    megafauna kill/scavenge="y";
   };
   Date(Prior("Pay Paso 1 (Start component 1)","PP_S_1.prior"))
   {
    site="Pay Paso 1";
    latitude=-30.2685;
    longitude=-57.460639;
    country="Uruguay";
    type="open-air";
    high altitude="n";
    province="Paraguay-Parana Lowlands";
    technology="Fishtail";
    megafauna kill/scavenge="y";
   };
   Date(Prior("Cueva del Medio (Start Fell I)","CM_S_Fell_I.prior"))
   {
    site="Cueva del Medio";
    latitude=-51.576;
    longitude=-72.583;
    country="Chile";
    type="cave";
    high altitude="n";
    province="Patagonian Plateau";
    technology="Fishtail";
    megafauna kill/scavenge="y";
   };
   Date(Prior("Cueva de Fell (Start Period I)","CF_S_Fell.prior"))
   {
    site="Cueva de Fell";
    latitude=-52.044444;
    longitude=-70.056389;
    country="Chile";
    type="rockshelter";
    high altitude="n";
    province="Patagonian Plateau";
    technology="Fishtail";
    megafauna kill/scavenge="y";
   };
   Date(Prior("Tagua Tagua 1 & 2 (Start Paleoindian level)","TT_S_Pleis.prior"))
   {
    site="Tagua Tagua 1 & 2";
    latitude=-34.4;
    longitude=-71.2;
    country="Chile";
    type="open-air";
    high altitude="n";
    province="Andes";
    technology="Fishtail";
    megafauna kill/scavenge="y";
   };
   Date(Prior("Paso Otero 5 (Start)","PO5_S.prior"))
   {
    site="Paso Otero 5";
    latitude=-38.2022;
    longitude=-59.1327;
    country="Argentina";
    type="open-air";
    high altitude="n";
    province="Paraguay-Parana Lowlands";
    technology="Fishtail";
    megafauna kill/scavenge="y";
   };
   Date(Prior("Cerro el Sombrero Abrigo 1 (Start)","CSA1_S.prior"))
   {
    site="Cerro el Sombrero Abrigo 1";
    latitude=-37.8166;
    longitude=-58.5666;
    country="Argentina";
    type="rockshelter";
    high altitude="n";
    province="Paraguay-Parana Lowlands";
    technology="Fishtail";
    megafauna kill/scavenge="n";
   };
   Date(Prior("Abrigo los Pinos (Start)","AlP_S.prior"))
   {
    site="Abrigo los Pinos";
    latitude=-37.4;
    longitude=-58.9;
    country="Argentina";
    type="rockshelter";
    high altitude="n";
    province="Paraguay-Parana Lowlands";
    technology="Fishtail";
    megafauna kill/scavenge="n";
   };
   Date(Prior("Valiente CT14 (Start)","Valiente_S.prior"))
   {
    site="Valiente CT14";
    latitude=-32.028333;
    longitude=-71.160833;
    country="Chile";
    type="open-air";
    high altitude="n";
    province="Andes";
    technology="Fishtail";
    megafauna kill/scavenge="n";
   };
   Date(Prior("Cerro La China I-III (Start)","CLC_S.prior"))
   {
    site="Cerro La China I-III";
    latitude=-37.95;
    longitude=-58.6166;
    country="Argentina";
    type="rockshelter";
    high altitude="n";
    province="Paraguay-Parana Lowlands";
    technology="Fishtail";
    megafauna kill/scavenge="n";
   };
   Date(Prior("Je-996 (Start)","Je_S.prior"))
   {
    site="Je-996";
    latitude=-7.059;
    longitude=-79.397;
    country="Peru";
    type="open-air";
    high altitude="n";
    province="Andes";
    technology="Paiján and Fishtail";
    megafauna kill/scavenge="n";
   };
   R_Date("Urupez II (Date)",11690,80)
   {
    site="Urupez II";
    material="charcoal";
    latitude="-34.83";
    longitude="-55.32";
    country="Uruguay"type="open-air";
    high altitude="n";
    technology="Fishtal";
    megafauna kill/scavenge="n";
    code="Beta-211398";
   };
   R_Date("Amalia Sitio 2 (Date)",10425,75)
   {
    site="Amalia Sitio 2";
    material="carbon vegetal";
    latitude="-37.8";
    longitude="-58.10";
    country="Argentina"type="rockshelter";
    high altitude="n";
    technology="Fishtail";
    megafauna kill/scavenge="n";
    code="AA-35499";
   };
   R_Date("El Inga (Date)",10410,35)
   {
    site="El Inga";
    material="charcoal";
    latitude="-0.05";
    longitude="-78.55";
    country="Ecuador"type="open-air";
    high altitude="y";
    technology="Fishtal";
    megafauna kill/scavenge="n";
    code="PRI-13-029";
   };
  };
  Boundary("End Fishtail");
 };
};
```

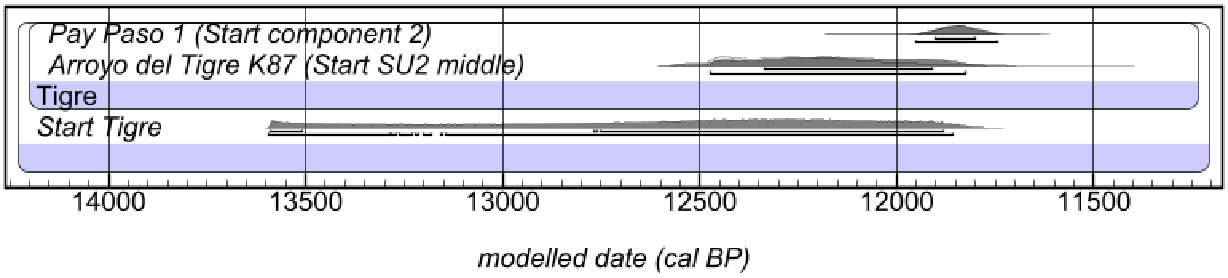

**Supplementary Figure 16.** Bayesian model showing the estimated start of Tigre technology (13593-11858 cal BP), including all pertaining ACR/YD-aged cultural components. The latter were entered as either start boundaries or single ages given available data (noted next to each component name). Brackets beneath each age estimate show 68.3% and 95.4% CI.

```
Plot()
{
 Sequence()
 {
  Boundary("Start Tigre");
  Phase("Tigre")
  {
   Date(Prior("Arroyo del Tigre K87 (Start SU2 middle)","K87_S_mid.prior"))
   {
    site="Arroyo del Tigre K87";
    latitude=-30.4;
    longitude=-57.8;
    country="Uruguay";
    type="open-air";
    high altitude="n";
    province="Paraguay-Parana Lowlands";
    technology="Tigre";
    megafauna kill/scavenge="n";
   };
   Date(Prior("Pay Paso 1 (Start component 2)","PP_S_2.prior"))
   {
    site="Pay Paso 1";
    latitude=-30.2685;
    longitude=-57.460639;
    country="Uruguay";
    type="open-air";
    high altitude="n";
    province="Paraguay-Parana Lowlands";
    technology="Tigre";
    megafauna kill/scavenge="y";
   };
  };
  Boundary("End Tigre");
 };
};
```

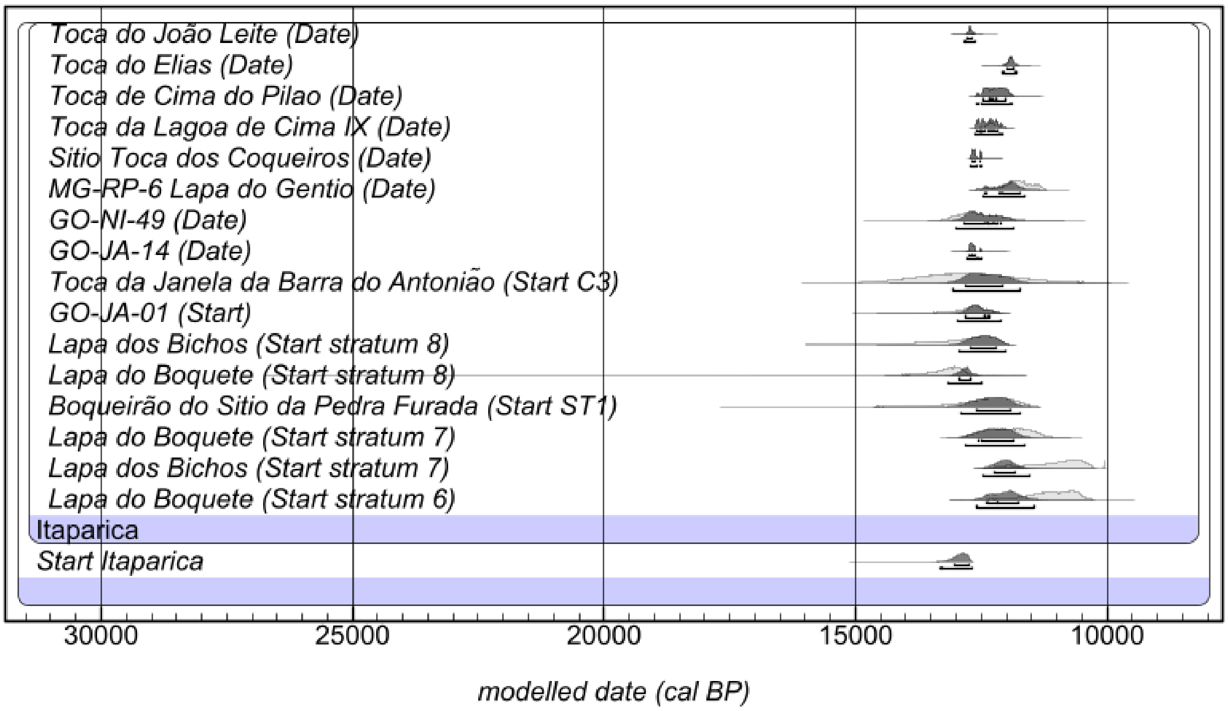


**Supplementary Figure 17.** Bayesian model showing the estimated start of Itaparica technology (13308-12697 cal BP), including all pertaining ACR/YD-aged cultural components. The latter were entered as either start boundaries or single ages given available data (noted next to each component name). Brackets beneath each age estimate show 68.3% and 95.4% CI.

```
Plot()
{
 Sequence()
 {
  Boundary("Start Itaparica");
  Phase("Itaparica")
  {
 Date(Prior("Lapa do Boquete (Start stratum 6)","LBo_S_6.prior"))
   {
   site="Lapa do Boquete";
   latitude=-14.98;
   longitude=-44.44;
   country="Brazil";
   type="cave";
   high altitude="n";
   province="Brazilian Highlands";
   technology="Itaparica";
   megafauna kill/scavenge="n";
   };
   R_Date("GO-JA-02 (Date)",10120,80)
   {
   site="GO-JA-02";
   material="charcoal";
   latitude="-18.45";
   longitude="-52.01";
   country="Brazil";type="rockshelter";
   high altitude="n";
   technology="Itaparica";
   megafauna kill/scavenge="n";
   code="SI-3108";
   };
   Date(Prior("Lapa dos Bichos (Start stratum 7)","LBi_S_7.prior"))
   {
   site="Lapa dos Bichos";
   latitude=-14.98;
   longitude=-44.44;
   country="Brazil";
   type="cave";
   high altitude="n";
   province="Brazilian Highlands";
   technology="Itaparica";
   megafauna kill/scavenge="n";
   };
   Date(Prior("Lapa do Boquete (Start stratum 7)","LBo_S_7.prior"))
   {
   site="Lapa do Boquete";
   latitude=-14.98;
   longitude=-44.44;
   country="Brazil";
   type="cave";
   high altitude="n";
   province="Brazilian Highlands";
   technology="Itaparica";
   megafauna kill/scavenge="n";
   };
   Date(Prior("Boqueirão do Sitio da Pedra Furada (Start ST1)","PF_S_ST1.prior"))
   {
   site="Boqueirão do Sitio da Pedra Furada";
   latitude=-8.85;
   longitude=-42.55;
   country="Brazil";
   type="rockshelter";
   high altitude="n";
   province="Brazilian Highlands";
   technology="Itaparica";
   megafauna kill/scavenge="n";
   };
   Date(Prior("Lapa do Boquete (Start stratum 8)","LBo_S_8.prior"))
   {
   site="Lapa do Boquete";
   latitude=-14.98;
   longitude=-44.44;
   country="Brazil";
   type="cave";
   high altitude="n";
   province="Brazilian Highlands";
   technology="Itaparica";
   megafauna kill/scavenge="n";
   };
   Date(Prior("Lapa dos Bichos (Start stratum 8)","LBi_S_8.prior"))
   {
   site="Lapa dos Bichos";
   latitude=-14.98;
   longitude=-44.44;
   country="Brazil";
   type="cave";
   high altitude="n";
   province="Brazilian Highlands";
   technology="Itaparica";
   megafauna kill/scavenge="n";
   };
   Date(Prior("GO-JA-01 (Start)","GOJA01_S.prior"))
   {
   site="GO-JA-01";
   latitude=-18.28638889;
   longitude=-52.04777778;
   country="Brazil";
   type="rockshelter";
   high altitude="n";
   province="Brazilian Highlands";
   technology="Itaparica";
   megafauna kill/scavenge="n";
   };
   Date(Prior("Toca da Janela da Barra do Antonião (Start C3)","TDJ_S_C3.prior"))
   {
   site="Toca da Janela da Barra do Antonião";
   latitude=-8.802222;
   longitude=-42.433056;
   country="Brazil";
   type="rockshelter";
   high altitude="n";
   province="Brazilian Highlands";
   technology="Itaparica";
   megafauna kill/scavenge="n";
   };
   Curve("SHCal20","shcal20.14c");
   R_Date("GO-JA-14 (Date)",10740,85)
   {
   site="GO-JA-14";
   material="charcoal";
   latitude="-14.48";
   longitude="-49.47";
   country="Brazil";type="rockshelter";
   high altitude="n";
   technology="Itaparica";
   megafauna kill/scavenge="n";
   code="SI-3111";
   };
   R_Date("GO-NI-49 (Date)",10750,300)
   {
   site="GO-NI-49";
   material="charcoal";
   latitude="-23.00";
   longitude="-65.45";
   country="Argentina";type="rockshelter";
   high altitude="y";
   technology="uniface/biface";
   megafauna kill/scavenge="n";
   code="SI-2769";
   };
   R_Date("MG-RP-6 Lapa do Gentio (Date)",10190,120)
   {
   site="MG-RP-6 Lapa do Gentio";
   material="charcoal";
   latitude="-16.26";
   longitude="-46.05";
   country="Brazil";type="rockshelter";
   high altitude="n";
   technology="Itaparica";
   megafauna kill/scavenge="n";
   code="SI-6837";
   };
   R_Date("Sitio Toca dos Coqueiros (Date)",10640,50)
   {
   site="Sitio Toca dos Coqueiros";
   material="hair";
   latitude="-29.72";
   longitude="-56.66";
   country="Brazil";type="open-air";
   high altitude="n";
   technology="projectile point";
   megafauna kill/scavenge="n";
   code="Beta-104571";
   };
   R_Date("Toca da Lagoa de Cima IX (Date)",10480,50)
   {
   site="Toca da Lagoa de Cima IX";
   material="charcoal";
   latitude="-8.7";
   longitude="-42.6";
   country="Brazil";type="rockshelter/cave";
   high altitude="n";
   technology="Itaparica";
   megafauna kill/scavenge="n";
   code="Beta-233909";
   };
   R_Date("Toca de Cima do Pilao (Date)",10390,80)
   {
   site="Toca de Cima do Pilao";
   material="unreported";
   latitude="-8.87";
   longitude="-42.59";
   country="Brazil";type="rockshelter/cave";
   high altitude="n";
   technology="Itaparica";
   megafauna kill/scavenge="n";
   code="Beta-27345";
   };
   R_Date("Toca do Elias (Date)",10270,35)
   {
   site="Toca do Elias";
   material="charcoal";
   latitude="-8.84";
   longitude="-42.56";
   country="Brazil";type="rockshelter/cave";
   high altitude="n";
   technology="Itaparica";
   megafauna kill/scavenge="n";
   code="CAMS-95865";
   };
   R_Date("Toca do João Leite (Date)",10800,70)
   {
   site="Toca do João Leite";
   material="charcoal";
   latitude="-8.74";
   longitude="-42.74";
   country="Brazil";type="rockshelter/cave";
   high altitude="n";
   technology="Itaparica";
   megafauna kill/scavenge="n";
   code="Beta-220088";
   };
  };
  Boundary("End Itaparica");
 };
};
```

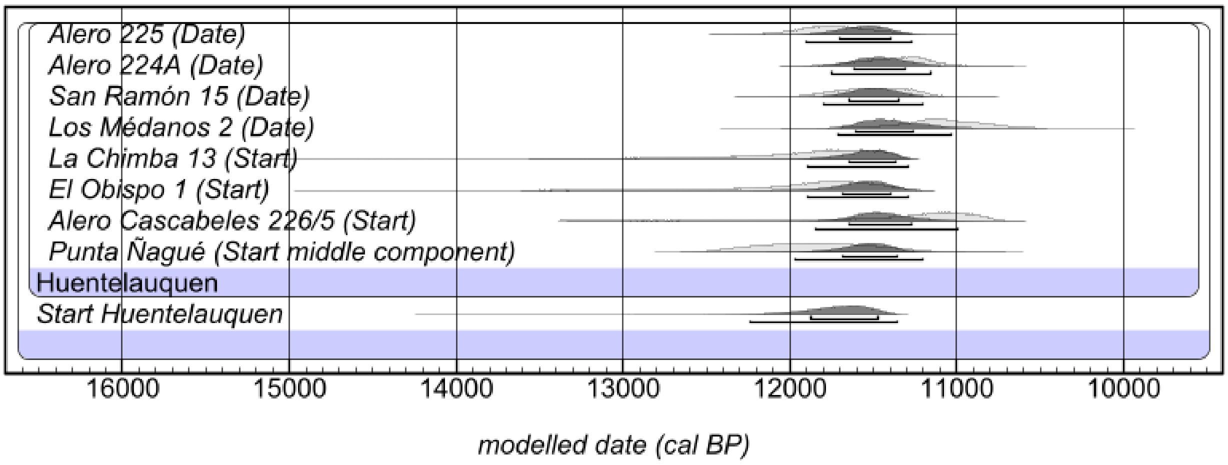


**Supplementary Figure 18.** Bayesian model showing the estimated start of Huentelauquén technology (12240-11360 cal BP), including all pertaining ACR/YD-aged cultural components. The latter were entered as either start boundaries or single ages given available data (noted next to each component name). Brackets beneath each age estimate show 68.3% and 95.4% CI.

```
Plot()
{
 Sequence()
 {
  Boundary("Start Huentelauquen");
  Phase("Huentelauquen")
  {
   Date(Prior("Punta Ñagué (Start middle component)","PN_S_middle.prior"))
   {
    site="Punta Ñagué";
    latitude=-31.85;
    longitude=-71.52;
    country="Chile";
    type="open-air";
    high altitude="n";
    province="Andes";
    technology="Huentelauquén";
    megafauna kill/scavenge="n";
   };
   Date(Prior("Alero Cascabeles 226/5 (Start)","AC_226_S.prior"))
   {
    site="Alero Cascabeles 226/5";
    latitude=-25.2;
    longitude=-70.4;
    country="Chile";
    type="rockshelter";
    high altitude="n";
    province="Andes";
    technology="Huentelauquén";
    megafauna kill/scavenge="n";
   };
   Date(Prior("El Obispo 1 (Start)","EO1_S.prior"))
   {
    site="El Obispo 1";
    latitude=-26.3;
    longitude=-70.6;
    country="Chile";
    type="open-air";
    high altitude="n";
    province="Andes";
    technology="Huentelauquén";
    megafauna kill/scavenge="n";
   };
   Date(Prior("La Chimba 13 (Start)","LC13_S.prior"))
   {
    site="La Chimba 13";
    latitude=-23.545556;
    longitude=-70.368889;
    country="Chile";
    type="open-air";
    high altitude="n";
    province="Andes";
    technology="Huentelauquén";
    megafauna kill/scavenge="n";
   };
   Date(Prior("Los Médanos 2 (Date)","LM2_D.prior"))
   {
    site="Los Médanos 2";
    latitude=-26.3;
    longitude=-70.6;
    country="Chile";
    type="open-air";
    high altitude="n";
    province="southern Andes";
    technology="Huentelauquén";
    megafauna kill/scavenge="n";
   };
   Date(Prior("San Ramón 15 (Date)","SR15_D.prior"))
   {
    site="San Ramón 15";
    latitude=-25.3;
    longitude=-70.4;
    country="Chile";
    type="open-air";
    high altitude="n";
    province="southern Andes";
    technology="Huentelauquén";
    megafauna kill/scavenge="n";
   };
   Date(Prior("Alero 224A (Date)","A224A_D.prior"))
   {
    site="Alero 224A";
    latitude=-25.2;
    longitude=-70.4;
    country="Chile";
    type="rockshelter";
    high altitude="n";
    province="southern Andes";
    technology="Huentelauquén";
    megafauna kill/scavenge="n";
   };
   Date(Prior("Alero 225 (Date)","A225_D.prior"))
   {
    site="Alero 225";
    latitude=-25.2;
    longitude=-70.4;
    country="Chile";
    type="rockshelter";
    high altitude="n";
    province="southern Andes";
    technology="Huentelauquén";
    megafauna kill/scavenge="n";
   };
  };
  Boundary("End Huentelauquen");
 };
};
```

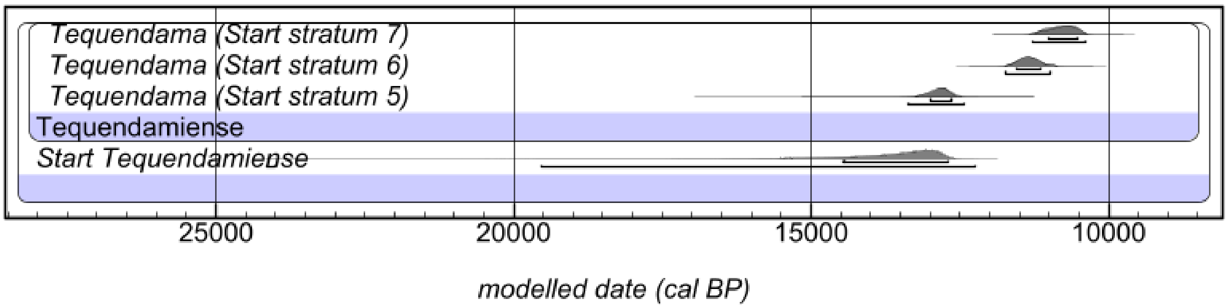

**Supplementary Figure 19.** Bayesian model showing the estimated start of Tequedamiense technology (24155-12245 cal BP) from start boundaries within the type-site. Brackets beneath each age estimate show 68.3% and 95.4% CI.

```
Plot()
{
 Sequence()
 {
  Boundary("Start Tequendamiense");
  Sequence("Tequendamiense")
  {
   Date(Prior("Tequendama (Start stratum 5)","Teq_S_5.prior"))
   {
    site="Tequendama";
    latitude=4.533;
    longitude=-74.275;
    country="Colombia";
    type="rockshelter";
    high altitude="n";
    province="Andes";
    technology="Tequendamiense";
    megafauna kill/scavenge="y";
   };
   Date(Prior("Tequendama (Start stratum 6)","Teq_S_6.prior"))
   {
    site="Tequendama";
    latitude=4.533;
    longitude=-74.275;
    country="Colombia";
    type="rockshelter";
    high altitude="n";
    province="Andes";
    technology="Tequendamiense";
    megafauna kill/scavenge="y";
   };
   Date(Prior("Tequendama (Start stratum 7)","Teq_S_7.prior"))
   {
    site="Tequendama";
    latitude=4.533;
    longitude=-74.275;
    country="Colombia";
    type="rockshelter";
    high altitude="n";
    province="Andes";
    technology="Tequendamiense";
    megafauna kill/scavenge="y";
   };
  };
  Boundary("End Tequendamiense");
 };
};
```

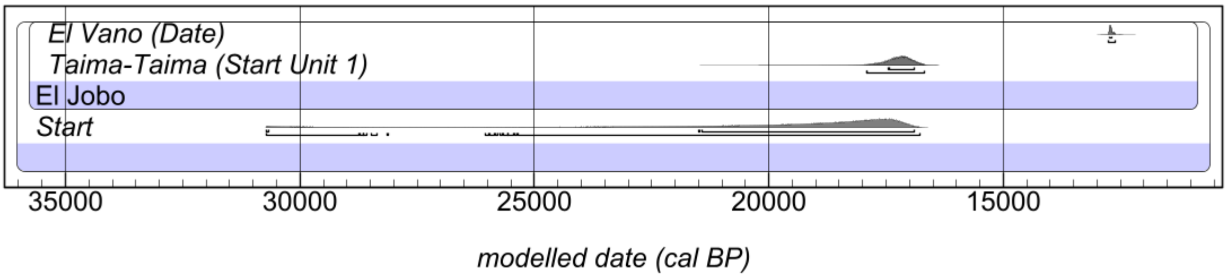


**Supplementary Figure 20.** Bayesian model showing the estimated start of El Jobo technology (17910-16675 cal BP at 95.4% CI or 17455-16885 at 68.2% CI) from start boundaries within the type-site. Brackets beneath each age estimate show 68.3% and 95.4% CI.

```
Plot()
{
 Sequence()
 {
  Boundary("Start");
  Phase("El Jobo")
  {
   Date(Prior("Taima-Taima (Start Unit 1)","TT_U1_S.prior"))
   {
    site="Taima-Taima";
    latitude=11.43;
    longitude=-69.75;
    country="Venezuela";
    type="open-air";
    high altitude="n";
    province="northern Andes";
    technology="El Jobo";
    megafauna kill/scavenge="y";
   };
   R_Date("El Vano (Date)",10710,60)
   {
    site="El Vano";
    material="unreported";
    latitude="9.62";
    longitude="-70";
    country="Venezuela";
    type="open-air";
    high altitude="n";
    technology="El Jobo";
    megafauna kill/scavenge="y";
    code="Beta-95602";
   };
  };
  Boundary("End");
 };
};
```

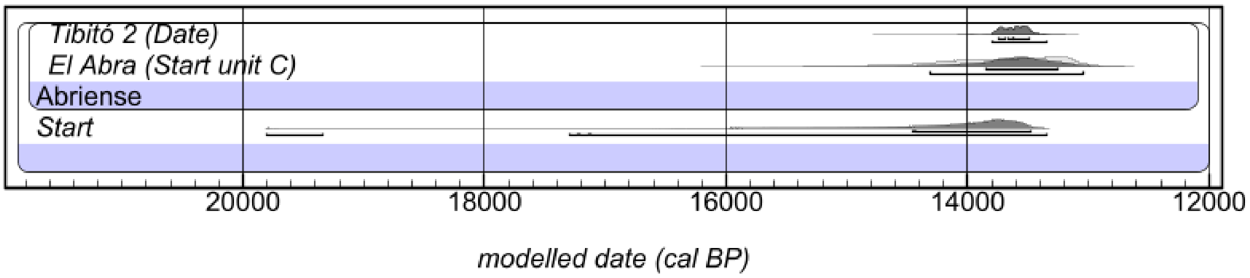


**Supplementary Figure 21.** Bayesian model showing the estimated start of Abriense technology (19805-13340 cal BP at 95.4% CI or 14460-13470 cal BP at 68.2% CI) from start boundaries within the type-site. Brackets beneath each age estimate show 68.3% and 95.4% CI.

```
Plot()
{
 Sequence()
 {
  Boundary("Start");
  Phase("Abriense")
  {
Date(Prior("El Abra (Start unit C)","EA_S_C.prior"))
 {
  site="El Abra";
  latitude=5.02;
  longitude=-73.937;
  country="Colombia";
  type="rockshelter";
  high altitude="n";
  province="northern Andes";
  technology="Abriense";
  megafauna kill/scavenge="n";
 };
 R_Date("Tibitó 2 (Date)",11740,110)
 {
  site="Tibitó 2";
  material="";
  latitude="4.98";
  longitude="-73.98";
  country="Colombia";
  type="open-air";
  high altitude="n";
  technology="Abriense";
  megafauna kill/scavenge="y";
  code="GrN-9375";
 };
  };
  Boundary("End");
 };
};
```

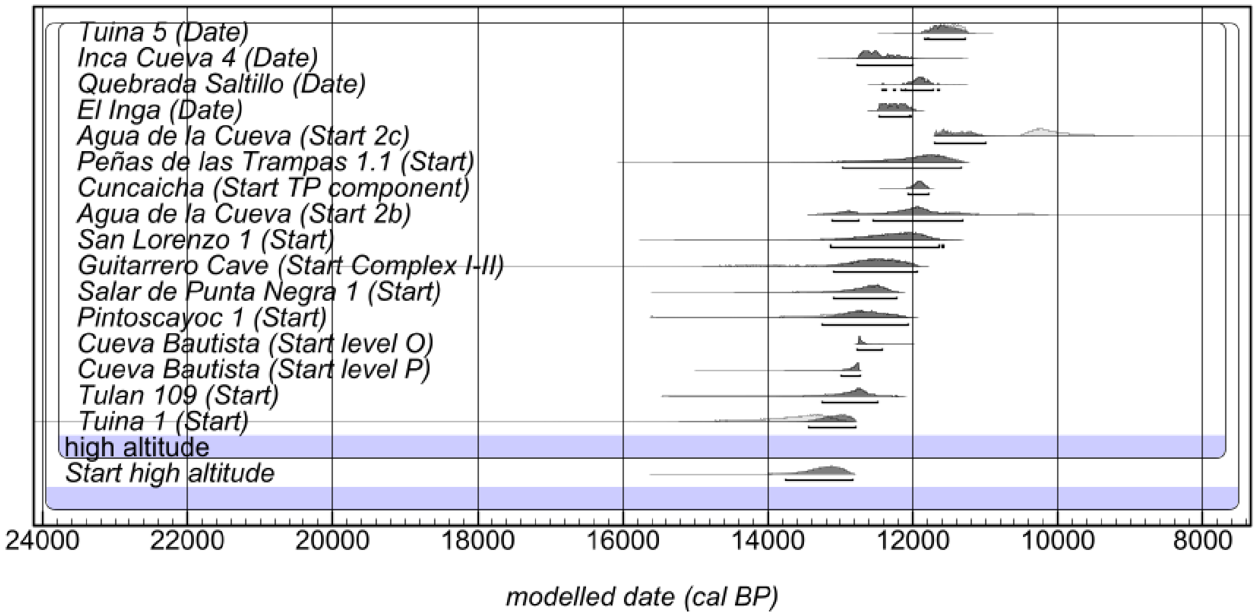


**Supplementary Figure 22.** Bayesian model showing the estimated start of high-altitude occupation (13750-12830 cal BP at 95.4% or 13385-12935 cal BP at 68.3% CI), including all pertaining ACR/YD-aged cultural components. The latter were entered as either start boundaries or single ages given available data (noted next to each component name). Brackets beneath each age estimate show 68.3% and 95.4% CI.

```
Plot()
{
 Sequence()
 {
  Boundary("Start high altitude");
  Phase("high altitude")
  {
   Date(Prior("Tuina 1 (Start)","Tuina1_S.prior"))
   {
    site="Tuina 1";
    latitude=-22.8;
    longitude=-68.3;
    country="Chile";
    type="rockshelter";
    high altitude="y";
    province="southern Andes";
    technology="uniface/biface";
    megafauna kill/scavenge="n";
   };
   Date(Prior("Tulan 109 (Start)","Tulan9_S.prior"))
   {
    site="Tulan 109";
    latitude=-23.6;
    longitude=-67.9;
    country="Chile";
    type="cave";
    high altitude="y";
    province="southern Andes";
    technology="uniface/biface";
    megafauna kill/scavenge="n";
   };
   Date(Prior("Cueva Bautista (Start level P)","CB_S_P.prior"))
   {
    site="Cueva Bautista";
    latitude=-21.338;
    longitude=-67.574;
    country="Bolivia";
    type="rockshelter";
    high altitude="y";
    province="central Andes";
    technology="uniface/biface";
    megafauna kill/scavenge="n";
   };
   Date(Prior("Cueva Bautista (Start level O)","CB_S_O.prior"))
   {
    site="Cueva Bautista";
    latitude=-21.338;
    longitude=-67.574;
    country="Bolivia";
    type="rockshelter";
    high altitude="y";
    province="central Andes";
    technology="uniface/biface";
    megafauna kill/scavenge="n";
   };
   Date(Prior("Pintoscayoc 1 (Start)","Pinto1_S.prior"))
   {
    site="Pintoscayoc 1";
    latitude=-22.9444;
    longitude=-65.4182;
    country="Argentina";
    type="rockshelter";
    high altitude="y";
    province="southern Andes";
    technology="projectile point";
    megafauna kill/scavenge="n";
   };
   Date(Prior("Salar de Punta Negra 1 (Start)","SPN1_S.prior"))
   {
    site="Salar de Punta Negra 1";
    latitude=-24.466667;
    longitude=-68.883333;
    country="Chile";
    type="open-air";
    high altitude="y";
    province="southern Andes";
    technology="projectile point";
    megafauna kill/scavenge="n";
   };
   Date(Prior("Guitarrero Cave (Start Complex I-II)","GC_S_I_II.prior"))
   {
    site="Guitarrero Cave";
    latitude=-9.20069;
    longitude=-77.7098;
    country="Peru";
    type="cave";
    high altitude="y";
    province="central Andes";
    technology="uniface/biface";
    megafauna kill/scavenge="n";
   };
   Date(Prior("San Lorenzo 1 (Start)","SLoren1_S.prior"))
   {
    site="San Lorenzo 1";
    latitude=-23.4;
    longitude=-67.9;
    country="Chile";
    type="cave";
    high altitude="y";
    province="southern Andes";
    technology="uniface/biface";
    megafauna kill/scavenge="n";
   };
   Date(Prior("Agua de la Cueva (Start 2b)","AdC_2b_S.prior"))
   {
    site="Agua de la Cueva";
    latitude=-32.6169;
    longitude=-69.1636;
    country="Argentina";
    type="rockshelter";
    high altitude="y";
    province="southern Andes";
    technology="uniface/biface";
    megafauna kill/scavenge="n";
   };
   Date(Prior("Cuncaicha (Start TP component)","Cun_S_TP.prior"))
   {
    site="Cuncaicha";
    latitude=-15.373;
    longitude=-72.618;
    country="Peru";
    type="rockshelter";
    high altitude="y";
    province="central Andes";
    technology="projectile point";
    megafauna kill/scavenge="n";
   };
   Date(Prior("Peñas de las Trampas 1.1 (Start)","PdlT1_S.prior"))
   {
    site="Peñas de las Trampas 1.1";
    latitude=-26.0175;
    longitude=-67.35;
    country="Argentina";
    type="rockshelter";
    high altitude="y";
    province="southern Andes";
    technology="uniface/biface";
    megafauna kill/scavenge="n";
   };
   Date(Prior("Agua de la Cueva (Start 2c)","AdC_2c_S.prior"))
   {
    site="Agua de la Cueva";
    latitude=-32.6169;
    longitude=-69.1636;
    country="Argentina";
    type="rockshelter";
    high altitude="y";
    province="southern Andes";
    technology="uniface/biface";
    megafauna kill/scavenge="n";
   };
   Curve("SHCal20","shcal20.14c");
   R_Date("El Inga (Date)",10410,35)
   {
    site="El Inga";
    material="charcoal";
    latitude="-0.05";
    longitude="-78.55";
    country="Ecuador";type="open-air";
    high altitude="y";
    technology="Fishtail";
    megafauna kill/scavenge="n";
    code="PRI-13-029";
   };
R_Date("Quebrada Saltillo (Date)",10260,60)
{
 site="RS-I-66 Milton Almeida";
 material="charcoal";
 latitude="-24.3";
 longitude="-68.9";
 country="Chile";
 type="open-air";
 high altitude="y";
 province=y;
 technology="projectile point";
 megafauna kill/scavenge="n";
 code="Beta-191578";
};
   R_Date("Inca Cueva 4 (Date)",10620,140)
   {
    site="Inca Cueva 4";
    material="";
    latitude="-7.06";
    longitude="-79.40";
    country="Peru";type="open-air";
    high altitude="n";
    technology="Paiján";
    megafauna kill/scavenge="n";
    code="LP-137";
   };
   R_Date("Tuina 5 (Date)",10060,70)
   {
    site="Tuina 5";
    material="";
    latitude="-22.8";
    longitude="-68.3";
    country="Chile";type="cave";
    high altitude="y";
    technology="uniface/biface";
    megafauna kill/scavenge="n";
    code="Beta-107120";
   };
  };
  Boundary("End high altitude");
 };
};
```

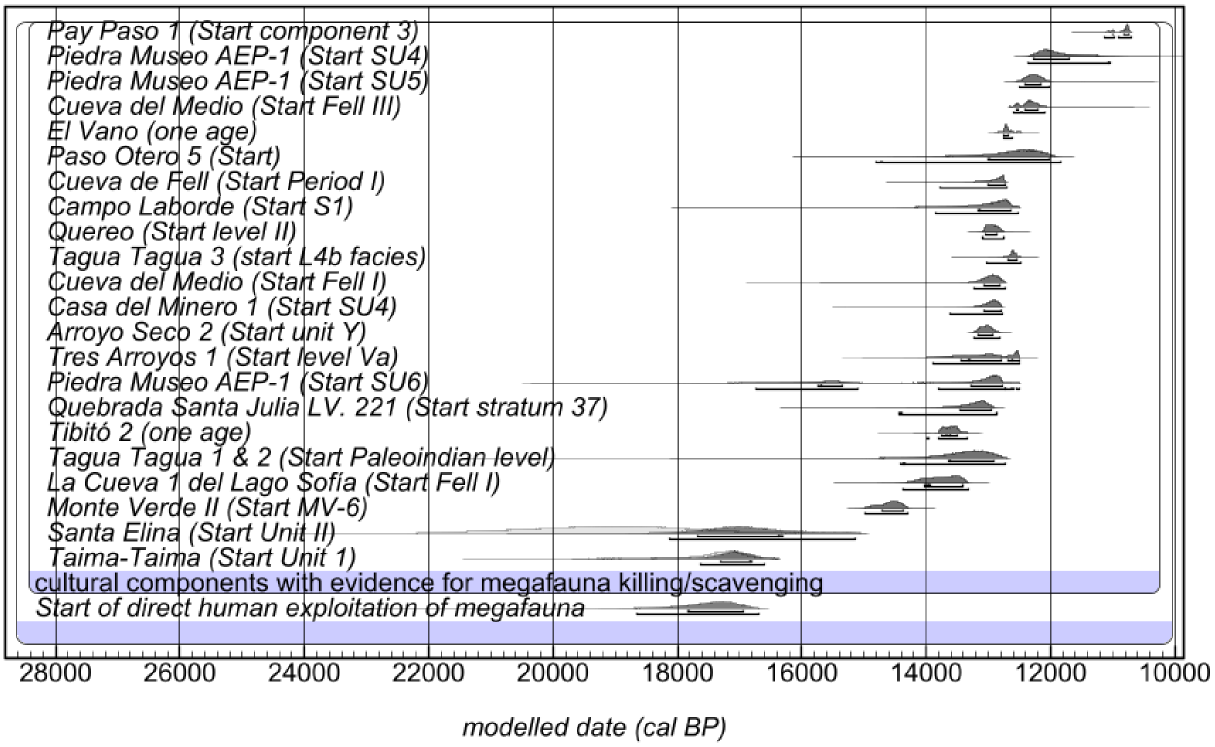


**Supplementary Figure 23.** Bayesian model showing the estimated start human exploitation of megafauna (18660-16695 cal BP), including all pertaining ACR/YD-aged cultural components with direct evidence for killing/scavenging as start boundaries. Brackets beneath each age estimate show 68.3% and 95.4% CI.

```
Plot()
{
 Sequence()
 {
  Boundary("Start of direct human exploitation of megafauna");
  Phase("cultural components with evidence for megafauna killing/scavenging")
  {
   Date(Prior("Taima-Taima (Start Unit 1)","TT_U1_S.prior"))
   {
    site="Taima-Taima";
    latitude=11.43;
    longitude=-69.75;
    country="Venezuela";
    type="open-air";
    high altitude="n";
    province="northern Andes";
    technology="El Jobo";
    megafauna kill/scavenge="y";
   };
   Date(Prior("Santa Elina (Start Unit II)","SE_S_II.prior"))
   {
    site="Santa Elina";
    latitude=-15.4577778;
    longitude=-56.7925;
    country="Brazil";
    type="rockshelter";
    high altitude="n";
    province="Brazilian Highlands";
    technology="Itaparica";
    megafauna kill/scavenge="y";
   };
   Date(Prior("Monte Verde II (Start MV-6)","MVII_S_MV6.prior"))
   {
    site="Monte Verde II";
    latitude=-41.51;
    longitude=-73.2;
    country="Chile";
    type="open-air";
    high altitude="n";
    province="southern Andes";
    technology="uniface/biface";
    megafauna kill/scavenge="y";
   };
   Date(Prior("La Cueva 1 del Lago Sofía (Start Fell I)","CLS_S_Fell_I.prior"))
   {
    site="La Cueva 1 del Lago Sofía";
    latitude=-51.53;
    longitude=-72.33;
    country="Chile";
    type="cave";
    high altitude="n";
    province="Patagonian Plateau";
    technology="uniface/biface";
    megafauna kill/scavenge="y";
   };
   Date(Prior("Tagua Tagua 1 & 2 (Start Paleoindian level)","TT_S_Pleis.prior"))
   {
    site="Tagua Tagua 1 & 2";
    latitude=-34.4;
    longitude=-71.2;
    country="Chile";
    type="open-air";
    high altitude="n";
    province="southern Andes";
    technology="Fishtail";
    megafauna kill/scavenge="y";
   };
   R_Date("Tibitó 2 (one age)",11740,110)
   {
    site="Tibitó 2";
    material="";
    latitude="4.98";
    longitude="-73.98";
    country="Colombia";type="open-air";
    high altitude="n";
    technology="Abriense";
    megafauna kill/scavenge="y";
    code="GrN-9375";
   };
   Date(Prior("Quebrada Santa Julia LV. 221 (Start stratum 37)","QSJ_S_37.prior"))
   {
    site="Quebrada Santa Julia LV. 221";
    latitude=-31.836917;
    longitude=-71.488278;
    country="Chile";
    type="open-air";
    high altitude="n";
    province="southern Andes";
    technology="projectile point";
    megafauna kill/scavenge="y";
   };
   Date(Prior("Piedra Museo AEP-1 (Start SU6)","PM_S_SU6.prior"))
   {
    site="Piedra Museo AEP-1";
    latitude=-47.895;
    longitude=-67.867778;
    country="Argentina";
    type="rockshelter";
    high altitude="n";
    province="Patagonian Plateau";
    technology="uniface/biface";
    megafauna kill/scavenge="y";
   };
   Date(Prior("Tres Arroyos 1 (Start level Va)","TA1_S_Va.prior"))
   {
    site="Tres Arroyos 1";
    latitude=-53.383;
    longitude=-68.783;
    country="Chile";
    type="rockshelter";
    high altitude="n";
    province="Patagonian Plateau";
    technology="uniface/biface";
    megafauna kill/scavenge="y";
   };
   Date(Prior("Arroyo Seco 2 (Start unit Y)","AS2_S_unitY.prior"))
   {
    site="Arroyo Seco 2";
    latitude=-38.360556;
    longitude=-60.244167;
    country="Argentina";
    type="open-air";
    high altitude="n";
    province="Paraguay-Parana Lowlands";
    technology="uniface/biface";
    megafauna kill/scavenge="y";
   };
   Date(Prior("Casa del Minero 1 (Start SU4)","CM1_S_SU4.prior"))
   {
    site="Casa del Minero 1";
    latitude=-48.575833;
    longitude=-68.9125;
    country="Argentina";
    type="cave";
    high altitude="n";
    province="Patagonian Plateau";
    technology="uniface/biface";
    megafauna kill/scavenge="y";
   };
   Date(Prior("Cueva del Medio (Start Fell I)","CM_S_Fell_I.prior"))
   {
    site="Cueva del Medio";
    latitude=-51.576;
    longitude=-72.583;
    country="Chile";
    type="cave";
    high altitude="n";
    province="Patagonian Plateau";
    technology="Fishtail";
    megafauna kill/scavenge="y";
   };
   Date(Prior("Tagua Tagua 3 (start L4b facies)","TT4_S.prior"))
   {
    site="Tagua Tagua 3";
    latitude=-34.4;
    longitude=-71.2;
    country="Chile";
    type="open-air";
    high altitude="n";
    province="southern Andes";
    technology="uniface/biface";
    megafauna kill/scavenge="y";
   };
   Date(Prior("Quereo (Start level II)","Q_S_QuereoII.prior"))
   {
    site="Quereo";
    latitude=-31.93;
    longitude=-71.51;
    country="Chile";
    type="open-air";
    high altitude="n";
    province="southern Andes";
    technology="uniface/biface";
    megafauna kill/scavenge="y";
   };
   Date(Prior("Campo Laborde (Start S1)","CL_S_1.prior"))
   {
    site="Campo Laborde";
    latitude=-37.1;
    longitude=-60.38;
    country="Argentina";
    type="open-air";
    high altitude="n";
    province="Paraguay-Parana Lowlands";
    technology="uniface/biface";
    megafauna kill/scavenge="y";
   };
   Date(Prior("Cueva de Fell (Start Period I)","CF_S_Fell.prior"))
   {
    site="Cueva de Fell";
    latitude=-52.044444;
    longitude=-70.056389;
    country="Chile";
    type="rockshelter";
    high altitude="n";
    province="Patagonian Plateau";
    technology="Fishtail";
    megafauna kill/scavenge="y";
   };
   Date(Prior("Paso Otero 5 (Start)","PO5_S.prior"))
   {
    site="Paso Otero 5";
    latitude=-38.2022;
    longitude=-59.1327;
    country="Argentina";
    type="open-air";
    high altitude="n";
    province="Paraguay-Parana Lowlands";
    technology="Fishtail";
    megafauna kill/scavenge="y";
   };
   R_Date("El Vano (one age)",10710,60)
   {
    site="El Vano";
    material="unreported";
    latitude="9.62";
    longitude="-70";
    country="Venezuela";type="open-air";
    high altitude="n";
    technology="El Jobo";
    megafauna kill/scavenge="y";
    code="Beta-95602";
   };
   Date(Prior("Cueva del Medio (Start Fell III)","CM_S_Fell_III.prior"))
   {
    site="Cueva del Medio";
    latitude=-51.576;
    longitude=-72.583;
    country="Chile";
    type="cave";
    high altitude="n";
    province="Patagonian Plateau";
    technology="Fishtail";
    megafauna kill/scavenge="y";
   };
   Date(Prior("Piedra Museo AEP-1 (Start SU5)","PM_S_SU5.prior"))
   {
    site="Piedra Museo AEP-1";
    latitude=-47.895;
    longitude=-67.867778;
    country="Argentina";
    type="rockshelter";
    high altitude="n";
    province="Patagonian Plateau";
    technology="Fishtail";
    megafauna kill/scavenge="y";
   };
   Date(Prior("Piedra Museo AEP-1 (Start SU4)","PM_S_SU4.prior"))
   {
    site="Piedra Museo AEP-1";
    latitude=-47.895;
    longitude=-67.867778;
    country="Argentina";
```

```
    type="rockshelter";
    high altitude="n";
    province="Patagonian Plateau";
    technology="uniface/biface";
    megafauna kill/scavenge="y";
   };
   Date(Prior("Pay Paso 1 (Start component 3)","PP_S_3.prior"))
   {
    site="Pay Paso 1";
    latitude=-30.2685;
    longitude=-57.460639;
    country="Uruguay";
    type="open-air";
    high altitude="n";
    province="Paraguay-Parana Lowlands";
    technology="Pay Paso";
    megafauna kill/scavenge="y";
   };
  };
  Boundary("End");
 };
};
```

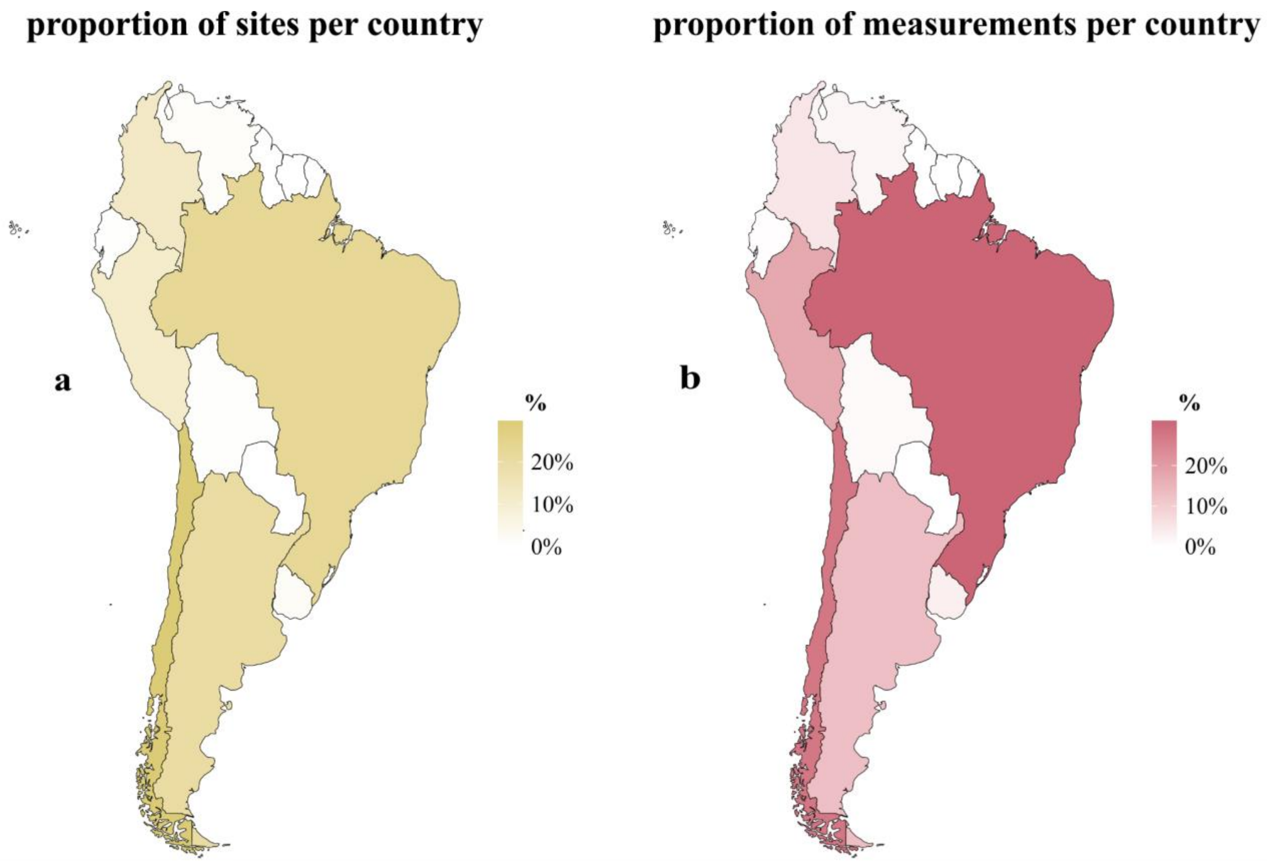


**Supplementary Figure 24.** Maps showing **a.** the proportion of archaeological sites per country (yellow), and **b.** the proportion of chronometric measurements per country (pink).

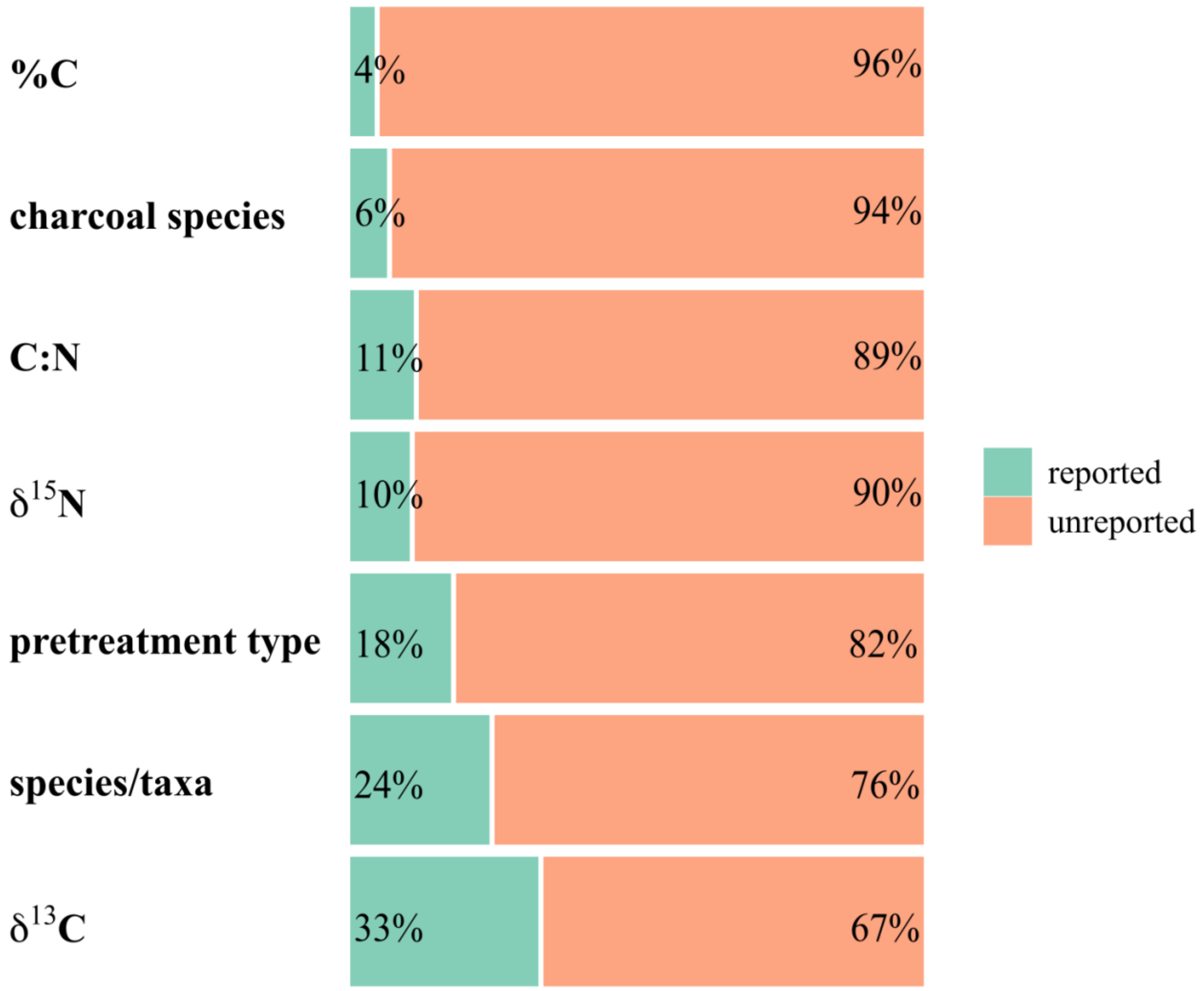


**Supplementary Figure 25.** Percentage of unreported (orange) versus reported (green) information pertaining to radiocarbon measurements in the database (Supplementary Data 1). C:N and $\delta^{15}N$ values are for bone samples not listed as 'apatite'.

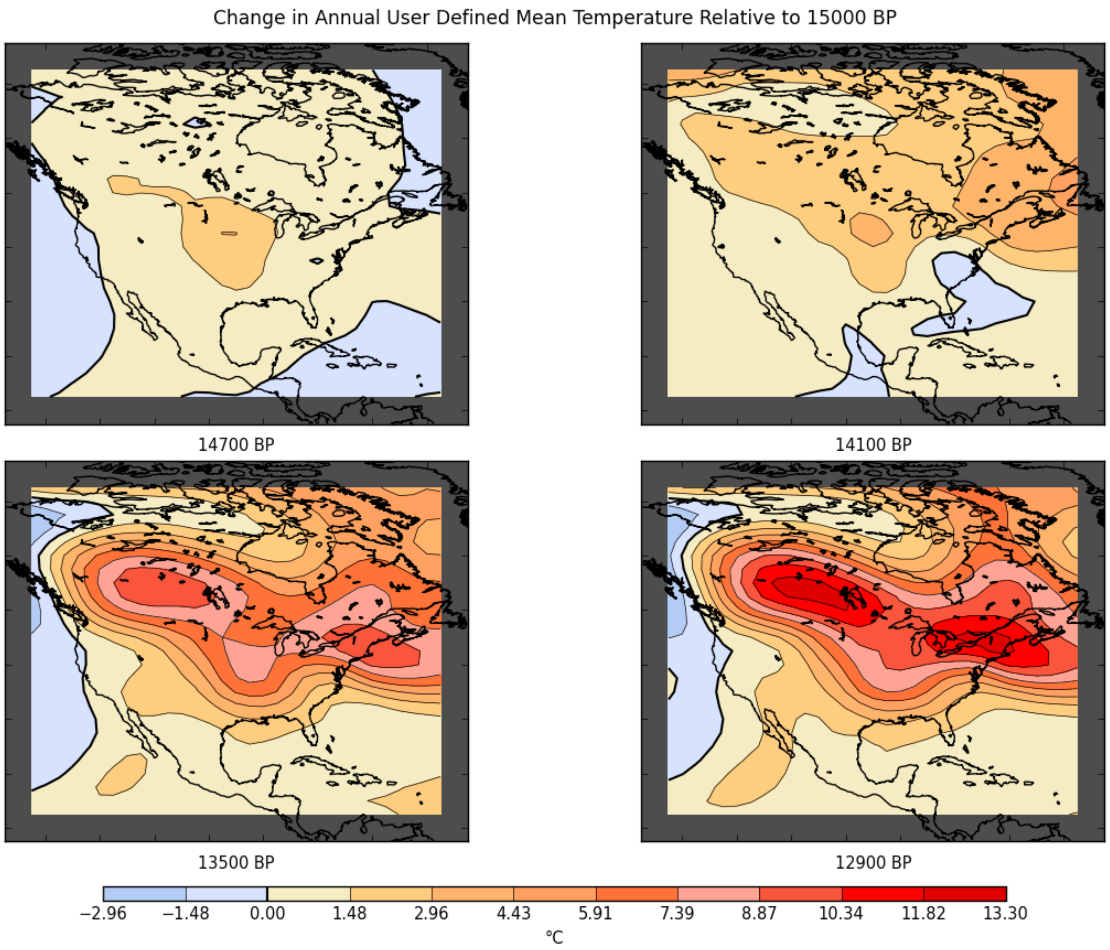


**Supplementary Figure 26.** Annual mean temperature during ACR relative to 15ka in North America, based on TraCE21k simulations[4,5] processed through PaleoView[6]. It is noteworthy that temperature changes in North America are considerably more pronounced compared to those in South America (Supplementary Figure 27). Colour gradient at bottom of figure.

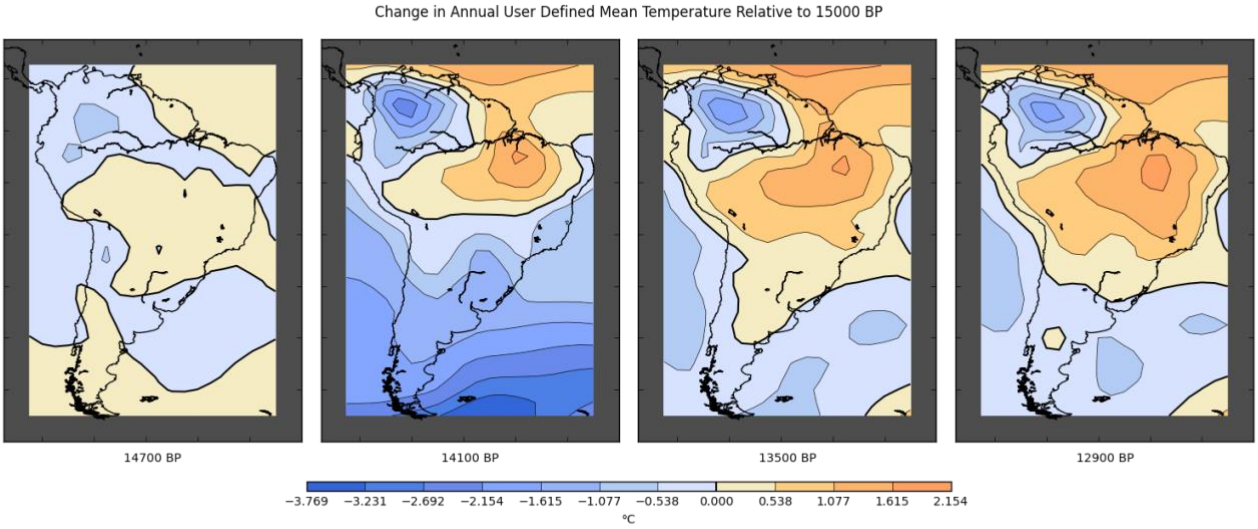


**Supplementary Figure 27.** Annual mean temperature during ACR relative to 15ka in South America, based on TraCE21k simulations[4,5] processed through PaleoView[6]. It is noteworthy that temperature changes in South America are considerably less pronounced compared to those in North America (Supplementary Figure 26). Colour gradient at bottom of figure.

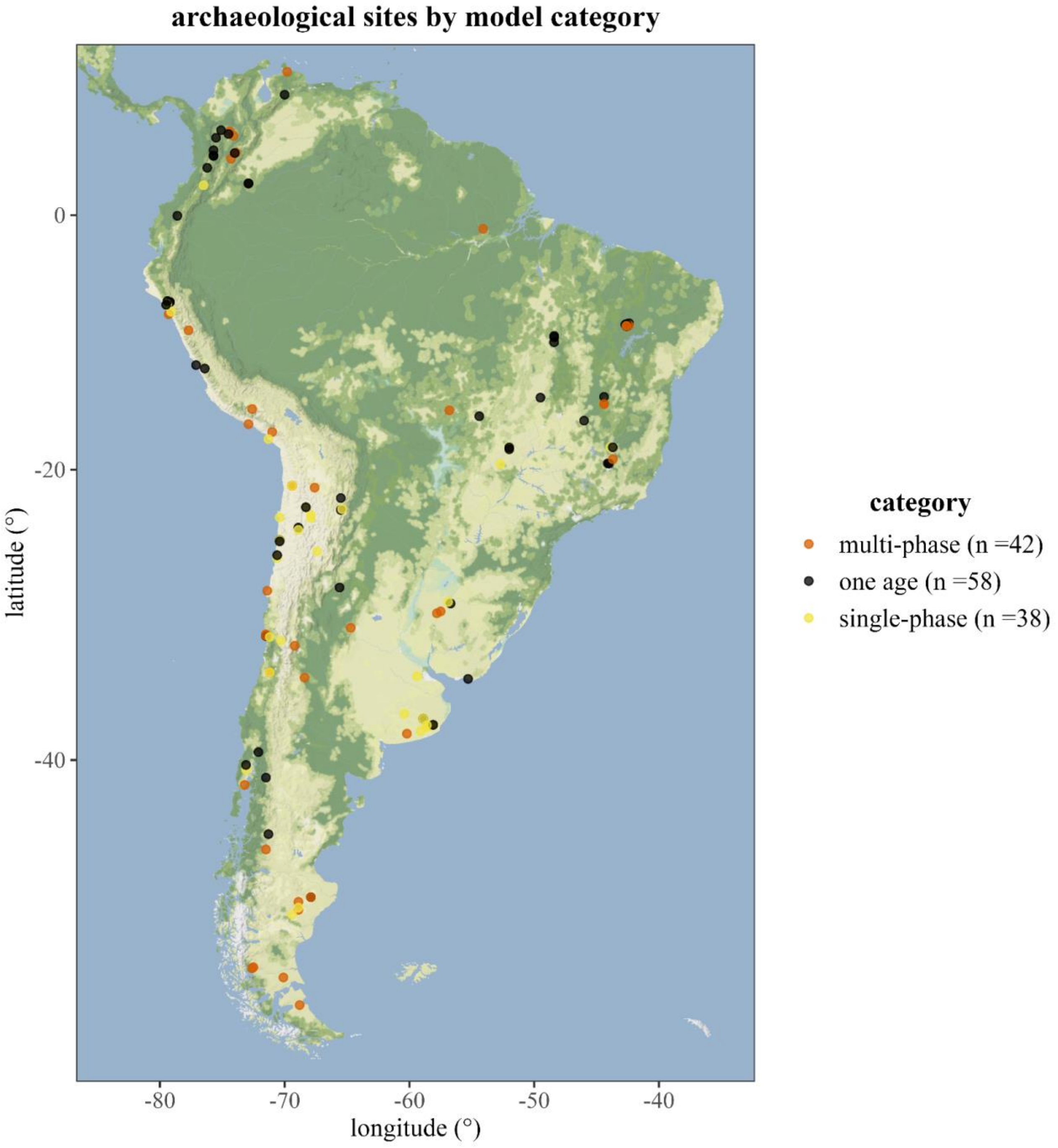


**Supplementary Figure 28.** Map showing the spatial distribution of archaeological data included in the modelling according to model category: multi-phase (orange), single-phase (black) and one age (yellow).

**Supplementary Notes**

This section includes lithic tradition descriptions, site reports (including Bayesian modelling analysis), sensitivity testing, and OxCal code (either in the last section or after each figure).

## 1. Lithic traditions

### *1.2. Abriense/Tequendamiense*

Found in Colombia, Abriense and Tequendamiense are both uniface traditions. The latter is more elaborate, contains exogenous material, and is only found in basal levels of the site Tequendama[7–9]. Although generally treated as distinct traditions, Nieuwenhuis' (2002)[10] argues that they are the same (but see Correal Urrego's[11] rebuttal).

### *1.3. El Jobo*

Found in Venezuela, El Jobo tradition or industry includes lanceolate projectile points and other bifacial tools[12,13].

### *1.4. Fishtail/Fell*

Originally found in Chile in Cueva de Fell[14], Fishtail (or 'Fell') projectile points are located across South America, and can be both fluted or unfluted[15–24].

### *1.5. Huentelauquén*

Found in coastal northern Chile, the Huentelauquén tradition is characterised by lanceolate projectile points and disc-shaped stones[25–29].

### *1.6. Itaparica*

Found in central and northwestern Brazil, the Itaparica tradition or technocomplex includes unifacial limaces or *lesmas*[30–33].

### *1.7. Paiján*

Found in coastal northern Peru, the Paiján tradition includes bifacial, projectile points with a narrow basal stem and unifaces (limaces)[34–38]. Paiján is different from the Fishtail tradition, in that it contains bi- and uni-facial tools[37].

*1.8. Pay Paso and Tigre*

Found in Uruguay at the K87 type site, Tigre and Pay Paso complexes include bifacial point technology. Tigre includes stemmed projectile points and asymmetrical bifaces, whilst the later Pay Paso is represented by points with short, highly-concave stems[24].

## 2. Site reports

### *2.1. Argentina*

2.1.1. Agua de la Cueva

Agua de la Cueva is a high-altitude (2,900 m.a.s.l.) rockshelter located in Argentina (-32.6169, -69.1636), containing lithic technology and faunal remains (including *Lama guanicoe*)[39–42]. There are three lithostratigraphic units subdivided into five, with subunits 2b and 2c including the earliest archaeological evidence (see Figs. 2-3 in García et al.[39]).

Bayesian modelling estimates the start of subunits 2b and 2c at 13265-11290 cal BP and 11270-8740 cal BP, respectively, with six outliers (five in subunit 2a and one in 2b; all charcoal; see Supplementary Figure 29 and OxCal code). These are likely due to bioturbation caused by *Ctenomys Medocinus* activity[42].

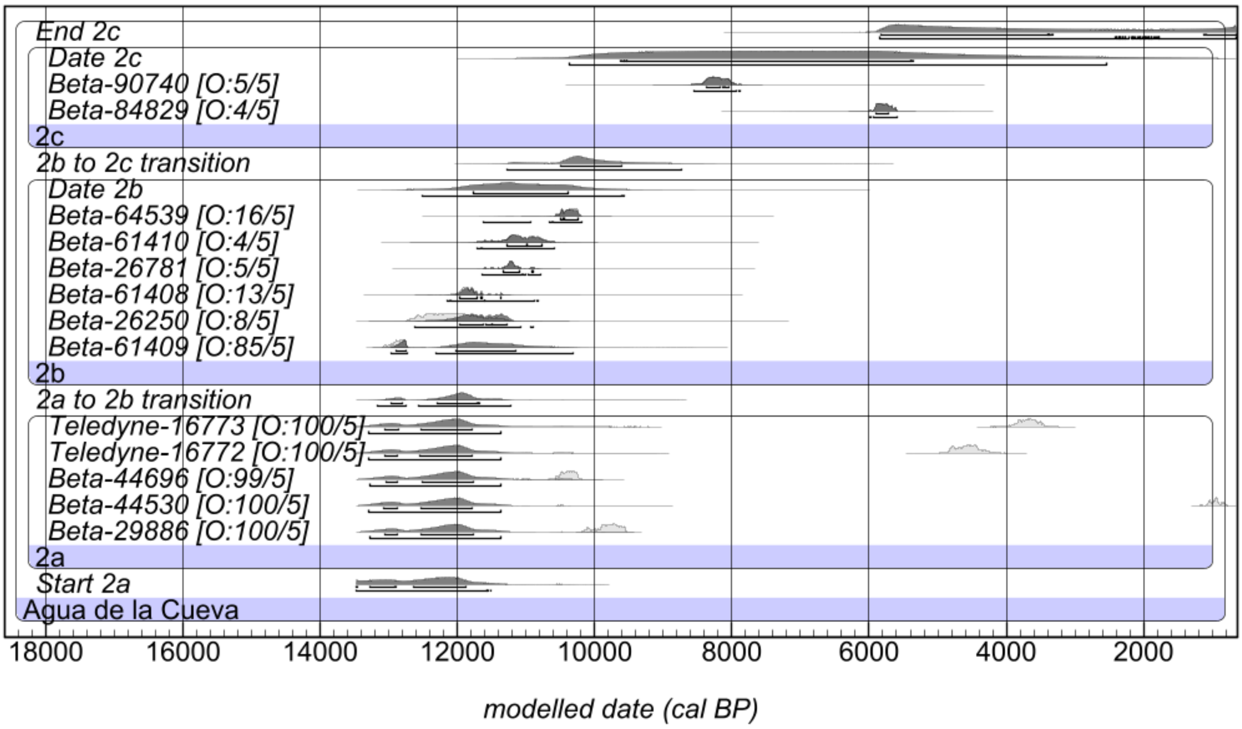


**Supplementary Figure 29.** Bayesian age model for Agua de la Cueva. Brackets beneath each age estimate show 68.3% and 95.4% CI. Outlier analysis output is noted as 'O:posterior probability/prior probability'.

```
Plot()
{
 Curve("SHCal20","shcal20.14c");
 Outlier_Model("General",T(5),U(0,4),"t");
 Sequence("Agua de la Cueva")
 {
  Boundary("Start 2a");
  Phase("2a")
  {
   R_Date("Beta-29886",8800,70)
   {
    Outlier("General", 0.05);
    context="subunit 2a, pit C54";
   };
   R_Date("Beta-44530",1090,60)
   {
    Outlier("General", 0.05);
    context="subunit 2a, pit G33a";
   };
   R_Date("Beta-44696",9250,70)
   {
    Outlier("General", 0.05);
    context="subunit 2a, pit E33b";
   };
   R_Date("Teledyne-16772",4090,110)
   {
    Outlier("General", 0.05);
    context="subunit 2a, pit B49";
   };
   R_Date("Teledyne-16773",3460,100)
   {
    Outlier("General", 0.05);
    context="subunit 2a, pit C51";
   };
  };
  Boundary("2a to 2b transition");
  Phase("2b")
  {
   R_Date("Beta-61409",10950,90)
   {
    Outlier("General", 0.05);
    context="subunit 2b, pit H10a";
   };
   R_Date("Beta-26250",10350,220)
   {
    Outlier("General", 0.05);
    context="subunit 2b, pit C46";
   };
   R_Date("Beta-61408",10240,60)
   {
    Outlier("General", 0.05);
    context="subunit 2b, pit H9d";
   };
   R_Date("Beta-26781",9840,90)
   {
    Outlier("General", 0.05);
    context="subunit 2b, pit C45";
   };
   R_Date("Beta-61410",9760,160)
   {
    Outlier("General", 0.05);
    context="subunit 2b, pit 110a";
   };
   R_Date("Beta-64539",9210,70)
   {
    Outlier("General", 0.05);
    context="subunit 2b, pit H8j2";
   };
   Date("Date 2b");
   Interval("Duration 2b");
  };
  Boundary("2b to 2c transition");
  Phase("2c")
  {
   R_Date("Beta-84829",5080,70)
   {
    Outlier("General", 0.05);
    context="subunit 2c, pit E28a";
   };
   R_Date("Beta-90740",7450,140)
   {
    Outlier("General", 0.05);
    context="subunit 2c, pit E29c";
   };
   Interval("Duration 2c");
   Date("Date 2c");
  };
  Boundary("End 2c");
 };
 Difference("Difference 2b vs 2c","2a to 2b transition","2b to 2c transition");
};
```

### 2.1.2. Arroyo Seco 2 (AS2)

Arroyo Seco 2 (AS2) is an open-air site in Argentina (-38.360556, -60.244167), containing lithic technology, extinct fauna (*Equus neogeus*, *Hippidion sp.*, *Toxodon platensis*, *Megatherium americanum*, *Eutatus seguini*, *Glossotherium robustum*, *Macrauchenia* sp., *Glyptodon* sp., and Camelidae cf. *Hemiauchenia*) and ceramics[43–47]. The site also contains ~50 human burials dated to the early- and mid-Holocene[48]. There are four stratigraphic units at the site as defined by sedimentological evidence: X, Y, S and Z (top to bottom) (see Fig. 3 in Politis et al.[44]). Unit X contains lithics, extant fauna, pottery and historical artefacts. Unit Y contains lithics and extant/extinct fauna, including Pleistocene mammals towards the middle and base. Unit S contains lithics and extinct fauna, as well as intrusive human burials (n=7). Unit Z presents a relatively limited number of faunal remains and lithics, multiple human burials (n=26), and evidence of vertical mixing (downward). All human burials located in units S and Z are intrusions that begin in the middle to upper sections of Unit Y. Over 50 radiocarbon dates have been produced, with 21 representing extinct mammals from units Y and S. There are no ages for younger unit X.

Using 21 dates on extinct fauna (including same-sample replicates), Bayesian modelling estimates the start of Unit S at 14065-13005 cal BP and Unit Y at 13240-12820 cal BP (with overlap at 95% CI; see Supplementary Figure 30 and OxCal code). There are a large number of major outliers within the sequence, however, with most of the replicates (3/4) failing $\chi^2$ testing. This is most likely due to differences in pretreatment protocols leading to ineffective sample decontamination. The earliest estimations for Unit S can be considered a *terminus post quem* for human activity at the site.

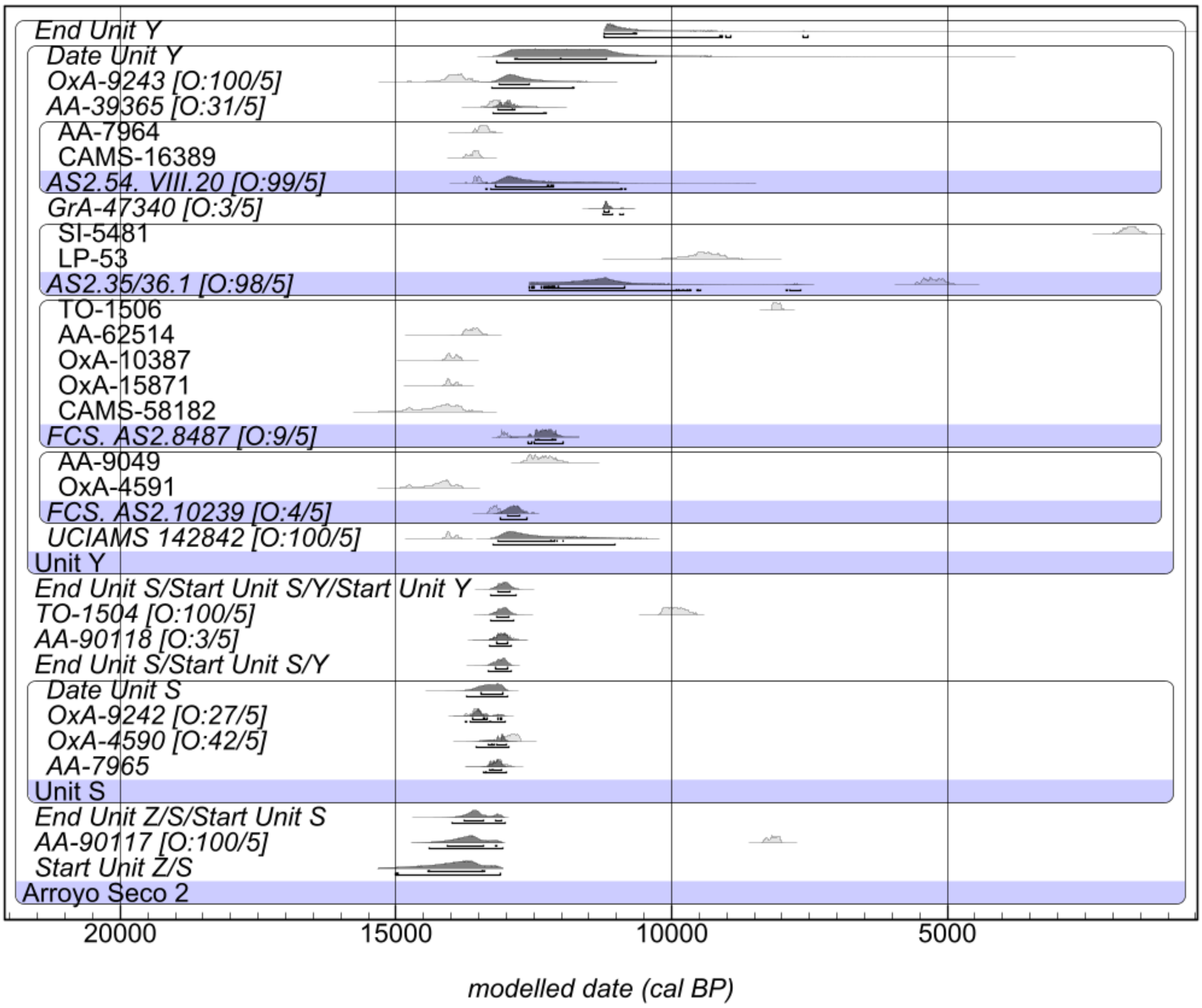


**Supplementary Figure 30.** Bayesian age model for Arroyo Seco 2. Brackets beneath each age estimate show 68.3% and 95.4% CI. Outlier analysis output is noted as 'O:posterior probability/prior probability'.

```
Plot()
{
 Curve("SHCal20","shcal20.14c");
 Outlier_Model("SSimple",N(0,2),0,"s");
 Outlier_Model("General",T(5),U(0,4),"t");
 Sequence("Arroyo Seco 2")
 {
  Boundary("Start Unit Z/S");
  R_Date("AA-90117",7388,74)
  {
   sample=FCS. AS2.15265;
   context="Unit S/Z";
   Outlier("General",0.05);
  };
  Boundary("End Unit Z/S/Start Unit S");
  Phase("Unit S")
  {
   R_Date("AA-7965",11250,105)
   {
    sample=AS2.53.1;
    context="Unit S";
   };
   R_Date("OxA-4590",11000,100)
   {
    Outlier("General",0.05);
    sample=AS2.54.IX.12;
    context="Unit S";
   };
   R_Date("OxA-9242",11730,70)
   {
    Outlier("General",0.05);
    sample=FCS. AS2.10369;
    context="Unit Base-Y/S";
   };
   Interval("Duration Unit S");
   Date("Date Unit S");
  };
  Boundary("End Unit S/Start Unit S/Y");
  R_Date("AA-90118",11190,110)
  {
   Outlier("General",0.05);
   sample=FCS. AS2.15000;
   context="Unit Y/S";
  };
  R_Date("TO-1504",8890,90)
  {
   Outlier("General",0.05);
   sample=AS2.45.17;
   context="Unit Base-Y/S";
  };
  Boundary("End Unit S/Start Unit S/Y/Start Unit Y");
  Phase("Unit Y")
  {
   R_Date("UCIAMS 142842",12170,45)
   {
    Outlier("General",0.05);
    sample=FCS. AS2.14260;
    context="Unit Base-Y";
   };
   R_Combine("FCS. AS2.10239")
   {
    R_Date("OxA-4591",12240,110)
    {
     Outlier("SSimple",0.05);
     sample=FCS. AS2.10239;
     context="Unit Base-Y";
    };
    R_Date("AA-9049",10500,90)
    {
     Outlier("SSimple",0.05);
     sample=FCS. AS2.10239;
     context="Unit Base-Y";
    };
    Outlier("General",0.05);
   };
   R_Combine("FCS. AS2.8487")
   {
    R_Date("CAMS-58182",12200,170)
    {
     Outlier("SSimple",0.05);
     sample=FCS. AS2.8487;
     context="Unit Base-Y";
    };
    R_Date("OxA-15871",12170,55)
    {
     Outlier("SSimple",0.05);
     sample=FCS. AS2.8487;
     context="Unit Base-Y";
    };
    R_Date("OxA-10387",12155,70)
    {
     Outlier("SSimple",0.05);
     sample=FCS. AS2.8487;
     context="Unit Base-Y";
    };
    R_Date("AA-62514",11770,120)
    {
     Outlier("SSimple",0.05);
     sample=FCS. AS2.8487;
     context="Unit Base-Y";
    };
    R_Date("TO-1506",7320,50)
    {
     Outlier("SSimple",0.05);
     sample=FCS. AS2.8487;
     context="Unit Base-Y";
    };
    Outlier("General",0.05);
   };
   R_Combine("AS2.35/36.1")
   {
    R_Date("LP-53",8470,240)
    {
     Outlier("SSimple",0.05);
     sample=AS2.35/36.1;
     context="Unit Base-Y";
    };
    R_Date("SI-5481",1800,110)
    {
     Outlier("SSimple",0.05);
     sample=AS2.35/36.1;
     context="Unit Base-Y";
    };
    Outlier("General",0.05);
   };
   R_Date("GrA-47340",9775,45)
   {
    Outlier("General",0.05);
    sample=FCS. AS2.10351;
    context="Unit Base-Y";
   };
   R_Combine("AS2.54. VIII.20")
   {
    R_Date("CAMS-16389",11750,70)
    {
     Outlier("SSimple",0.05);
     sample=AS2.54. VIII.20;
     context="Unit Base-Y";
    };
    R_Date("AA-7964",11590,90)
    {
     Outlier("SSimple",0.05);
     sample=AS2.54. VIII.20;
     context="Unit Base-Y";
    };
    Outlier("General",0.05);
   };
   R_Date("AA-39365",11320,110)
   {
    Outlier("General",0.05);
    sample=FCS. AS2.9177;
    context="Unit Base-Y";
   };
   R_Date("OxA-9243",12070,140)
   {
    Outlier("General",0.05);
    sample=FCS. AS2.1987;
    context="Unit Base-Y";
   };
   Interval("Duration Unit Y");
   Date("Date Unit Y");
  };
  Boundary("End Unit Y");
 };
 Difference("Difference S and Y","End Unit Z/S/Start Unit S","End Unit S/Start Unit S/Y/Start Unit Y");
};
```

### 2.1.3. Campo Laborde

Campo Laborde is an open-air site located in Argentina (-37.10, -60.38), containing lithic and bone tool (on *Megatherium americanum*) technology in association with *Megatherium americanum* (cut-marked rib) and glyptodont (*Neosclerocalyptus* sp. and *Doedicurus* sp.) remains[49–54]. There are six strata, with stratum 1 (paleoswamp) showing the earliest evidence of human activity (see Fig. 5 in Politis et al.[54]). The presence of lithic flakes at bracketing levels, Guerrero Member and stratum 2, is reported as vertical mixing (animal burrowing is observed). There is no other early Holocene evidence in the site.

A single-phase Bayesian model including all bone ages estimates the start of stratum 1 at 13870-12510 cal BP (see Supplementary Figure 31 and OxCal code). Sensitivity testing shows that this estimate is comparable to that obtained from a model containing only XAD collagen ages CAMS-171861, -171851 and -171852 (Supplementary Figure 32-33).

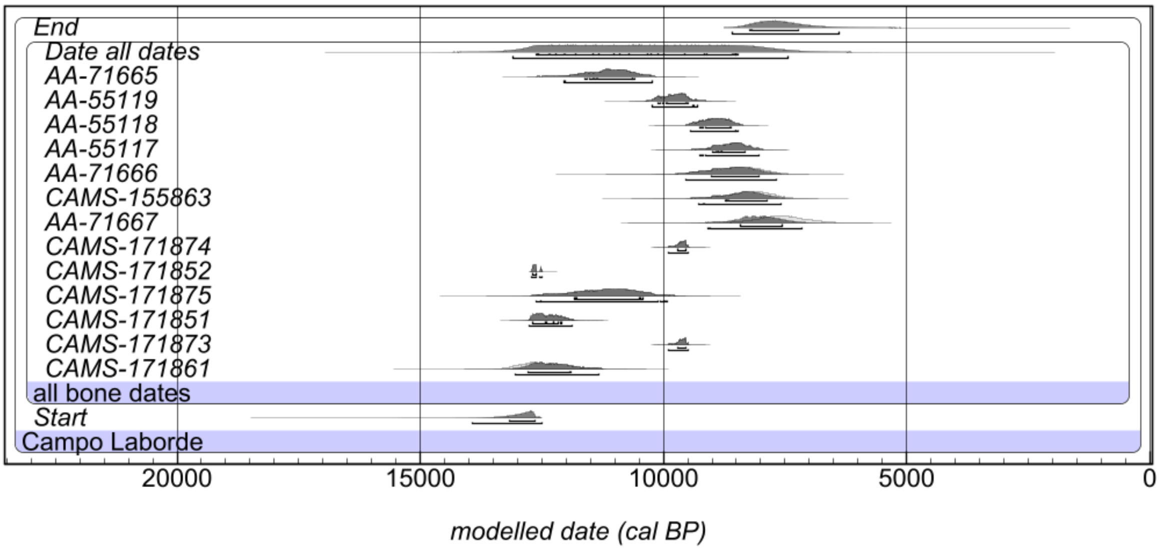


**Supplementary Figure 31.** Bayesian age model for Campo Laborde (all bone dates). Brackets beneath each age estimate show 68.3% and 95.4% CI.

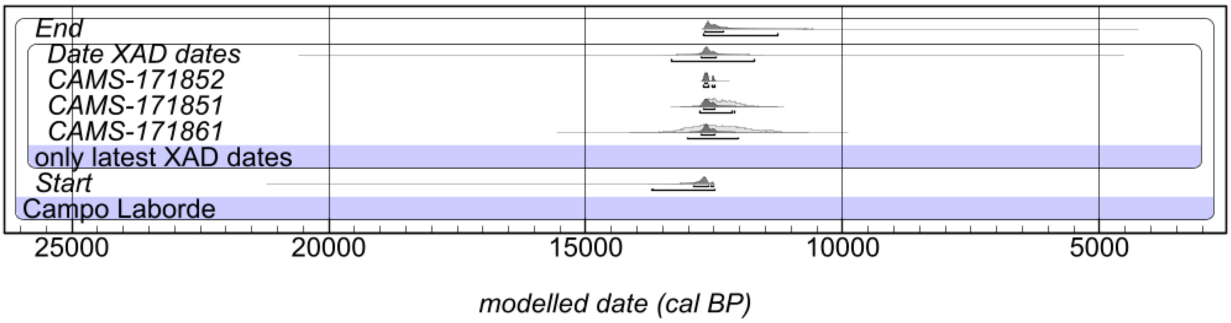


**Supplementary Figure 32.** Bayesian age model for Campo Laborde (only XAD dates). Brackets beneath each age estimate show 68.3% and 95.4% CI.

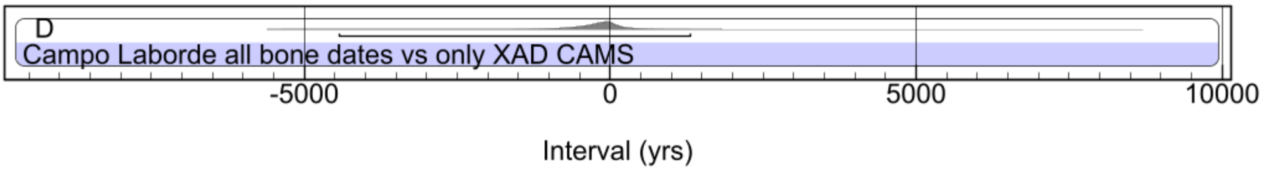


**Supplementary Figure 33.** Probability density function for the difference ('D') between the start of cultural activity at Campo Laborde using all bone dates (Supplementary Figure 31) vs only XAD measurements (Supplementary Figure 32). These results suggest that there is no significant difference between the modelled outputs, as the distributions include zero at 95.4% CI (black bracket beneath).

```
Plot()
{
 Curve("SHCal20","shcal20.14c");
 Sequence("Campo Laborde")
 {
  Boundary("Start");
  Phase("all bone dates")
  {
   R_Date("CAMS-171861",10690,380)
   {
    material="XAD KOH-extracted decalcified bone collagen";
   };
   R_Date("CAMS-171873",8670,80)
   {
    material="fulvic acids eluted from CAMS-171861 XAD resin";
   };
   R_Date("CAMS-171851",10570,170)
   {
    material="XAD KOH-extracted decalcified bone collagen";
   };
   R_Date("CAMS-171875",9720,450)
   {
    material="fulvic acids eluted from CAMS-171851 XAD resin";
   };
   R_Date("CAMS-171852",10655,35)
   {
    material="XAD KOH-extracted decalcified bone collagen";
   };
   R_Date("CAMS-171874",8670,80)
   {
    material="fulvic acids eluted from CAMS-171852 XAD resin";
   };
   R_Date("AA-71667",6740,480)
   {
    material="ABA treated bone";
   };
   R_Date("CAMS-155863",7380,410)
   {
    material="UF bone gelatin";
   };
   R_Date("AA-71666",7630,460)
   {
    material="ABA treated bone";
   };
   R_Date("AA-55117",7750,250)
   {
    material="gelatinized HCl-insoluble residue";
   };
   R_Date("AA-55118",8080,200)
   {
    material="gelatinized HCl-insoluble residue";
   };
   R_Date("AA-55119",8720,190)
   {
    material="gelatinized HCl-insoluble residue";
   };
   R_Date("AA-71665",9730,290)
   {
    material="ABA treated bone";
   };
   Interval("Duration all dates");
   Date("Date all dates");
  };
  Boundary("End");
 };
};
Plot()
{
 Curve("SHCal20","shcal20.14c");
 Sequence("Campo Laborde")
 {
  Boundary("Start");
  Phase("only latest XAD dates")
  {
   R_Date("CAMS-171861",10690,380)
   {
    material="XAD KOH-extracted decalcified bone collagen";
   };
   R_Date("CAMS-171851",10570,170)
   {
    material="XAD KOH-extracted decalcified bone collagen";
   };
   R_Date("CAMS-171852",10655,35)
   {
    material="XAD KOH-extracted decalcified bone collagen";
   };
   Date("Date XAD dates");
   Interval("Duration XAD dates");
  };
  Boundary("End");
 };
};
```

### 2.1.4. Casa del Minero 1

Casa del Minero 1 is a cave site located in Argentina (-48.575833, -68.912500), containing lithic technology and extinct faunal remains (*Hemiauchenia* cf. *paradoxa*)[19,55–59]. There are five stratigraphic units with the basal layer (5) being archaeologically sterile (see Fig. 1 in Paunero[58]).

Bayesian modelling estimates the start of SU 4 at 13635-12760 cal BP and SU 3c (inferior) at 12750-12020 cal BP, with no outliers (Supplementary Figure 34). There is a gap of up to 950 years between SU 4 and 3c, although more chronometric data is required to test this since there is a limited number of ages overall. Following SU 4 and 3c, cultural activity is evidenced in SU 3a (superior), which is estimated to have started at 8110-5775 cal BP (68.3% CI).

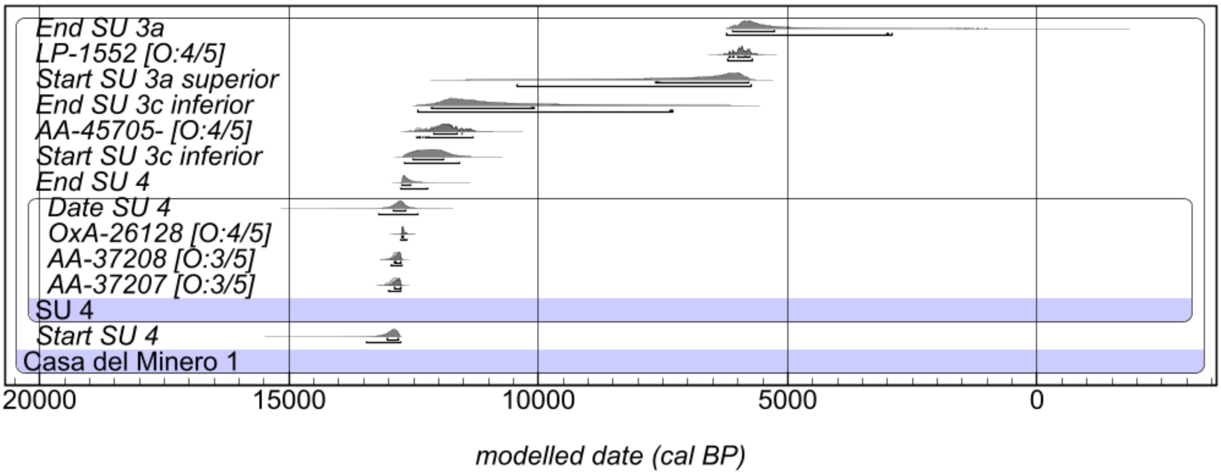


**Supplementary Figure 34.** Bayesian age model for Casa del Minero 1. Brackets beneath each age estimate show 68.3% and 95.4% CI. Outlier analysis output is noted as ‘O:posterior probability/prior probability’.

```
Plot()
{
 Curve("SHCal20","shcal20.14c");
 Outlier_Model("General",T(5),U(0,4),"t");
 Sequence("Casa del Minero 1")
 {
  Boundary("Start SU 4");
  Phase("SU 4")
  {
   R_Date("AA-37207",10999,55)
   {
    Outlier("General", 0.05);
    SU="4";
   };
   R_Date("AA-37208",10967,55)
   {
    Outlier("General", 0.05);
    SU="4";
   };
   R_Date("OxA-26128",10750,45)
   {
    Outlier("General", 0.05);
    SU="4";
   };
   Interval("Duration SU 4");
   Date("Date SU 4");
  };
  Boundary("End SU 4");
  Interval("Duration SU4-3c gap");
  Boundary("Start SU 3c inferior");
  R_Date("AA-45705-",10250,110)
  {
   Outlier("General", 0.05);
   SU="3c inferior";
  };
  Boundary("End SU 3c inferior");
  Boundary("Start SU 3a superior");
  R_Date("LP-1552",5190,80)
  {
   Outlier("General", 0.05);
   SU="3a superior";
  };
  Boundary("End SU 3a");
 };
 Difference("Difference SU 4 and 3c","Start SU 4","Start SU 3c inferior");
 Sequence()
 {
  Boundary("=Start SU 3c inferior");
  Interval("Duration 3c inferior");
  Date("Date 3c inferior");
  Boundary("=End SU 3c inferior");
 };
};
```

## 2.1.5. Cerro La China (1-3)

Cerro La China, including rockshelter and open-air sites 1-3, is an archaeological locality found in Argentina (approximately -37.95, -58.62), containing lithic technology (Fishtail and other uniface/biface)[60–62]. According to Flegenheimer[62], the basal/lowest component of site 3 corresponds to, archaeologically, levels 2 from sites 1-2. Although there is only uniface/biface technology in the former, the latter include Fishtail points. Authors argue that inhabitants are likely from one group engaging in different activities.

A single-phase Bayesian model estimates the start of occupation at 13135-12700 cal BP (see Supplementary Figure 35 and OxCal code).

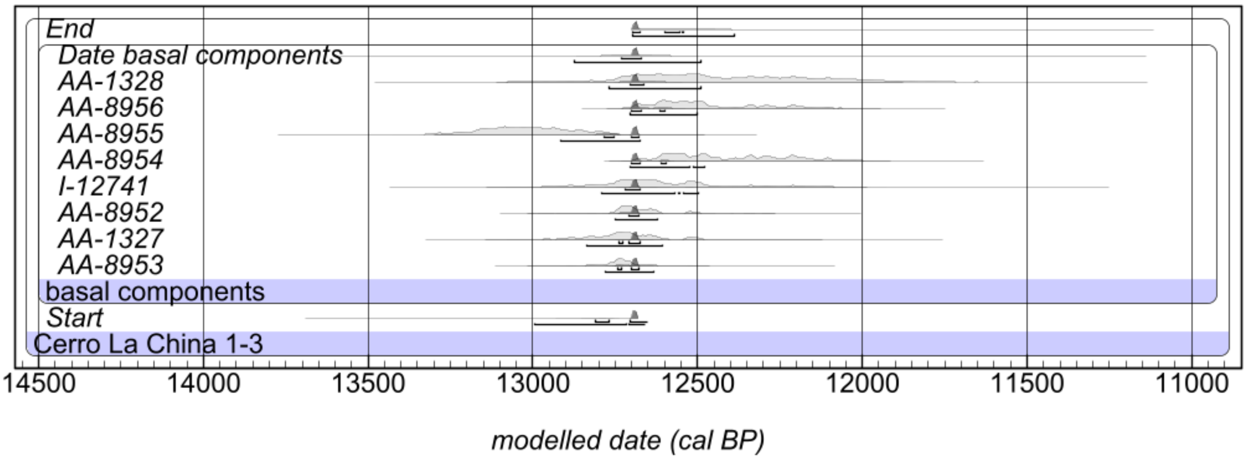


**Supplementary Figure 35.** Bayesian age model for Cerro La China (1-3). Brackets beneath each age estimate show 68.3% and 95.4% CI.

```
Plot()
{
 Curve("SHCal20","shcal20.14c");
 Sequence("Cerro La China 1-3")
 {
  Boundary("Start");
  Phase("basal components")
  {
   R_Date("AA-8953",10804,75)
   {
    site="Cerro La China I";
   };
   R_Date("AA-1327",10790,120)
   {
    site="Cerro La China I";
   };
   R_Date("AA-8952",10745,75)
   {
    site="Cerro La China I";
   };
   R_Date("I-12741",10720,150)
   {
    site="Cerro La China I";
   };
   R_Date("AA-8954",10525,75)
   {
    site="Cerro La China I";
   };
   R_Date("AA-8955",11150,135)
   {
    site="Cerro La China II";
   };
   R_Date("AA-8956",10560,75)
   {
    site="Cerro La China II";
   };
   R_Date("AA-1328",10610,180)
   {
    site="Cerro La China III";
   };
   Date("Date basal components");
   Interval("Duration basal components");
  };
  Boundary("End");
 };
};
```

2.1.6. Cerro Tres Tetas (C3T)

Cerro Tres Tetas (C3T) is an open-air site located in Argentina (-48.149444, -68.933333), containing lithic technology, hearths, and associated *Lama guanicoe* remains (including cut marks)[45,63–70]. There are a number of stratigraphic units as defined by sedimentological and cultural evidence, with unit 5 containing the earliest cultural occupation.

Bayesian modelling estimates the start of layer 5 (lower) at 13500-12815 cal BP and layer 5 (upper) at 12920-12305 cal BP (see Supplementary Figure 36 and OxCal code). Within layer 5, LP-525 is identified as a likely underestimate with a 100% outlier posterior probability. This is likely due to incomplete decontamination since it is a younger replicate measurement. The averaged age, however, is not an outlier. Following layer 5, cultural activity is evidenced at layer 4, which is estimated to have started at 6285-2300 cal BP (although likely overestimated due to poor chronological resolution).

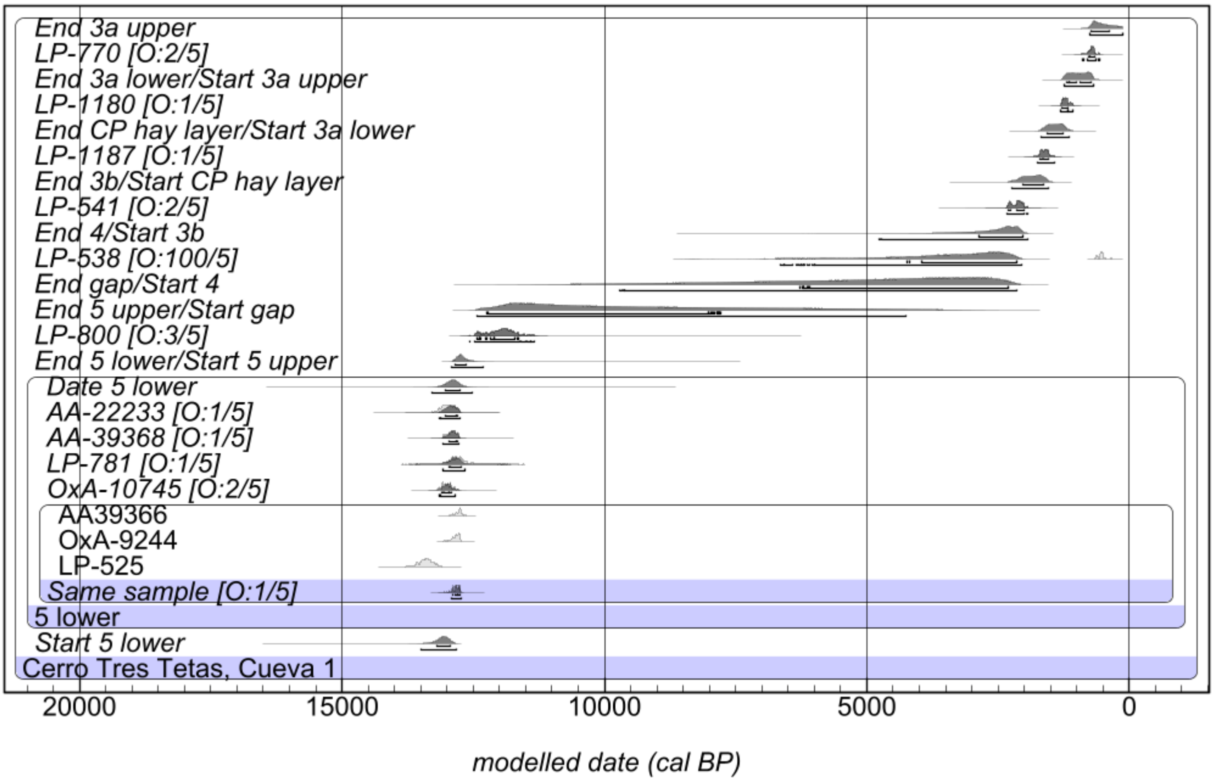


**Supplementary Figure 36.** Bayesian age model for Cerro Tres Tetas. Brackets beneath each age estimate show 68.3% and 95.4% CI. Outlier analysis output is noted as ‘O:posterior probability/prior probability’.

```
Plot()
{
 Curve("SHCal20","shcal20.14c");
 Outlier_Model("SSimple",N(0,2),0,"s");
 Outlier_Model("General",T(5),U(0,4),"t");
 Sequence("Cerro Tres Tetas, Cueva 1")
 {
  Boundary("Start 5 lower");
  Phase("5 lower")
  {
   R_Combine("Same sample")
   {
    R_Date("LP-525",11560,140)
    {
     Outlier("SSimple",0.05);
     context="5 lower";
    };
    R_Date("OxA-9244",10915,65)
    {
     Outlier("SSimple",0.05);
     context="5 lower";
    };
    R_Date("AA39366",10853,70)
    {
     Outlier("SSimple",0.05);
     context="5 lower";
    };
    Outlier("General",0.05);
   };
   R_Date("OxA-10745",11145,60)
   {
    Outlier("General",0.05);
    context="5 lower";
   };
   R_Date("LP-781",10850,150)
   {
    Outlier("General",0.05);
    context="5 lower";
   };
   R_Date("AA-39368",11015,66)
   {
    Outlier("General",0.05);
    context="5 lower";
   };
   R_Date("AA-22233",11100,150)
   {
    Outlier("General",0.05);
    context="5 lower";
   };
   Interval("Duration 5 lower");
   Date("Date 5 lower");
  };
  Boundary("End 5 lower/Start 5 upper");
  R_Date("LP-800",10260,110)
  {
   Outlier("General",0.05);
   context="5 upper";
  };
  Boundary("End 5 upper/Start gap");
  Boundary("End gap/Start 4");
  R_Date("LP-538",552,70)
  {
   Outlier("General",0.05);
   context="4";
  };
  Boundary("End 4/Start 3b");
  R_Date("LP-541",2190,70)
  {
   Outlier("General",0.05);
   context="3b";
  };
  Boundary("End 3b/Start CP hay layer");
  R_Date("LP-1187",1740,60)
  {
   Outlier("General",0.05);
   context="CP hay layer (between E and 3a lower)";
  };
  Boundary("End CP hay layer/Start 3a lower");
  R_Date("LP-1180",1340,50)
  {
   Outlier("General",0.05);
   context="3a lower";
  };
  Boundary("End 3a lower/Start 3a upper");
  R_Date("LP-770",830,60)
  {
   Outlier("General",0.05);
   context="3a upper";
  };
  Boundary("End 3a upper");
 };
 Sequence()
 {
  Boundary("=Start 5 lower)");
  Interval("Duration of 5 (lower and upper)");
  Date("Date of 5 (lower and upper)");
  Boundary("=End 5 upper/Start gap");
 };
};
```

## 2.1.7. El Alto 3

El Alto 3 is a rockshelter located in Argentina (-31.4, -64.7), containing lithic technology[71,72]. There are five sedimentary units (SU), with the earliest archaeological evidence found in units 4 and 3 (see Fig. 1 in Rivero & Roldán[71]). The latter, for which no radiocarbon measurements have been produced, includes ten lanceolate projectile points.

Bayesian modelling estimates the start of SU4 and 3 at 18095-12745 cal BP and 11260-4910 cal BP (if taken to be the end of SU4), respectively, with no outliers (see Supplementary Figure 37 and OxCal code). The former estimate is likely overestimated due to poor chronological resolution at the site.

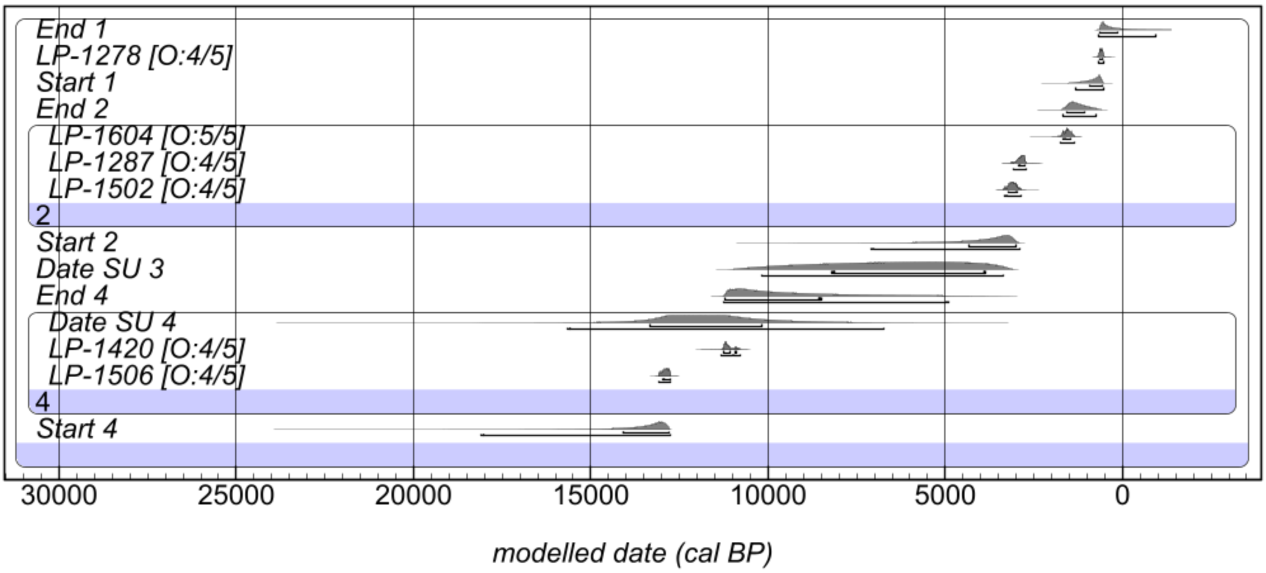


**Supplementary Figure 37.** Bayesian age model for El Alto 3. Brackets beneath each age estimate show 68.3% and 95.4% CI. Outlier analysis output is noted as ‘O:posterior probability/prior probability’.

```
Plot()
{
 Outlier_Model("General",T(5),U(0,4),"t");
 Curve("SHCal20","shcal20.14c");
 Sequence()
 {
  Boundary("Start 4");
  Phase("4")
  {
   R_Date("LP-1506",11010,80)
   {
    Outlier("General", 0.05);
   };
   R_Date("LP-1420",9790,80)
   {
    Outlier("General", 0.05);
   };
   Date("Date SU 4")
   {
    Outlier("General", 0.05);
   };
   Interval("Duration SU 4");
  };
  Boundary("End 4");
  Date("Date SU 3");
  Interval("Duration SU 3");
  Boundary("Start 2");
  Phase("2")
  {
   R_Date("LP-1502",2990,70)
   {
    Outlier("General", 0.05);
   };
   R_Date("LP-1287",2770,80)
   {
    Outlier("General", 0.05);
   };
   R_Date("LP-1604",1690,70)
   {
    Outlier("General", 0.05);
   };
  };
  Boundary("End 2");
  Boundary("Start 1");
  R_Date("LP-1278",670,50)
  {
   Outlier("General", 0.05);
  };
  Boundary("End 1");
 };
};
```

## 2.1.8. Gruta del Indio

Gruta del Indio is a cave site located in Argentina (-31.4, -64.7), containing lithic technology and extinct fauna (*Mylodon* and *Megatherium*)[73–76]. There are four cultural components, with Atuel IV being the oldest. Although there are extinct fauna remains within this level, it is unclear whether humans interacted with the animals[73,75]. Therefore, although there are direct radiocarbon measurements on the fauna, Gil[73] argues that the antiquity of human activity at Gruta del Indio might only be inferred from the charcoal ages.

Bayesian modelling estimates the start of Atuel IV (using only charcoal ages) at 12800-11695 cal BP, with one outlier (GrN-5394; charcoal in Atuel IV; see Supplementary Figure 38 and OxCal code). This is likely an age underestimate due to vertical mixing or pretreatment issues. Following Atuel IV, cultural activity is evidenced at Pre Atuel III, which is estimated to have started at 9970-8435 cal BP.

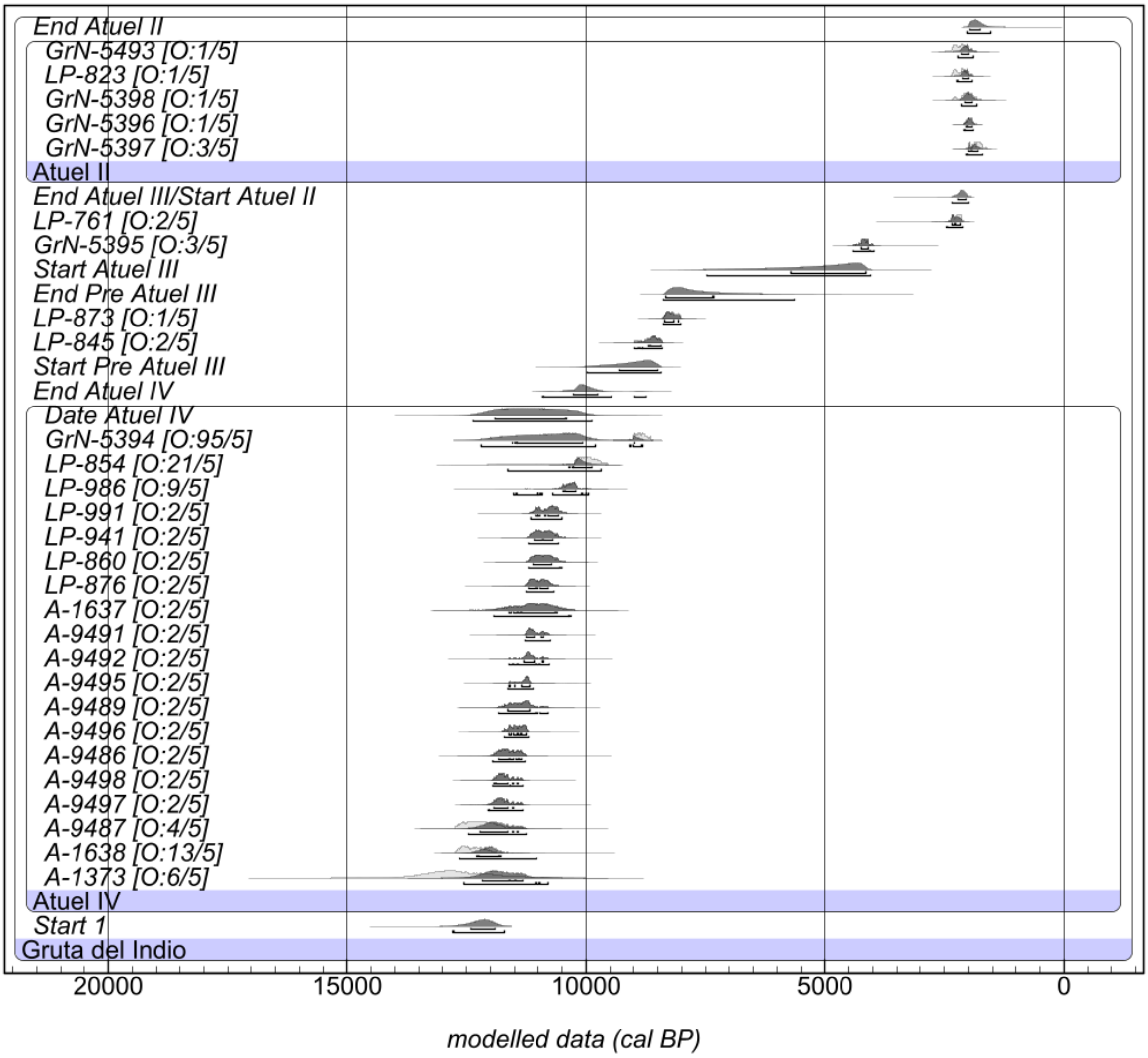


**Supplementary Figure 38.** Bayesian age model for Gruta del Indio. Brackets beneath each age estimate show 68.3% and 95.4% CI. Outlier analysis output is noted as ‘O:posterior probability/prior probability’.

```
Plot()
{
 Outlier_Model("General",T(5),U(0,4),"t");
 Curve("SHCal20","shcal20.14c");
 Sequence("Gruta del Indio")
 {
  Boundary("Start 1");
  Phase("Atuel IV")
  {
   R_Date("A-1373",10930,540)
   {
    Outlier("General", 0.05);
    material="charcoal";
   };
   R_Date("A-1638",10530,140)
   {
    Outlier("General", 0.05);
    material="charcoal";
   };
   R_Date("A-9487",10440,222)
   {
    Outlier("General", 0.05);
    material="charcoal";
   };
   R_Date("A-9497",10195,80)
   {
    Outlier("General", 0.05);
    material="charcoal";
   };
   R_Date("A-9498",10170,70)
   {
    Outlier("General", 0.05);
    material="charcoal";
   };
   R_Date("A-9486",10135,95)
   {
    Outlier("General", 0.05);
    material="charcoal";
   };
   R_Date("A-9496",9990,75)
   {
    Outlier("General", 0.05);
    material="charcoal";
   };
   R_Date("A-9489",9905,140)
   {
    Outlier("General", 0.05);
    material="charcoal";
   };
   R_Date("A-9495",9890,75)
   {
    Outlier("General", 0.05);
    material="charcoal";
   };
   R_Date("A-9492",9825,95)
   {
    Outlier("General", 0.05);
    material="charcoal";
   };
   R_Date("A-9491",9770,85)
   {
    Outlier("General", 0.05);
    material="charcoal";
   };
   R_Date("A-1637",9740,280)
   {
    Outlier("General", 0.05);
    material="charcoal";
   };
   R_Date("LP-876",9700,110)
   {
    Outlier("General", 0.05);
    material="charcoal";
   };
   R_Date("LP-860",9590,120)
   {
    Outlier("General", 0.05);
    material="charcoal";
   };
   R_Date("LP-941",9580,105)
   {
    Outlier("General", 0.05);
    material="charcoal";
   };
   R_Date("LP-991",9510,90)
   {
    Outlier("General", 0.05);
    material="charcoal";
   };
   R_Date("LP-986",9160,90)
   {
    Outlier("General", 0.05);
    material="charcoal";
   };
   R_Date("LP-854",8920,110)
   {
    Outlier("General", 0.05);
    material="charcoal";
   };
   R_Date("GrN-5394",8045,55)
   {
    Outlier("General", 0.05);
    material="charcoal";
   };
   Date("Date Atuel IV");
   Interval("Duration Atuel IV");
  };
  Boundary("End Atuel IV");
  Boundary("Start Pre Atuel III");
  R_Date("LP-845",7860,90)
  {
   Outlier("General", 0.05);
   context="Pre Atuel III";
  };
  R_Date("LP-873",7430,90)
  {
   Outlier("General", 0.05);
   context="Pre Atuel III";
  };
  Boundary("End Pre Atuel III");
  Boundary("Start Atuel III");
  R_Date("GrN-5395",3830,40)
  {
   Outlier("General", 0.05);
   context="Atuel III";
  };
  R_Date("LP-761",2300,60)
  {
   Outlier("General", 0.05);
   context="Atuel III";
  };
  Boundary("End Atuel III/Start Atuel II");
  Phase("Atuel II")
  {
   R_Date("GrN-5397",1910,60)
   {
    Outlier("General", 0.05);
    context="Atuel II";
   };
   R_Date("GrN-5396",2065,40)
   {
    Outlier("General", 0.05);
    context="Atuel II";
   };
   R_Date("GrN-5398",2095,95)
   {
    Outlier("General", 0.05);
    context="Atuel II";
   };
   R_Date("LP-823",2200,70)
   {
    Outlier("General", 0.05);
    context="Atuel II";
   };
   R_Date("GrN-5493",2210,90)
   {
    Outlier("General", 0.05);
    context="Atuel II";
   };
  };
  Boundary("End Atuel II");
 };
};
```

## 2.1.9. Paso Otero 5

Paso Otero 5 is an open-air site part of the Otero archaeological locality found in Argentina (-38.2022, -59.1327), containing lithic technology (Fishtail) and extinct fauna (*Megatherium americanum*, *Macrauchenia* cf. *patachonica* and *Equus neogeus*)[77–80]. There are a number of stratigraphic units as defined by sedimentological evidence, with the archaeological component mostly located within the Puesto Callejón Viejo Paleosol (including layers ACb6 and Ab6; see Fig. 2 in Martinez & Gutierrez[79]). There are currently six radiocarbon measurements on bone, with the three younger ages considered anomalous by Martinez and Guitiérrez[79]. As such, a single-phase model was built using the three remaining bone dates and one soil measurement.

A single-phase Bayesian model estimates the start of occupation at 14780-11845 cal BP (see Supplementary Figure 39 and OxCal code).

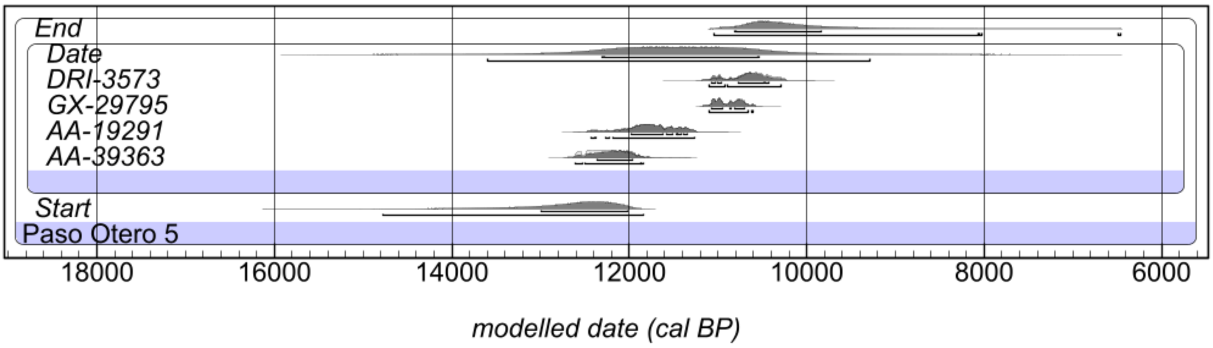


**Supplementary Figure 39.** Bayesian age model for Paso Otero 5. Brackets beneath each age estimate show 68.3% and 95.4% CI.

```
Plot()
{
 Curve("SHCal20","shcal20.14c");
 Sequence("Paso Otero 5")
 {
  Boundary("Start");
  Phase("")
  {
   R_Date("AA-39363",10440,100);
   R_Date("AA-19291",10190,120);
   R_Date("GX-29795",9560,50);
   R_Date("DRI-3573",9399,116);
   Date("Date");
   Interval("Duration");
  };
  Boundary("End");
 };
};
```

2.1.9. Piedra Museo (AEP-1)

Within the Piedra Museo Locality, Alero El Puesto 1 (AEP-1) is a rockshelter site located in Argentina (-47.895000, -67.867778), containing lithic technology and extinct faunal remains (*Hippidion saldiasi* and *Mylodon* sp.)[45,81–86]. There are six stratigraphic units (SUs) at the site, which are based on sedimentological differences (see Fig. 1 in [87]). The lower levels, 5 and 6, have been affected by water table fluctuations. There are two cultural components separated by a chronological hiatus (SU3). The Lower Component is composed of SUs 6-4, whilst the Upper Component includes SUs 2-1.

Bayesian modelling of AEP-1 estimates the start of SU6 at 15850-12515 cal BP, SU5 at 12550-12060 cal BP (Fishtail projectile point at SU4/5, in association with AA-8428 in SU5), and SU4 at 12375-11025 cal BP (see Supplementary Figure 40 and OxCal code). SU6 and the SU5/6 transition contain all outliers identified (AA-20125, charcoal; OxA-9509, charcoal; OxA-8528, *Hippidion* bone; AA-39362, *Hippidion* bone; OxA-9508, charcoal). The *Hippidion* bone samples—replicates that fail a $\chi^2$ test and, as such, are significantly different ($\chi^2$ = 66.031; df = 2; p = 6.0)—likely denote pretreatment issues, with the oldest date probably being more reliable. The rest are likely under- *and* over-estimates, suggesting vertical mixing, inbuilt age for charcoal, and/or pretreatment issues. SUs 6-4 are estimated to have lasted up to 4,405 years. Following these strata, cultural activity is evidenced at the Upper Component, which is estimated to have started at 9705-8200 cal BP.

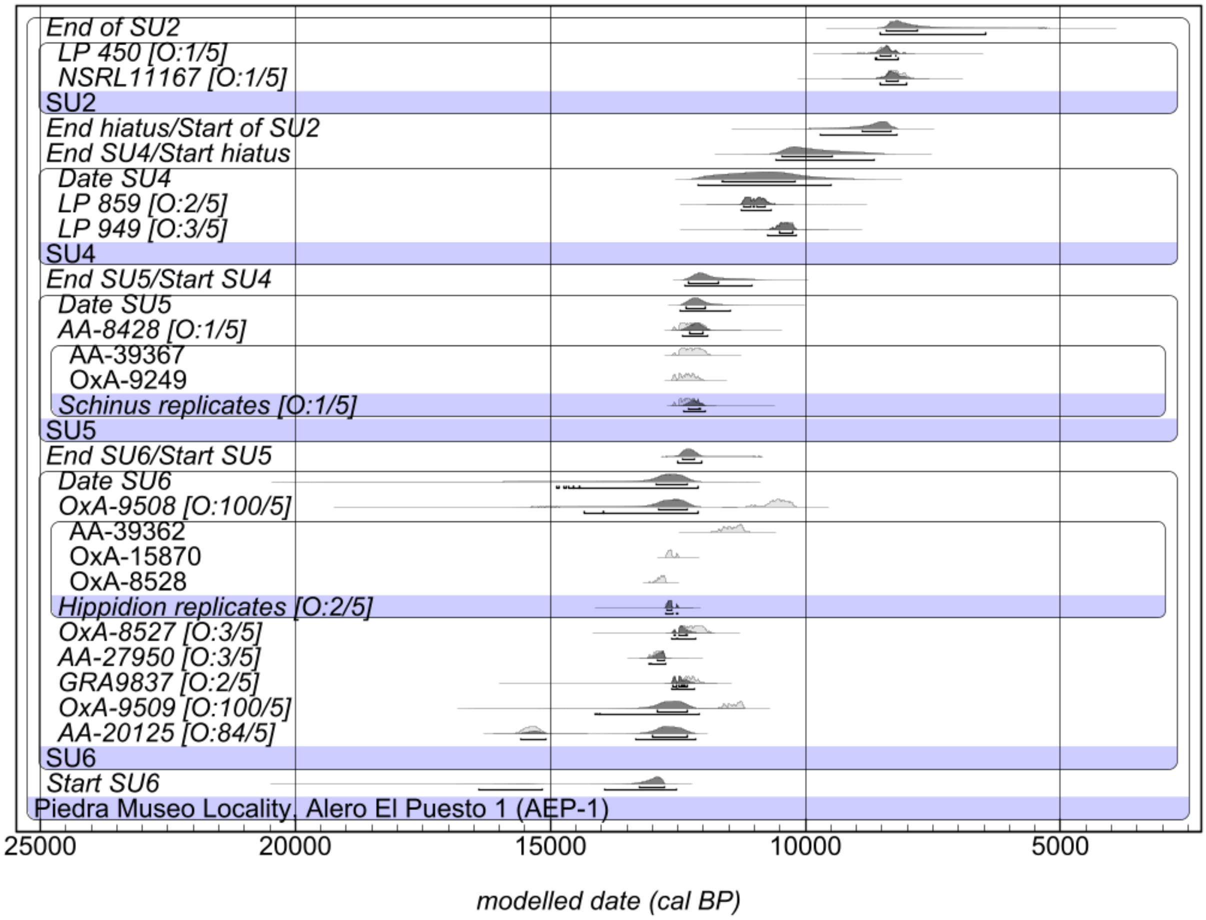


**Supplementary Figure 40.** Bayesian age model for AEP-1. Brackets beneath each age estimate show 68.3% and 95.4% CI. Outlier analysis output is noted as ‘O:posterior probability/prior probability’.

```
Plot()
{
 Curve("SHCal20","shcal20.14c");
 Outlier_Model("SSimple",N(0,2),0,"s");
 Outlier_Model("General",T(5),U(0,4),"t");
 Sequence("Piedra Museo Locality, Alero El Puesto 1 (AEP-1)")
 {
  Boundary("Start SU6");
  Phase("SU6")
  {
   R_Date("AA-20125",12890,90)
   {
    Outlier("General", 0.05);
    context="stratigraphic unit 6 bottom";
    material="charcoal";
   };
   R_Date("OxA-9509",9950,75)
   {
    Outlier("General", 0.05);
    context="5/6 transition";
    material="charcoal";
   };
   R_Date("GRA9837",10470,60)
   {
    Outlier("General", 0.05);
    context="stratigraphic unit 6 bottom";
    material="charcoal";
   };
   R_Date("AA-27950",11000,65)
   {
    Outlier("General", 0.05);
    context="stratigraphic unit 6 bottom";
    material="charcoal";
   };
   R_Date("OxA-8527",10390,70)
   {
    Outlier("General", 0.05);
    context="stratigraphic unit 6 middle";
    material="bone";
   };
   R_Combine("Hippidion replicates")
   {
    //Hippidion replicate
    R_Date("OxA-8528",10925,65)
    {
     Outlier("SSimple", 0.05);
     context="stratigraphic unit 6 bottom";
     material="bone";
    };
    //Hippidion replicate
    R_Date("OxA-15870",10675,55)
    {
     Outlier("SSimple", 0.05);
     context="stratigraphic unit 6 bottom";
     material="bone";
    };
    //Hippidion replicate
    R_Date("AA-39362",9952,97)
    {
     Outlier("SSimple", 0.05);
     context="stratigraphic unit 6 bottom";
     material="bone";
    };
    Outlier("General", 0.05);
   };
   R_Date("OxA-9508",9350,130)
   {
    Outlier("General", 0.05);
    context="5/6 transition";
    material="charcoal";
   };
   Interval("Duration SU6");
   Date("Date SU6");
  };
  Boundary("End SU6/Start SU5");
  Phase("SU5")
  {
   R_Combine("Schinus replicates")
   {
    //Schinus replicate
    R_Date("OxA-9249",10470,65)
    {
     Outlier("SSimple", 0.05);
     context="stratigraphic unit 5 bottom";
     material="charcoal";
    };
    //Schinus replicate
    R_Date("AA-39367",10400,79)
    {
     Outlier("SSimple", 0.05);
     context="stratigraphic unit 5 bottom";
     material="charcoal";
    };
    Outlier("General", 0.05);
   };
   R_Date("AA-8428",10400,80)
   {
    Outlier("General", 0.05);
    context="stratigraphic unit 5 middle";
    material="bone";
   };
   Interval("Duration SU5");
   Date("Date SU5");
  };
  Boundary("End SU5/Start SU4");
  Phase("SU4")
  {
   R_Date("LP 949",9230,105)
   {
    Outlier("General", 0.05);
    context="stratigraphic unit 4 top";
    material="bone";
   };
   R_Date("LP 859",9710,105)
   {
    Outlier("General", 0.05);
    context="stratigraphic unit 4 bottom";
    material="bone";
   };
   Interval("Duration SU4");
   Date("Date SU4");
  };
  Boundary("End SU4/Start hiatus");
  Interval("Duration hiatus");
  Boundary("End hiatus/Start of SU2");
  Phase("SU2")
  {
   R_Date("NSRL11167",7470,140)
   {
    Outlier("General", 0.05);
    context="stratigraphic unit 2 middle";
    material="charcoal";
   };
   R_Date("LP 450",7670,110)
   {
    Outlier("General", 0.05);
    context="stratigraphic unit 2 bottom";
    material="bone";
   };
   Interval("Duration SU2");
  };
  Boundary("End of SU2");
 };
 Difference("Difference SU6 and 5","Start SU6","End SU6/Start SU5");
 Difference("Difference SU6 and 4","Start SU6","End SU5/Start SU4");
 Sequence()
 {
  Boundary("=Start SU6");
  Interval("Duration SU6-4");
  Date("Date SU6-4");
  Boundary("=End SU4/Start hiatus");
 };
};
```

### 2.1.10. La Moderna

La Moderna is an open-air site located in Argentina  (-37.13, -60.08), containing lithic technology in association with a *Doedicurus clavicaudatus*[88,89]. There are four geological levels, with the archaeological material found in transitional unit LU(a'), between the Guerrero and Río Salado members (see Fig. 1 in [88]). Although there is a bone collagen age at 12350 ± 370 BP (TO-1507), this is considered to be anomalous and the chronology has been so far set at 7500 BP. This site was not included in the Bayesian analysis.

## *2.2. Brazil*

### 2.2.1. Abrigo do Sol

Abrigo do Sol is a rockshelter site located in Brazil (-14.2, -59.9), containing lithic technology[90,91]. Initial excavations were carried out in the 1970's, with reports showing archeo-chronometric inconsistencies. Although recent efforts aimed to elucidate these through a review of published and unpublished data, there is insufficient stratigraphic clarity (and relationship) for Bayesian modelling. As such, this site was not included in the Bayesian analysis.

2.2.2. Boqueirão do Sítio da Pedra Furada

Boqueirão do Sítio da Pedra Furada is a rockshelter site located in Brazil (-8.85, -42.550556), containing lithic technology (including Itaparica), stone structures (intentional conglomerations) and hearths[92–104]. There are two principal components: Holocene and Upper Pleistocene. The latter has been labelled the Pedra Furada phase and there is controversy regarding its archaeological nature[93]. The main phases are further divided into different layers: PF 1-3 within the Pedra Furada phase, and Serra Talhada (ST) 1, ST 2 and Agreste (AG) for the Holocene component (see Fig. 15 in Parenti[103]). These layers are not defined by sedimentology, but rather by cultural characteristics and chronology.

Bayesian modelling including most finite radiocarbon dates for the site estimates the end of the Pleistocene component (PF 3) at 17830-12910 cal BP (with an estimated age range at 27438-15580 cal BP), and the start of the Holocene component (ST 1) at 13755-11465 cal BP, with a gap of between 300 and 5,610 years in between (see Supplementary Figure 41 and OxCal code). There are no outliers. Fourteen dates were not included in the model as no phase, i.e., PF 1 etc, was noted. Following ST1, cultural activity is evidenced at ST2, which is estimated to have started at 9180-8425 cal BP.

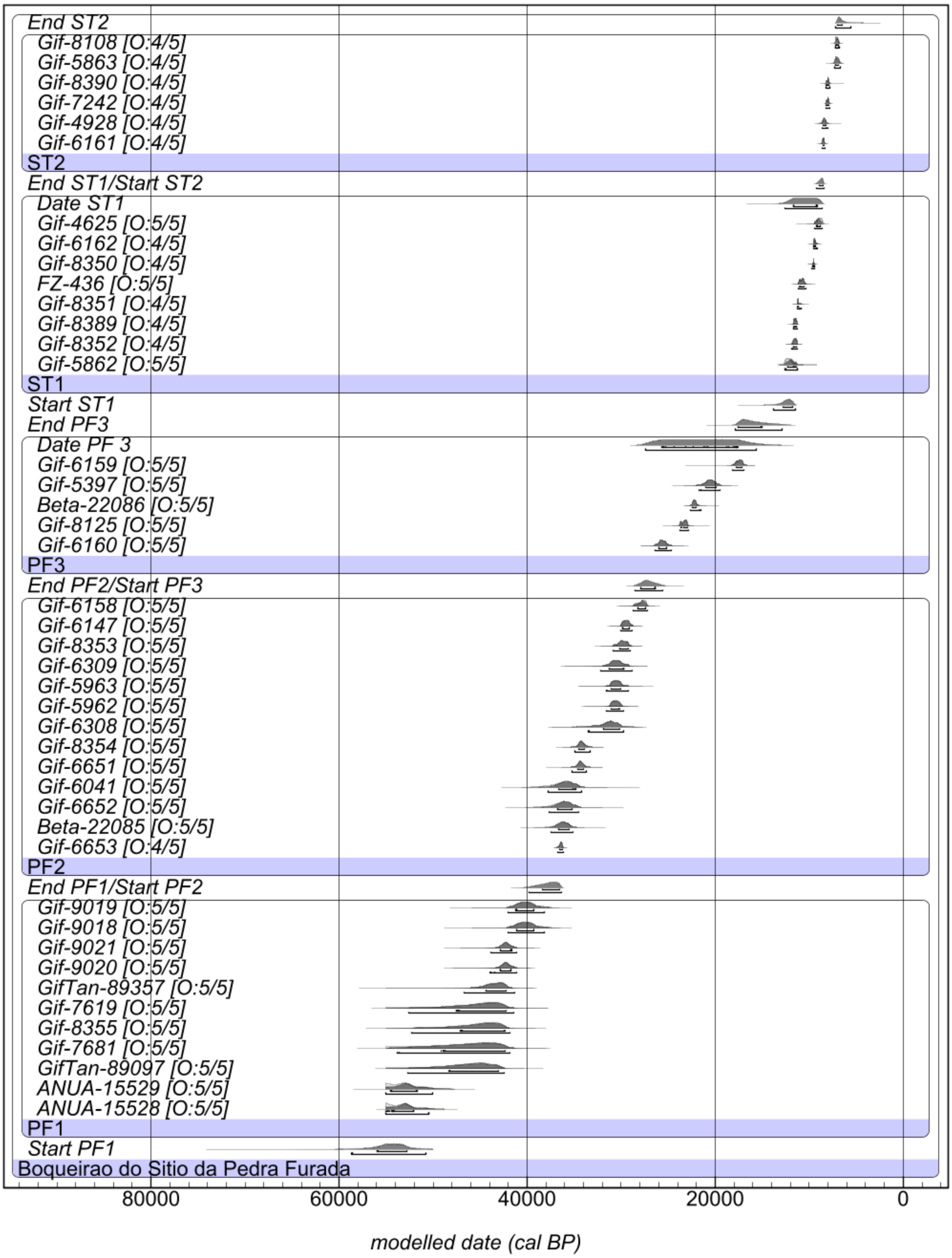


**Supplementary Figure 41.** Bayesian age model for Boqueirão do Sitio da Pedra Furada. Brackets beneath each age estimate show 68.3% and 95.4% CI. Outlier analysis output is noted as 'O:posterior probability/prior probability'.

```
Plot()
{
 Curve("SHCal20","shcal20.14c");
 Outlier_Model("General",T(5),U(0,4),"t");
 Sequence("Boqueirao do Sitio da Pedra Furada")
 {
  Boundary("Start PF1");
  Phase("PF1")
  {
   R_Date("ANUA-15528",55575,4078)
   {
    Outlier("General", 0.05);
    stage="PF1";
   };
   R_Date("ANUA-15529",53120,3127)
   {
    Outlier("General", 0.05);
    stage="PF1";
   };
   R_Date("GifTan-89097",42400,2600)
   {
    Outlier("General", 0.05);
    stage="PF1";
   };
   R_Date("Gif-7681",41500,3527)
   {
    Outlier("General", 0.05);
    stage="PF1";
   };
   R_Date("Gif-8355",41000,2517)
   {
    Outlier("General", 0.05);
    stage="PF1";
   };
   R_Date("Gif-7619",40800,2740)
   {
    Outlier("General", 0.05);
    stage="PF1";
   };
   R_Date("GifTan-89357",39500,1600)
   {
    Outlier("General", 0.05);
    stage="PF1";
   };
   R_Date("Gif-9020",38000,1000)
   {
    Outlier("General", 0.05);
    stage="PF1";
   };
   R_Date("Gif-9021",38000,1000)
   {
    Outlier("General", 0.05);
    stage="PF1";
   };
   R_Date("Gif-9018",35000,1000)
   {
    Outlier("General", 0.05);
    stage="PF1";
   };
   R_Date("Gif-9019",35000,1000)
   {
    Outlier("General", 0.05);
    stage="PF1";
   };
  };
  Boundary("End PF1/Start PF2");
  Phase("PF2")
  {
   R_Date("Gif-6653",32160,100)
   {
    Outlier("General", 0.05);
    stage="PF2";
   };
   R_Date("Beta-22085",31860,560)
   {
    Outlier("General", 0.05);
    stage="PF2";
   };
   R_Date("Gif-6652",31700,830)
   {
    Outlier("General", 0.05);
    stage="PF2";
   };
   R_Date("Gif-6041",31500,950)
   {
    Outlier("General", 0.05);
    stage="PF2";
   };
   R_Date("Gif-6651",29860,350)
   {
    Outlier("General", 0.05);
    stage="PF2";
   };
   R_Date("Gif-8354",29740,350)
   {
    Outlier("General", 0.05);
    stage="PF2";
   };
   R_Date("Gif-6308",27000,800)
   {
    Outlier("General", 0.05);
    stage="PF2";
   };
   R_Date("Gif-5962",26400,500)
   {
    Outlier("General", 0.05);
    stage="PF2";
   };
   R_Date("Gif-5963",26300,600)
   {
    Outlier("General", 0.05);
    stage="PF2";
   };
   R_Date("Gif-6309",26300,800)
   {
    Outlier("General", 0.05);
    stage="PF2";
   };
   R_Date("Gif-8353",25600,450)
   {
    Outlier("General", 0.05);
    stage="PF2";
   };
   R_Date("Gif-6147",25200,320)
   {
    Outlier("General", 0.05);
    stage="PF2";
   };
   R_Date("Gif-6158",23500,390)
   {
    Outlier("General", 0.05);
    stage="PF2";
   };
  };
  Boundary("End PF2/Start PF3");
  Phase("PF3")
  {
   R_Date("Gif-6160",21400,400)
   {
    Outlier("General", 0.05);
    stage="PF3";
   };
   R_Date("Gif-8125",19300,200)
   {
    Outlier("General", 0.05);
    stage="PF3";
   };
   R_Date("Beta-22086",18310,190)
   {
    Outlier("General", 0.05);
    stage="PF3";
   };
   R_Date("Gif-5397",17000,400)
   {
    Outlier("General", 0.05);
    stage="PF3";
   };
   R_Date("Gif-6159",14300,210)
   {
    Outlier("General", 0.05);
    stage="PF3";
   };
   Date("Date PF 3");
  };
  Boundary("End PF3");
  Interval("Duration gap");
  Boundary("Start ST1");
  Phase("ST1")
  {
   R_Date("Gif-5862",10400,180)
   {
    Outlier("General", 0.05);
    stage="ST1";
   };
   R_Date("Gif-8352",10050,80)
   {
    Outlier("General", 0.05);
    stage="ST1";
   };
   R_Date("Gif-8389",10040,40)
   {
    Outlier("General", 0.05);
    stage="ST1";
   };
   R_Date("Gif-8351",9800,60)
   {
    Outlier("General", 0.05);
    stage="ST1";
   };
   R_Date("FZ-436",9506,133)
   {
    Outlier("General", 0.05);
    stage="ST1";
   };
   R_Date("Gif-8350",8600,60)
   {
    Outlier("General", 0.05);
    stage="ST1";
   };
   R_Date("Gif-6162",8450,80)
   {
    Outlier("General", 0.05);
    stage="ST1";
   };
   R_Date("Gif-4625",8050,170)
   {
    Outlier("General", 0.05);
    stage="ST1";
   };
   Date("Date ST1");
  };
  Boundary("End ST1/Start ST2");
  Phase("ST2")
  {
   R_Date("Gif-6161",7750,80)
   {
    Outlier("General", 0.05);
    stage="ST2";
   };
   R_Date("Gif-4928",7640,140)
   {
    Outlier("General", 0.05);
    stage="ST2";
   };
   R_Date("Gif-7242",7230,80)
   {
    Outlier("General", 0.05);
    stage="ST2";
   };
   R_Date("Gif-8390",7220,80)
   {
    Outlier("General", 0.05);
    stage="ST2";
   };
   R_Date("Gif-5863",6160,130)
   {
    Outlier("General", 0.05);
    stage="ST2";
   };
   R_Date("Gif-8108",6150,60)
   {
    Outlier("General", 0.05);
    stage="ST2";
   };
  };
  Boundary("End ST2");
 };
};
```

### 2.2.3. Caverna da Pedra Pintada

Caverna da Pedra Pintada (also known as Gruta do Pilão) is a cave site in Brazil (-1.1, -54.06), containing lithic technology and ceramics[105–110]. The site was excavated in the 1990s under Roosevelt[107] and in 2014 under Pereira[110]. As for Roosevelt's excavation, 20 archaeological strata divided into four main groups—culturally sterile (strata 21-18), Pleistocene/preceramic (strata 17-16), culturally sterile (strata 15-13), and Holocene/ceramic (strata 12-1)—were identified (see Fig. 5 in Roosevelt et al.[107]). Strata 17-16 were subdivided into four main periods: initial, early, middle and late. In the 2014 excavation, ten archaeological layers were defined by sedimentological characteristics and the presence/absence of cultural material (see Fig. 4 in Rodet et al.[109] and Table 2 in Pereira & Moraes[110]). Stratigraphy emerging from both excavations was correlated by Pereira (per Rodet et al.[109]), but no such information was found.

Bayesian modelling of Roosevelt's data estimates the start of the initial period for all units at 13035-12195 cal BP, the early period at 12400-12100 cal BP, and the late period at 11920-11720 cal BP (with only the former two overlapping at 95% CI; Supplementary Figure 42 and OxCal code). There are four major outliers, including two humic fractions (GX19523CAMS and GX19539CAMS; underestimates), one wood charcoal (Beta-76954CAMS; overestimate), and one carbonised seed (GX17413; overestimate). These likely denote modern carbon presence, inbuilt age, and/or vertical mixing. Modelling of Pereira's data estimates the start of level I at 13390-12240 cal BP, with one major outlier in level VIII (Beta-434985, charcoal or charred seeds; underestimate) (Supplementary Figure 43 and OxCal code). The estimates for the start of cultural occupation from both datasets (Roosevelt and Pereira) are comparable (Supplementary Figure 44). Luminescence ages were excluded given that ascertaining the period (i.e., initial, early or late) was not possible. As noted by Roosevelt[107], however, the radiocarbon-based chronology fits well within that derived from these measurements (large error margins).

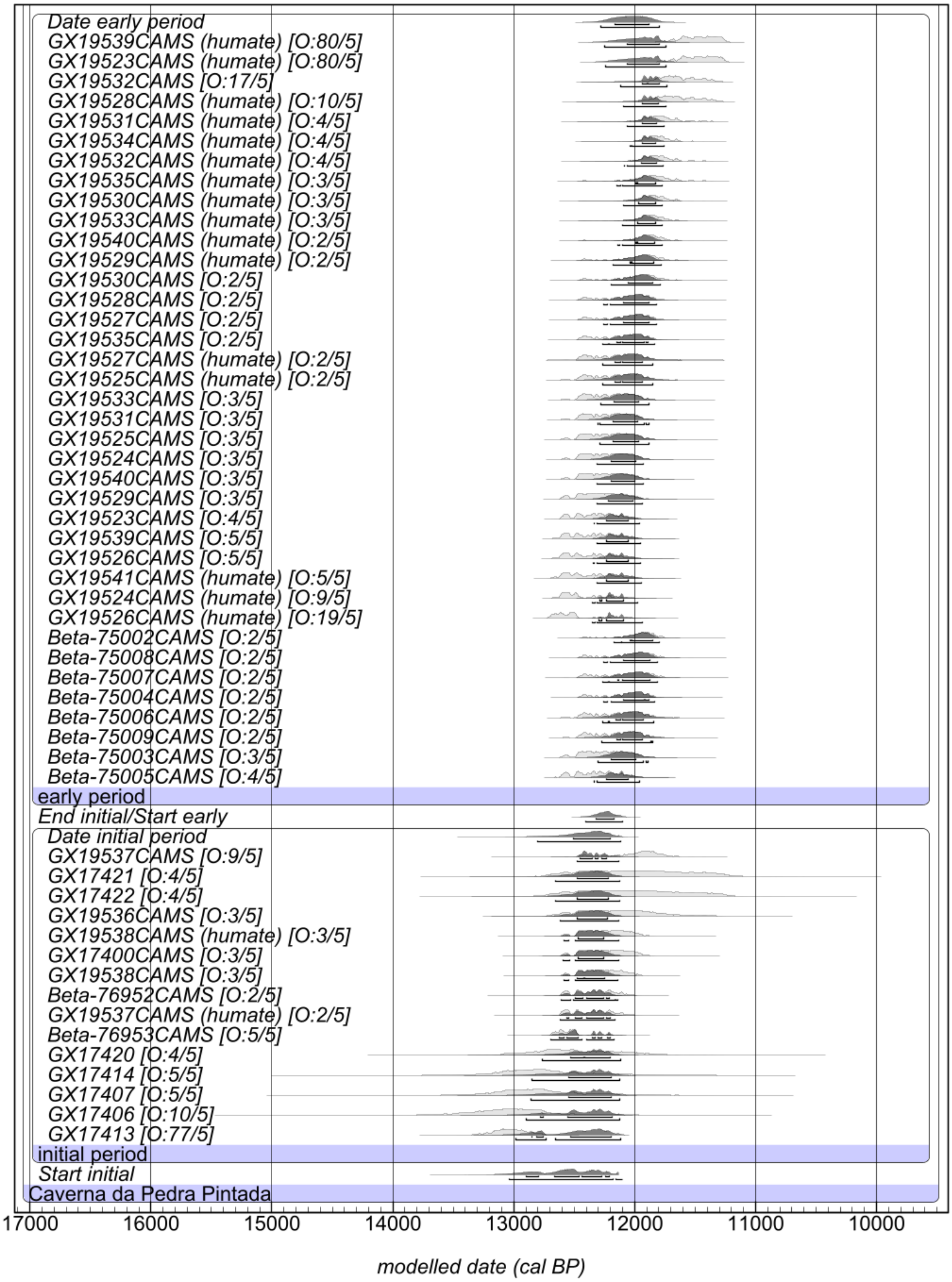


**Supplementary Figure 42.** Bayesian age model for Caverna da Pedra Pintada using Roosevelt's data. Brackets beneath each age estimate show 68.3% and 95.4% CI. Outlier analysis output is noted as 'O:posterior probability/prior probability'.

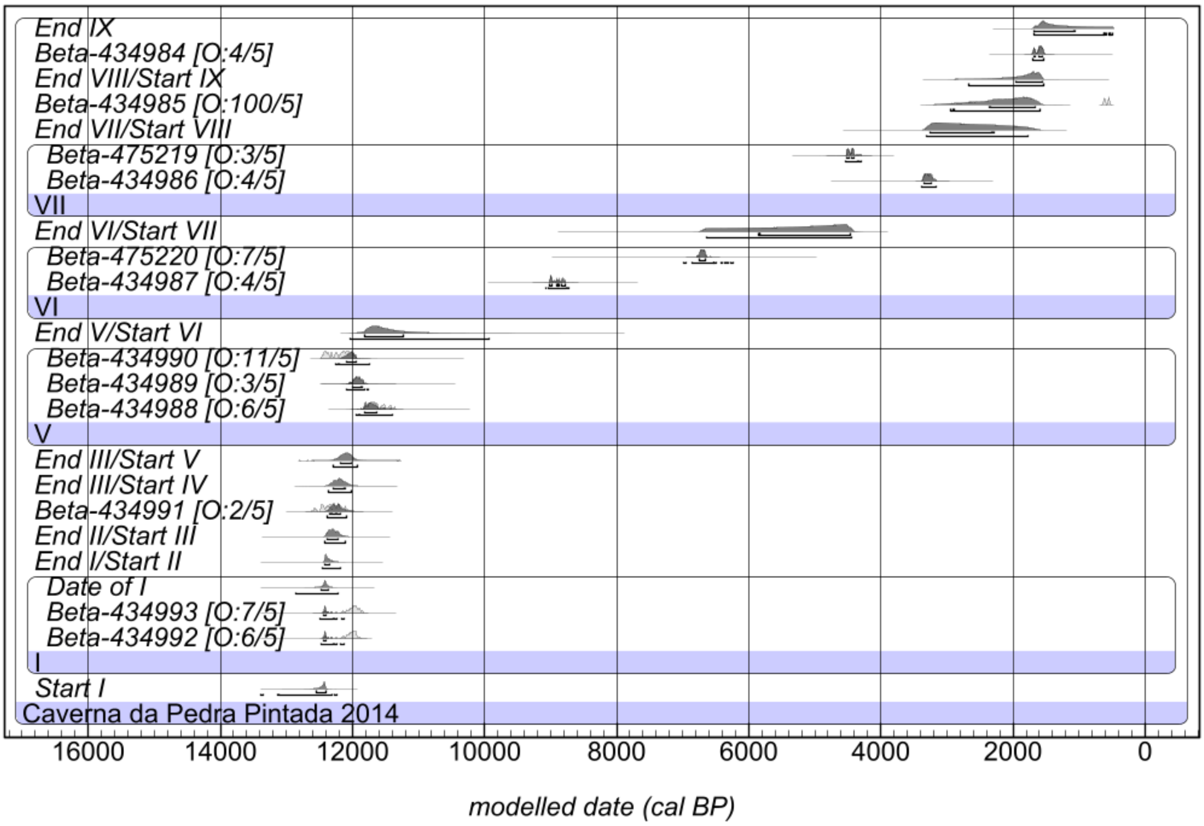


**Supplementary Figure 43.** Bayesian age model for Caverna da Pedra Pintada using Pereira's data. Brackets beneath each age estimate show 68.3% and 95.4% CI. Outlier analysis output is noted as 'O:posterior probability/prior probability'.

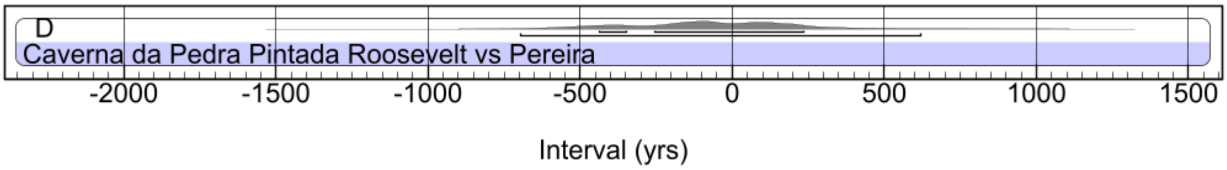


**Supplementary Figure 44.** Probability density function for the 2('D') between the start of cultural activity at Caverna da Pedra Pintada using Roosevelt's (Supplementary Figure 46) vs Pereira's (Supplementary Figure 43) data. These results suggest that there is no significant difference between the modelled outputs, as the distributions include zero at 95.4% CI (black bracket beneath).

```
Plot()
{
 Curve("IntCal20","intcal20.14c");
 Curve("SHCal20","shcal20.14c");
 Mix_Curve("Mixed","IntCal20","SHCal20",U(0,100));
 Outlier_Model("General",T(5),U(0,4),"t");
 Sequence("Caverna da Pedra Pintada - Roosevelt data")
 {
  Boundary("Start initial");
  Phase("initial period")
  {
   R_Date("GX17413",11145,135)
   {
    Outlier("General", 0.05);
    context="unit 5, initial period, prov. no. 8314";
    material="carbonised seeds";
   };
   R_Date("GX17406",11110,310)
   {
    Outlier("General", 0.05);
    context="unit 2, initial period, prov. no. 8231";
    material="carbonised seeds";
   };
   R_Date("GX17407",10905,295)
   {
    Outlier("General", 0.05);
    context="unit 2, initial period, prov. no. 8231";
    material="carbonised seeds";
   };
   R_Date("GX17414",10875,295)
   {
    Outlier("General", 0.05);
    context="unit 5, initial period, prov. no. 8314";
    material="carbonised seeds";
   };
   R_Date("GX17420",10655,285)
   {
    Outlier("General", 0.05);
    context="unit 6, initial period, prov. no. 8345";
    material="carbonised seeds";
   };
   R_Date("Beta-76953CAMS",10560,60)
   {
    Outlier("General", 0.05);
    context="unit 2, initial period, prov. no. 8231";
    material="carbonised seed";
   };
   R_Date("GX19537CAMS (humate)",10470,70)
   {
    Outlier("General", 0.05);
    context="unit 10, initial period, prov. no. 9274";
    material="humate from same";
   };
   R_Date("Beta-76952CAMS",10450,60)
   {
    Outlier("General", 0.05);
    context="unit 2, initial period, prov. no. 8231";
    material="wood charcoal";
   };
   R_Date("GX19538CAMS",10410,60)
   {
    Outlier("General", 0.05);
    context="unit 10, initial period, prov. no. 9274";
    material="carbonised seed";
   };
   R_Date("GX17400CAMS",10392,78)
   {
    Outlier("General", 0.05);
    context="unit 5, initial period, prov. no. 8314";
    material="carbonised seed";
   };
   R_Date("GX19538CAMS (humate)",10390,70)
   {
    Outlier("General", 0.05);
    context="unit 10, initial period, prov. no. 9274";
    material="humate from same";
   };
   R_Date("GX19536CAMS",10350,170)
   {
    Outlier("General", 0.05);
    context="unit 10, initial period, prov. no. 9274";
    material="humate from carb. A. microcarpa seeds";
   };
   R_Date("GX17422",10305,275)
   {
    Outlier("General", 0.05);
    context="unit 6, initial period, prov. no. 8346";
    material="carbonised seeds";
   };
   R_Date("GX17421",10275,275)
   {
    Outlier("General", 0.05);
    context="unit 6, initial period, prov. no. 8345";
    material="carbonised seeds";
   };
   R_Date("GX19537CAMS",10250,70)
   {
    Outlier("General", 0.05);
    context="unit 10, initial period, prov. no. 9274";
    material="carbonised seed";
   };
   Interval("Duration initial period");
   Date("Date initial period");
  };
  Boundary("End initial/Start early");
  Phase("early period")
  {
   R_Date("Beta-75005CAMS",10450,60)
   {
    Outlier("General", 0.05);
    context="unit 7, initial/early period, prov. no. 9147";
    material="carbonised seed";
   };
   R_Date("Beta-75003CAMS",10390,70)
   {
    Outlier("General", 0.05);
    context="unit 7, initial/early period, prov. no. 9147";
    material="carbonised seed";
   };
   R_Date("Beta-75009CAMS",10330,60)
   {
    Outlier("General", 0.05);
    context="unit 7, initial/early period, prov. no. 9148";
    material="carbonised seed";
   };
   R_Date("Beta-75006CAMS",10320,70)
   {
    Outlier("General", 0.05);
    context="unit 7, initial/early period, prov. no. 9147";
    material="carbonised seed";
   };
   R_Date("Beta-75004CAMS",10300,60)
   {
    Outlier("General", 0.05);
    context="unit 7, initial/early period, prov. no. 9147";
    material="wood charcoal";
   };
   R_Date("Beta-75007CAMS",10290,80)
   {
    Outlier("General", 0.05);
    context="unit 7, initial/early period, prov. no. 9147";
    material="wood charcoal";
   };
   R_Date("Beta-75008CAMS",10280,70)
   {
    Outlier("General", 0.05);
    context="unit 7, initial/early period, prov. no. 9148";
    material="wood charcoal";
   };
   R_Date("Beta-75002CAMS",10260,60)
   {
    Outlier("General", 0.05);
    context="unit 7, initial/early period, prov. no. 9147";
    material="carbonised seed";
   };
   R_Date("GX19526CAMS (humate)",10570,70)
   {
    Outlier("General", 0.05);
    context="unit 8, early period, prov. no. 9204";
    material="humate from same";
   };
   R_Date("GX19524CAMS (humate)",10510,60)
   {
    Outlier("General", 0.05);
    context="unit 8, early period, prov. no. 9204";
    material="humate from same";
   };
   R_Date("GX19541CAMS (humate)",10490,80)
   {
    Outlier("General", 0.05);
    context="unit 11, early period, prov. no. 9296";
    material="humate from A. microcarpa seed";
   };
   R_Date("GX19526CAMS",10480,70)
   {
    Outlier("General", 0.05);
    context="unit 8, early period, prov. no. 9204";
    material="wood charcoal";
   };
   R_Date("GX19539CAMS",10470,70)
   {
    Outlier("General", 0.05);
    context="unit 11, early period, prov. no. 9296";
    material="wood charcoal";
   };
   R_Date("GX19523CAMS",10450,60)
   {
    Outlier("General", 0.05);
    context="unit 11, early period, prov. no. 9294";
    material="carbonised seed";
   };
   R_Date("GX19529CAMS",10420,70)
   {
    Outlier("General", 0.05);
    context="unit 9, early period, prov. no. 9245";
    material="carbonised seed";
   };
   R_Date("GX19540CAMS",10390,60)
   {
    Outlier("General", 0.05);
    context="unit 11, early period, prov. no. 9296";
    material="wood charcoal";
   };
   R_Date("GX19524CAMS",10380,60)
   {
    Outlier("General", 0.05);
    context="unit 8, early period, prov. no. 9204";
    material="carbonised seed";
   };
   R_Date("GX19525CAMS",10370,70)
   {
    Outlier("General", 0.05);
    context="unit 8, early period, prov. no. 9204";
    material="wood charcoal";
   };
   R_Date("GX19531CAMS",10370,60)
   {
    Outlier("General", 0.05);
    context="unit 9, early period, prov. no. 9246";
    material="carbonised seed";
   };
   R_Date("GX19533CAMS",10360,60)
   {
    Outlier("General", 0.05);
    context="unit 10, early period, prov. no. 9272";
    material="wood charcoal";
   };
   R_Date("GX19525CAMS (humate)",10330,70)
   {
    Outlier("General", 0.05);
    context="unit 8, early period, prov. no. 9204";
    material="humate from same";
   };
   R_Date("GX19527CAMS (humate)",10330,70)
   {
    Outlier("General", 0.05);
    context="unit 10, early period, prov. no. 9272";
    material="humate from same";
   };
   R_Date("GX19535CAMS",10310,70)
   {
    Outlier("General", 0.05);
    context="unit 10, early period, prov. no. 9272";
    material="wood charcoal";
   };
   R_Date("GX19527CAMS",10290,70)
   {
    Outlier("General", 0.05);
    context="unit 10, early period, prov. no. 9272";
    material="carbonised seed";
   };
   R_Date("GX19528CAMS",10290,70)
   {
```

```
   Outlier("General", 0.05);
   context="unit 10, early period, prov. no. 9272";
   material="carbonised seed";
  };
  R_Date("GX19530CAMS",10260,70)
  {
   Outlier("General", 0.05);
   context="unit 9, early period, prov. no. 9245";
   material="wood charcoal";
  };
  R_Date("GX19529CAMS (humate)",10250,70)
  {
   Outlier("General", 0.05);
   context="unit 9, early period, prov. no. 9245";
   material="humate from same";
  };
  R_Date("GX19540CAMS (humate)",10230,60)
  {
   Outlier("General", 0.05);
   context="unit 11, early period, prov. no. 9296";
   material="humate from same";
  };
  R_Date("GX19533CAMS (humate)",10220,60)
  {
   Outlier("General", 0.05);
   context="unit 10, early period, prov. no. 9272";
   material="humate from same";
  };
  R_Date("GX19530CAMS (humate)",10210,60)
  {
   Outlier("General", 0.05);
   context="unit 9, early period, prov. no. 9245";
   material="humate from same";
  };
  R_Date("GX19535CAMS (humate)",10210,70)
  {
   Outlier("General", 0.05);
   context="unit 10, early period, prov. no. 9272";
   material="humate from same";
  };
  R_Date("GX19532CAMS (humate)",10190,60)
  {
   Outlier("General", 0.05);
   context="unit 10, early period, prov. no. 9272";
   material="humate from same";
  };
  R_Date("GX19534CAMS (humate)",10190,50)
  {
   Outlier("General", 0.05);
   context="unit 10, early period, prov. no. 9272";
   material="humate from A. microcarpa seed";
  };
  R_Date("GX19531CAMS (humate)",10180,60)
  {
   Outlier("General", 0.05);
   context="unit 9, early period, prov. no. 9246";
   material="humate from same";
  };
  R_Date("GX19528CAMS (humate)",10120,70)
  {
   Outlier("General", 0.05);
   context="unit 10, early period, prov. no. 9272";
   material="humate from same";
  };
  R_Date("GX19532CAMS",10110,60)
  {
   Outlier("General", 0.05);
   context="unit 10, early period, prov. no. 9272";
   material="carbonised seed";
  };
  R_Date("GX19523CAMS (humate)",10000,60)
  {
   Outlier("General", 0.05);
   context="unit 11, early period, prov. no. 9294";
   material="humate from same";
  };
  R_Date("GX19539CAMS (humate)",10000,60)
  {
   Outlier("General", 0.05);
   context="unit 11, early period, prov. no. 9296";
   material="humate from same";
  };
  Date("Date early period");
  Interval("Duration early period");
 };
 Boundary("End early/Start late");
 Phase("late period")
 {
  R_Date("Beta-75001CAMS",10230,60)
  {
   Outlier("General", 0.05);
   context="unit 7, late period, prov. no. 9142";
   material="carbonised seed";
  };
  R_Date("Beta-76955CAMS",10210,60)
  {
   Outlier("General", 0.05);
   context="unit 11, late period, prov. no. 9290";
   material="carbonised seed";
  };
  R_Date("Beta-76954CAMS",10360,50)
  {
   Outlier("General", 0.05);
   context="unit 11, late period, prov. no. 9290";
   material="wood charcoal";
  };
  Interval("Duration late period");
  Interval("Date late period");
 };
 Boundary("End late");
};
Difference("Difference initial and early","Start initial","End initial/Start early");
Difference("Difference initial and late","Start initial","End early/Start late");
Difference("Difference early and late","End initial/Start early","End early/Start late");
Sequence()
{
 Boundary("=Start intial");
 Interval("Duration intial to late");
 Date("Date initial to late");
 Boundary("=End late");
};
};

Plot()
{
 Curve("IntCal20","intcal20.14c");
 Curve("SHCal20","shcal20.14c");
 Mix_Curve ("Mixed","IntCal20","SHCal20",U(0,100));
 Outlier_Model("General",T(5),U(0,4),"t");
 Sequence("Caverna da Pedra Pintada - Pereira data")
 {
  Boundary("Start I");
  Phase("I")
  {
   R_Date("Beta-434992",10310,30)
   {
    Outlier("General", 0.05);
    context="layer I, L-2";
   };
   R_Date("Beta-434993",10290,40)
   {
    Outlier("General", 0.05);
    context="layer I, L-2";
   };
   Date("Date of I");
  };
  Boundary("End I/Start II");
  Boundary("End II/Start III");
  R_Date("Beta-434991",10430,40)
  {
   Outlier("General", 0.05);
   context="layer III, K";
  };
  Boundary("End III/Start IV");
  Boundary("End III/Start V");
  Phase("V")
  {
   R_Date("Beta-434988",10100,40)
   {
    Outlier("General", 0.05);
    context="layer V, I";
   };
   R_Date("Beta-434989",10260,40)
   {
    Outlier("General", 0.05);
    context="layer V, J-2";
   };
   R_Date("Beta-434990",10360,40)
   {
    Outlier("General", 0.05);
    context="layer V, J-3";
   };
  };
  Boundary("End V/Start VI");
  Phase("VI")
  {
   R_Date("Beta-434987",8050,30)
   {
    Outlier("General", 0.05);
    context="layer VI, F-2";
   };
   R_Date("Beta-475220",5890,30)
   {
    Outlier("General", 0.05);
    context="layer VI, F-2";
   };
  };
  Boundary("End VI/Start VII");
  Phase("VII")
  {
   R_Date("Beta-434986",3080,30)
   {
    Outlier("General", 0.05);
    context="layer VII, E";
   };
   R_Date("Beta-475219",3990,30)
   {
    Outlier("General", 0.05);
    context="layer VII, F";
   };
  };
  Boundary("End VII/Start VIII");
  R_Date("Beta-434985",590,30)
  {
   Outlier("General", 0.05);
   context="layer VIII, C";
  };
  Boundary("End VIII/Start IX");
  R_Date("Beta-434984",1720,30)
  {
   Outlier("General", 0.05);
   context="layer IX, B";
  };
  Boundary("End IX");
 };
};
```

## 2.2.4. Lapa do Boquete

Lapa do Boquete is a cave site located in Brazil (-14.98, -44.44), containing lithic technology (Itaparica) and human remains[33,111–118]. There are nine strata as defined by sedimentological characteristics, with archaeological levels denoted by ‘upper’, ‘middle’, ‘lower’ and ‘base’ sections (see Fig. 5.21 in Kipnis[33]). Stratum 9 is archaeologically sterile.

Bayesian modelling estimates the start of strata 8 at 14600-12625 cal BP (Itaparica), 7 at 12860-11205 cal BP and 6 at 11975-10355 cal BP, with eleven outliers (see Supplementary Figure 45 and OxCal code). The discrepant ages likely denote vertical mixing, inbuilt age and/or pretreatment issues. Three dates (clear outliers) were removed from the model as it failed to converge with these included (CDTN-2731, CDTN-1087), and a third with no laboratory code but an age of 3540 ± 150 BP. Following strata 8-6, stratum 5 and 4 are noted as being poor in archaeological vestiges[33]. Stratum 3, containing basketry, is estimated to have started at 8690-7905 cal BP.

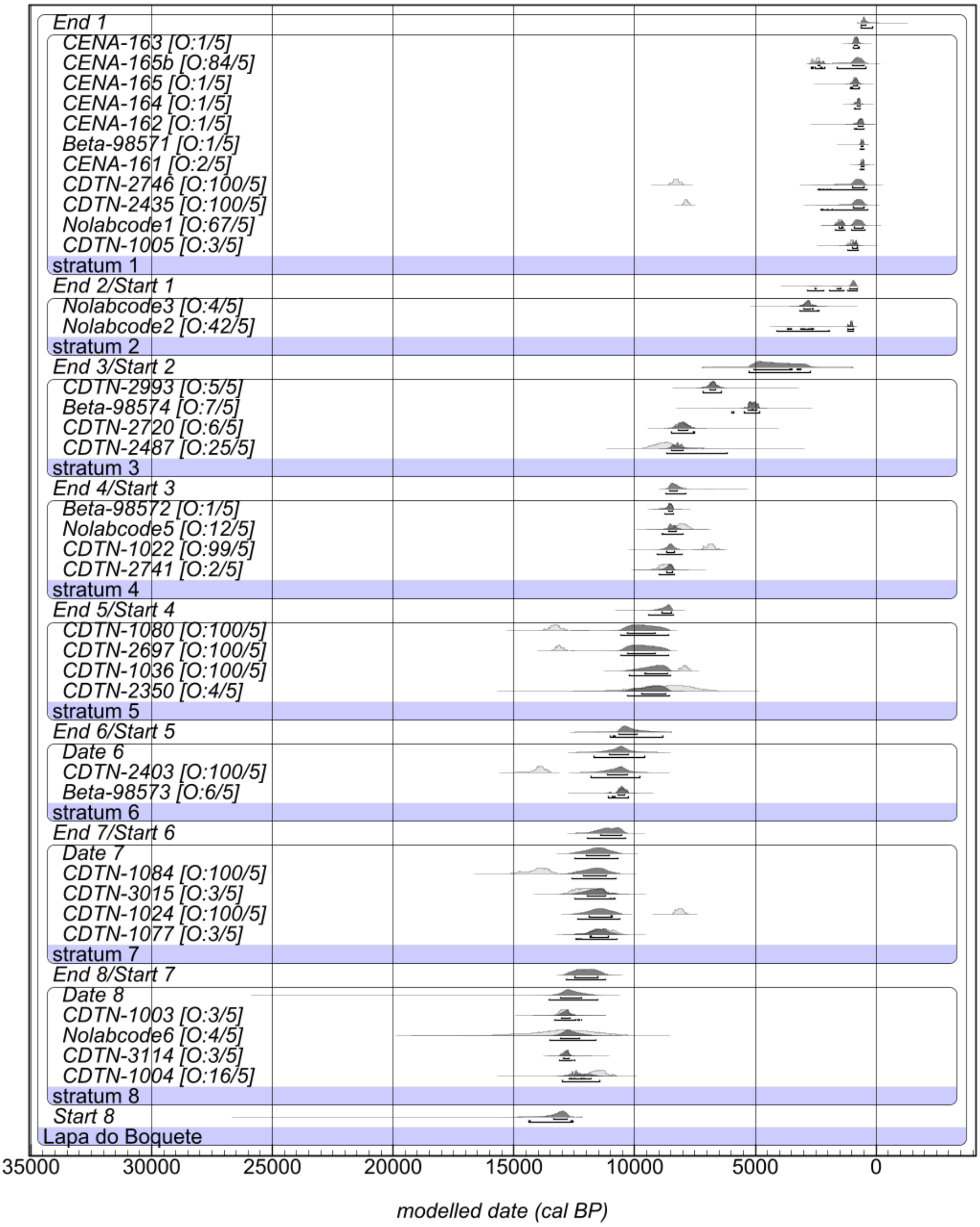


**Supplementary Figure 45.** Bayesian age model for Lapa do Boquete. Brackets beneath each age estimate show 68.3% and 95.4% CI. Outlier analysis output is noted as 'O:posterior probability/prior probability'.

```
Plot()
{
 Curve("SHCal20","shcal20.14c");
 Outlier_Model("General",T(5),U(0,4),"t");
 Sequence("Lapa do Boquete")
 {
  Boundary("Start 8");
  Phase("stratum 8")
  {
   R_Date("CDTN-1004",10000,232)
   {
    Outlier("General", 0.05);
    context="square K11, stratum or level 8";
   };
   R_Date("CDTN-3114",10910,140)
   {
    Outlier("General", 0.05);
    context="square K9, stratum or level 8 middle";
   };
   R_Date("Nolabcode6",11000,1000)
   {
    Outlier("General", 0.05);
    context="square L11, stratum or level 8";
   };
   R_Date("CDTN-1003",11000,232)
   {
    Outlier("General", 0.05);
    context="square M10, stratum or level 8 middle";
   };
   Date("Date 8");
   Interval("Duration 8");
  };
  Boundary("End 8/Start 7");
  Phase("stratum 7")
  {
   R_Date("CDTN-1077",9870,260)
   {
    Outlier("General", 0.05);
    context="square K11, stratum or level 7 upper";
   };
   R_Date("CDTN-1024",7350,160)
   {
    Outlier("General", 0.05);
    context="square M11, stratum or level 7";
   };
   R_Date("CDTN-3015",10250,345)
   {
    Outlier("General", 0.05);
    context="square K11, stratum or level 7 middle";
   };
   R_Date("CDTN-1084",12000,300)
   {
    Outlier("General", 0.05);
    context="square J10, stratum or level 7 base/8";
   };
   Date("Date 7");
   Interval("Duration 7");
  };
  Boundary("End 7/Start 6");
  Phase("stratum 6")
  {
   R_Date("Beta-98573",9350,80)
   {
    Outlier("General", 0.05);
    context="square M18, stratum or level 6 b";
   };
   R_Date("CDTN-2403",12070,170)
   {
    Outlier("General", 0.05);
    context="square K10, stratum or level 6 base";
   };
   Interval("Duration 6");
   Date("Date 6");
  };
  Boundary("End 6/Start 5");
  Phase("stratum 5")
  {
   R_Date("CDTN-2350",7585,1000)
   {
    Outlier("General", 0.05);
    context="square J12, stratum or level 4/5";
   };
   R_Date("CDTN-1036",7155,140)
   {
    Outlier("General", 0.05);
    context="square S2, stratum or level 5 (burial 1)";
   };
   R_Date("CDTN-2697",11250,150)
   {
    Outlier("General", 0.05);
    context="square K11, stratum or level 5 lower";
   };
   R_Date("CDTN-1080",11440,240)
   {
    Outlier("General", 0.05);
    context="square L8, stratum or level 5 lower";
   };
   Interval("Duration 5");
  };
  Boundary("End 5/Start 4");
  Phase("stratum 4")
  {
   R_Date("CDTN-2741",7900,180)
   {
    Outlier("General", 0.05);
    context="square K11, stratum or level 4 upper";
   };
   R_Date("CDTN-1022",6040,120)
   {
    Outlier("General", 0.05);
    context="square S2, stratum or level 4 (burial 1)";
   };
   R_Date("Nolabcode5",7240,220)
   {
    Outlier("General", 0.05);
    context="square S2, stratum or level 4";
   };
   R_Date("Beta-98572",7810,80)
   {
    Outlier("General", 0.05);
    context="square M19, stratum or level 4 middle";
   };
   Interval("Duration 4");
  };
  Boundary("End 4/Start 3");
  Phase("stratum 3")
  {
   R_Date("CDTN-2487",7940,300)
   {
    Outlier("General", 0.05);
    context="square K11, stratum or level 3 upper";
   };
   R_Date("CDTN-2720",7260,210)
   {
    Outlier("General", 0.05);
    context="square K9, stratum or level 3";
   };
   R_Date("Beta-98574",4480,70)
   {
    Outlier("General", 0.05);
    context="square M21, stratum or level 3 lower (burial 4)";
   };
   R_Date("CDTN-2993",5960,100)
   {
    Outlier("General", 0.05);
    context="square K11, stratum or level 3 lower";
   };
   Interval("Duration 3");
  };
  Boundary("End 3/Start 2");
  Phase("stratum 2")
  {
   R_Date("Nolabcode2",1180,40)
   {
    Outlier("General", 0.05);
    context="square M11, stratum or level 2";
   };
   //not sure where to place
   R_Date("Nolabcode3",2740,120)
   {
    Outlier("General", 0.05);
    context="square S2, stratum or level burial";
   };
   Interval("Duration 2");
  };
  Boundary("End 2/Start 1");
  Phase("stratum 1")
  {
   R_Date("CDTN-1005",1120,100)
   {
    Outlier("General", 0.05);
    context="square M19, stratum or level 0/1";
   };
   R_Date("Nolabcode1",1650,100)
   {
    Outlier("General", 0.05);
    context="square S1, stratum or level I";
   };
   R_Date("CDTN-2435",7080,80)
   {
    Outlier("General", 0.05);
    context="square K11, stratum or level 1 middle/1 lower";
   };
   R_Date("CDTN-2746",7520,140)
   {
    Outlier("General", 0.05);
    context="square L8, stratum or level 1 lower base";
   };
   R_Date("CENA-161",570,60)
   {
    Outlier("General", 0.05);
    context="square N19, stratum or level silo 4";
   };
   R_Date("Beta-98571",600,50)
   {
    Outlier("General", 0.05);
    context="square N19, stratum or level silo 4";
   };
   R_Date("CENA-162",730,120)
   {
    Outlier("General", 0.05);
    context="square N19, stratum or level silo 4";
   };
   R_Date("CENA-164",860,60)
   {
    Outlier("General", 0.05);
    context="square N19, stratum/level silo 4";
   };
   R_Date("CENA-165",1010,80)
   {
    Outlier("General", 0.05);
    context="square M19, stratum or level silo 4";
   };
   //same code as another
   R_Date("CENA-165b",2420,70)
   {
    Outlier("General", 0.05);
    context="square M19, stratum or level silo 4";
   };
   R_Date("CENA-163",960,60)
   {
    Outlier("General", 0.05);
    context="square M19, stratum or level silo 1";
   };
   Interval("Duration 1");
  };
  Boundary("End 1");
 };
 Difference("Difference 8 and 7","Start 8","End 8/Start 7");
 Difference("Difference 7 and 6","End 8/Start 7","End 7/Start 6");
 Sequence()
 {
  Boundary("=Start 8");
  Interval("Duration 8-6");
  Date("Date 8-6");
  Boundary("=End 6/Start 5");
 };
};
//manually excluded clear outlier as model fails to run
//R_Date("CDTN-2731",10200,250)
//context=square L8, stratum or level 2 middle;
//R_Date("Nolabcode4",3540,150)
//context=square M11, stratum or level 4;
//R_Date("CDTN-1087",1350,150)
//context=square J10, stratum or level 8 base;
```

2.2.5. Lapa dos Bichos

Lapa dos Bichos is a cave site located in Brazil (-14.98, -44.44; four kilometres from Lapa do Boquete), containing lithic technology and hearths[33]. There are nine strata, with the bottom layer (9) being archaeologically sterile (see Fig. 5.26. in Kipnis[33]).

Bayesian modelling estimates the start of stratum 8 at 15985-1190 cal BP or 13445-12065 cal BP (68.3% CI; Itaparica) and stratum 7 at 12204-10117 cal BP (both overlap at 95% CI), with no outliers (see Supplementary Figure 46 and OxCal code). Following strata 8 and 7, cultural activity is evidenced at stratum 6 (although noted as containing 'little archaeological material' by Kipnis[33], which is estimated to have started at 10085-8280 cal BP.

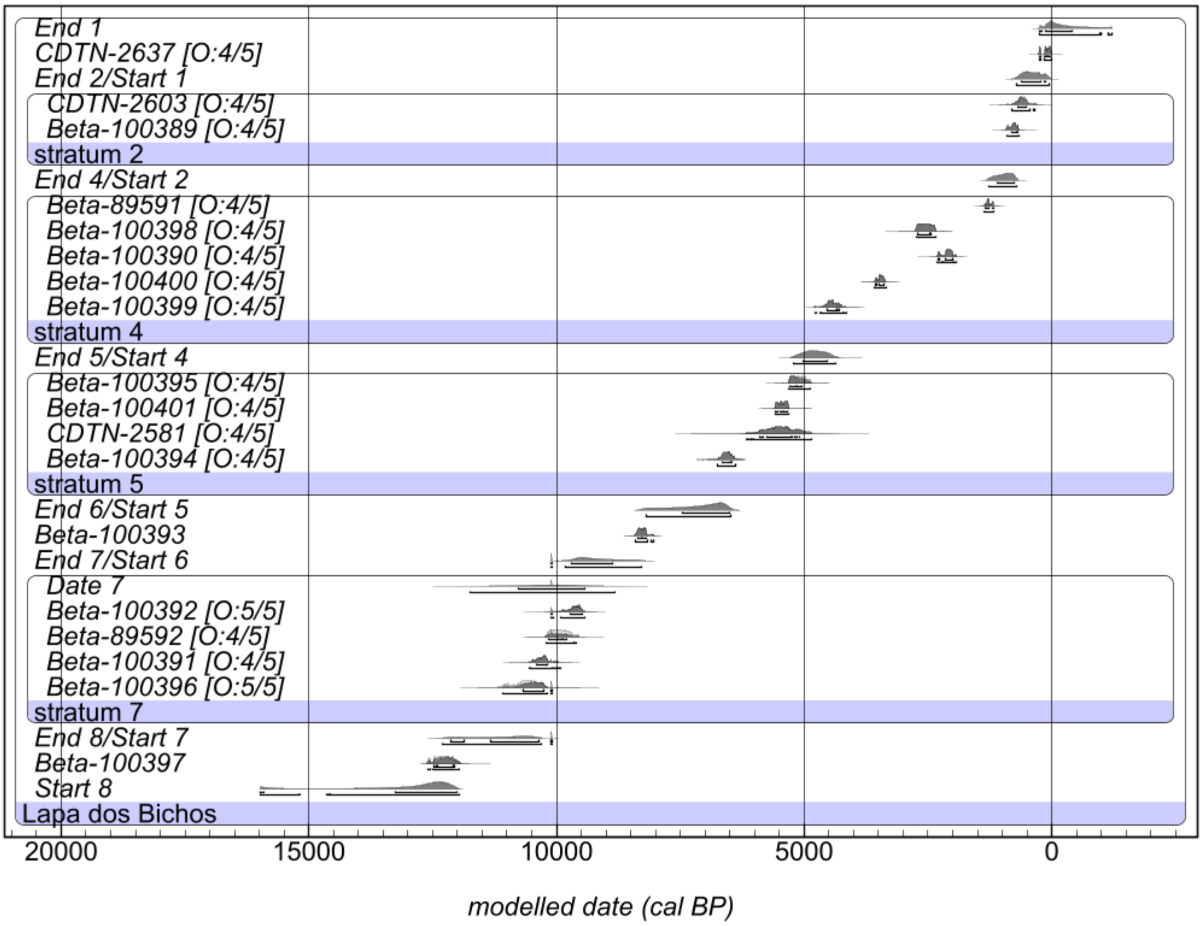


**Supplementary Figure 46.** Bayesian age model for Lapa dos Bichos. Brackets beneath each age estimate show 68.3% and 95.4% CI. Outlier analysis output is noted as 'O:posterior probability/prior probability'.

```
Plot()
{
 Curve("SHCal20","shcal20.14c");
 Outlier_Model("General",T(5),U(0,4),"t");
 Sequence("Lapa dos Bichos")
 {
  Boundary("Start 8");
  R_Date("Beta-100397",10450,70)
  {
   context=square G 17, stratum 8;
  };
  Boundary("End 8/Start 7");
  Phase("stratum 7")
  {
   R_Date("Beta-100396",9390,160)
   {
    Outlier("General", 0.05);
    context="square G 17, stratum 7";
   };
   R_Date("Beta-100391",9140,90)
   {
    Outlier("General", 0.05);
    context="square O 20, stratum 7";
   };
   R_Date("Beta-89592",8890,90)
   {
    Outlier("General", 0.05);
    context="square O 20, stratum 7";
   };
   R_Date("Beta-100392",8640,90)
   {
    Outlier("General", 0.05);
    context="square O 20, stratum 7";
   };
   Interval("Duration 7");
   Date("Date 7");
  };
  Boundary("End 7/Start 6");
  R_Date("Beta-100393",7300,70)
  {
   context="square O 20, stratum 6";
  };
  Boundary("End 6/Start 5");
  Phase("stratum 5")
  {
   R_Date("Beta-100394",5800,70)
   {
    Outlier("General", 0.05);
    context="square O 20, stratum 5";
   };
   R_Date("CDTN-2581",4800,270)
   {
    Outlier("General", 0.05);
    context="square K 13, stratum 5";
   };
   R_Date("Beta-100401",4750,70)
   {
    Outlier("General", 0.05);
    context="square K 24, stratum 5";
   };
   R_Date("Beta-100395",4500,70)
   {
    Outlier("General", 0.05);
    context="square C 30, stratum 5";
   };
   Interval("Duration 5");
  };
  Boundary("End 5/Start 4");
  Phase("stratum 4")
  {
   R_Date("Beta-100399",4020,70)
   {
    Outlier("General", 0.05);
    context="square P 11, stratum 4";
   };
   R_Date("Beta-100400",3280,50)
   {
    Outlier("General", 0.05);
    context="square K 24, stratum 4";
   };
   R_Date("Beta-100390",2160,60)
   {
    Outlier("General", 0.05);
    context="square G 17, stratum 4";
   };
   R_Date("Beta-100398",2510,80)
   {
    Outlier("General", 0.05);
    context="square K 24, stratum 4";
   };
   R_Date("Beta-89591",1410,50)
   {
    Outlier("General", 0.05);
    context="square G 17, stratum 4";
   };
   Interval("Duration 4");
  };
  Boundary("End 4/Start 2");
  Phase("stratum 2")
  {
   R_Date("Beta-100389",910,60)
   {
    Outlier("General", 0.05);
    context="square C 30, stratum 2";
   };
   R_Date("CDTN-2603",625,125)
   {
    Outlier("General", 0.05);
    context="square K 12, stratum 2";
   };
   Interval("Duration 2");
  };
  Boundary("End 2/Start 1");
  R_Date("CDTN-2637",130,20)
  {
   Outlier("General", 0.05);
   context="square K 12, stratum 1";
  };
  Boundary("End 1");
 };
 Difference("Difference 8 and 7","Start 8","End 8/Start 7");
 Sequence()
 {
  Boundary("=Start 8");
  Interval("Duration 8");
  Date("Date 8");
  Boundary("=End 8/Start 7");
 };
};
```

## 2.2.6. Lapa Vermelha IV

Lapa Vermelha IV is a cave site located in Brazil (-19.666667, -43.883333), containing human remains and extinct fauna[119–121]. There are three strata (A-C) as defined by Feathers et al.[119] in a reanalysis of depositional data. An individual named ‘Luzia’ and represented by a disarticulated skeleton was found at the site, but there are uncertainties regarding their provenience (elements likely moved as the rockshelter surface slopes from north to south). It is believed, however, that Luiza was recovered near the interface of strata A and C.

A Bayesian model using OSL ages from strata A and C, plus the two radiocarbon measurements that potentially bracket Luzia (Gif-3727 and -3903), was created. Given the large uncertainties for the OSL ages, the provenience of Luiza and the correlation of radiocarbon ages with the OSL chronology, this is not reported. As such, this site is not included in the Bayesian analysis.

## 2.2.7. Santa Elina

Santa Elina is a rockshelter site located in Brazil (-15.4577778, -56.7925), containing lithic technology and extinct faunal remains (*Glossotherium letsomi*)[122–128]. There are four stratigraphic units at site (I-IV) as defined by sedimentological, archaeological and palaeontological evidence (see Fig. 1 in Pansani et al.[126]). Unit IV does not contain archaeological material. Units III (3 & 4) and II (1b & 2) include spatially associated cultural and paleontological evidence.

Bayesian modelling estimates the start of Unit II at 21710-16790 cal BP (or 20320-17750 cal BP at 68.3% CI), with no outliers (Supplementary Figure 47). The age range for this component is estimated at 18965-7815 cal BP, overlapping the ACR-YD period. Following Unit II, cultural activity is evidenced at Unit I, which is estimated to have started at 7945-7690 cal BP. Sensitivity testing shows that the incorporation of UGAMS-51687 and -51688 as the calculated bone collagen dates rather than the original apatite measurements makes no significant difference to model outputs (start of Unit II; Supplementary Figures 48-49). The estimates here provided are yielded from the model that uses the apatite dates.

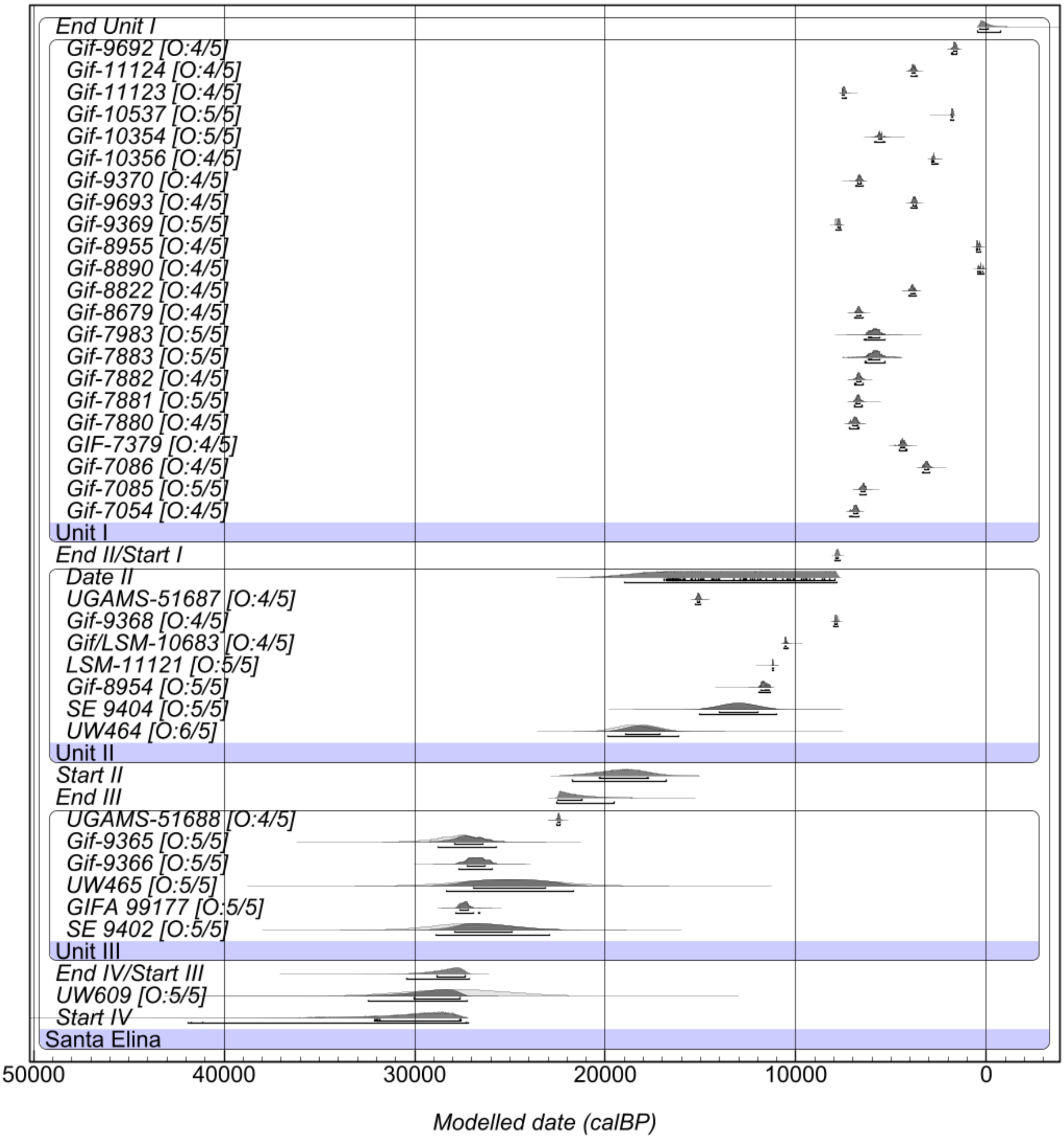


**Supplementary Figure 47.** Bayesian age model for Santa Elina (with original bone apatite dates). Brackets beneath each age estimate show 68.3% and 95.4% CI. Outlier analysis output is noted as 'O:posterior probability/prior probability'.

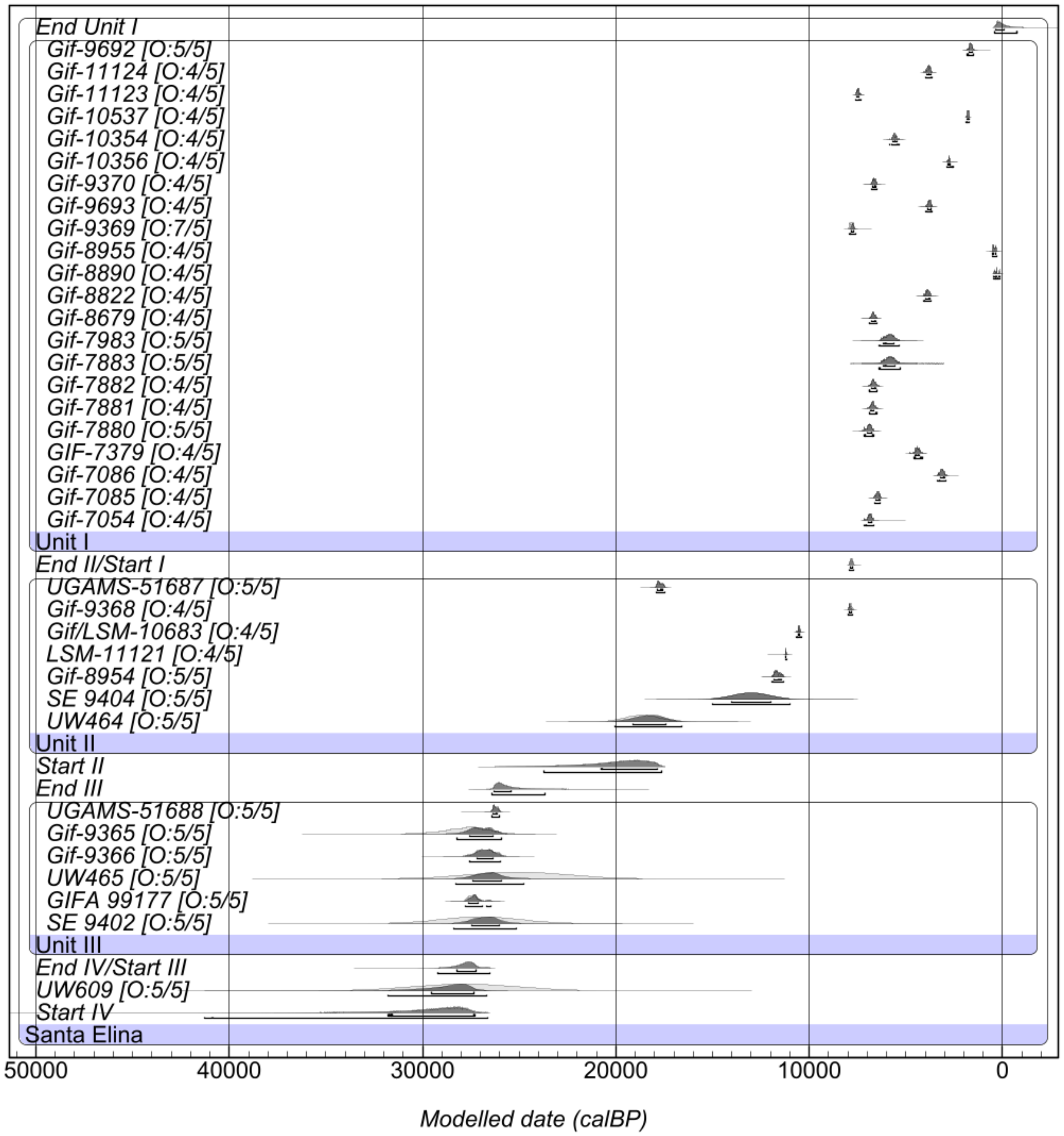


**Supplementary Figure 48.** Bayesian age model for Santa Elina (with calculated bone collagen dates). Brackets beneath each age estimate show 68.3% and 95.4% CI. Outlier analysis output is noted as ‘O:posterior probability/prior probability’.

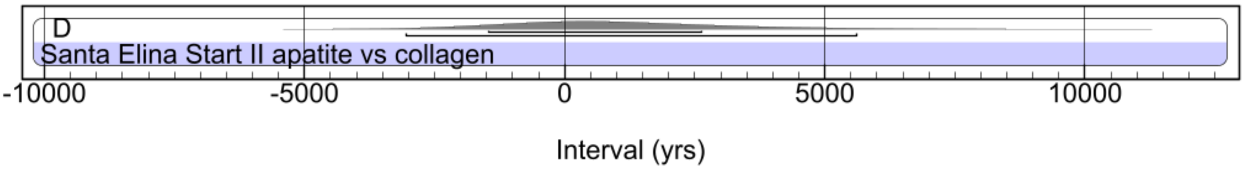


**Supplementary Figure 49.** Probability density function for the difference (‘D’) between the start of Unit II using the original apatite dates (Supplementary Figure 47) vs the calculated collagen ages (Supplementary Figure 48). These results suggest that there is no significant difference between the modelled outputs, as the distributions include zero at 95.4% CI (black bracket beneath).

```
Plot()
{
 Curve("IntCal20","intcal20.14c");
 Curve("SHCal20","shcal20.14c");
 Mix_Curve ("Mixed","IntCal20","SHCal20",U(0,100));
 Outlier_Model("General",T(5),U(0,4),"t");
 Sequence("Santa Elina")
 {
  Boundary("Start IV");
  Phase("Unit IV")
  {
   //collection date date estimated from publication date (2005)
   Date("UW466", N(2004-31800,1600))
   {
    Outlier("General", 0.05);
   };
   //collection date date estimated from publication date (2005)
   Date("UW609", N(2004-27800,2700))
   {
    Outlier("General", 0.05);
   };
  };
  Boundary("End IV/Start III");
  Phase("Unit III")
  {
   //collection date date estimated from publication date (2005)
   Date("UW610", N(2004-35500, 2400))
   {
    Outlier("General", 0.05);
   };
   //collection date date estimated from publication date (2005)
   Date("UW462", N(2004-27800, 1800))
   {
    Outlier("General", 0.05);
   };
   //collection date date estimated from publication date (2005)
   Date("UW465", N(2004-25100, 2500))
   {
    Outlier("General", 0.05);
   };
   Date("SE 9402", N(calBP(27000),2000))
   {
    Outlier("General", 0.05);
   };
   R_Date("Gif-9365",23320,1000)
   {
    Outlier("General", 0.05);
   };
   R_Date("GIFA 99177",23120,260)
   {
    Outlier("General", 0.05);
   };
   R_Date("Gif-9366",22500,500)
   {
    Outlier("General", 0.05);
   };
   R_Date("UGAMS-51688", 18580, 45)
   {
    color="red";
    Outlier("General", 0.05);
   };
   Interval("Duration III");
  };
  Boundary("End III");
  Interval("Duration gap III to II");
  Boundary("Start II");
  Phase("Unit II")
  {
   //measurement date estimated from publication date (2005)
   Date("UW464", N(2004-18700, 900))
   {
    Outlier("General", 0.05);
   };
   Date("SE 9404", N(calBP(13000),1000))
   {
    Outlier("General", 0.05);
   };
   R_Date("Gif-8954",10120,60)
   {
    Outlier("General", 0.05);
   };
   R_Date("LSM-11121",9790,20)
   {
    Outlier("General", 0.05);
   };
   R_Date("Gif/LSM-10683",9340,20)
   {
    Outlier("General", 0.05);
   };
   R_Date("Gif-9368",7050,55)
   {
    Outlier("General", 0.05);
   };
   R_Date("UGAMS-51687", 12690, 40)
   {
    color="red";
    Outlier("General", 0.05);
   };
   Interval("Duration II");
   Date("Date II");
  };
  Boundary("End II/Start I");
  Phase("Unit I")
  {
   R_Date("Gif-7054",6040,70)
   {
    Outlier("General", 0.05);
   };
   R_Date("Gif-7085", 5690, 70)
   {
    Outlier("General", 0.05);
   };
   R_Date("Gif-7086", 2990, 60)
   {
    Outlier("General", 0.05);
   };
   R_Date("GIF-7379", 3970, 60)
   {
    Outlier("General", 0.05);
   };
   R_Date("Gif-7880", 6060, 80)
   {
    Outlier("General", 0.05);
   };
   R_Date("Gif-7881", 5920, 70)
   {
    Outlier("General", 0.05);
   };
   R_Date("Gif-7882", 5890, 70)
   {
    Outlier("General", 0.05);
   };
   R_Date("Gif-7883", 5080, 230)
   {
    Outlier("General", 0.05);
   };
   R_Date("Gif-7983", 5110, 230)
   {
    Outlier("General", 0.05);
   };
   R_Date("Gif-8679", 5890, 70)
   {
    Outlier("General", 0.05);
   };
   R_Date("Gif-8822", 3600, 60)
   {
    Outlier("General", 0.05);
   };
   R_Date("Gif-8890", 275, 40)
   {
    Outlier("General", 0.05);
   };
   R_Date("Gif-8955", 400, 50)
   {
    Outlier("General", 0.05);
   };
   R_Date("Gif-9369", 7010, 70)
   {
    Outlier("General", 0.05);
   };
   R_Date("Gif-9693", 3530, 50)
   {
    Outlier("General", 0.05);
   };
   R_Date("Gif-9370", 5860, 60)
   {
    Outlier("General", 0.05);
   };
   R_Date("Gif-10356", 2660, 50)
   {
    Outlier("General", 0.05);
   };
   R_Date("Gif-10354", 4880, 70)
   {
    Outlier("General", 0.05);
   };
   R_Date("Gif-10537", 1890, 20)
   {
    Outlier("General", 0.05);
   };
   R_Date("Gif-11123", 6590, 60)
   {
    Outlier("General", 0.05);
   };
   R_Date("Gif-11124", 3560, 50)
   {
    Outlier("General", 0.05);
   };
   R_Date("Gif-9692",1770,60)
   {
    Outlier("General", 0.05);
   };
   Interval("Duration I");
  };
  Boundary("End Unit I");
 };
};
```

```
Plot()
{
 Curve("IntCal20","intcal20.14c");
 Curve("SHCal20","shcal20.14c");
 Mix_Curve ("Mixed","IntCal20","SHCal20",U(0,100));
 Outlier_Model("General",T(5),U(0,4),"t");
 Sequence("Santa Elina")
 {
  Boundary("Start IV");
  Phase("Unit IV")
  {
   //collection date date estimated from publication date (2005)
   Date("UW466", N(2004-31800,1600))
   {
    Outlier("General", 0.05);
   };
   //collection date date estimated from publication date (2005)
   Date("UW609", N(2004-27800,2700))
   {
    Outlier("General", 0.05);
   };
  };
  Boundary("End IV/Start III");
  Phase("Unit III")
  {
   //collection date date estimated from publication date (2005)
   Date("UW610", N(2004-35500, 2400))
   {
    Outlier("General", 0.05);
   };
   //collection date date estimated from publication date (2005)
   Date("UW462", N(2004-27800, 1800))
   {
    Outlier("General", 0.05);
   };
   //collection date date estimated from publication date (2005)
   Date("UW465", N(2004-25100, 2500))
   {
    Outlier("General", 0.05);
   };
   Date("SE 9402", N(calBP(27000),2000))
   {
    Outlier("General", 0.05);
   };
   R_Date("Gif-9365",23320,1000)
   {
    Outlier("General", 0.05);
   };
   R_Date("GIFA 99177",23120,260)
   {
    Outlier("General", 0.05);
   };
   R_Date("Gif-9366",22500,500)
   {
    Outlier("General", 0.05);
   };
   //calculated collagen date
   R_Date("UGAMS-51688", 22042, 45)
   {
    Outlier("General", 0.05);
   };
   Interval("Duration III");
  };
  Boundary("End III");
  Interval("Duration gap III to II");
  Boundary("Start II");
  Phase("Unit II")
  {
   //measurement date estimated from publication date (2005)
   Date("UW464", N(2004-18700, 900))
   {
    Outlier("General", 0.05);
   };
   Date("SE 9404", N(calBP(13000),1000))
   {
    Outlier("General", 0.05);
   };
   R_Date("Gif-8954",10120,60)
   {
    Outlier("General", 0.05);
   };
   R_Date("LSM-11121",9790,20)
   {
    Outlier("General", 0.05);
   };
   R_Date("Gif/LSM-10683",9340,20)
   {
    Outlier("General", 0.05);
   };
   R_Date("Gif-9368",7050,55)
   {
    Outlier("General", 0.05);
   };
   //calculated collagen date
   R_Date("UGAMS-51687", 14547, 40)
   {
    Outlier("General", 0.05);
   };
   Interval("Duration II");
   Date("Date II");
  };
  Boundary("End II/Start I");
  Phase("Unit I")
  {
   R_Date("Gif-7054",6040,70)
   {
    Outlier("General", 0.05);
   };
   R_Date("Gif-7085", 5690, 70)
   {
    Outlier("General", 0.05);
   };
   R_Date("Gif-7086", 2990, 60)
   {
    Outlier("General", 0.05);
   };
   R_Date("GIF-7379", 3970, 60)
   {
    Outlier("General", 0.05);
   };
   R_Date("Gif-7880", 6060, 80)
   {
    Outlier("General", 0.05);
   };
   R_Date("Gif-7881", 5920, 70)
   {
    Outlier("General", 0.05);
   };
   R_Date("Gif-7882", 5890, 70)
   {
    Outlier("General", 0.05);
   };
   R_Date("Gif-7883", 5080, 230)
   {
    Outlier("General", 0.05);
   };
   R_Date("Gif-7983", 5110, 230)
   {
    Outlier("General", 0.05);
   };
   R_Date("Gif-8679", 5890, 70)
   {
    Outlier("General", 0.05);
   };
   R_Date("Gif-8822", 3600, 60)
   {
    Outlier("General", 0.05);
   };
   R_Date("Gif-8890", 275, 40)
   {
    Outlier("General", 0.05);
   };
   R_Date("Gif-8955", 400, 50)
   {
    Outlier("General", 0.05);
   };
   R_Date("Gif-9369", 7010, 70)
   {
    Outlier("General", 0.05);
   };
   R_Date("Gif-9693", 3530, 50)
   {
    Outlier("General", 0.05);
   };
   R_Date("Gif-9370", 5860, 60)
   {
    Outlier("General", 0.05);
   };
   R_Date("Gif-10356", 2660, 50)
   {
    Outlier("General", 0.05);
   };
   R_Date("Gif-10354", 4880, 70)
   {
    Outlier("General", 0.05);
   };
   R_Date("Gif-10537", 1890, 20)
   {
    Outlier("General", 0.05);
   };
   R_Date("Gif-11123", 6590, 60)
   {
    Outlier("General", 0.05);
   };
   R_Date("Gif-11124", 3560, 50)
   {
    Outlier("General", 0.05);
   };
   R_Date("Gif-9692",1770,60)
   {
    Outlier("General", 0.05);
   };
   Interval("Duration I");
  };
  Boundary("End Unit I");
 };
};
```

### 2.2.8. RS-I-69 (Laranjito)

RS-I-69 (Laranjito) is a site located in Brazil (-14.98, -44.44), containing lithic technology[129]. The lithic technology found is noted as ‘Uruguai’, which includes bifaces, unifaces and projectile points. Limited stratigraphic information is provided in Dias and Jacobus[129], but the concentration of archaeological material is noted a z range of 680-710 cm, with the oldest measurement SI-2630 (10985±100 BP), an outlier, found 2 m above more reliable dates SI-2631 (9620 ± 110 BP) and SI-3106 (10240±80 BP). As such, a single-phase model was constructed using five measurements.

A single-phase Bayesian model estimates the start of occupation at 14780-11845 cal BP (see Supplementary Figure 50 and OxCal code).

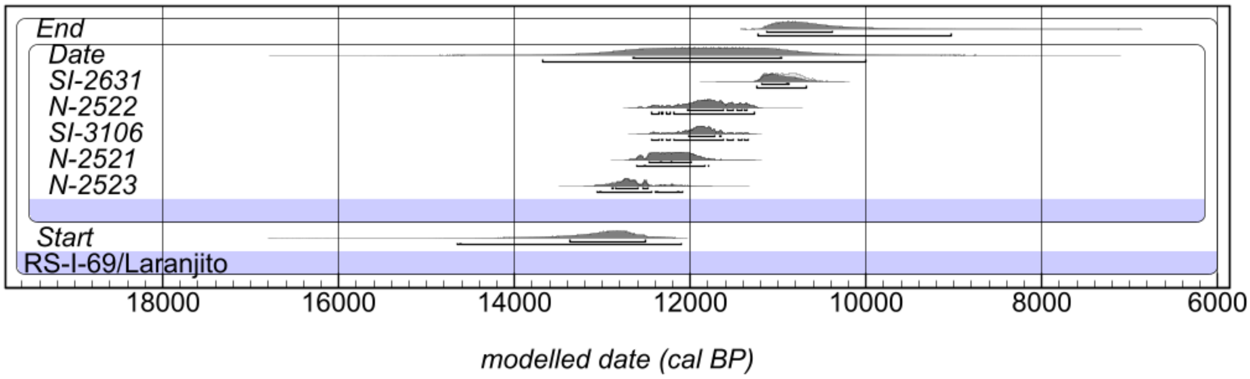


**Supplementary Figure 50.** Bayesian age model for *RS-I-69*. Brackets beneath each age estimate show 68.3% and 95.4% CI.

```
Plot()
{
 Curve("SHCal20","shcal20.14c");
 Sequence("RS-I-69/Laranjito")
 {
  Boundary("Start");
  Phase("")
  {
   R_Date("N-2523",10800,150);
   R_Date("N-2521",10400,110);
   R_Date("SI-3106",10240,80);
   R_Date("N-2522",10200,125);
   R_Date("SI-2631",9620,110);
   Date("Date");
   Interval("Duration");
  };
  Boundary("End");
 };
};
```

## 2.2.9. Santana do Riacho

Santana do Riacho is a rockshelter site located in Brazil (approximately at -19.2, -43.7), containing lithic technology, human remains and hearth features[33,130–134]. There are seven stratigraphic units within three different excavation units (SR1-3), with SR1 containing most human burials (see Fig. 4 in Neves et al.[130]). Strata 0, 5, and 6 are archaeologically sterile, and stratum 3 contains most human burials. It is unclear whether stratum 7 represents cultural or merely geological activity (charcoal from natural fires).

A single-phase Bayesian model including all bone ages apart from Beta-96759 estimates the start of burial and potentially cultural activity at 14465-9030 cal BP (see Supplementary Figure 51 and OxCal code). Beta-96759 was excluded as the $\delta^{13}$C value, -33.2‰, is anomalous and there is no other data with which to assess reliability. Following stratum 3, cultural activity is evidenced at stratum 2, which includes ages 8185 ± 110 BP (CDTN-1039) and 8381 ± 280 BP [CDTN-1044; both at approximately 9200 cal BP (mean)]. Sensitivity testing shows that the 14465-9030 cal BP estimate for the potential start of cultural activity is comparable to that obtained from a multi-phase model containing unidentified charcoal samples from strata 1 and 3 at SR3 (Supplementary Figures 52-53).

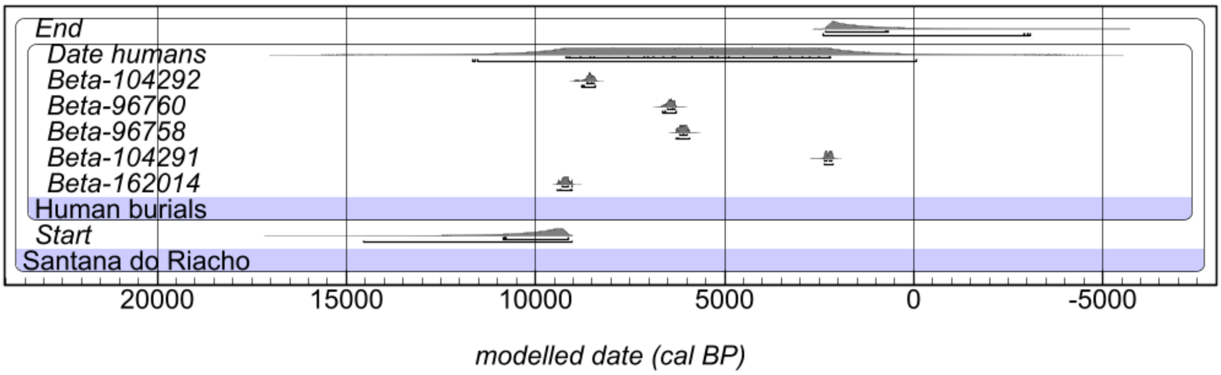


**Supplementary Figure 51.** Bayesian age model for Santana do Riacho (all bone ages). Brackets beneath each age estimate show 68.3% and 95.4% CI.

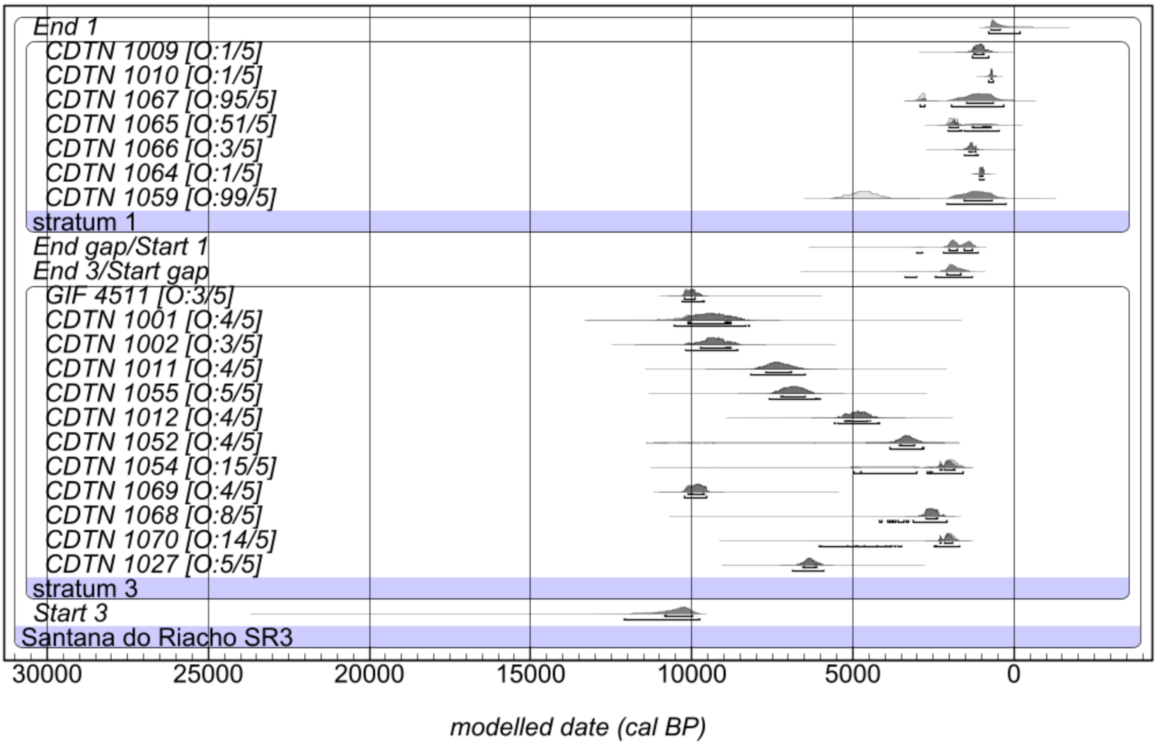


**Supplementary Figure 52.** Bayesian age model for Santana do Riacho (unidentified charcoal samples from strata 1 and 3 at SR3). Brackets beneath each age estimate show 68.3% and 95.4% CI. Outlier analysis output is noted as ‘O:posterior probability/prior probability’.

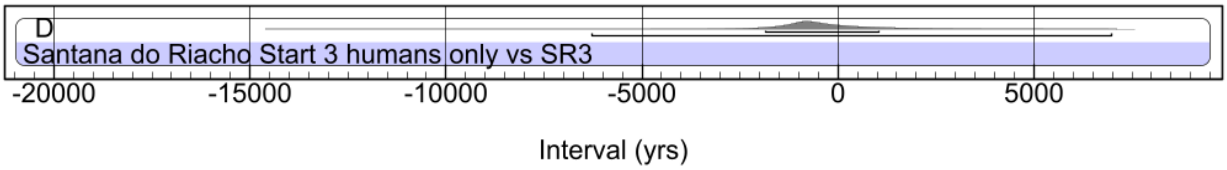


**Supplementary Figure 53.** Probability density function for the difference (‘D’) between the start of cultural activity at Santana do Riacho using all bone dates [Supplementary Figure 51 vs unidentified charcoal samples from strata 1 and 3 at SR3 (Supplementary Figure 52)]. These results suggest that there is no significant difference between the modelled outputs, as the distributions include zero at 95.4% CI (black bracket beneath).

```
Plot()
{
 Curve("SHCal20","shcal20.14c");
 Sequence("Santana do Riacho")
 {
  Boundary("Start");
  Phase("Human burials")
  {
   R_Date("Beta-162014", 8280, 40);
   R_Date("Beta-104291", 2310, 50);
   R_Date("Beta-96758", 5350, 60);
   R_Date("Beta-96760", 5680, 70);
   R_Date("Beta-104292", 7820, 60);
   Date("Date humans");
  };
  Boundary("End");
 };
};
//not used R_Date("Beta-96759", 12760, 70)

Plot()
{
 Curve("SHCal20","shcal20.14c");
 Outlier_Model("General",T(5),U(0,4),"t");
 Sequence("Santana do Riacho SR3")
 {
  Boundary("Start 3");
  Phase("stratum 3")
  {
   R_Date("CDTN 1027",5580,140)
   {
    Outlier("General", 0.05);
    context="SR3, zone/square Q 29, stratum 3";
   };
   R_Date("CDTN 1070",2070,100)
   {
    Outlier("General", 0.05);
    context="SR3, zone/square Q 32, stratum 3 upper";
   };
   R_Date("CDTN 1068",2530,120)
   {
    Outlier("General", 0.05);
    context="SR3, zone/square KL 36, stratum 3 lower";
   };
   R_Date("CDTN 1069",8840,130)
   {
    Outlier("General", 0.05);
    context="SR3, zone/square N 32, stratum 3 lower";
   };
   R_Date("CDTN 1054",2025,130)
   {
    Outlier("General", 0.05);
    context="SR3, zone/square Q 28, stratum 3";
   };
   R_Date("CDTN 1052",3165,170)
   {
    Outlier("General", 0.05);
    context="SR3, zone/square P 27, stratum 3";
   };
   R_Date("CDTN 1012",4300,210)
   {
    Outlier("General", 0.05);
    context="SR3, zone/square Q 27, stratum 3 middle";
   };
   R_Date("CDTN 1055",6020,300)
   {
    Outlier("General", 0.05);
    context="SR3, zone/square Q 25, stratum 3 t";
   };
   R_Date("CDTN 1011",6500,360)
   {
    Outlier("General", 0.05);
    context="SR3, zone/square Q 26, stratum 3 k";
   };
   R_Date("CDTN 1002",8400,300)
   {
    Outlier("General", 0.05);
    context="SR3, zone/square Q 26, stratum 3 base";
   };
   R_Date("CDTN 1001",8500,500)
   {
    Outlier("General", 0.05);
    context="SR3, zone/square R 25, stratum 3 q";
   };
   R_Date("GIF 4511",8990,100)
   {
    Outlier("General", 0.05);
    context="SR3, zone/square P 27, stratum 3 lower";
   };
   Interval("Duration 3");
  };
  Boundary("End 3/Start gap");
  Boundary("End gap/Start 1");
  Phase("stratum 1")
  {
   R_Date("CDTN 1059",4180,270)
   {
    Outlier("General", 0.05);
    context="SR3, zone/square O 29, stratum 1";
   };
   R_Date("CDTN 1064",1140,40)
   {
    Outlier("General", 0.05);
    context="SR3, zone/square O 33, stratum 1 upper";
   };
   R_Date("CDTN 1066",1475,80)
   {
    Outlier("General", 0.05);
    context="SR3, zone/square O 33, stratum 1 lower";
   };
   R_Date("CDTN 1065",2020,70)
   {
    Outlier("General", 0.05);
    context="SR3, zone/square O 33, stratum 1 lower";
   };
   R_Date("CDTN 1067",2770,70)
   {
    Outlier("General", 0.05);
    context="SR3, zone/square O 33, stratum 1 lower";
   };
   R_Date("CDTN 1010",810,40)
   {
    Outlier("General", 0.05);
    context="SR3, zone/square Q 26, stratum 1 upper";
   };
   R_Date("CDTN 1009",1200,130)
   {
    Outlier("General", 0.05);
    context="SR3, zone/square R 26, stratum 1 middle";
   };
   Interval("Duration 1");
  };
  Boundary("End 1");
 };
};
//R_Date("CDTN 1056",860,100)
//context="SR3, zone/square R 30, stratum 0";
```

### 2.2.10. Toca da Janela da Barra do Antonião

Toca da Janela is a rockshelter site located in Brazil (-8.802222, -42.433056), containing lithic and bone technology, as well as megafauna remains[135,136]. During excavations in 2013, six sedimentary units (C1-6) containing seven archaeological levels were found, with the oldest archaeological material found in C6a (basal, although not mentioned as a cultural occupation at the end of Layahe et al.[136]), C5a, C4a, and C3a-b (the latter containing Itaparica technology).

Bayesian modelling estimates the start of C4 and C3 at 19000-13565 cal BP (or 17310-14380 cal BP at 68.3% CI) and 14650-10850 cal BP (or 13790-11880 cal BP at 68.3%), respectively, with no outliers (Supplementary Figure 54 and OxCal code). The latter, associated with Itaparica technology, dates to a range of 15501-12995 cal BP (at 68.3%). Given that the sequence is dominated by imprecise OSL measurements, however, these estimates require careful interpretation.

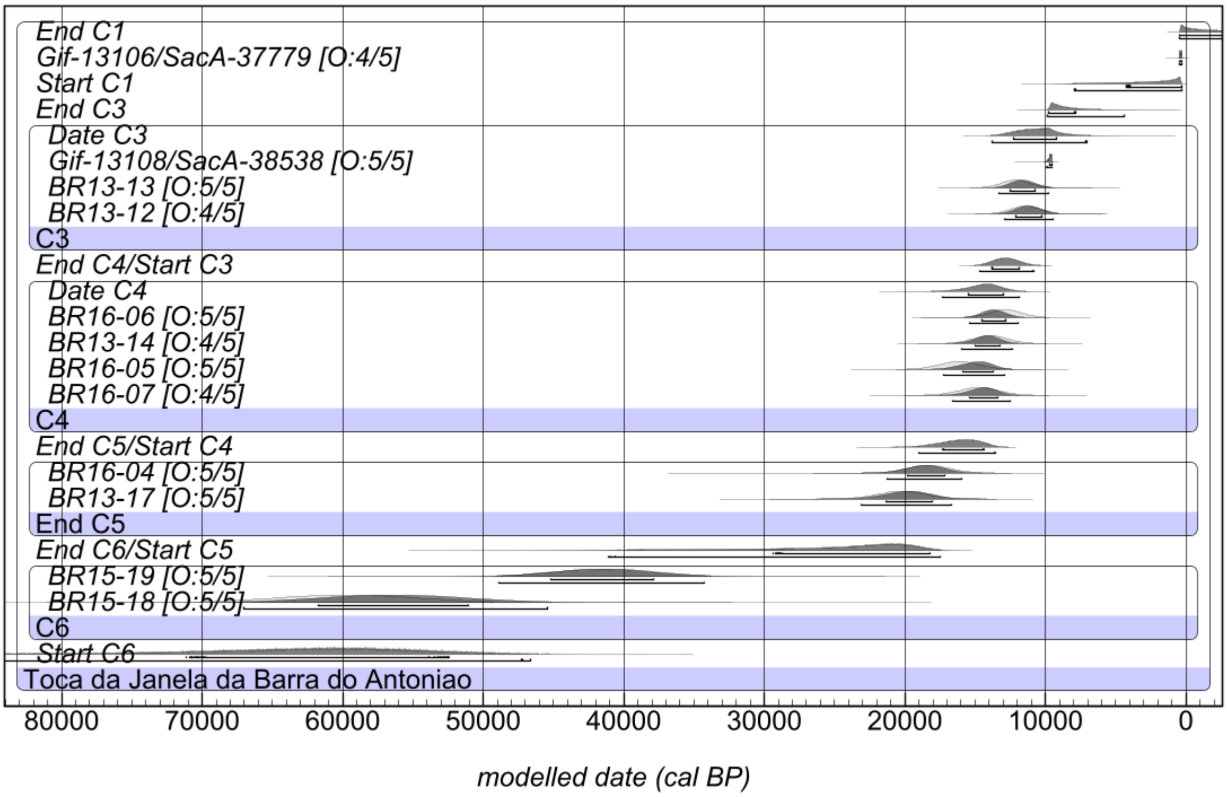


**Supplementary Figure 54.** Bayesian age model for Toca da Janela da Barra do Antonião. Brackets beneath each age estimate show 68.3% and 95.4% CI. Outlier analysis output is noted as ‘O:posterior probability/prior probability’.

```
Plot()
{
 Curve("IntCal20","intcal20.14c");
 Curve("SHCal20","shcal20.14c");
 Mix_Curve ("Mixed","IntCal20","SHCal20",U(0,100));
 Outlier_Model("General",T(5),U(0,4),"t");
 Sequence("Toca da Janela da Barra do Antoniao")
 {
  Boundary("Start C6");
  Phase("C6")
  {
   Date("BR15-18", N(2015-59800,5000))
   {
    context="C6 inf";
    Outlier("General", 0.05);
   };
   Date("BR15-19", N(2015-41300,3600))
   {
    context="C6 sup";
    Outlier("General", 0.05);
   };
   Interval("Duration C6");
  };
  Boundary("End C6/Start C5");
  Phase("End C5")
  {
   Date("BR13-17", N(2013-20300,1700))
   {
    context="C5";
    Outlier("General", 0.05);
   };
   Date("BR16-04", N(2016-18400,1500))
   {
    context="C5";
    Outlier("General", 0.05);
   };
   Interval("Duration C5");
  };
  Boundary("End C5/Start C4");
  Phase("C4")
  {
   Date("BR16-07", N(2016-14800,1400))
   {
    context="C4 sup";
    Outlier("General", 0.05);
   };
   Date("BR16-05", N(2016-16200,1400))
   {
    context="C4 inf";
    Outlier("General", 0.05);
   };
   Date("BR13-14", N(2013-14000,1200))
   {
    context="C4";
    Outlier("General", 0.05);
   };
   Date("BR16-06", N(2016-12900,1100))
   {
    context="C4";
    Outlier("General", 0.05);
   };
   Interval("Duration C4");
   Date("Date C4");
  };
  Boundary("End C4/Start C3");
  Phase("C3")
  {
   Date("BR13-12", N(2013-11500,1000))
   {
    context="C3 sup";
    Outlier("General", 0.05);
   };
   Date("BR13-13", N(2013-12200,1000))
   {
    context="C3 inf";
    Outlier("General", 0.05);
   };
   R_Date("Gif-13108/SacA-38538",8730,40)
   {
    context="C2b/C3a, Itaparica horizon";
    Outlier("General", 0.05);
   };
   Interval("Duration C3");
   Date("Date C3");
  };
  Boundary("End C3");
  Boundary("Start C1");
  R_Date("Gif-13106/SacA-37779",330,30)
  {
   context="C1";
   Outlier("General", 0.05);
  };
  Boundary("End C1");
 };
};
```

## 2.2.11. Toca do Sítio do Meio

Toca do Sítio do Meio is a rockshelter site located in Brazil (-8.839167, -42.563889), containing lithic technology and hearths[98,102,137–139]. There are two main units (lower and upper) separated by a rock-

fall layer (see Fig. 3 in Boëda et al.[137]). In Aimola et al.[98], the authors define four stratigraphic units (I-V, top to bottom) within sector 2, with IV representing 'fallen heterometric sandstone blocks'.

Excavation and documentation methods between field programs at Toca do Sítio de Meio[98] have differed, making the correlation of radiocarbon dates, within their archaeological context, a challenge. Therefore, a simple Bayesian model was created with two phases corresponding to the main lower and upper units. A number of dates were placed within the upper unit but this assignment is uncertain. These are coloured in purple and seem to be congruent with the chronology (no outliers). This model estimates the start of the upper unit at 18580-16245 cal BP (Itaparica), with no outliers (see Supplementary Figure 55 and OxCal code). The age range for this component is estimated at 17240-7865 cal BP, overlapping the ACR-YD period.

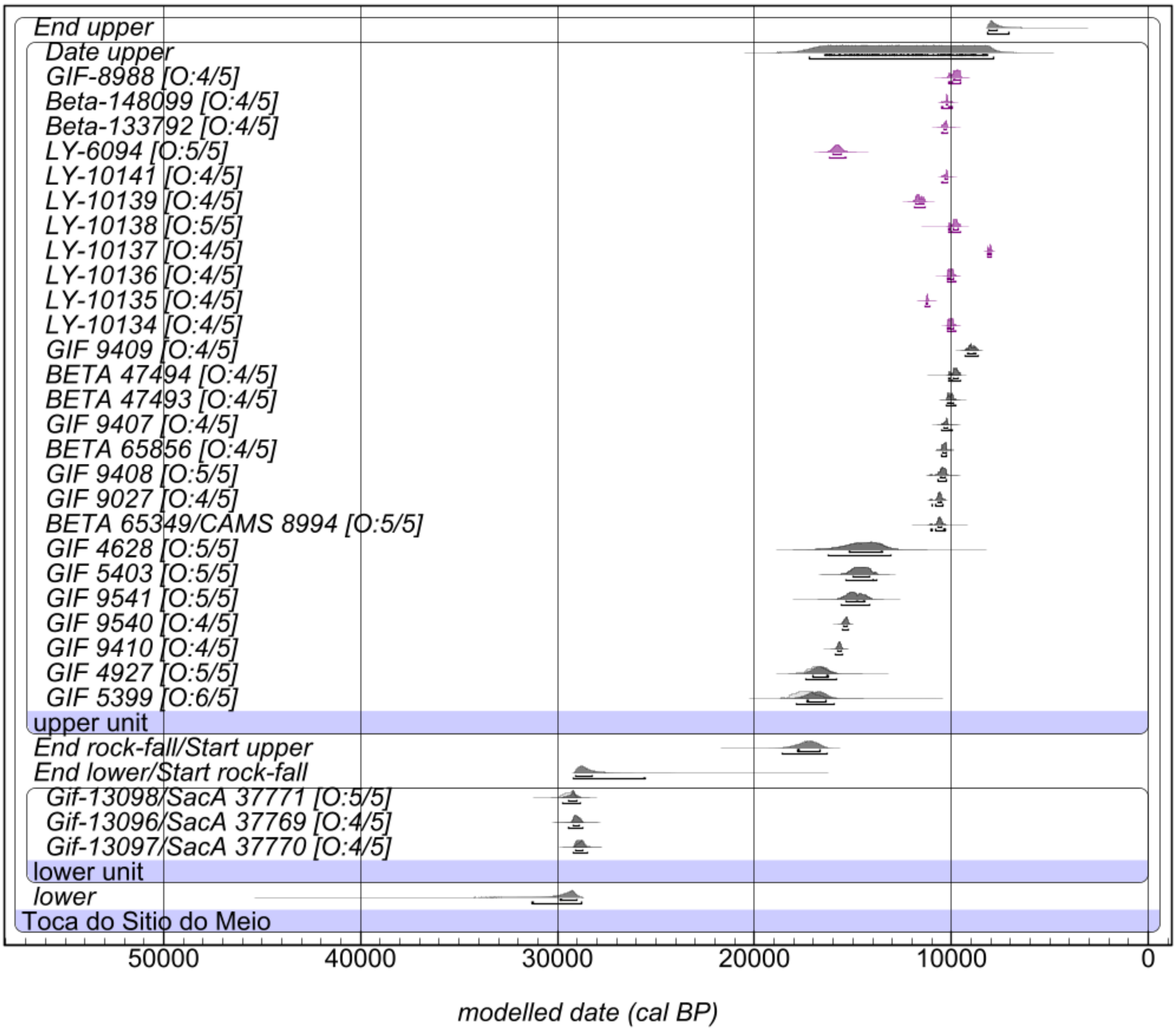


**Supplementary Figure 55.** Bayesian age model for Toca do Sítio do Meio. Purple distributions denote ages for which their stratigraphic assignment is uncertain (although none of them are identified as major outliers). Brackets beneath each age estimate show 68.3% and 95.4% CI. Outlier analysis output is noted as 'O:posterior probability/prior probability'.

```
Plot()
{
 Curve("IntCal20","intcal20.14c");
 Curve("SHCal20","shcal20.14c");
 Mix_Curve ("Mixed","IntCal20","SHCal20",U(0,100));
 Outlier_Model("General",T(5),U(0,4),"t");
 Sequence("Toca do Sitio do Meio")
 {
  Boundary("lower");
  Phase("lower unit")
  {
   R_Date("Gif-13097/SacA 37770",24520,170)
   {
    Outlier("General", 0.05);
    context=lower unit;
   };
   R_Date("Gif-13096/SacA 37769",24850,190)
   {
    Outlier("General", 0.05);
    context=lower unit;
   };
   R_Date("Gif-13098/SacA 37771",25110,180)
   {
    Outlier("General", 0.05);
    context=lower unit;
   };
   Interval("Duration lower");
  };
  Boundary("End lower/Start rock-fall");
  Interval("Duration rock-fall");
  Boundary("End rock-fall/Start upper");
  Phase("upper unit")
  {
   R_Date("GIF 5399",14300,400)
   {
    Outlier("General", 0.05);
    context=upper unit, niveau XVIII;
   };
   R_Date("GIF 4927",13900,300)
   {
    Outlier("General", 0.05);
    context=upper unit, niveau VI;
   };
   R_Date("GIF 9410",13100,50)
   {
    Outlier("General", 0.05);
    context=upper unit, foyer;
   };
   R_Date("GIF 9540",12870,40)
   {
    Outlier("General", 0.05);
    context=upper unit, foyer;
   };
   R_Date("GIF 9541",12640,210)
   {
    Outlier("General", 0.05);
    context=upper unit, foyer;
   };
   R_Date("GIF 5403",12440,230)
   {
    Outlier("General", 0.05);
    context=upper unit, niveau XV;
   };
   R_Date("GIF 4628",12200,600)
   {
    Outlier("General", 0.05);
    context=upper unit, niveau V;
   };
   R_Date("BETA 65349/CAMS 8994",9400,70)
   {
    Outlier("General", 0.05);
    context=upper unit, foyer berge ruisseau;
   };
   R_Date("GIF 9027",9400,60)
   {
    Outlier("General", 0.05);
    context=upper unit, foyer;
   };
   R_Date("GIF 9408",9270,100)
   {
    Outlier("General", 0.05);
    context=fupper unit, foyer sect. 2 niveau 16;
   };
   R_Date("BETA 65856",9200,60)
   {
    Outlier("General", 0.05);
    context=upper unit,;
   };
   R_Date("GIF 9407",9110,80)
   {
    Outlier("General", 0.05);
    context=upper unit, foyer niveau 15;
   };
   R_Date("BETA 47493",8960,70)
   {
    Outlier("General", 0.05);
    context=upper unit;
   };
   R_Date("BETA 47494",8800,60)
   {
    Outlier("General", 0.05);
    context=upper unit, ultime chute de blocs;
   };
   R_Date("GIF 9409",8100,90)
   {
    Outlier("General", 0.05);
    context=upper unit, foyer;
   };
   R_Date("LY-10134",8920,50)
   {
    color="purple";
    Outlier("General", 0.05);
    context=sector 3;
   };
   R_Date("LY-10135",9826,55)
   {
    color="purple";
    Outlier("General", 0.05);
    context=sector 3;
   };
   R_Date("LY-10136",8925,55)
   {
    color="purple";
    Outlier("General", 0.05);
    context=sector 3;
   };
   R_Date("LY-10137",7240,45)
   {
    color="purple";
    Outlier("General", 0.05);
    context=sector 3;
   };
   R_Date("LY-10138",8804,53)
   {
    color="purple";
    Outlier("General", 0.05);
    context=sector 3;
   };
   R_Date("LY-10139",10110,55)
   {
    color="purple";
    Outlier("General", 0.05);
    context=sector 3;
   };
   R_Date("LY-10141",9110,60)
   {
    color="purple";
    Outlier("General", 0.05);
    context=sector 4;
   };
   R_Date("LY-6094",13180,130)
   {
    color="purple";
    Outlier("General", 0.05);
    context=sector 2;
   };
   R_Date("Beta-133792",9150,60)
   {
    color="purple";
    Outlier("General", 0.05);
    context=sector 4;
   };
   R_Date("Beta-148099",9080,60)
   {
    color="purple";
    Outlier("General", 0.05);
    context=sector 3;
   };
   R_Date("GIF-8988",8760,100)
   {
    color="purple";
    Outlier("General", 0.05);
    context=sector 2;
   };
   Date("Date upper");
   Interval("Duration upper");
  };
  Boundary("End upper");
 };
};
//Noted by Aimola et al., 2014 as probably natural
//R_Date("Gif-9542/LSM-9542",25170,140)
//context=lower unit
//R_Date("BETA 65350",20280,450)
//context=upper unit
```

## 2.2.12. Toca do Garrincho

Toca do Garrincho is a cave site located in Brazil (-8.930833, -42.614444), containing human and faunal remains[140–142]. There are two stratigraphic units, A (Holocene) and B (Pleistocene), which are separated by a stalagmite. A human incisor and molar (in alveolar bone) were found during excavations within trench 1 (at different levels), below the stalagmite [dated through associated charcoals to 10020 ± 290 BP (GIF-9335)]. The teeth were dated by Beta Analytic to 12210 ± 40 BP (Beta-136204), although no collagen was obtained. The date is noted as having been obtained from carbon within the acid washes, which would yield an unreliable measurement that is, most likely, a minimum age. No modelling was attempted given sample size and reliability issues. As such, this site is not included in the Bayesian analysis.

2.2.13. Vale da Pedra Furada

Vale da Pedra Furada is an open-air site in Brazil (approximately -8.85, -42.55), containing lithic technology[137,143–146]. There are fifteen distinct archaeological horizons (see Fig. 2 in Boëda et al.[145]), with C4 being culturally sterile. Deposits C7 and C3 contain most of the lithic material found at the site[144], and the oldest reported archaeological levels are C13 through to C7γ. In 2021, Boëda et al. made amendments to the nomenclature system.

Following the culturally sterile level C4, Bayesian modelling estimates the start of C3 and C2 at 20265-16090 cal BP and 16245-8740 cal BP (or 16230-13420 cal BP at 68.3% CI), respectively (see Supplementary Figure 56 and OxCal code). The age range for C3 is estimated at 19320-11700 cal BP, overlapping the ACR-YD period. There are three major outliers in the sequence, all from preceding levels. Radiocarbon measurement Gif-13101/SacA-37774 was excluded as contextual information was not identified.

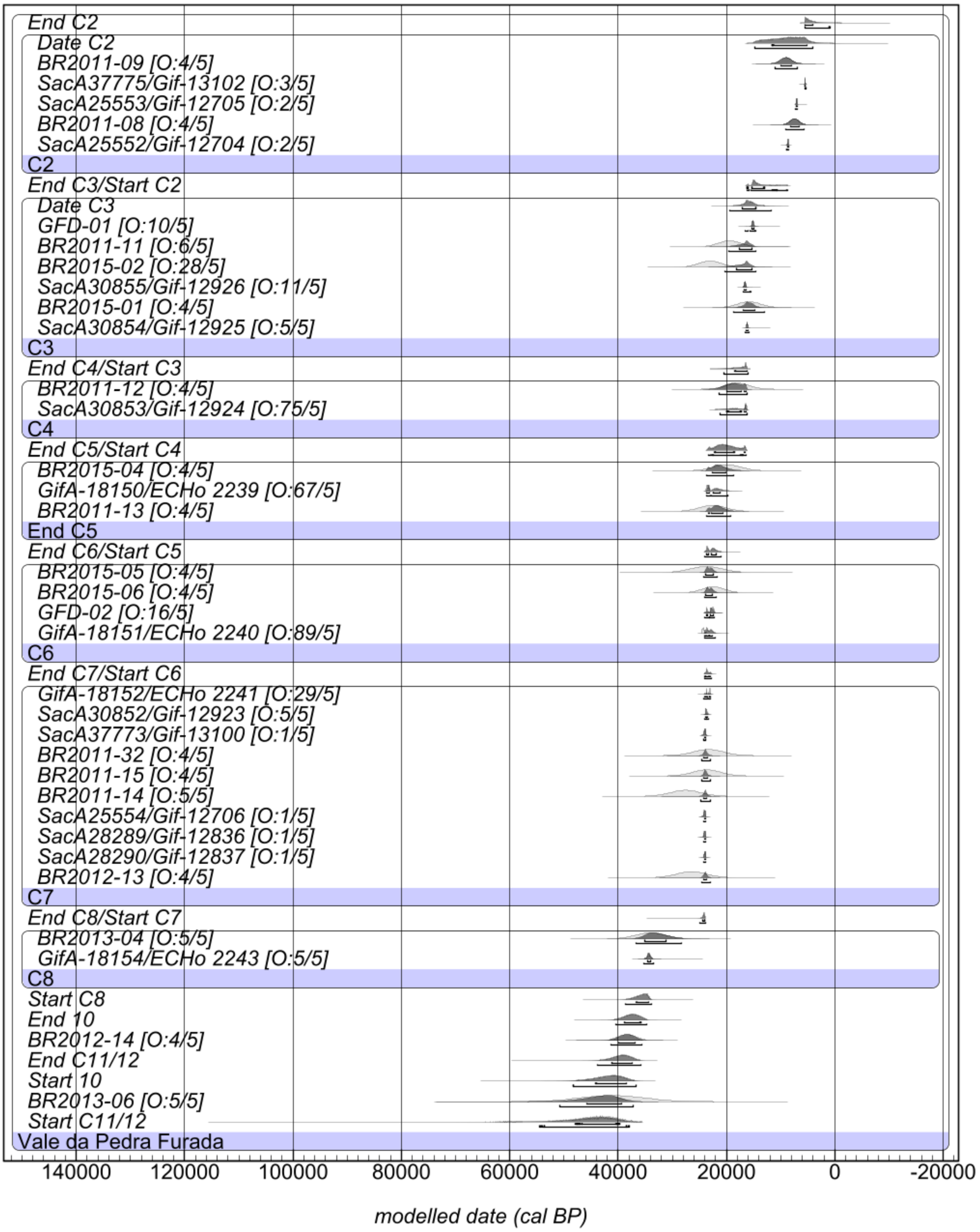


**Supplementary Figure 56.** Bayesian age model for Vale da Pedra Furada. Brackets beneath each age estimate show 68.3% and 95.4% CI. Outlier analysis output is noted as ‘O:posterior probability/prior probability’.

```
Plot()
{
Curve("IntCal20","intcal20.14c");
Curve("SHCal20","shcal20.14c");
Mix_Curve ("Mixed","IntCal20","SHCal20",U(0,100));
Outlier_Model("General",T(5),U(0,4),"t");
Sequence("Vale da Pedra Furada")
{
 Boundary("Start C11/12");
 //collection date assumed from sample name
 Date("BR2013-06", N(2013-41200, 5900))
 {
  Outlier("General", 0.05);
  context="C11/12";
 };
 Boundary("End C11/12");
 Boundary("Start 10");
 //date presented as before AD 2000
 Date("BR2012-14", N(2000-38400, 1700))
 {
  Outlier("General", 0.05);
  context="C10";
 };
 Boundary("End 10");
 Boundary("Start C8");
 Phase("C8")
 {
  R_Date("GifA-18154/ECHo 2243",29880,350)
  {
   Outlier("General", 0.05);
   context="C8";
  };
  //collection date assumed from sample name
  Date("BR2013-04", N(2013-34100, 2700))
  {
   Outlier("General", 0.05);
   context="C8";
  };
  Interval("Duration C8");
 };
 Boundary("End C8/Start C7");
 Phase("C7")
 {
  //date presented as before AD 2000
  Date("BR2012-13", N(2000-26500, 2800))
  {
   Outlier("General", 0.05);
   context="C7b";
  };
  R_Date("SacA28290/Gif-12837",19970,100)
  {
   Outlier("General", 0.05);
   context="C7α";
  };
  R_Date("SacA28289/Gif-12836",20070,100)
  {
   Outlier("General", 0.05);
   context="C7α";
  };
  R_Date("SacA25554/Gif-12706",20090,120)
  {
   Outlier("General", 0.05);
   context="C7α";
  };
  //date presented as before AD 2000
  Date("BR2000-14", N(2011-27600, 2800))
  {
   Outlier("General", 0.05);
   context="C7α";
  };
  //date presented as before AD 2000
  Date("BR2011-15", N(2000-23700, 2600))
  {
   Outlier("General", 0.05);
   context="C7α";
  };
  //date presented as before AD 2000
  Date("BR2011-32", N(2000-23400, 2800))
  {
   Outlier("General", 0.05);
   context="C7α";
  };
  R_Date("SacA37773/Gif-13100",19990,110)
  {
   Outlier("General", 0.05);
   context="C7β";
  };
  R_Date("SacA30852/Gif-12923",19700,100)
  {
   Outlier("General", 0.05);
  };
  R_Date("GifA-18152/ECHo 2241",19180,120)
  {
   Outlier("General", 0.05);
   context="C7γα";
  };
  Interval("Duration C7");
 };
 Boundary("End C7/Start C6");
 Phase("C6")
 {
  R_Date("GifA-18151/ECHo 2240",20250,120)
  {
   Outlier("General", 0.05);
   context="C5/C6";
  };
  R_Date("GFD-02",18660,260)
  {
   Outlier("General", 0.05);
  };
  //collection date assumed from sample name
  Date("BR2015-06", N(2015-22500, 2000))
  {
   Outlier("General", 0.05);
   context="C6 base";
  };
  //collection date assumed from sample name
  Date("BR2015-05", N(2015-23800, 2900))
  {
   Outlier("General", 0.05);
   context="C6 top";
  };
  Interval("Duration C6");
 };
 Boundary("End C6/Start C5");
 Phase("End C5")
 {
  //date presented as before AD 2000
  Date("BR2011-13", N(2000-22600, 2400))
  {
   Outlier("General", 0.05);
   context="C5";
  };
  R_Date("GifA-18150/ECHo 2239",19500,110)
  {
   Outlier("General", 0.05);
   context="C5";
  };
  //collection date assumed from sample name
  Date("BR2015-04", N(2015-20000, 2500))
  {
   Outlier("General", 0.05);
   context="C5";
  };
  Interval("Duration C5");
 };
 Boundary("End C5/Start C4");
 Phase("C4")
 {
  R_Date("SacA30853/Gif-12924",13590,60)
  {
   Outlier("General", 0.05);
   context="C4";
  };
  //date presented as before AD 2000
  Date("BR2011-12", N(2000-18000, 2200))
  {
   Outlier("General", 0.05);
   context="C4";
  };
  Interval("Duration C4");
 };
 Boundary("End C4/Start C3");
 Phase("C3")
 {
  R_Date("SacA30854/Gif-12925",13460,50)
  {
   Outlier("General", 0.05);
   context="C3α";
  };
  //collection date assumed from sample name
  Date("BR2015-01", N(2015-15900, 2200))
  {
   Outlier("General", 0.05);
   context="C3α";
  };
  R_Date("SacA30855/Gif-12926",13740,60)
  {
   Outlier("General", 0.05);
   context="C3β";
  };
  //collection date assumed from sample name
  Date("BR2015-02", N(2015-23100, 2100))
  {
   Outlier("General", 0.05);
   context="C3ν";
  };
  //date presented as before AD 2000
  Date("BR2011-11", N(2000-19500, 2000))
  {
   Outlier("General", 0.05);
   context="C3σ/ε";
  };
  R_Date("GFD-01",12700,90)
  {
   Outlier("General", 0.05);
   context="C2/C3";
  };
  Interval("Duration C3");
  Date("Date C3");
 };
 Boundary("End C3/Start C2");
 Phase("C2")
 {
  R_Date("SacA25552/Gif-12704",7875,40)
  {
   Outlier("General", 0.05);
   context="C2a";
  };
  //date presented as before AD 2000
  Date("BR2011-08", N(2000-7500, 800))
  {
   Outlier("General", 0.05);
   context="C2a";
  };
  R_Date("SacA25553/Gif-12705",6190,35)
  {
   Outlier("General", 0.05);
   context="C2a/b";
  };
  R_Date("SacA37775/Gif-13102",4790,30)
  {
   Outlier("General", 0.05);
   context="C2b";
  };
  //date presented as before AD 2000
  Date("BR2011-09", N(2000-9100, 1000))
  {
   Outlier("General", 0.05);
   context="C2b";
  };
  Date("Date C2");
  Interval("Duration C2");
 };
 Boundary("End C2");
};
Difference("Difference C4 and C2","End C4/Start C3","End C3/Start C2");
Sequence()
{
 Boundary("=End C4/Start C3");
 Interval("Duration C3 and C2");
 Date("Date C3 and C2");
 Boundary("=End C3/Start C2");
};
};
```

*2.3. Bolivia*

2.3.1. Cueva Bautista (AL03)

Cueva Bautista (AL03) is a high-altitude (3,933 masl) rockshelter located in Bolivia (-21.338, -67.574), containing lithic technology (unifacial) and hearths[147–149]. There are 17 stratigraphic levels (A-Q), with P showing the earliest signs of human activity (see Fig. 3. in Capriles et al.[147]).

Bayesian modelling estimates the start of level P at 13010-12735 cal BP and level O at 12770-12410 cal BP, with no outliers (see Supplementary Figure 57 and OxCal code). Following the late Pleistocene/early Holocene occupation, there is a late Archaic horizon dated to ~4000 cal BP[147].

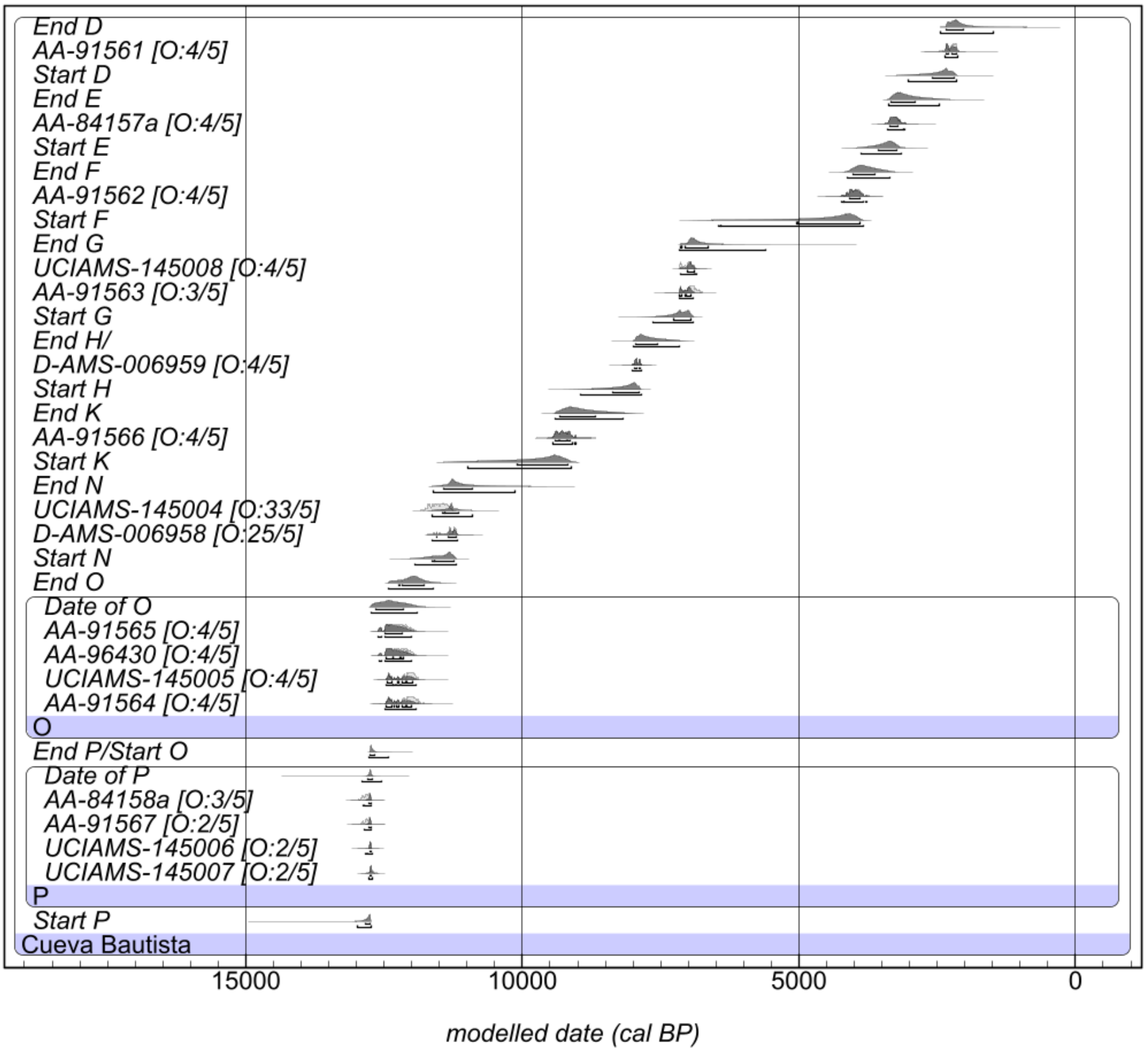


**Supplementary Figure 57.** Bayesian age model for Cueva Bautista. Brackets beneath each age estimate show 68.3% and 95.4% CI. Outlier analysis output is noted as ‘O:posterior probability/prior probability’.

```
Plot()
{
 Outlier_Model("General",T(5),U(0,4),"t");
 Curve("SHCal20","shcal20.14c");
 Sequence("Cueva Bautista")
 {
  Boundary("Start P");
  Phase("P")
  {
   R_Date("UCIAMS-145007",10800,45)
   {
    Outlier("General", 0.05);
    context="level P, unit 16";
    material="faunal bone";
   };
   R_Date("UCIAMS-145006",10850,45)
   {
    Outlier("General", 0.05);
    context="level P, unit 10";
    material="tooth";
   };
   R_Date("AA-91567",10904,61)
   {
    Outlier("General", 0.05);
    context="level P, unit 8";
    material="charcoal";
   };
   R_Date("AA-84158a",10917,69)
   {
    Outlier("General", 0.05);
    context="level P, unit 9";
    material="charcoal";
   };
   Date("Date of P");
   Interval("Duration of P");
  };
  Boundary("End P/Start O");
  Phase("O")
  {
   R_Date("AA-91564",10336,63)
   {
    Outlier("General", 0.05);
    context="level O, unit 13";
    material="charcoal";
   };
   R_Date("UCIAMS-145005",10340,45)
   {
    Outlier("General", 0.05);
    context="level O, unit 10";
    material="faunal bone";
   };
   R_Date("AA-96430",10412,60)
   {
    Outlier("General", 0.05);
    context="level O, unit 4";
    material="charcoal";
   };
   R_Date("AA-91565",10433,67)
   {
    Outlier("General", 0.05);
    context="level O, unit 10";
    material="charcoal";
   };
   Date("Date of O");
   Interval("Duration of O");
  };
  Boundary("End O");
  Boundary("Start N");
  R_Date("D-AMS-006958",9850,44)
  {
   Outlier("General", 0.05);
   context="level N, unit 13";
   material="soil (bulk)";
  };
  R_Date("UCIAMS-145004",10070,45)
  {
   Outlier("General", 0.05);
   context="level N, unit 14";
   material="faunal bone";
  };
  Boundary("End N");
  Boundary("Start K");
  R_Date("AA-91566",8311,54)
  {
   Outlier("General", 0.05);
   context=level K, unit 8;
   material="wood";
  };
  Boundary("End K");
  Boundary("Start H");
  R_Date("D-AMS-006959",7139,33)
  {
   Outlier("General", 0.05);
   context="level H, unit 13";
   material="soil (bulk)";
  };
  Boundary("End H/");
  Boundary("Start G");
  R_Date("AA-91563",6122,56)
  {
   Outlier("General", 0.05);
   context="level G, unit 3";
   material="charcoal";
  };
  R_Date("UCIAMS-145008",6160,30)
  {
   Outlier("General", 0.05);
   context="level G, unit 4";
   material="faunal bone";
  };
  Boundary("End G");
  Boundary("Start F");
  R_Date("AA-91562",3690,64)
  {
   Outlier("General", 0.05);
   context="level F, unit 18";
   material="charcoal";
  };
  Boundary("End F");
  Boundary("Start E");
  R_Date("AA-84157a",3111,54)
  {
   Outlier("General", 0.05);
   context="level E, unit 9";
   material="leather";
  };
  Boundary("End E");
  Boundary("Start D");
  R_Date("AA-91561",2307,42)
  {
   Outlier("General", 0.05);
   context="level D, unit 13";
   material="charcoal";
  };
  Boundary("End D");
 };
 Difference("Difference P and O","Start P","End P/Start O");
 Sequence()
 {
  Boundary("=Start P");
  Interval("Duration P and O");
  Date("Date P and O");
  Boundary("=End O");
 };
};
```

*2.4. Chile*

2.4.1. La Cueva 1 del Lago Sofía (or Lago Sofía 1)

La Cueva 1 del Lago Sofia (CLS 1) is a cave site located in Chile (-51.53, -72.53), containing lithic technology, a hearth, and extinct faunal remains (*Hippidion saldiasi* and *Mylodon darwinii*)[150,151]. There are three stratigraphic levels, including strata 1a-c, 2a-b, and 3 (see Fig 6. in Prieto[150]). The Paleoindian component is found in stratum 2a, with the hearth in 2b.

Bayesian modelling estimates the start of Fell I at 14360-13325 cal BP, with no outliers (see Supplementary Figure 58 and OxCal code). Following Fell I, evidence for cultural activity is found in a cremation event(s), dating to ~4200 cal BP (mean).

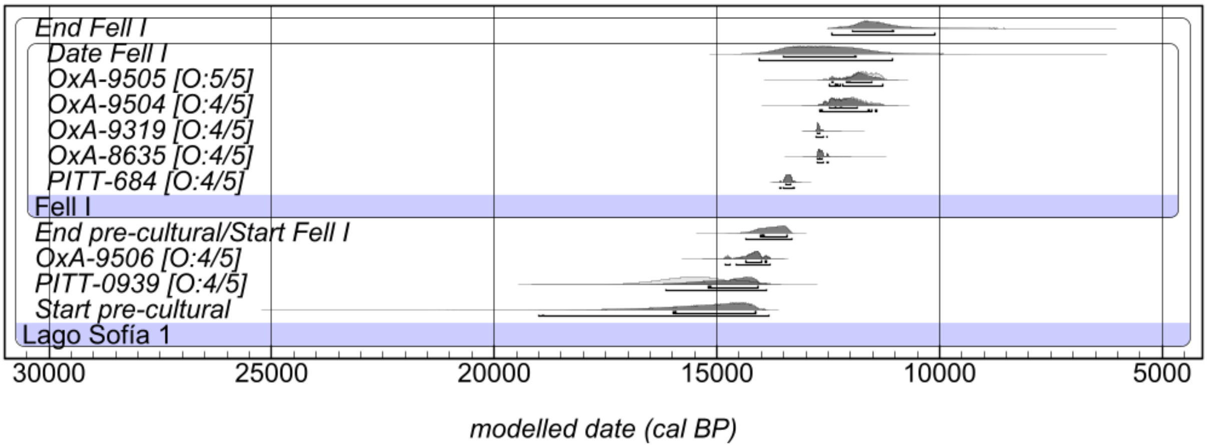


**Supplementary Figure 58.** Bayesian age model for la Cueva 1 del Lago Sofía. Brackets beneath each age estimate show 68.3% and 95.4% CI. Outlier analysis output is noted as 'O:posterior probability/prior probability'.

```
Plot()
{
 Curve("SHCal20","shcal20.14c");
 Outlier_Model("General",T(5),U(0,4),"t");
 Sequence("Lago Sofia 1")
 {
  Boundary("Start pre-cultural");
  R_Date("PITT-0939",12990,490)
  {
   Outlier("General", 0.05);
  };
  R_Date("OxA-9506",12250,110)
  {
   Outlier("General", 0.05);
  };
  Boundary("End pre-cultural/Start Fell I");
  Phase("Fell I")
  {
   R_Date("PITT-684",11570,60)
   {
    Outlier("General", 0.05);
   };
   R_Date("OxA-8635",10710,70)
   {
    Outlier("General", 0.05);
   };
   R_Date("OxA-9319",10780,60)
   {
    Outlier("General", 0.05);
   };
   R_Date("OxA-9504",10310,160)
   {
    Outlier("General", 0.05);
   };
   R_Date("OxA-9505",10140,120)
   {
    Outlier("General", 0.05);
   };
   Date("Date Fell I");
   Interval("Duration Fell I");
  };
  Boundary("End Fell I");
 };
};
```

2.4.2. Cueva de Fell

Cueva de Fell is a rockshelter located in Chile (-52.044444, -70.056389), containing lithic technology, hearths and extinct faunal remains (*Hippidion saldiasi* and *Mylodon darwinii*)[14,152–158]. This is the type-site for Fishtail projectile points. Cueva de Fell has been excavated by both professionals and amateurs multiple times for over 80 years and so different categorisation systems for the stratigraphy apply[153]. According to Bird[14], however, there are five archaeological layers (see Fig. 4 in Martin[153]), with sterile debris from roof-fall separating the most basal stratum (V; Pleistocene; 'Period I') from overlying, Holocene layers ('Period II'). Later, layers 18-20 were attributed to Bird's Period I, whilst 13-17 were correlated with Period II[153].

A single-phase Bayesian model estimates the start of Period I at 13755-12710 cal BP (see Supplementary Figure 59 and OxCal code). I-5144 (9030 ± 230 BP) from Period II was excluded from the model as there is uncertainty regarding its reliability[153].

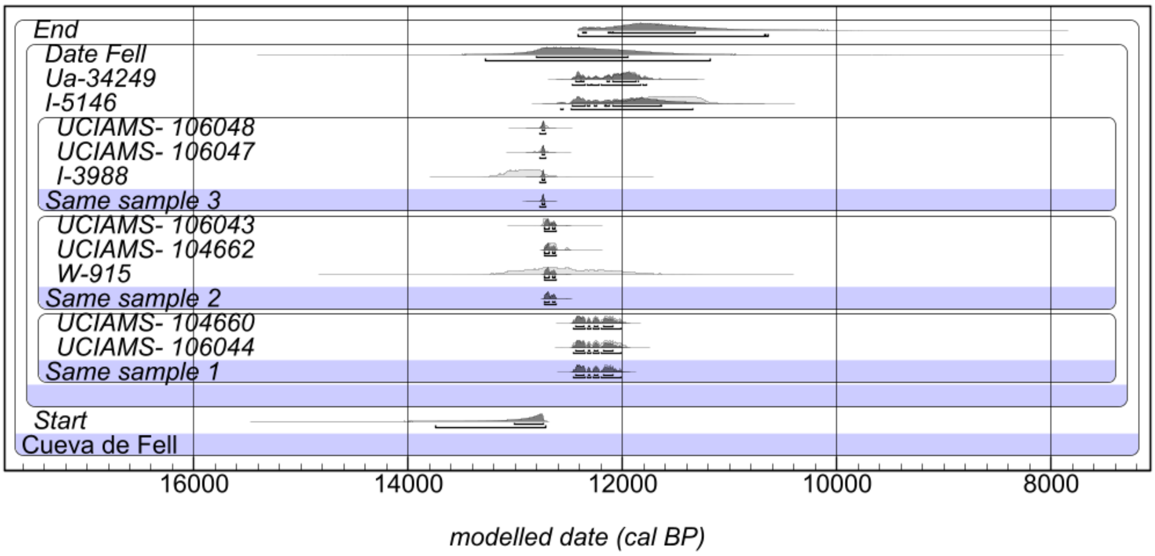


**Supplementary Figure 59.** Bayesian age model for Cueva de Fell. Brackets beneath each age estimate show 68.3% and 95.4% CI.

```
Plot()
{
 Curve("SHCal20","shcal20.14c");
 Sequence("Cueva de Fell")
 {
  Boundary("Start");
  Phase("")
  {
   Combine("Same sample 1")
   {
    R_Date("UCIAMS- 106044",10395,40)
    {
     context="Layer 18, sensu Waters et al. 2015";
     material="charcoal";
    };
    R_Date("UCIAMS- 104660",10395,30)
    {
     context="Layer 18, sensu Waters et al. 2015";
     material="charcoal";
    };
   };
   Combine("Same sample 2")
   {
    R_Date("W-915",10720,300)
    {
     context="Fell 1";
     material="charcoal";
    };
    R_Date("UCIAMS- 104662",10675,40)
    {
     context="Layer 19, sensu Waters et al. 2015";
     material="charcoal";
    };
    R_Date("UCIAMS- 106043",10760,60)
    {
     context="Layer 19, sensu Waters et al. 2015";
     material="charcoal";
    };
   };
   Combine("Same sample 3")
   {
    R_Date("I-3988",11000,170)
    {
     context="Fell 1";
     material="charcoal";
    };
    R_Date("UCIAMS- 106047",10835,50)
    {
     context="Layer 20, sensu Waters et al. 2015";
     material="charcoal";
    };
    R_Date("UCIAMS- 106048",10810,50)
    {
     context="Layer 20, sensu Waters et al. 2015";
     material="humic acids from charcoal";
    };
   };
   R_Date("I-5146",10080,160)
   {
    context="fin periodo I inicio período II, Layer 17 or 18";
    material="charcoal";
   };
   R_Date("Ua-34249",10295,65)
   {
    context="Layer 17";
    material="bone";
   };
   Date("Date Fell");
  };
  Boundary("End");
 };
};
```

## 2.4.3. Cueva de la Vieja

Cueva de la Vieja is a cave site located in Chile (-45.2742, -71.54) containing lithic technology and faunal remains[159]. There are five stratigraphic units (SUs) at the site as defined by sedimentological evidence (see Fig. 4 in Méndez et al.[159], with archaeological evidence in SU1 and the more basal SU2.

Bayesian modelling estimates the start of SU-3 contact at 14825-11350 cal BP (see Supplementary Figure 60 and OxCal code). UGAMS-19835 was entered as an outlier at 100% prior probability, as retrieved from a rodent burrow. Apart from this date, there are X major outliers—Beta-317159 and -317158 (both likely underestimates in SU3-2 contact)—which is consistent with evidence of vertical

mixing. Following SU2 (SU3 contact), evidence for cultural activity is found in SU2, which is estimated to have started at 7400-5705 cal BP.

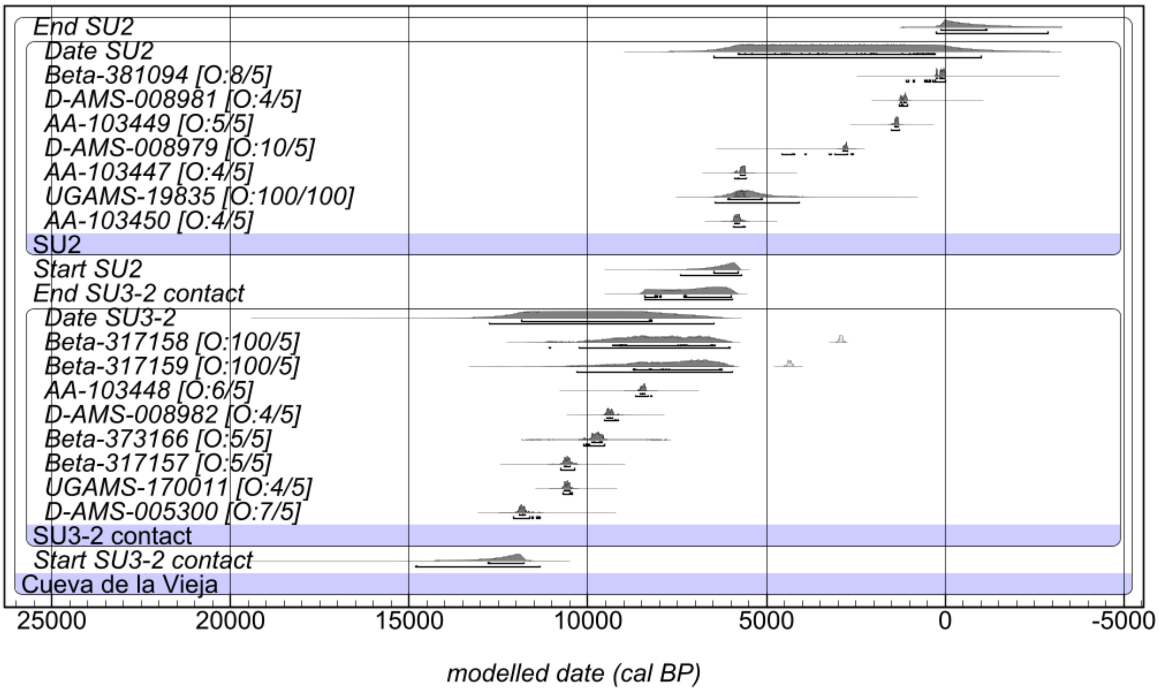


**Supplementary Figure 60.** Bayesian age model for Cueva de la Vieja. Brackets beneath each age estimate show 68.3% and 95.4% CI. Outlier analysis output is noted as ‘O:posterior probability/prior probability’.

```
Plot()
{
 Curve("SHCal20","shcal20.14c");
 Outlier_Model("General",T(5),U(0,4),"t");
 Sequence("Cueva de la Vieja")
 {
  Boundary("Start SU3-2 contact");
  Phase("SU3-2 contact")
  {
   R_Date("D-AMS-005300",10226,43)
   {
    Outlier("General",0.05);
    context="SU2 (contact SU3)";
   };
   R_Date("UGAMS-170011",9400,25)
   {
    Outlier("General",0.05);
    context="SU2 (contact SU3)";
   };
   R_Date("Beta-317157",9400,40)
   {
    Outlier("General",0.05);
    context="SU2 (contact SU3)";
   };
   R_Date("Beta-373166",8790,50)
   {
    Outlier("General",0.05);
    context="SU2 (contact SU3)";
   };
   R_Date("D-AMS-008982",8413,50)
   {
    Outlier("General",0.05);
    context="SU2 (contact SU3)";
   };
   R_Date("AA-103448",7693,54)
   {
    Outlier("General",0.05);
    context="SU3 (contact SU2)";
   };
   R_Date("Beta-317159",3960,30)
   {
    Outlier("General",0.05);
    context="SU2 (contact SU3)";
   };
   R_Date("Beta-317158",2850,30)
   {
    Outlier("General",0.05);
    context="SU2 (contact SU3)";
   };
   Interval("Duration SU3-2");
   Date("Date SU3-2");
  };
  Boundary("End SU3-2 contact");
  Boundary("Start SU2");
  Phase("SU2")
  {
   R_Date("AA-103450",5117,47)
   {
    Outlier("General",0.05);
    context="SU2";
   };
   //retrieved from rodent burrow
   R_Date("UGAMS-19835",5030,25)
   {
    Outlier("General",1);
    context="SU2";
   };
   R_Date("AA-103447",4977,49)
   {
    Outlier("General",0.05);
    context="SU2";
   };
   R_Date("D-AMS-008979",2706,26)
   {
    Outlier("General",0.05);
    context="SU2";
   };
   R_Date("AA-103449",1521,38)
   {
    Outlier("General",0.05);
    context="SU2";
   };
   R_Date("D-AMS-008981",1282,24)
   {
    Outlier("General",0.05);
    context="SU2";
   };
   R_Date("Beta-381094",130,30)
   {
    Outlier("General",0.05);
    context="SU2";
   };
   Date("Date SU2");
  };
  Boundary("End SU2");
 };
};
```

## 2.4.4. Cueva del Medio

Cueva del Medio is a cave site located in Chile (-51.576, -72.583), containing lithic technology and extinct faunal remains (*Hippidion saldiasi* and *Mylodon*)[160–165]. As defined by Nami, there are two

archaeological components labelled as Fell, Magallanes or Bird 'I' and "III', with the former being the oldest. Technology at Bird III shares characteristics with that found at the Pali Aike and Fell sites in Chile[14]. A Fishtail projectile point was found in close association with charcoal and calcined (extinct) horse remains in Fell I. A recent excavation expanding from Nami's produced multiple bone ages from faunal samples[164,165]. Stratigraphic information on these is unclear, however, including how each relates to Nami's archaeological components. Samples explicitly noted as cultural (AA-100228 at 11830 ± 30 BP and Beta-319538 at 10410 ± 50 BP), fall within range of previously obtained data. Martin et al.[164] argue that components Fell I and Fell III should be considered as a single cultural component.

Bayesian modelling using Nami et al., data estimates the start of Fell I and Fell III at 13235-12660 (Fishtail) and 12690-12090 cal BP, respectively (see Supplementary Figure 61 and OxCal code). There are three major outliers: PIT-0343 (burned bone; overestimate), PITT-0244 (charcoal; underestimate), and Beta-40281 (mammal bone; underestimate). These likely denote vertical mixing or, for the latter, incomplete decontamination.

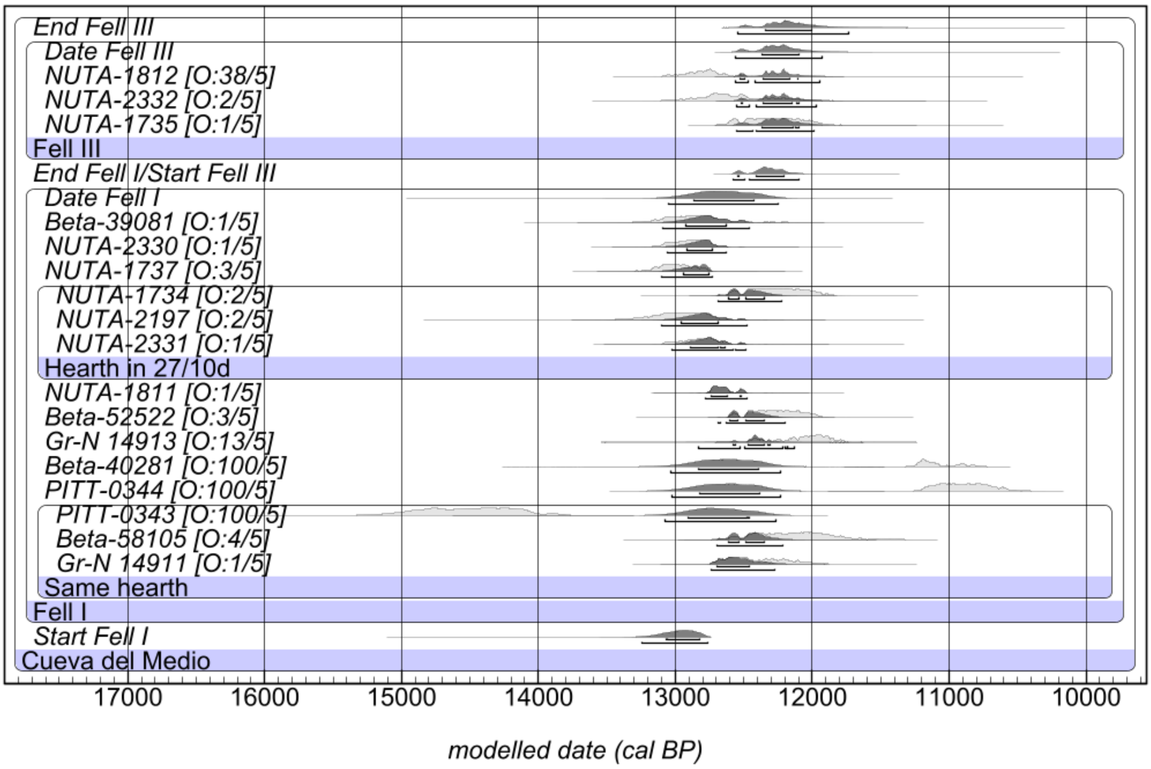


**Supplementary Figure 61.** Bayesian age model for Cueva del Medio. Brackets beneath each age estimate show 68.3% and 95.4% CI. Outlier analysis output is noted as 'O:posterior probability/prior probability'.

```
Plot()
{
 Curve("SHCal20","shcal20.14c");
 Outlier_Model("General",T(5),U(0,4),"t");
 Sequence("Cueva del Medio")
 {
  Boundary("Start Fell I");
  Phase("Fell I")
  {
   Phase("Same hearth")
   {
    R_Date("Gr-N 14911",10550,120)
    {
     Outlier("General", 0.05);
     context="inner portion of the cave, Fell I";
    };
    R_Date("Beta-58105",10350,130)
    {
     Outlier("General", 0.05);
     context="inner portion of the cave, Fell I";
    };
    R_Date("PITT-0343",12390,180)
    {
     Outlier("General", 0.05);
     context="inner portion of the cave, Fell I";
    };
   };
   R_Date("PITT-0344",9595,115)
   {
    Outlier("General", 0.05);
    context="inner portion of the cave, Fell I";
   };
   R_Date("Beta-40281",9770,70)
   {
    Outlier("General", 0.05);
    context="inner portion of the cave, Fell I";
   };
   R_Date("Gr-N 14913",10310,70)
   {
    Outlier("General", 0.05);
    context="inner portion of the cave, Fell I";
   };
   R_Date("Beta-52522",10430,80)
   {
    Outlier("General", 0.05);
    context="front portion of the cave, Fell I";
   };
   R_Date("NUTA-1811",10710,100)
   {
    Outlier("General", 0.05);
    context="inner portion of the cave, Fell 1, 29/8c";
   };
   Phase("Hearth in 27/10d")
   {
    R_Date("NUTA-2331",10860,160)
    {
     Outlier("General", 0.05);
    };
    R_Date("NUTA-2197",11040,250)
    {
     Outlier("General", 0.05);
    };
    R_Date("NUTA-1734",10430,100)
    {
     Outlier("General", 0.05);
    };
   };
   R_Date("NUTA-1737",11120,130)
   {
    Outlier("General", 0.05);
    context="inner portion of the cave, Fell 1, 26/5a";
   };
   R_Date("NUTA-2330",10960,150)
   {
    Outlier("General", 0.05);
    context="inner portion of the cave, Fell 1, 26/5a";
   };
   R_Date("Beta-39081",10930,230)
   {
    Outlier("General", 0.05);
    context="inner portion of the cave, Fell I";
   };
   Date("Date Fell I");
   Interval("Duration Fell I");
  };
  Boundary("End Fell I/Start Fell III");
  Phase("Fell III")
  {
   R_Date("NUTA-1735",10450,100)
   {
    Outlier("General", 0.05);
    context="Fell 3, 28/6c";
   };
   R_Date("NUTA-2332",10710,190)
   {
    Outlier("General", 0.05);
    context="Fell 3, 28/6b";
   };
   R_Date("NUTA-1812",10850,130)
   {
    Outlier("General", 0.05);
    context="Fell 3, 28/6d";
   };
   Date("Date Fell III");
   Interval("Duration Fell III");
  };
  Boundary("End Fell III");
 };
 Difference("Difference Fell I and III","Start Fell I","End Fell I/Start Fell III");
};
```

## 2.4.5. Los Rieles

Los Rieles is an open-air site located in Chile (-31.93, -71.52), containing human burials, lithic technology, faunal remains and hearths[166]. There is a Middle Holocene shell mound (or 'midden', as per the authors) unit containing three human burials (individuals 3, 4, and 6), and a second, underlying palaeo-dune layer containing two further burials (individuals 1 and 2). Individual 5 is represented by an intrusive femur within the latter. Stable isotope data for the humans shows that individuals 1, 2 and 5 had a partly marine diet, whilst the remaining, geologically younger individuals show a preference for terrestrial resources. Given this, mixing models using the R package 'simmr'[167] were created to calculate dietary proportions for the former three individuals (see R code below). Regional stable isotope values for marine and terrestrial (both fauna and plant) were taken from Alfonso-Durruty[168], and diet-to-collagen offset values of 4.8 ± 0.5‰ ($\delta^{13}C$) and 5.5 0.5‰ ($\delta^{15}N$)[169] were applied. Results show an estimated marine contribution of 48.1 ± 12.1%, 25.4 ± 11.5% and 41.5 ± 12.2% for individuals 1, 2 and 5, respectively. $\Delta R$ values applied to each individual (1, 2 and 5) were calculated in OxCal following Macario et al.[170], using temporally-matched, paired ages (terrestrial/marine) from the same latitude reported by Carré et al.[171]. The OxCal code for these $\Delta R$ calculations can be found below.

Bayesian modelling estimates the start of the palaeo-dune unit at 17130-10815 cal BP (or 13825-10925 cal BP at 68.3% CI; Supplementary Figure 62). As mentioned by Jackson et al.[166], the ages for Individual 1 fail a $\chi^2$ test ($\chi^2$ = 8.187; df = 2; p = 6.0) and dates UCIAMS-79662 and BETA-251901 are identified as major outliers. Together with the fact that human osteological material is noted as having undergone conservation, this likely denotes contamination. The combined and modelled age for Individual 1 is 11625-11560 cal BP.

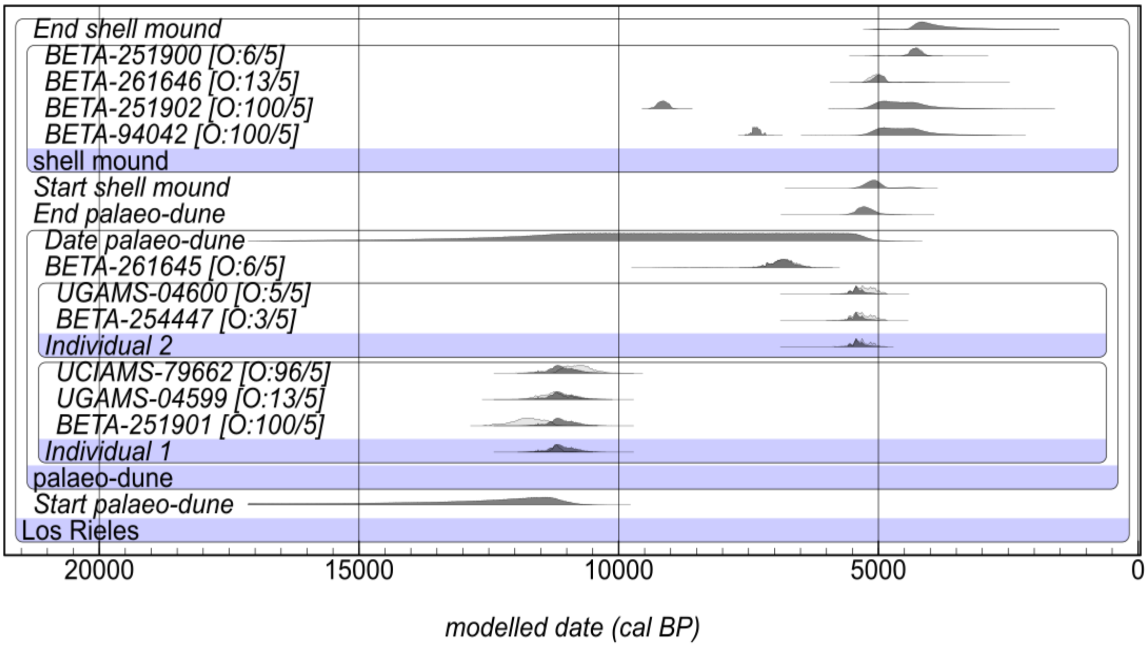


**Supplementary Figure 62.** Bayesian age model for Los Rieles. Brackets beneath each age estimate show 68.3% and 95.4% CI. Outlier analysis output is noted as 'O:posterior probability/prior probability'.

```
# Install and run libraries
libs <- c(
  "simmr", "R2jags", "rjags", "coda")

installed_libs <- libs %in% rownames(
  installed.packages()
)

if(any(installed_libs == F)){
  install.packages(
    libs[!installed_libs]
  )
}

invisible(lapply(libs, library, character.only = T))

############################## Individuo 1 ##############################

# Enter individual/s and baseline isotope information
ind1 = matrix(c(-13.5, 18.0), ncol = 2, nrow = 1)
colnames(ind1) = c('d13C', 'd15N')

# Source information from doi:10.1002/ajpa.23263
s_names = c("marine", "terrestrial fauna", "C4", "C3")
s_means = matrix(c(-14.2, -18.4, -9.7, -25.6, 16.2, 7.6, 7.3, 7.9), ncol=2, nrow=4)
s_sds = matrix(c(0.9, 1.5, 0.4, 0.9, 1, 1.8, 3.4, 1.3), ncol=2, nrow=4)

# Diet-to-tissue isotope offsets from doi:10.1002/ajpa.22788
c_means = matrix(c(4.8,4.8,4.8,4.8,5.5,5.5,5.5,5.5), ncol=2, nrow=4)
c_sds = matrix(c(0.5,0.5,0.5,0.5,0.5,0.5,0.5,0.5), ncol=2, nrow=4)

# Load data into simmr
simmr_in_ind1 = simmr_load(mixtures=ind1,
                     source_names=s_names,
                     source_means=s_means,
                     source_sds=s_sds,
                     correction_means=c_means,
                     correction_sds=c_sds)

# Set high resolution and proper dimensions for the JPEG plot
jpeg("simmr_in_ind1_plot.jpeg",
     width = 1200,  # Width in pixels
     height = 900,  # Height in pixels
     res = 300,     # Resolution in DPI
     quality = 100) # Quality of the JPEG

# Plot data
plot(simmr_in_ind1,
     xlab=expression(paste(delta^13, "C (\u2030)",sep="")),
     ylab=expression(paste(delta^15, "N (\u2030)",sep="")),
     title='Los Rieles - Individuo 1 ')

# Close the graphics device to save the file
dev.off()

# Run MCMC on the simmr object
simmr_out_ind1 = simmr_mcmc(simmr_in_ind1)

# Summarize results
summary(simmr_out_ind1,type='diagnostics')
summary(simmr_out_ind1,type='statistics')
summary(simmr_out_ind1,type='quantiles')

# Plot results
#plot(simmr_out_ind1,type='density')
#plot(simmr_out_ind1,type='matrix')

# Compare sources
compare_sources(
  simmr_out_ind1,
  source_names=c("marine", "terrestrial fauna", "C4", "C3"))

############################## Individuo 2 bone ##############################

# Enter individual/s and baseline isotope information
ind2b=matrix(c(-11,15.6), ncol=2, nrow=1)
colnames(ind2b) = c('d13C','d15N')

# Source information
# Taken from doi:10.1002/ajpa.23263
s_names = c("marine", "terrestrial fauna", "C4", "C3")
s_means = matrix(c(-14.2, -18.4, -9.7, -25.6, 16.2, 7.6, 7.3, 7.9), ncol=2, nrow=4)
s_sds = matrix(c(0.9, 1.5, 0.4, 0.9, 1, 1.8, 3.4, 1.3), ncol=2, nrow=4)

# Diet-to-tissue isotope offsets from doi:10.1002/ajpa.22788
c_means = matrix(c(4.8,4.8,4.8,4.8,5.5,5.5,5.5,5.5), ncol=2, nrow=4)
c_sds = matrix(c(0.5,0.5,0.5,0.5,0.5,0.5,0.5,0.5), ncol=2, nrow=4)

# Load data into simmr
simmr_in_ind2b = simmr_load(mixtures=ind2b,
                     source_names=s_names,
                     source_means=s_means,
                     source_sds=s_sds,
                     correction_means=c_means,
                     correction_sds=c_sds)

# Set high resolution and proper dimensions for the JPEG plot
jpeg("simmr_in_ind2b_plot.jpeg",
     width = 1200,  # Width in pixels
     height = 900,  # Height in pixels
     res = 300,     # Resolution in DPI
     quality = 100) # Quality of the JPEG

# Plot data
plot(simmr_in_ind2b,
     xlab=expression(paste(delta^13, "C (\u2030)",sep="")),
     ylab=expression(paste(delta^15, "N (\u2030)",sep="")),
     title='Los Rieles - Individuo 2 bone ')

# Close the graphics device to save the file
dev.off()

# Run MCMC on the simmr object
simmr_out_ind2b = simmr_mcmc(simmr_in_ind2b)

# Summarize results
summary(simmr_out_ind2b,type='diagnostics')
summary(simmr_out_ind2b,type='statistics')
summary(simmr_out_ind2b,type='quantiles')

# Plot results
#plot(simmr_out_ind2b,type='density')
#plot(simmr_out_ind2b,type='matrix')

# Compare sources
compare_sources(
  simmr_out_ind2b,
  source_names=c("marine", "terrestrial fauna", "C4", "C3"))

############################## Individuo 2 tooth ##############################

# Enter individual/s and baseline isotope information
ind2t=matrix(c(-13.2,16.2), ncol=2, nrow=1)
colnames(ind2t) = c('d13C','d15N')

# Source information
# Taken from doi:10.1002/ajpa.23263
s_names = c("marine", "terrestrial fauna", "C4", "C3")
s_means = matrix(c(-14.2, -18.4, -9.7, -25.6, 16.2, 7.6, 7.3, 7.9), ncol=2, nrow=4)
s_sds = matrix(c(0.9, 1.5, 0.4, 0.9, 1, 1.8, 3.4, 1.3), ncol=2, nrow=4)

# Diet-to-tissue isotope offsets from doi:10.1002/ajpa.22788
c_means = matrix(c(4.8,4.8,4.8,4.8,5.5,5.5,5.5,5.5), ncol=2, nrow=4)
c_sds = matrix(c(0.5,0.5,0.5,0.5,0.5,0.5,0.5,0.5), ncol=2, nrow=4)

# Load data into simmr
simmr_in_ind2t = simmr_load(mixtures=ind2t,
                     source_names=s_names,
                     source_means=s_means,
                     source_sds=s_sds,
                     correction_means=c_means,
                     correction_sds=c_sds)

# Set high resolution and proper dimensions for the JPEG plot
jpeg("simmr_in_ind2t_plot.jpeg",
     width = 1200,  # Width in pixels
     height = 900,  # Height in pixels
     res = 300,     # Resolution in DPI
     quality = 100) # Quality of the JPEG

# Plot data
plot(simmr_in_ind2t,
     xlab=expression(paste(delta^13, "C (\u2030)",sep="")),
     ylab=expression(paste(delta^15, "N (\u2030)",sep="")),
     title='Los Rieles - Individuo 2 tooth ')

# Close the graphics device to save the file
dev.off()

# Run MCMC on the simmr object
simmr_out_ind2t = simmr_mcmc(simmr_in_ind2t)

# Summarize results
summary(simmr_out_ind2t,type='diagnostics')
summary(simmr_out_ind2t,type='statistics')
summary(simmr_out_ind2t,type='quantiles')

# Plot results
#plot(simmr_out_ind2t,type='density')
#plot(simmr_out_ind2t,type='matrix')

# Compare sources
compare_sources(
  simmr_out_ind2t,
  source_names=c("marine", "terrestrial fauna", "C4", "C3"))

############################## Individuo 5 ##############################

# Enter individual/s and baseline isotope information
ind5=matrix(c(-13.3,17.4), ncol=2, nrow=1)
colnames(ind5) = c('d13C','d15N')

# Source information
# Taken from doi:10.1002/ajpa.23263
s_names = c("marine", "terrestrial fauna", "C4", "C3")
s_means = matrix(c(-14.2, -18.4, -9.7, -25.6, 16.2, 7.6, 7.3, 7.9), ncol=2, nrow=4)
s_sds = matrix(c(0.9, 1.5, 0.4, 0.9, 1, 1.8, 3.4, 1.3), ncol=2, nrow=4)

# Diet-to-tissue isotope offsets from doi:10.1002/ajpa.22788
c_means = matrix(c(4.8,4.8,4.8,4.8,5.5,5.5,5.5,5.5), ncol=2, nrow=4)
c_sds = matrix(c(0.5,0.5,0.5,0.5,0.5,0.5,0.5,0.5), ncol=2, nrow=4)

# Load data into simmr
simmr_in_ind5 = simmr_load(mixtures=ind5,
                     source_names=s_names,
                     source_means=s_means,
                     source_sds=s_sds,
                     correction_means=c_means,
                     correction_sds=c_sds)

# Set high resolution and proper dimensions for the JPEG plot
jpeg("simmr_in_ind5_plot.jpeg",
     width = 1200,  # Width in pixels
     height = 900,  # Height in pixels
     res = 300,     # Resolution in DPI
     quality = 100) # Quality of the JPEG

# Plot data
plot(simmr_in_ind5,
     xlab=expression(paste(delta^13, "C (\u2030)",sep="")),
     ylab=expression(paste(delta^15, "N (\u2030)",sep="")),
     title='Los Riles - Individuo 5 ')

# Close the graphics device to save the file
dev.off()

# Run MCMC on the simmr object
simmr_out_ind5 = simmr_mcmc(simmr_in_ind5)

# Summarize results
summary(simmr_out_ind5,type='diagnostics')
summary(simmr_out_ind5,type='statistics')
summary(simmr_out_ind5,type='quantiles')

# Plot results
#plot(simmr_out_ind2,type='density')
#plot(simmr_out_ind2,type='matrix')

# Compare sources
compare_sources(
  simmr_out_ind5,
  source_names=c("marine", "terrestrial fauna", "C4", "C3"))
```

```
 // Delta_R values updated for Marine20
 //method for dR calculation using OxCal in doi:10.1016/j.jenvrad.2015.02.002
 //paired dates from from doi:10.1016/j.yqres.2015.12.002
 Plot()
 {
  Sequence()
  {
   Boundary("Start PPLV80");
   Phase("PPLV80")
```

```
   {
    Curve("SHCal20","shcal20.14c");
    R_Date("OS-60566", 10050, 50);
    R_Date("OS-60559", 10050, 55);
    Curve("Marine20","marine20.14c");
    Delta_R("Delta R for PPLV80",U(-1000,1000));
    R_Date("OS-63181", 10400, 50);
   };
   Boundary("End PPLV80");
  };
  Sequence()
  {
   Boundary("Start LV079");
   Phase("LV079")
   {
    Curve("=SHCal20");
    R_Date("Beta-342528", 9790, 40);
    Curve("=Marine20");
    Delta_R("Delta R for LV079",U(-1000,1000));
    R_Date("Beta-293612", 10360, 50);
    R_Date("Beta-293613", 10640, 60);
   };
   Boundary("End LV079");
  };
  Sequence()
  {
   Boundary("Start Huentelauquen");
   Phase("Huentelauquen")
   {
    Curve("=SHCal20");
    R_Date("Beta-292185", 6000, 40);
    Curve("=Marine20");
    Delta_R("Delta R for Huentelauquen",U(-1000,1000));
    R_Date("Beta-281204", 6350, 40);
   };
   Boundary("End Huentelauquen");
  };
  Sequence()
  {
   Boundary("Start LV007");
   Phase("LV007")
   {
    Curve("=SHCal20");
    R_Date("OS-60569", 3090, 40);
    Curve("=Marine20");
    Delta_R("Delta R for LV007",U(-1000,1000));
    R_Date("OS-63180", 3560, 35);
   };
   Boundary("End LV007");
  };
 };
// Delta_R values updated for Marine20
Plot()
{
 Outlier_Model("SSimple",N(0,2),0,"s");
 Outlier_Model("General",T(5),U(0,4),"t");
 Sequence("Los Rieles")
 {
  Boundary("Start palaeo-dune");
  Phase("palaeo-dune")
  {
   Curve("SHCal20","shcal20.14c");
   Curve("Marine20","marine20.14c");
   Delta_R("Delta R for LV079",243,307);
   Mix_Curve("Mixed1","SHCal20","Delta R for LV079",45.6,10.8);
   //uncertain if duplicates so Combine rather than R_Combine
   Combine("Individual 1")
   {
    R_Date("BETA-251901",10470,60)
    {
     Outlier("SSimple", 0.05);
     context=palaeo-dune unit;
     material=tooth collagen;
     sample=Individual 1;
    };
    R_Date("UGAMS-04599",10150,30)
    {
     Outlier("SSimple", 0.05);
     context=palaeo-dune unit;
     material=tooth collagen;
     sample=Individual 1;
    };
    R_Date("UCIAMS-79662",9815,30)
    {
     Outlier("SSimple", 0.05);
     context=palaeo-dune unit;
     material=petrosal collagen;
     sample=Individual 1;
    };
    Outlier("General", 0.05);
   };
   Delta_R("Delta R for Huentelauquen",-115,382);
   Mix_Curve("Mixed2","SHCal20","Delta R for Huentelauquen",26.3,11.8);
   Combine("Individual 2")
   {
    R_Date("BETA-254447",4720,40)
    {
     Outlier("SSimple", 0.05);
     context=shell mound/midden;
     material=tooth collagen;
     sample=Individual 2;
    };
    R_Date("UGAMS-04600",4680,25)
    {
     Outlier("SSimple", 0.05);
     context=shell mound/midden;
     material=bone collagen;
     sample=Individual 2;
    };
    Outlier("General", 0.05);
   };
   Delta_R("Delta R for LV007",14,384);
   Mix_Curve("Mixed3","SHCal20","Delta R for LV007",40.1,11.3);
   R_Date("BETA-261645",6040,40)
   {
    Outlier("General", 0.05);
    context=palaeo-dune unit, intrusive;
    material=bone collagen;
    sample=Individual 5;
   };
   Date("Date palaeo-dune");
   Interval("Duration palaeo-dune");
  };
  Boundary("End palaeo-dune");
  Interval("Duration potential gap");
  Boundary("Start shell mound");
  Phase("shell mound")
  {
   R_Date("BETA-94042",6440,80)
   {
    Outlier("General", 0.05);
    context=shell mound/midden;
    material=shell;
    sample=shell midden;
   };
   Curve("=Marine20");
   R_Date("BETA-251902",8680,50)
   {
    Outlier("General", 0.05);
    context=palaeo-dune unit;
    material=bone collagen;
    sample=Individual 4;
   };
   R_Date("BETA-261646",4940,40)
   {
    Outlier("General", 0.05);
    context=shell mound/midden;
    material=bone collagen;
    sample=Individual 3;
   };
   R_Date("BETA-251900",4330,40)
   {
    Outlier("General", 0.05);
    context=shell mound/midden;
    material=charcoal;
    sample=hearth;
   };
   Interval("Duration shell mound");
  };
  Boundary("End shell mound");
 };
};
```

## 2.4.6. Pampa Ramaditas 5

Pampa Ramadita 5 is an open-air site in Chile (-21.2, -69.4), containing lithic technology (projectile point)[3,172]. The site is in close vicinity to other Pampa del Tamarugal sites, including Pampa Ramaditas 7 and Quebrada Maní 12. Although obtained from archaeological deposits, >11000 BP ages are considered anomalous by the authors as likely impacted by old wood effects[172].

A single Bayesian model including only <11000 BP measurements estimates the start to 13730-12010 cal BP (Supplementary Figure 63 and OxCal code).

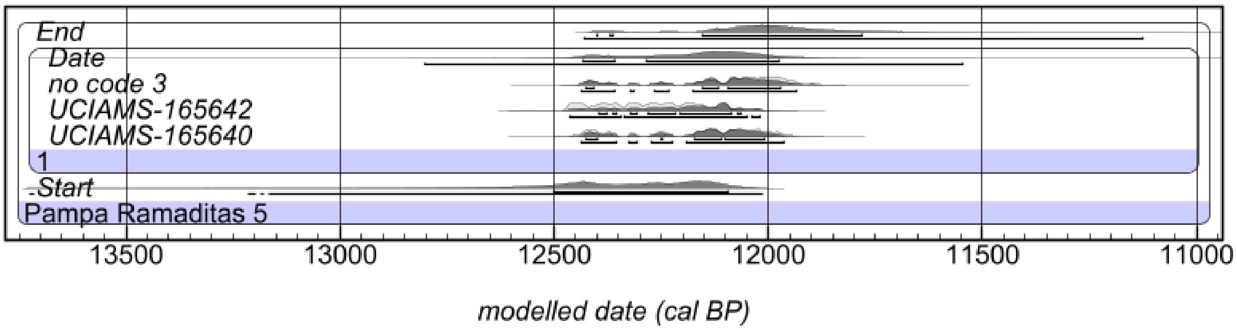


**Supplementary Figure 63.** Bayesian age model for Pampa Ramaditas 5. Brackets beneath each age estimate show 68.3% and 95.4% CI.

```
Plot()
{
 Curve("SHCal20","shcal20.14c");
 Sequence("Pampa Ramaditas 5")
 {
  Boundary("Start");
  Phase("1")
  {
   R_Date("UCIAMS-165640", 10370, 30);
   R_Date("UCIAMS-165642", 10425, 30);
   R_Date("no code 3", 10342, 32);
   Date("Date");
   Interval("Duration");
  };
  Boundary("End");
 };
};
```

2.4.7. Monte Verde I & II

Monte Verde II (MV-II) is an open-air site in Chile (-41.51, -73.2) within the Monte Verde complex, containing lithic and wooden technology, hearths, fibre-based artefacts, and extinct faunal remains (*Cuvieronius hyodon* and *Paleolama sp.*)[173–178]. MV-II is interpreted as a campsite. Habitation surfaces at the site are located within the framework of eight geological strata within the Monte Verde Formation (MV-1 to MV-8). The MV-II habitation surface is found in the upper levels of MV-7 and within MV-6, in three distinct occupational areas (Zones A, D and C). MV-5 includes a peat layer, which developed after the site was abandoned. It is worth noting that the Monte Verde complex includes three other archaeological sites—Monte Verde I (MV-I), Chinchihuapi I (CH-I), and Chinchihuapi II (CH-II)—which also reportedly date to the late Pleistocene and early Holocene periods. In 2015, new findings from MV-I claimed evidence of cultural activity dated to 'at least ~18,500 [...] cal BP' [177].

Bayesian modelling of MV-II estimates the start of MV-7 at 15875-14390 cal BP and MV-6 at 14975-14295 cal BP (marginal overlap at 95.4% CI), with site abandonment (end of MV-6) at 14015-13610 cal BP (see Supplementary Figure 64 and OxCal code). The duration of MV-7 and -6 is estimated at 390 to 2,395 years. Within MV-7 and -6, there is one major outlier (TX-3208, charcoal)—a likely overestimate. Although there were considerations regarding the presence of long-lived species in the site whilst sampling[174], it is possible that this sample represents old wood.

To reflect the pre-ACR evidence in Figure 2, a single-phase model for the 2015 findings at MV-I was created. This has a date range of 24795-11850 cal BP. The OxCal code can be found below.

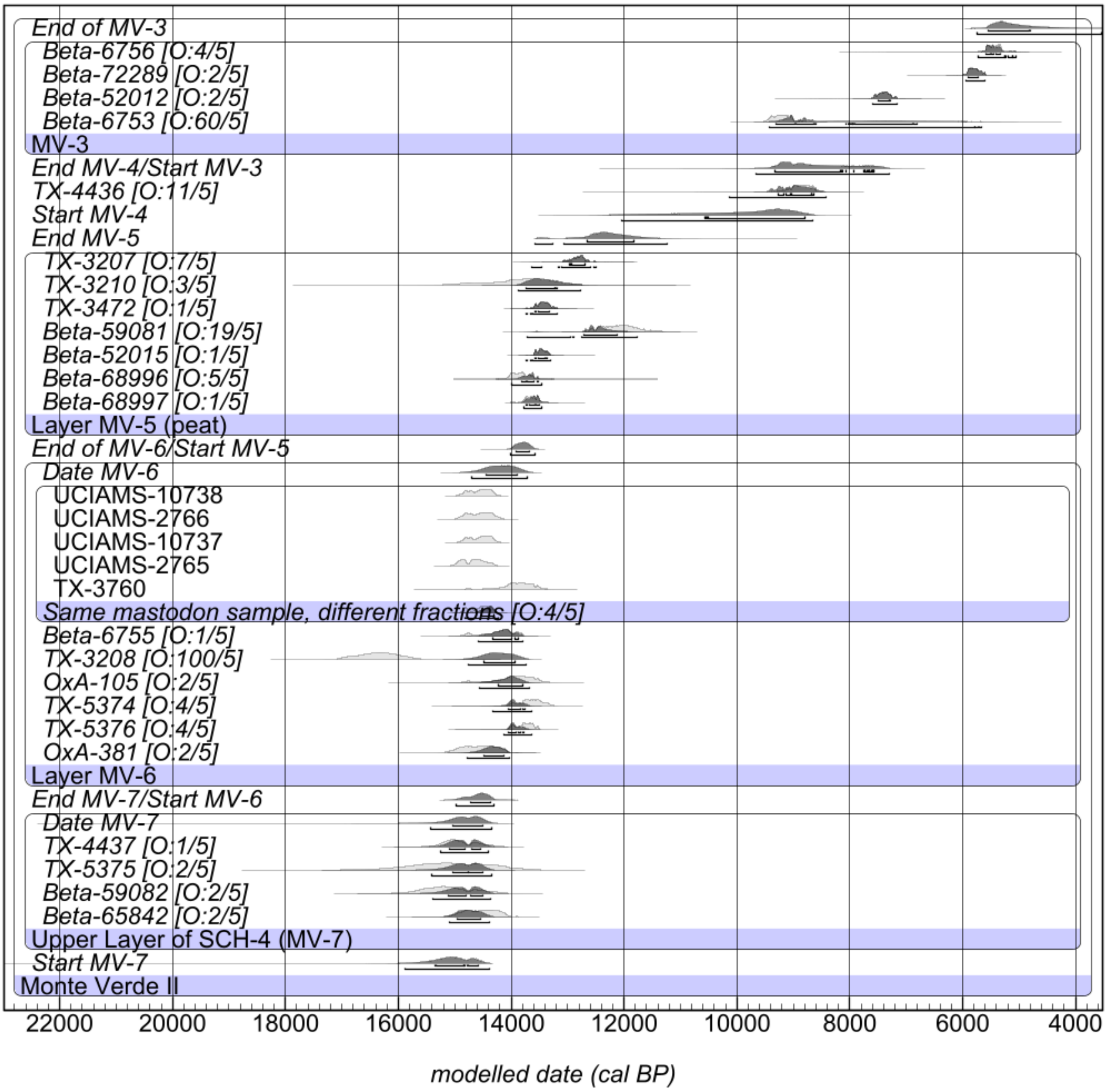


**Supplementary Figure 64.** Bayesian age model for Monte Verde II. Brackets beneath each age estimate show 68.3% and 95.4% CI. Outlier analysis output is noted as 'O:posterior probability/prior probability'.

```
Plot()
{
 Curve("SHCal20","shcal20.14c");
 Outlier_Model("SSimple",N(0,2),0,"s");
 Outlier_Model("General",T(5),U(0,4),"t");
 Sequence("Monte Verde II")
 {
  Boundary("Start MV-7");
  Phase("Upper Layer of SCH-4 (MV-7)")
  {
   R_Date("Beta-65842", 12420, 130)
   {
    Outlier("General", 0.05);
   };
   R_Date("Beta-59082", 12780, 240)
   {
    Outlier("General", 0.05);
   };
   R_Date("TX-5375", 12740, 440)
   {
    Outlier("General", 0.05);
   };
   R_Date("TX-4437", 12650, 130)
   {
    Outlier("General", 0.05);
   };
   Date("Date MV-7");
  };
  Boundary("End MV-7/Start MV-6");
  Phase("Layer MV-6")
  {
   R_Date("OxA-381", 12450, 150)
   {
    Outlier("General", 0.05);
   };
   R_Date("TX-5376", 11920, 120)
   {
    Outlier("General", 0.05);
   };
   R_Date("TX-5374", 11790, 200)
   {
    Outlier("General", 0.05);
   };
   R_Date("OxA-105", 12000, 250)
   {
    Outlier("General", 0.05);
   };
   R_Date("TX-3208", 13565, 250)
   {
    Outlier("General", 0.05);
   };
   R_Date("Beta-6755", 12230, 140)
   {
    Outlier("General", 0.05);
   };
   R_Combine("Same mastodon sample, different fractions")
   {
    R_Date("TX-3760", 11990, 200)
    {
     Outlier("SSimple", 0.05);
    };
    R_Date("UCIAMS-2765", 12510, 60)
    {
     Outlier("SSimple", 0.05);
    };
    R_Date("UCIAMS-10737", 12450, 40)
    {
     Outlier("SSimple", 0.05);
    };
    R_Date("UCIAMS-2766", 12450, 60)
    {
     Outlier("SSimple", 0.05);
    };
    R_Date("UCIAMS-10738", 12455, 40)
    {
     Outlier("SSimple", 0.05);
    };
    Outlier("General", 0.05);
   };
   Date("Date MV-6");
  };
  Boundary("End of MV-6/Start MV-5");
  Phase("Layer MV-5 (peat)")
  {
   R_Date("Beta-68997", 11800, 80)
   {
    Outlier("General", 0.05);
   };
   R_Date("Beta-68996", 12000, 110)
   {
    Outlier("General", 0.05);
   };
   R_Date("Beta-52015", 11640, 90)
   {
    Outlier("General", 0.05);
   };
   R_Date("Beta-59081", 10330, 160)
   {
    Outlier("General", 0.05);
   };
   R_Date("TX-3472", 11600, 120)
   {
    Outlier("General", 0.05);
   };
   R_Date("TX-3210", 11760, 470)
   {
    Outlier("General", 0.05);
   };
   R_Date("TX-3207", 10860, 130)
   {
    Outlier("General", 0.05);
   };
  };
  Boundary("End MV-5");
  Boundary("Start MV-4");
  R_Date("TX-4436", 8030, 130)
  {
   Outlier("General", 0.05);
  };
  Boundary("End MV-4/Start MV-3");
  Phase("MV-3")
  {
   R_Date("Beta-6753", 8270, 110)
   {
    Outlier("General", 0.05);
   };
   R_Date("Beta-52012", 6530, 110)
   {
    Outlier("General", 0.05);
   };
   R_Date("Beta-72289", 5090, 70)
   {
    Outlier("General", 0.05);
   };
   R_Date("Beta-6756", 4750, 90)
   {
    Outlier("General", 0.05);
   };
  };
  Boundary("End of MV-3");
 };
 Difference("Difference MV-7 and -6","Start MV-7","End MV-7/Start MV-6");
 Sequence()
 {
  Boundary("=Start MV-7");
  Interval("Duration MV-II occupation");
  Boundary("=End of MV-6/Start MV-5");
 };
};
Plot()
{
 Curve("SHCal20","shcal20.14c");
 Sequence()
 {
  Boundary("Start");
  Phase("2015 MV-I")
  {
   R_Date("PRI-15-036-1",11959,33);
   R_Date("Beta-372889",16000,60);
   R_Date("Beta-369125",13200,60);
   R_Date("Beta-403545",12350,40);
   R_Date("Beta-372893",15219,30);
   R_Date("Beta-375838",12980,40);
   R_Date("AA-75323",18910,300);
   Date("Date 2015 MV-I");
  };
  Boundary("End");
 };
};
```

## 2.4.8. Pilauco

Pilauco is an open-air palaeontological and archaeological site in Chile (-40.6, -73.1), containing lithic technology (unifacial), extinct faunal remains (including *Notiomastodon platensis*), and a human footprint[179–182]. There are nine layers (PB 1-9) (see Fig. 1 in Moreno et al.[180]), with strata PB-7 and -8 including archaeological evidence. The human footprint is found in the former.

Bayesian modelling estimates the start of PB-7 at 17050-16335 cal BP and PB-8 at 13295-12855 cal BP (marginal overlap at 95.4% CI; see Supplementary Figure 65 and OxCal code). A `Date` function for the footprint-related data yields an estimate of 16575-13120 cal BP (with a comparable 16570-13120 cal BP for PB-7), overlapping with the ACR-YD period. The replicates within this feature fail $\chi^2$ tests and, as such, are significantly different ($\chi^2$ = 59.040; df = 1; p = 3.8 & $\chi^2$ = 85.593; df = 1; p = 3.8). Overall, the sequence includes three major outliers: UCIAMS-101671 (seed or ‘bulk’, depending on publication), UCIAMS-101771 (seed or ‘bulk’, depending on publication), and

PSUAMS-2417 (tooth). These represent a mixture of under- and over-estimates. As such, potential issues include built-in age, insufficient pretreatment, and/or vertical mixing.

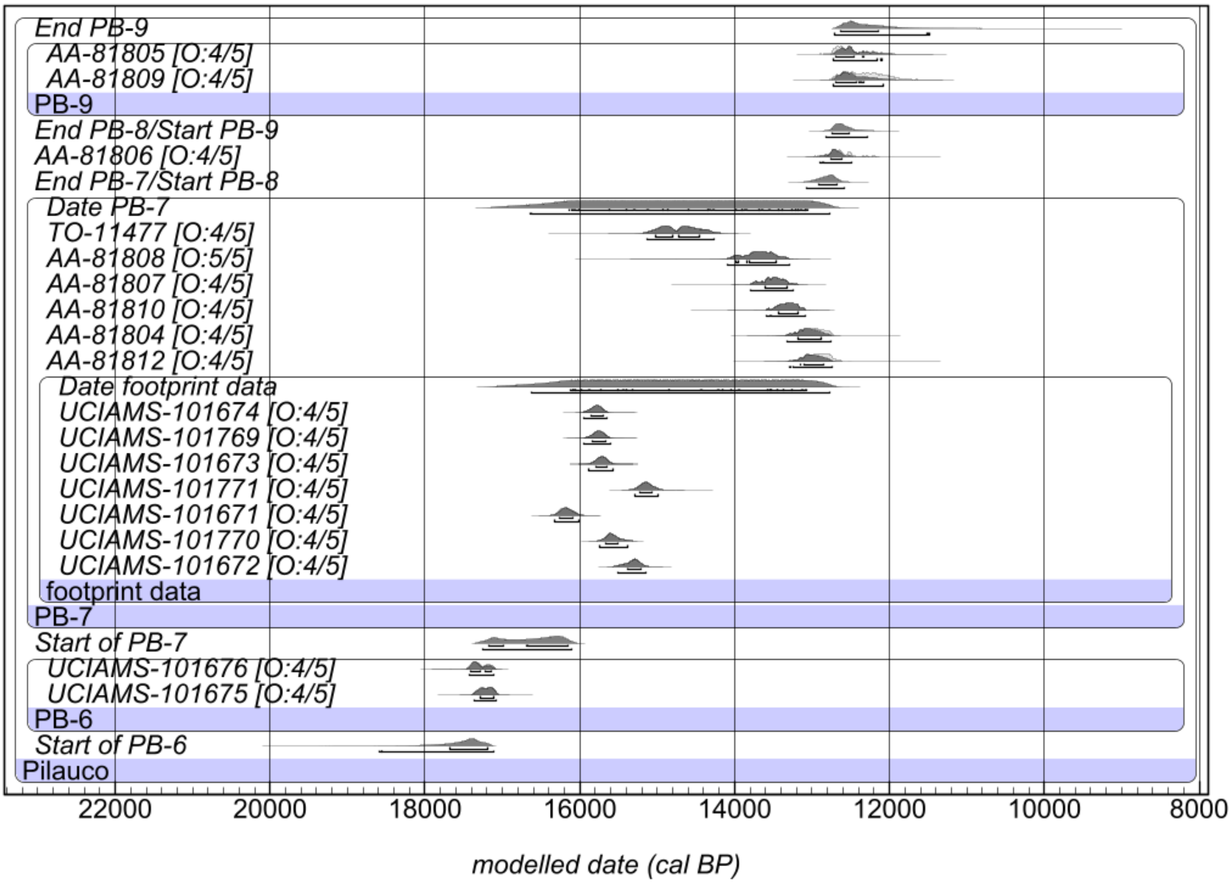


**Supplementary Figure 65.** Bayesian age model for Pilauco. Brackets beneath each age estimate show 68.3% and 95.4% CI. Outlier analysis output is noted as 'O:posterior probability/prior probability'.

```
Plot()
{
 Curve("SHCal20","shcal20.14c");
 Outlier_Model("General",T(5),U(0,4),"t");
 Sequence("Pilauco")
 {
  Boundary("Start of PB-6");
  Phase("PB-6")
  {
   R_Date("UCIAMS-101675",14195,35)
   {
    Outlier("General", 0.05);
    context="top of PB-6";
    material="wood";
   };
   R_Date("UCIAMS-101676",14300,40)
   {
    Outlier("General", 0.05);
    context="top of PB-6";
    material="wood";
   };
  };
  Boundary("Start of PB-7");
  Phase("PB-7")
  {
   Phase("footprint data")
   {
    R_Date("UCIAMS-101672",12860,35)
    {
     Outlier("General", 0.05);
     context="above the trackbed's infilling (PB7)";
     material="wood";
    };
    R_Date("UCIAMS-101770",13045,30)
    {
     Outlier("General", 0.05);
     context="trackbed's infilling";
     material="seed";
    };
    R_Date("UCIAMS-101671",13470,35)
    {
     Outlier("General", 0.05);
     context="trackbed's infilling";
     material="seed";
    };
    R_Date("UCIAMS-101771",12735,40)
    {
     Outlier("General", 0.05);
     context="below the trackbed's surface";
     material="seed";
    };
    R_Date("UCIAMS-101673",13145,35)
    {
     Outlier("General", 0.05);
     context="below the trackbed's surface";
     material="seed";
    };
    R_Date("UCIAMS-101769",13175,40)
    {
     Outlier("General", 0.05);
     context="below the trackbed's surface";
     material="seed";
    };
    R_Date("UCIAMS-101674",13195,35)
    {
     Outlier("General", 0.05);
     context="below the trackbed's surface";
     material="wood";
    };
    Date("Date footprint data");
   };
   R_Date("AA-81812",11004,186)
   {
    Outlier("General", 0.05);
    context="PB-7";
    material="coprolite";
   };
   R_Date("AA-81804",11122,178)
   {
    Outlier("General", 0.05);
    context="PB-7";
    material="wood";
   };
   R_Date("AA-81810",11457,140)
   {
    Outlier("General", 0.05);
    context="PB-7";
    material="teeth";
   };
   R_Date("AA-81807",11665,136)
   {
    Outlier("General", 0.05);
    context="PB-7";
    material="wood";
   };
   R_Date("AA-81808",11834,186)
   {
    Outlier("General", 0.05);
    context="PB-7";
    material="wood";
   };
   R_Date("TO-11477",12540,90)
   {
    Outlier("General", 0.05);
    context="PB-7";
    material="bone";
   };
   Date("Date PB-7");
  };
  Boundary("End PB-7/Start PB-8");
  R_Date("AA-81806",10739,128)
  {
   Outlier("General", 0.05);
   context="PB-8";
   material="wood";
  };
  Boundary("End PB-8/Start PB-9");
  Phase("PB-9")
  {
   R_Date("AA-81809",10517,150)
   {
    Outlier("General", 0.05);
    context="PB-9";
    material="charcoal";
   };
   R_Date("AA-81805",10630,124)
   {
    Outlier("General", 0.05);
    context="PB-9";
    material="wood";
   };
  };
  Boundary("End PB-9");
 };
};
```

### 2.4.9. Punta Ñagué

Punta Ñagué (LV 098A) is a shell mound located in Chile (-31.85, -71.52), containing lithic technology (Huentelauquén complex) and hearths[183,184]. There are three cultural levels with a thin, sterile layer separating the first and second components[183,185–188].

Bayesian modelling estimates the start of the lower component at 14760-12540 cal BP and the middle component at 12475-11185 cal BP, with no outliers (see Supplementary Figure 66 and OxCal code). ΔR values applied to the marine measurements were calculated in OxCal following Macario et al.[170], using roughly temporally-matched, paired ages (terrestrial/marine) from the same locality reported by Carré et al.[171]. The OxCal code for these ΔR calculations can be found below.

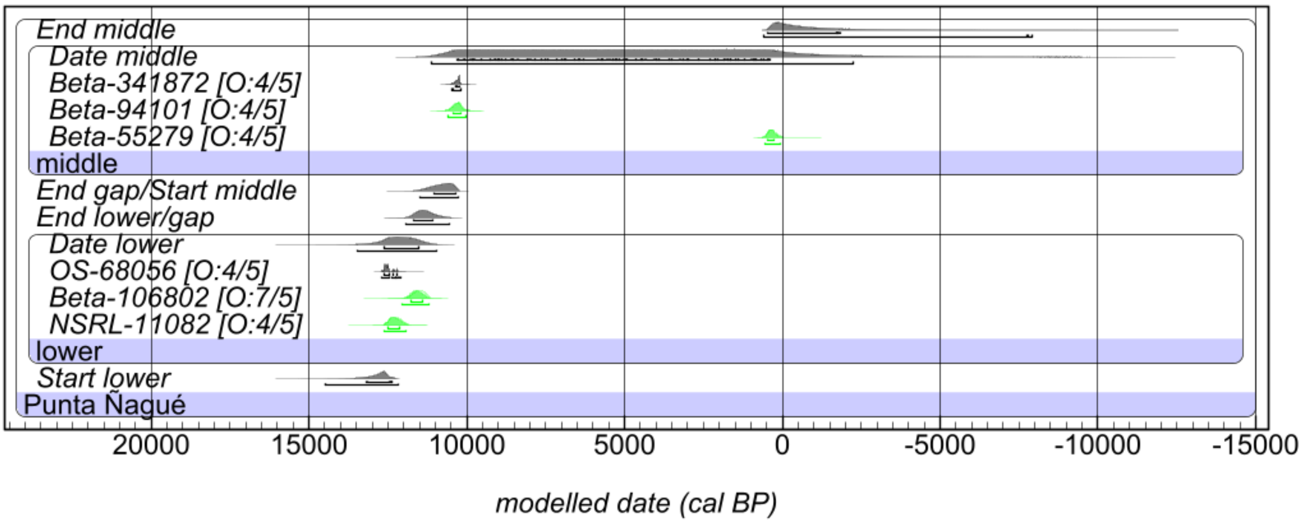


**Supplementary Figure 66.** Bayesian age model for Punta Ñagué. Brackets beneath each age estimate show 68.3% and 95.4% CI. Green distributions represent marine measurements. Outlier analysis output is noted as 'O:posterior probability/prior probability'.

```
// Delta_R values updated for Marine20
Plot()
{
 Outlier_Model("General",T(5),U(0,4),"t");
 Curve("SHCal20","shcal20.14c");
 Sequence("Punta Ñague")
 {
  Boundary("Start lower");
  Phase("lower")
  {
   Curve("Marine20","marine20.14c");
   Delta_R("NagueB", -812, 206);
   R_Date("NSRL-11082",11100,80)
   {
    Outlier("General", 0.05);
    context="lower cultural sequence";
    material="marine shell";
   };
   R_Date("Beta-106802",10600,70)
   {
    Outlier("General", 0.05);
    context="lower cultural sequence";
    material="marine shell";
   };
   Curve("=SHCal20");
   R_Date("OS-68056",10550,50)
   {
    Outlier("General", 0.05);
    context="lower cultural sequence";
    material="charcoal";
   };
   Date("Date lower");
  };
  Boundary("End lower/gap");
  Boundary("End gap/Start middle");
  Phase("middle")
  {
   Curve("=Marine20");
   Delta_R("=NagueB");
   R_Date("Beta-55279",1012,80)
   {
    Outlier("General", 0.05);
    context="middle cultural sequence";
    material="marine shell";
   };
   R_Date("Beta-94101",9730,60)
   {
    Outlier("General", 0.05);
    context="middle cultural sequence";
    material="marine shell";
   };
   Curve("=SHCal20");
   R_Date("Beta-341872",9160,40)
   {
    Outlier("General", 0.05);
    context="middle cultural sequence";
    material="charcoal";
   };
   Date("Date middle");
  };
  Boundary("End middle");
 };
};
// Delta_R values updated for Marine20
//method for dR calculation using OxCal in doi:10.1016/j.jenvrad.2015.02.002
//paired dates from from doi:10.1016/j.yqres.2015.12.002
Plot()
{
 Sequence()
 {
  Boundary("Start");
  Phase("NagueB")
  {
   Curve("SHCal20","shcal20.14c");
   R_Date("OS-61906", 9190, 45);
   R_Date("OS-61450", 9190, 130);
   Curve("Marine20","marine20.14c");
   Delta_R("Delta R for NagueB",U(-1000,1000));
   R_Date("OS-63177", 9310, 50);
  };
  Boundary("End");
 };
};
```

## 2.4.10. Tagua Tagua

Tagua Tagua 1 (TT-1), 2 (TT-2) and 3 (TT-2) are open-air sites located in Chile (approximately -34.4, -71.2), containing lithic technology and extinct faunal remains. The sites were excavated by J. Montané in 1967 (TT-1)[189,190], by L. Núñez in the 1990s (TT-1 was expanded and TT-2 first excavated)[191] and R. Labarca in 2019-2022[192]. Within lithostratigraphic units 5 and 6, at a depth range of 1.04-2.5 m, TT-1 and -2 sites contain the remains of extinct fauna—predominantly mastodon (*Stegomaston humboldti*), horse (*Equus*), and deer (*Antifer niemeyeri*)—found in close association with lithic and osseous artefacts. In total, the Tagua Tagua sites contain >300 skeletal elements distributed in fourteen different loci, as well as 22 lithic tools (including three Fell-style points extracted from TT-2) and four bone artefacts. Coupled with this evidence, the lack of anatomical arrangement of single individuals and the presence of cut marks in bone fragments suggest that TT-1 and -2 were Paleoindian butchering sites[193]. In 1968, the chronology at TT-1 was established by a single charcoal date of 11380 ± 320 BP (GX-1205)[190,194]. Following the Núñez excavations, charcoal

dates 9900 ± 100 BP (Beta-45519) and 10120 ± 30 BP (Beta-45520) were obtained for TT-2. Direct dating of the human-modified bone fragments had not been attempted since the 1960s when, according to Montané[194], the age of a mastodon bone could not be determined due to low collagen yield. In 2018, with the aim of directly dating the faunal remains, the present author obtained eight bone samples from TT-1 and -2 for radiocarbon dating. These included samples of *Stegomaston platensis* and *Equus* found by Montané in TT-1; a cut-marked *Stegomaston platensis* vertebra from TT-2 ; a fragment of a *Stegomaston platensis* mandible corresponding to a second, infant individual from TT-2; and cut-marked bird bone fragments from both sites. Unfortunately, none of the samples yielded collagen. This was consistent with previous[194] and recent[195] findings, suggesting poor collagen preservation. TT-3 has geological and archaeological chronometric data—which reportedly correlate with TT-1, but not TT-2—with evidence of early cultural activity in facies L4b (see Fig. 3 in Labarca et al.[192]). This site has evidence of *Gomphotherium* hunting.

Bayesian modelling of combined data from TT-1 and -2 estimates the start of the Paleoindian level at 14280-12735 cal BP (Fell style), with no outliers (see Supplementary Figure 67 and OxCal code). Following this, cultural activity is evidenced in a second component, which is estimated to have started at 7255-6675 cal BP and is based on one date (I-3987; 6130 ± 115 BP). As for TT-3, only archaeo-chronometric data from facies L4b (including a combustion feature) was entered into a single-phase Bayesian model. This estimates the start for the cultural activity to 13025-12475 cal BP (Supplementary Figure 68). There is overlap between the two distributions (for TT-1-2 and TT-3; Supplementary Figure 69) at 95.4% CI, although TT-1-2 might be considered marginally earlier.

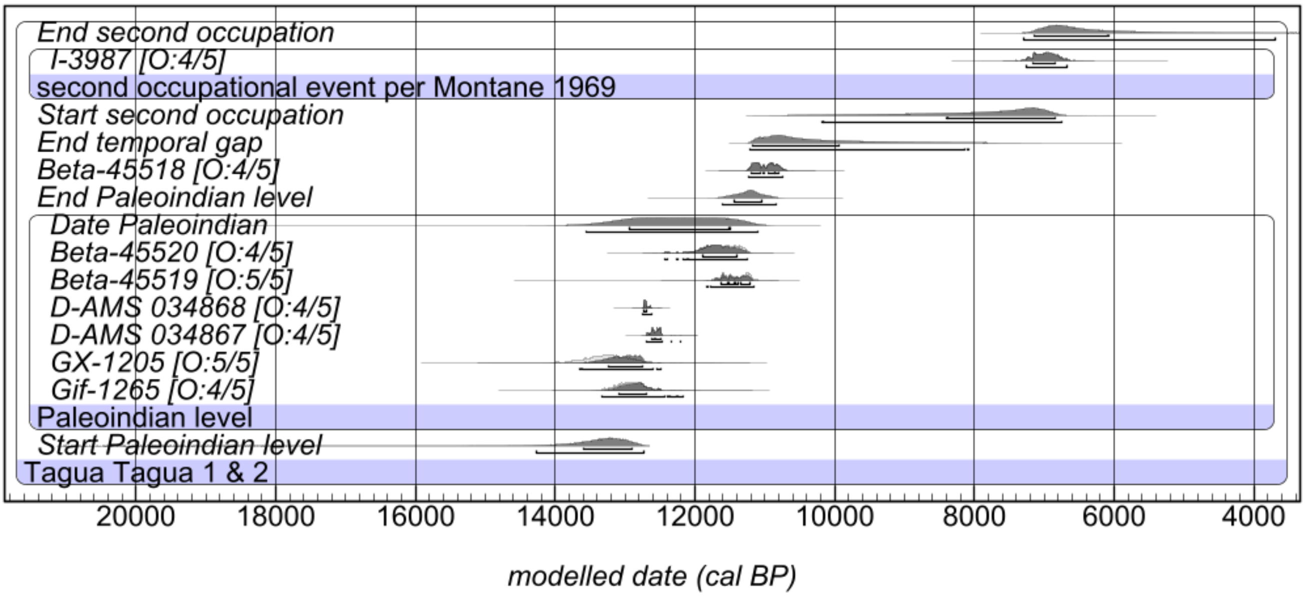


**Supplementary Figure 67.** Bayesian age model for Tagua Tagua 1-2. Brackets beneath each age estimate show 68.3% and 95.4% CI. Outlier analysis output is noted as 'O:posterior probability/prior probability'.

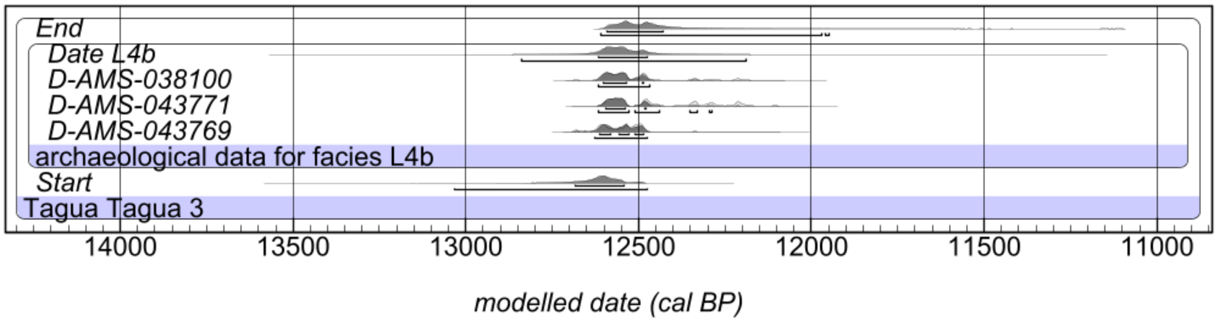


**Supplementary Figure 68.** Bayesian age model for Tagua Tagua 3. Brackets beneath each age estimate show 68.3% and 95.4% CI.

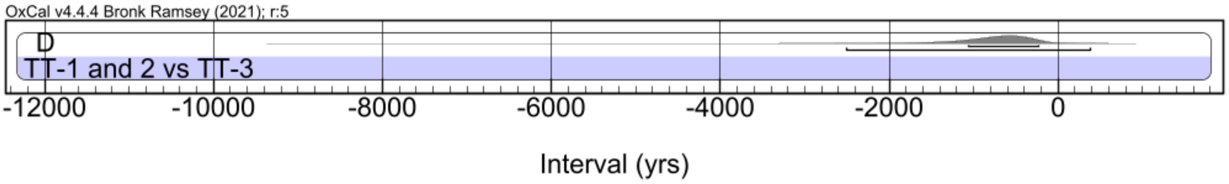


**Supplementary Figure 69.** Probability density function for the difference ('D') between the start cultural activity in TT-1 and -2 (Supplementary Figure 67) vs TT-3 (Supplementary Figure 68). These results suggest that there is no significant difference between the modelled outputs, as the distributions include zero at 95.4% CI (black bracket beneath).

```
Plot()
{
 Curve("SHCal20","shcal20.14c");
 Outlier_Model("General",T(5),U(0,4),"t");
 Sequence("Tagua Tagua 1 & 2")
 {
  Boundary("Start Paleoindian level");
  Phase("Paleoindian level")
  {
   R_Date("Gif-1265",11000,250)
   {
    Outlier("General", 0.05);
    context="late Pleistocene archaeological level";
   };
   R_Date("GX-1205",11380,320)
   {
    Outlier("General", 0.05);
    context="late Pleistocene archaeological level";
   };
   R_Date("D-AMS 034867",10578,48)
   {
    Outlier("General", 0.05);
    context="late Pleistocene archaeological level reported by Nuñez et al. (1994), excavation units C3–F3";
   };
   R_Date("D-AMS 034868",10738,43)
   {
    Outlier("General", 0.05);
    context="late Pleistocene archaeological level reported by Nuñez et al. (1994), excavation units C3–F3";
   };
   R_Date("Beta-45519",9900,100)
   {
    Outlier("General", 0.05);
    context="charcoal in same level as Mastodon";
   };
   R_Date("Beta-45520",10120,130)
   {
    Outlier("General", 0.05);
    context="charcoal adjecent to Mastodon rib";
   };
   Interval("Duration Paleoindian");
   Date("Date Paleoindian");
  };
  Boundary("End Paleoindian level");
  R_Date("Beta-45518",9710 ,90)
  {
   Outlier("General", 0.05);
   context="immediately above the Paleoindian level";
  };
  Boundary("End temporal gap");
  Boundary("Start second occupation");
  Phase("second occupational event per Montane 1969")
  {
   R_Date("I-3987",6130,115)
   {
    Outlier("General", 0.05);
    context="Holocene level, above 1 m depth";
   };
  };
  Boundary("End second occupation");
 };
};

Plot()
{
 Curve("SHCal20","shcal20.14c");
 Sequence("Tagua Tagua 3")
 {
  Boundary("Start");
  Phase("archaeological data for facies L4b")
  {
   R_Date("D-AMS-043769",10572,39);
   R_Date("D-AMS-043771",10503,36);
   R_Date("D-AMS-038100",10536,39);
   Date("Date L4b");
  };
  Boundary("End");
 };
};
```

## 2.4.11. Tres Arroyos 1 (TA1)

Tres Arroyos 1 (TA1) is a rockshelter located in Chile (-53.383, -68.783), containing extinct faunal remains [*Eutatus* sp., *Mylodon* sp., *Hemiauchenia paradoxa*, *G. robustum*, *Toxodon platensis*, *Macrauchenia patachonica*, *Glyptodon* sp., *M. americanum*, *Hippidion principale*, *E. (Amerhippus) neogeus*], lithic technology and hearths[196–199]. There are six levels (I-VI), with the earliest human occupation at Va. There is evidence of vertical mixing, most likely caused by rabbit burrowing[199].

Bayesian modelling estimates the start of Va at 13915-12490 cal BP (see Supplementary Figure 70 and OxCal code). There are four major outliers: Dic-2733 (bone; Level Vb), Beta-20219

(undetermined burnt bone; Level Va), Beta-101054 (bone; Level Va) and OxA-9245 (tooth; Level III). Dic-2733 and Beta-101054 are likely underestimate ages, whilst Beta-20219 and OxA-9245 are overestimates. Given evidence for bioturbation, these likely denote vertical mixing. However, since there is limited information regarding bone/tooth pretreatment and quality control parameters are unreported, incomplete decontamination for the underestimated ages cannot be excluded. Following Va, it is unclear whether IV represents a human occupation, with Borrero[199] noting that this layer, 'dated c. 1300 yr BP rests above Layer Va, which is Late Pleistocene.' For this work, it is assumed that cultural occupation is only evidenced at Va.

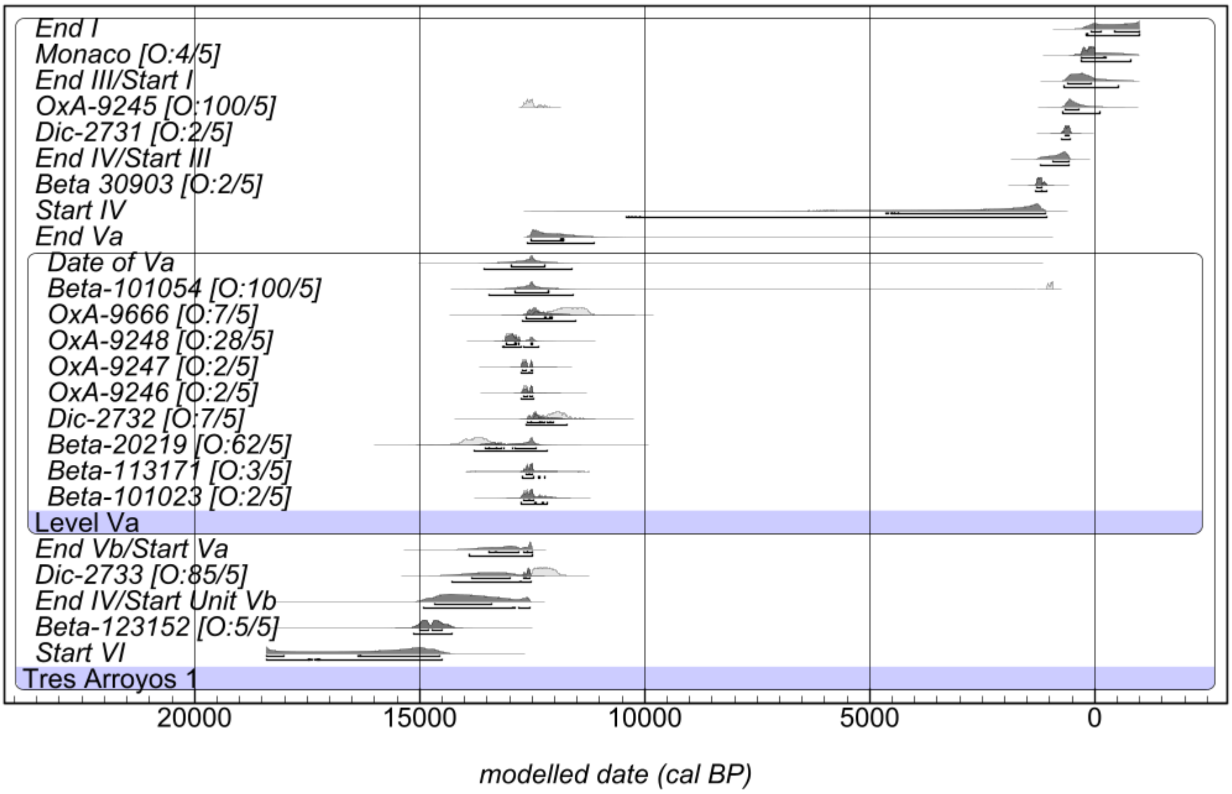


**Supplementary Figure 70.** Bayesian age model for Tres Arroyos 1. Brackets beneath each age estimate show 68.3% and 95.4% CI. Outlier analysis output is noted as 'O:posterior probability/prior probability'.

```
Plot()
{
 Curve("SHCal20","shcal20.14c");
 Outlier_Model("General",T(5),U(0,4),"t");
 Sequence("Tres Arroyos 1")
 {
  Boundary("Start VI");
  R_Date("Beta-123152",12540,70)
  {
   Outlier("General", 0.05);
   context=Level VI;
   material=bone;
  };
  Boundary("End IV/Start Unit Vb");
  R_Date("Dic-2733",10420,100)
  {
   Outlier("General", 0.05);
   context=Level Vb, Fell 1;
   material=bone;
  };
  Boundary("End Vb/Start Va");
  Phase("Level Va")
  {
   R_Date("Beta-101023",10600,90)
   {
    Outlier("General", 0.05);
    context=Level Va, Fell 1, fogón 2;
    material=charcoal;
   };
   R_Date("Beta-113171",10580,50)
   {
    Outlier("General", 0.05);
    context=Level Va, Fell 1, fogón 3;
    material=charcoal;
   };
   R_Date("Beta-20219",11880,250)
   {
    Outlier("General", 0.05);
    context=Level Va, Fell 1, fogón 1;
    material=undetermined burnt bones;
   };
   R_Date("Dic-2732",10280,110)
   {
    Outlier("General", 0.05);
    context=Level Va, Fell 1;
    material=bone;
   };
   R_Date("OxA-9246",10630,70)
   {
    Outlier("General", 0.05);
    context=Level Va, Fell 1;
    material=bone;
   };
   R_Date("OxA-9247",10685,70)
   {
    Outlier("General", 0.05);
    context=Level Va, Fell 1;
    material=bone;
   };
   R_Date("OxA-9248",11085,70)
   {
    Outlier("General", 0.05);
    context=Level Va, Fell 1;
    material=bone collagen;
   };
   R_Date("OxA-9666",10130,210)
   {
    Outlier("General", 0.05);
    context=Level Va, Fell 1, fogón 4;
    material=charcoal;
   };
   R_Date("Beta-101054",1120,40)
   {
    Outlier("General", 0.05);
    context=Level Va;
    material=bone;
   };
   Interval("Duration of Va");
   Date("Date of Va");
  };
  Boundary("End Va");
  Boundary("Start IV");
  R_Date("Beta 30903",1340,50)
  {
   Outlier("General", 0.05);
   context=Level IV;
   material=bone;
  };
  Boundary("End IV/Start III");
  R_Date("Dic-2731",700,70)
  {
   Outlier("General", 0.05);
   context=Level III;
   material=charcoal;
  };
  R_Date("OxA-9245",10575,65)
  {
   Outlier("General", 0.05);
   context=Level III;
   material=tooth;
  };
  Boundary("End III/Start I");
  R_Date("Monaco",135,85)
  {
   Outlier("General", 0.05);
   context=Level I;
   material=charcoal;
  };
  Boundary("End I");
 };
};
```

2.4.12. Quebrada Maní 12, 32 and 35

Quebrada Maní 12 (QM12), 32 (QM32) and 35 (QM35) are open-air sites within the Quebrada de Maní archaeological locality/catchment in Chile (approximately -21.2, -69.4; at 1,240 m.a.s.l.), containing lithic and wooden technology[3,172,200–203]. Within QM12, test pits QM12a, QM12b and QM12c were excavated, with the latter being expanded to a 2x2 area[200,204]. There are five stratigraphic strata (1-5), with stratum 5 bearing no cultural remains (see Fig 5 in Latorre et al.[200]). Although there is contextual information for QM 32 and 35, the stratigraphy is not described by Santoro et al.[172] or Herrera et al.[204]. Moreover, there is recently published archaeo-chronometric data available for the Quebrada de Maní locality[3], but it is unclear how this relates with previous information (stratigraphically). Occupational phases for QM32, for example, were defined after determining the distribution of the chronometric data using the `Sum` function in OxCal. QM35 contains projectile point technology, QM32 only unifacial, and QM12 surface-found projectile points[3,200]

A multi-phase Bayesian model for QM12c using Latorre et al.[200] and Herrera et al.[204] data estimates the start of stratum 4-1 at 13560-12025 cal BP, 12765-11970 cal BP, 11810-11530 cal BP and 11720-11110 cal BP, respectively (Supplementary Figure 71 and OxCal code). There are five major outliers. This includes ages within the two sets of charcoal duplicates at the beginning and end of the sequence (both fail each $\chi^2$ test), as well as two dates within stratum 2 (UCIAMS-89019 and -89016). Discrepancy within the duplicates likely reflects differences in pretreatment, whilst those within stratum 2 likely indicate vertical mixing and/or inbuilt age (both overestimates). A ΔR of 40±126 was applied to the marine gastropod age. This was calculated using Merino-Campos et al.[205] data for the north upwelling coastal region (18-30°S) of Chile, but updated using the Marine20 curve[206] through calib.org[207] (with the collection date set to the first year of the quoted range).

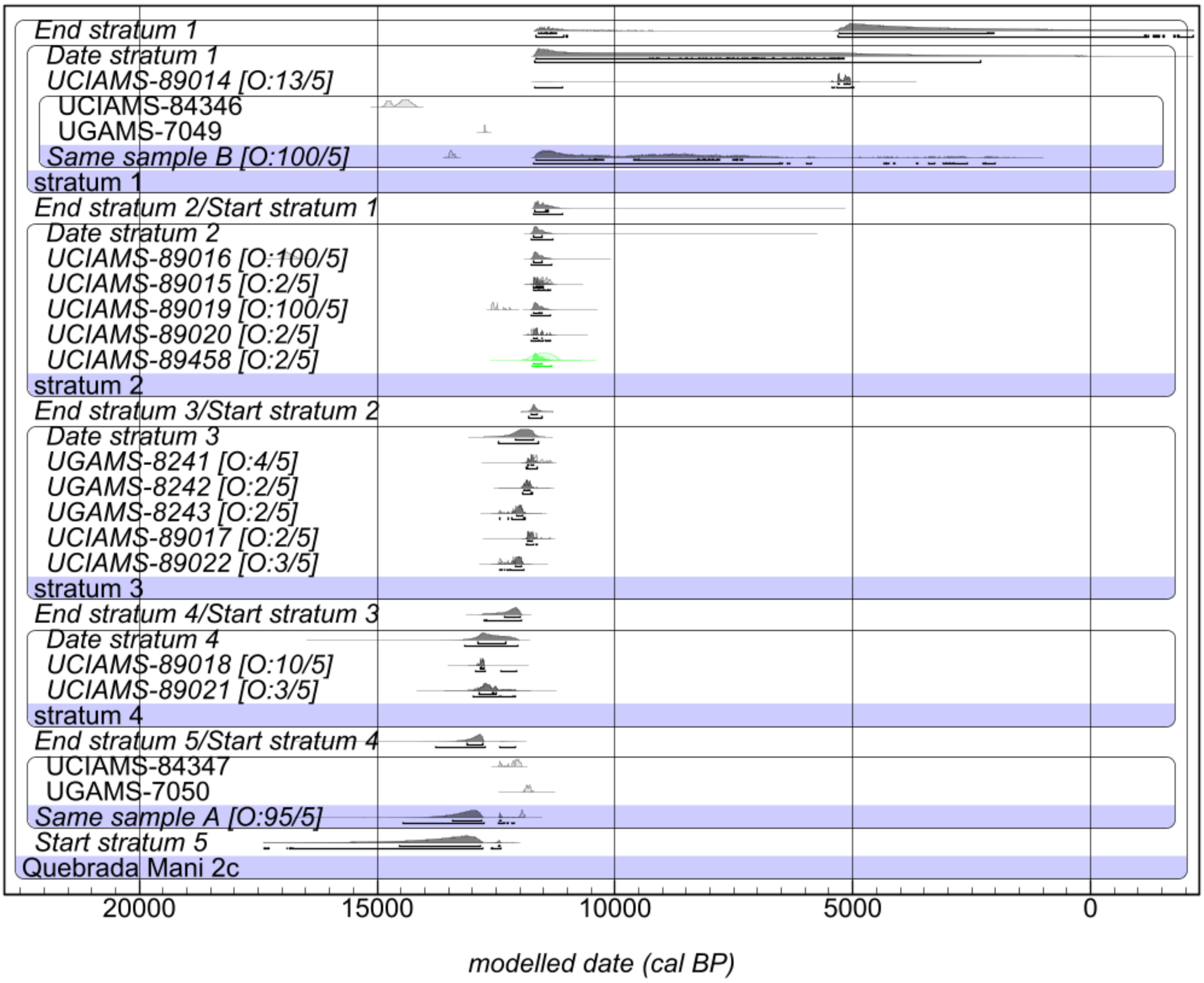


**Supplementary Figure 71.** Bayesian age model for QM12c. Brackets beneath each age estimate show 68.3% and 95.4% CI. The green distribution denotes a marine measurement. Outlier analysis output is noted as 'O:posterior probability/prior probability'.

Single-phase Bayesian models for QM32 and QM35 estimate the start of occupation at 15477-14428 cal BP and 13670-12925 cal BP (Supplementary Figures 72-73). The former is not statistically comparable to that of QM12c (stratum 4) and QM35, denoting an earlier cultural event in the same locality. This is contrary to the assertion in Ugalde et al.[3] that 'QM12 was the first location to be occupied by humans at the Pampa del Tamarugal and the Atacama Desert at large'.

The Bayesian estimates produced here do not coincide with those in Ugalde et al.[3]. Resolving this is a challenge as the OxCal code was not published and the model figures do not list laboratory codes.

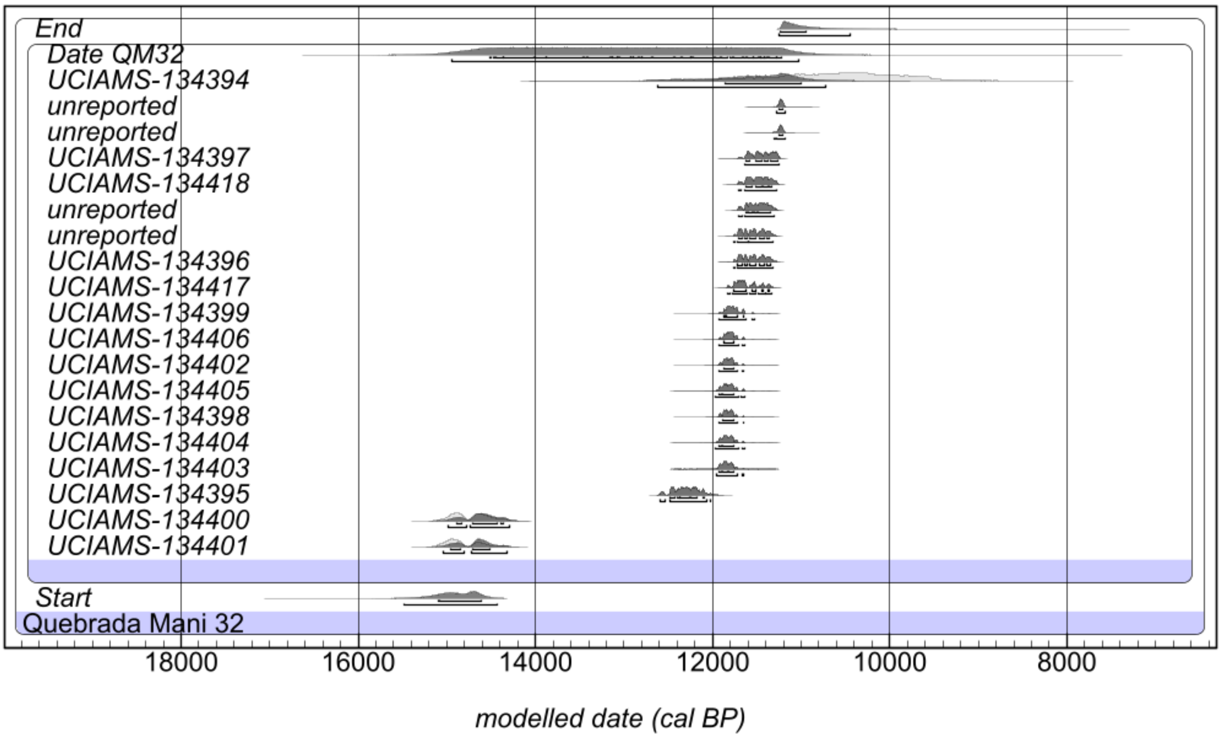


**Supplementary Figure 72.** Bayesian age model for QM32. Brackets beneath each age estimate show 68.3% and 95.4% CI. Outlier analysis output is noted as ‘O:posterior probability/prior probability’.

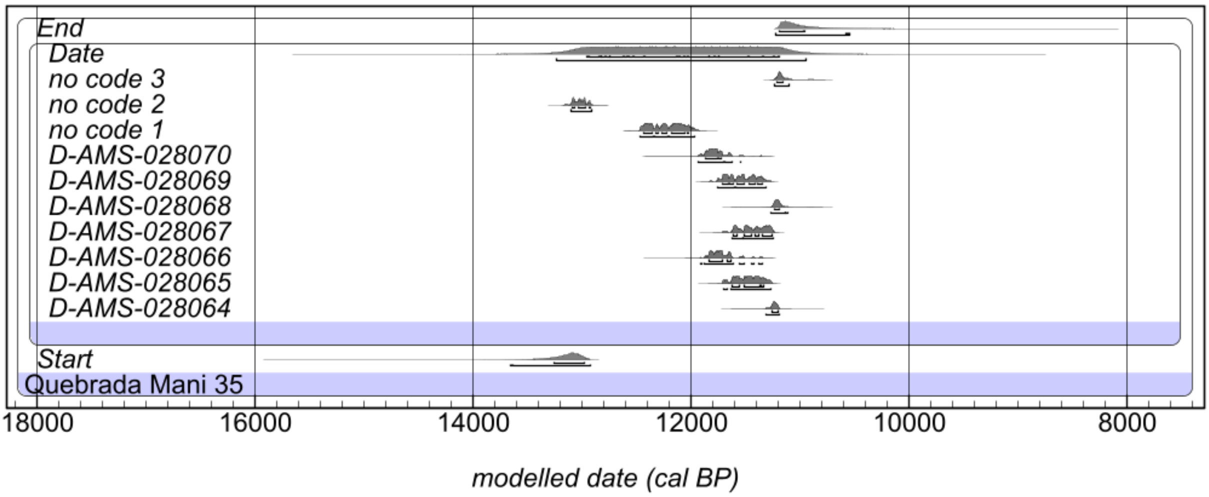


**Supplementary Figure 73.** Bayesian age model for QM35. Brackets beneath each age estimate show 68.3% and 95.4% CI. Outlier analysis output is noted as ‘O:posterior probability/prior probability’.

```
// Delta_R values updated for Marine20
Plot()
{
 Curve("SHCal20","shcal20.14c");
 Outlier_Model("SSimple",N(0,2),0,"s");
 Outlier_Model("General",T(5),U(0,4),"t");
 Sequence("Quebrada Mani 12c")
 {
  Boundary("Start stratum 5");
  R_Combine("Same sample A")
  {
   R_Date("UGAMS-7050", 10210, 30)
   {
    Outlier("SSimple", 0.05);
   };
   R_Date("UCIAMS-84347", 10365, 25)
   {
    Outlier("SSimple", 0.05);
   };
   Outlier("General", 0.05);
  };
  Boundary("End stratum 5/Start stratum 4");
  Phase("stratum 4")
  {
   R_Date("UCIAMS-89021", 10760, 170)
   {
    Outlier("General", 0.05);
   };
   R_Date("UCIAMS-89018", 10930, 30)
   {
    Outlier("General", 0.05);
   };
   Date("Date stratum 4");
   Interval("Interval stratum 4");
  };
  Boundary("End stratum 4/Start stratum 3");
  Phase("stratum 3")
  {
   R_Date("UCIAMS-89022", 10360, 30)
   {
    Outlier("General", 0.05);
   };
   R_Date("UCIAMS-89017", 10160, 25)
   {
    Outlier("General", 0.05);
   };
   R_Date("UGAMS-8243", 10340, 30)
   {
    Outlier("General", 0.05);
   };
   R_Date("UGAMS-8242", 10220, 30)
   {
    Outlier("General", 0.05);
   };
   R_Date("UGAMS-8241", 10130, 30)
   {
    Outlier("General", 0.05);
   };
   Date("Date stratum 3");
   Interval("Interval stratum 3");
  };
  Boundary("End stratum 3/Start stratum 2");
  Phase("stratum 2")
  {
   Curve("Marine20","marine20.14c");
   Delta_R("north upwelling region", 40, 126);
   R_Date("UCIAMS-89458", 10655, 25)
   {
    Outlier("General", 0.05);
   };
   Curve("=SHCal20");
   R_Date("UCIAMS-89020", 10120, 25)
   {
    Outlier("General", 0.05);
   };
   R_Date("UCIAMS-89019", 10505, 25)
   {
    Outlier("General", 0.05);
   };
   R_Date("UCIAMS-89015", 10080, 25)
   {
    Outlier("General", 0.05);
   };
   R_Date("UCIAMS-89016", 13920, 40)
   {
    Outlier("General", 0.05);
   };
   Date("Date stratum 2");
   Interval("Interval stratum 2");
  };
  Boundary("End stratum 2/Start stratum 1");
  Phase("stratum 1")
  {
   R_Combine("Same sample B")
   {
    R_Date("UGAMS-7049", 10800, 30)
    {
     Outlier("SSimple", 0.05);
    };
    R_Date("UCIAMS-84346", 12420, 35)
    {
     Outlier("SSimple", 0.05);
    };
    Outlier("General", 0.05);
   };
   R_Date("UCIAMS-89014", 4580, 20)
   {
    Outlier("General", 0.05);
   };
   Date("Date stratum 1");
   Interval("Interval stratum 1");
  };
  Boundary("End stratum 1");
 };
 Difference("Difference 4 and 3","End stratum 5/Start stratum 4","End stratum 4/Start stratum 3");
 Difference("Difference 3 and 2","End stratum 4/Start stratum 3","End stratum 3/Start stratum 2");
 Difference("Difference 4 and 2","End stratum 5/Start stratum 4","End stratum 3/Start stratum 2");
 Difference("Difference 1 and 2","End stratum 2/Start stratum 1","End stratum 3/Start stratum 2");
 Sequence()
 {
  Boundary("=End stratum 5/Start stratum 4");
  Interval("Duration occupation 4-2");
  Date("Date ocupation 4-2");
  Boundary("=End stratum 2/Start stratum 1");
 };
};


Plot()
{
 Curve("SHCal20","shcal20.14c");
 Sequence("Quebrada Mani 32")
 {
  Boundary("Start");
  Phase("")
  {
   R_Date("UCIAMS-134401",12580,50)
   {
    material="charcoal";
   };
   R_Date("UCIAMS-134400",12540,60)
   {
    context="";
    material="charcoal";
   };
   R_Date("UCIAMS-134395",10445,45)
   {
    context="";
    material="plant material";
   };
   R_Date("UCIAMS-134403",10220,40)
   {
    context="";
    material="charcoal";
   };
   R_Date("UCIAMS-134404",10215,45)
   {
    context="";
    material="charcoal";
   };
   R_Date("UCIAMS-134398",10210,35)
   {
    context="";
    material="plant material";
   };
   R_Date("UCIAMS-134405",10210,45)
   {
    context="";
    material="charcoal";
   };
   R_Date("UCIAMS-134402",10205,35)
   {
    context="";
    material="charcoal";
   };
   R_Date("UCIAMS-134406",10195,35)
   {
    context="";
    material="charcoal";
   };
   R_Date("UCIAMS-134399",10180,40)
   {
    context="";
    material="plant material";
   };
   R_Date("UCIAMS-134417",10115,35)
   {
    context="";
    material="rodent pellet (acid only)";
   };
   R_Date("UCIAMS-134396",10085,35)
   {
    context="";
    material="plant material";
   };
   R_Date("unreported",10082,30)
   {
    context="";
    material="wood";
   };
   R_Date("unreported",10057,28)
   {
    context="";
    material="hair";
   };
   R_Date("UCIAMS-134418",10040,35)
   {
    context="";
    material="plant material, cordage (acid only)";
   };
   R_Date("UCIAMS-134397",10005,45)
   {
    context="";
    material="plant material";
   };
   R_Date("unreported",9874,36)
   {
    context="";
    material="hair cordage";
   };
   R_Date("unreported",9865,33)
   {
    context="";
    material="wood";
   };
   R_Date("UCIAMS-134394",9290,510)
   {
    context="";
    material="plant material";
   };
   Date("Date QM32");
   Interval("Duration QM32");
  };
  Boundary("End");
 };
};

Plot()
{
 Curve("SHCal20","shcal20.14c");
 Sequence("Quebrada Mani 35")
 {
  Boundary("Start");
  Phase("")
  {
   R_Date("D-AMS-028064", 9886, 41);
   R_Date("D-AMS-028065", 10044, 39);
   R_Date("D-AMS-028066", 10160, 40);
```

```
  R_Date("D-AMS-028067", 10002, 45);
  R_Date("D-AMS-028068", 9828, 49);
  R_Date("D-AMS-028069", 10089, 37);
  R_Date("D-AMS-028070", 10189, 40);
  R_Date("no code 1",  10394,  39);
  R_Date("no code 2",  11146,  40);
  R_Date("no code 3",  9774,  31);
  Date("Date");
  Interval("Duration");
 };
 Boundary("End");
 };
};
```

## 2.4.13. Quebrada Santa Julia (LV. 221)

Quebrada Santa Julia (LV. 221) is an open-air site located in Chile (-28.83, -71.43), containing lithic technology (fluted projectile points) and a hearth in association with extinct fauna (*Equus sp*.)[208–210]. There are a number of strata at the site, with 37 containing the only archaeological material (see Figs. 2-3 in Jackson et al.[208]).

Bayesian modelling estimates the start of stratum 37 at 14400-12850 cal BP, with no outliers (see Supplementary Figure 74 and OxCal code).

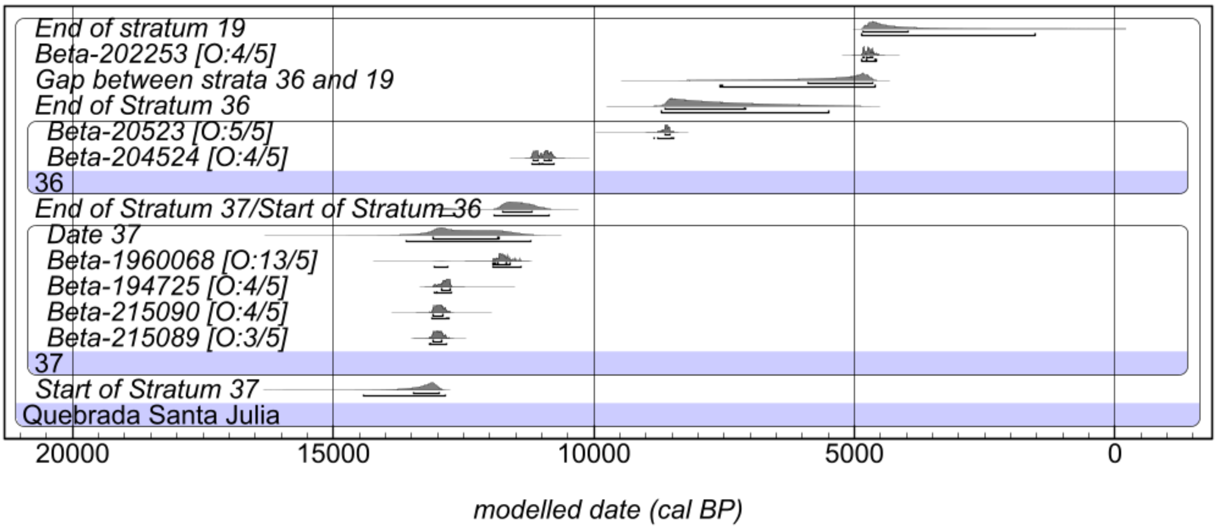


**Supplementary Figure 74.** Bayesian age model for Quebrada Santa Julia. Brackets beneath each age estimate show 68.3% and 95.4% CI. Outlier analysis output is noted as 'O:posterior probability/prior probability'.

```
Plot()
{
 Outlier_Model("General",T(5),U(0,4),"t");
 Sequence("Quebrada Santa Julia")
 {
  Boundary("Start of Stratum 37");
  Phase("37")
  {
   R_Date("Beta-215089", 11090, 80)
   {
    Outlier("General", 0.05);
   };
   R_Date("Beta-215090", 11060, 80)
   {
    Outlier("General", 0.05);
   };
   R_Date("Beta-194725", 10920, 80)
   {
    Outlier("General", 0.05);
   };
   R_Date("Beta-1960068", 10120, 50)
   {
    Outlier("General", 0.05);
   };
   Interval("Duration 37");
   Date("Date 37");
  };
  Boundary("End of Stratum 37/Start of Stratum 36");
  Phase("36")
  {
   R_Date("Beta-204524", 9640, 50)
   {
    Outlier("General", 0.05);
   };
   R_Date("Beta-20523", 7830, 40)
   {
    Outlier("General", 0.05);
   };
  };
  Boundary("End of Stratum 36");
  Boundary("Gap between strata 36 and 19");
  R_Date("Beta-202253", 4200, 40)
  {
   Outlier("General", 0.05);
  };
  Boundary("End of stratum 19");
 };
};
```

## 2.4.14. Quereo

Quereo is an open-air site located in Chile (-31.93; -71.51), containing lithic technology and extinct faunal remains (*Equus* sp.)[211,212]. There are six geological units and four cultural levels (Quereo I-IV, bottom to top) (see Fig. 3 in Ñuñez et al.[211]). Cultural evidence in Quereo I is seen as tentative by the excavators [211].

Bayesian modelling estimates the start of Quereo II at 13090-12760 cal BP, with no outliers (see Supplementary Figure 75 and OxCal code). Following Quereo II, cultural activity is evidenced at Quereo II, which is estimated to have started at 6040-2185 cal BP.

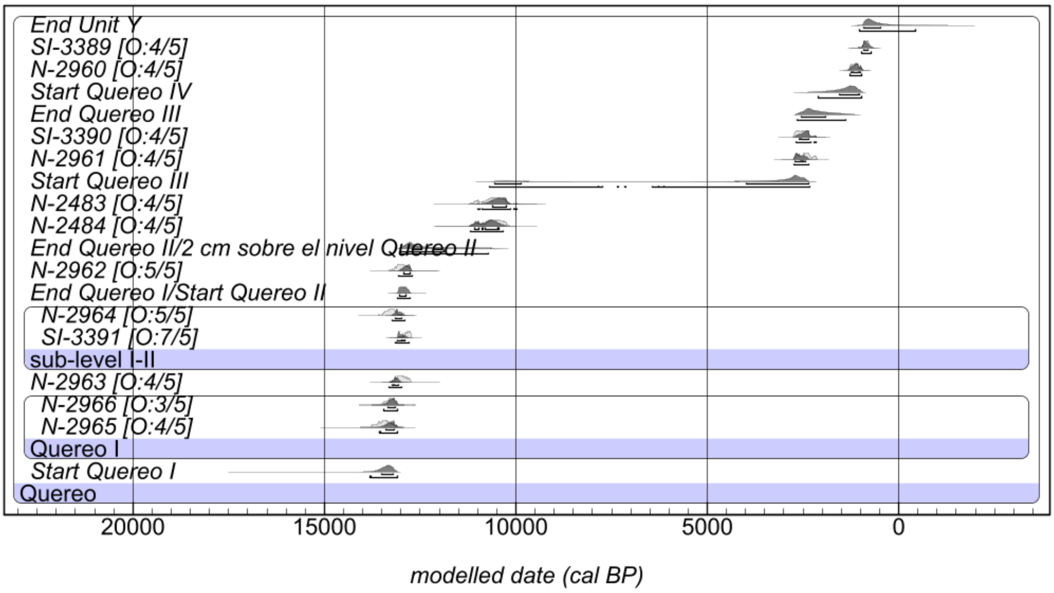


**Supplementary Figure 75.** Bayesian age model for Quereo. Brackets beneath each age estimate show 68.3% and 95.4% CI. Outlier analysis output is noted as 'O:posterior probability/prior probability'.

*2.5. Colombia*

2.5.1. El Abra

El Abra is a locality of rockshelter sites (I-IV) located in Colombia (5.020, -73.937), containing the Abriense or 'edge-trimmed tool' tradition (unifacial and poorly elaborated[9]) and faunal remains[8,213–219]. There are three units containing cultural evidence (C-E). Unit C is the most basal and is further subdivided into four subunits (C1-4). Excavation was carried out in 25 cm spits, corresponding to levels 1-9 (see Fig. 5 in Correal et al.[217]). There is no precise correspondence between the deposition units and artificial levels[216]. However, Muttillo et al.[216] correlate levels 6-9 with unit C, 3-4 with unit D, and 1-2 with unit E, with El Abra II and III containing the same sequence.

Bayesian modelling estimates the start of unit C at 14700-13030 cal BP, with no outliers (see Supplementary Figure 76 and OxCal code). GrN-5556 (12400 ± 160 BP) was not placed in unit C per Muttillo et al.[8], who found that this sample was from a sterile level with no lithic artefacts. Following unit C, cultural activity is evidenced at Unit D, which is estimated to have started at 10845-10275 cal BP.

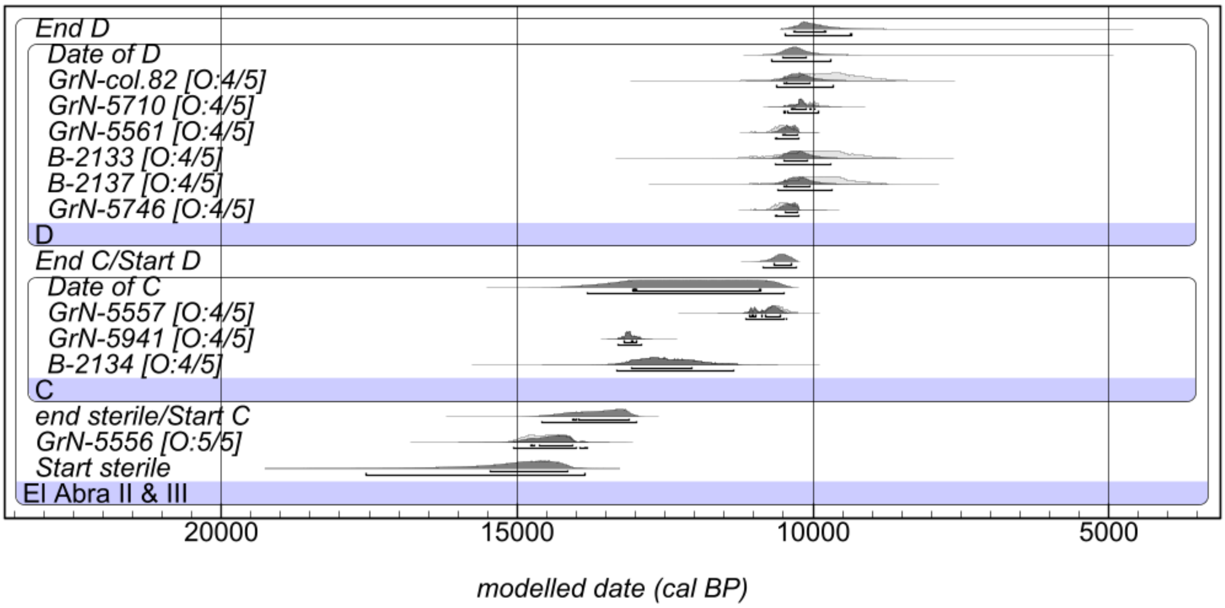


**Supplementary Figure 76.** Bayesian age model for El Abra. Brackets beneath each age estimate show 68.3% and 95.4% CI. Outlier analysis output is noted as 'O:posterior probability/prior probability'.

```
Plot()
{
 Outlier_Model("General",T(5),U(0,4),"t");
 Curve("IntCal20","intcal20.14c");
 Curve("SHCal20","shcal20.14c");
 Mix_Curve ("Mixed","IntCal20","SHCal20",U(0,100));
 Sequence("El Abra II & III")
 {
  Boundary("Start sterile");
  R_Date("GrN-5556",12400,160)
  {
   Outlier("General", 0.05);
   context="depositional unit C3, level 7";
  };
  Boundary("end sterile/Start C");
  Phase("C")
  {
   R_Date("B-2134",10720,400)
   {
    Outlier("General", 0.05);
    context="depositional unit C4";
   };
   R_Date("GrN-5941",11210,90)
   {
    Outlier("General", 0.05);
    context="SU C, level 8";
   };
   R_Date("GrN-5557",9420,110)
   {
    Outlier("General", 0.05);
    context="SU C, level 8";
   };
   Interval("Duration of C");
   Date("Date of C");
  };
  Boundary("End C/Start D");
  Phase("D")
  {
   R_Date("GrN-5746",9325,100)
   {
    Outlier("General", 0.05);
    context="depositional unit D";
   };
   R_Date("B-2137",8760,350)
   {
    Outlier("General", 0.05);
    context="depositional unit D";
   };
   R_Date("B-2133",8810,430)
   {
    Outlier("General", 0.05);
    context="depositional unit C4-D";
   };
   R_Date("GrN-5561",9340,90)
   {
    Outlier("General", 0.05);
    context="SU D, level 5";
   };
   R_Date("GrN-5710",9025,90)
   {
    Outlier("General", 0.05);
    context="SU D, level 4";
   };
   R_Date("GrN-col.82",8670,400)
   {
    Outlier("General", 0.05);
    context="SU D, level 4";
   };
   Interval("Duration of D");
   Date("Date of D");
  };
  Boundary("End D");
 };
 Difference("Difference C and D","end sterile/Start C","End C/Start D");
 Sequence()
 {
  Boundary("=end sterile/Start C");
  Interval("Duration C to D");
  Date("Date C to D");
  Boundary("=End D");
 };
};
```

## 2.5.2. La Palestina 2 (05YON002-02)

La Palestina 2 is an open-air site located in Colombia (6.716, -74.383), containing lithic technology[17,220]. There are a number of geological horizons (AP, A, AB, Bt, C), with Bt showing the earliest human evidence at 35-60 cm (see Fig. 5.6. in Lopez[17]).

A single-phase Bayesian model estimates the start of cultural occupation at 18135-11975 cal BP (or 13935-12125 cal BP at 68.3% CI; see Supplementary Figure 77 and OxCal code). Since there are no dated preceding levels, the distribution likely overextends in time.

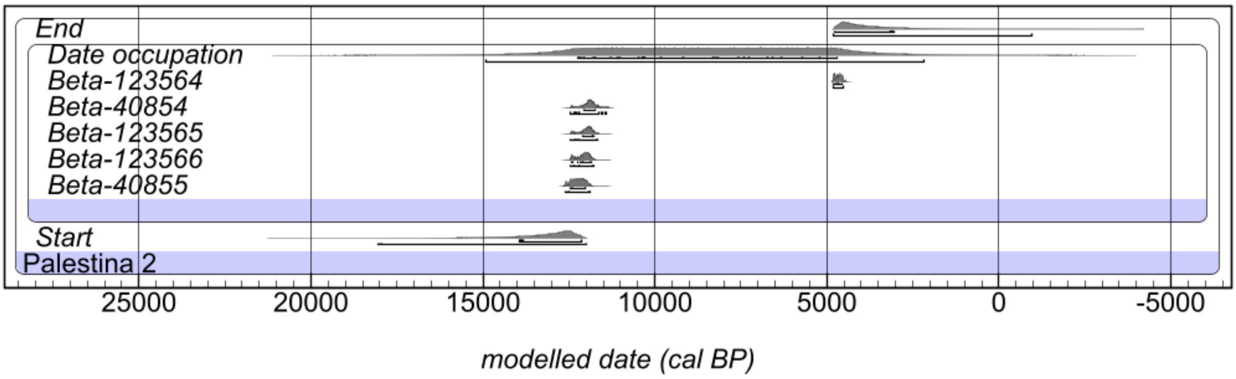


**Supplementary Figure 77.** Bayesian age model for La Palestina 2. Brackets beneath each age estimate show 68.3% and 95.4% CI. Outlier analysis output is noted as 'O:posterior probability/prior probability'.

```
Plot()
{
 Curve("IntCal20","intcal20.14c");
 Curve("SHCal20","shcal20.14c");
 Mix_Curve ("Mixed","IntCal20","SHCal20",U(0,100));
 Sequence("Palestina 2")
 {
  Boundary("Start");
  Phase("")
  {
   R_Date("Beta-40855",10400,90);
   R_Date("Beta-123566",10300,70);
   R_Date("Beta-123565",10260,70);
   R_Date("Beta-40854",10230,80);
   R_Date("Beta-123564",4140,40);
   Date("Date occupation");
  };
  Boundary("End");
 };
};
```

2.5.3. Nare (005PNA005)

Nare is an open-air site located in Colombia (6.35, -74.1), containing lithic technology[17,220]. There are a number of geological horizons (A, AB, Bt, and C), with AB showing the earliest human evidence at 30-45 cm, in 5 cm spits (numbered levels 1-16).

A single-phase Bayesian model estimates the start of cultural occupation at 20210-12055 cal BP (or 14615-12175 cal BP at 68.3% CI; see Supplementary Figure 78 and OxCal code). Since there are no dated preceding levels, the distribution likely overextends in time.

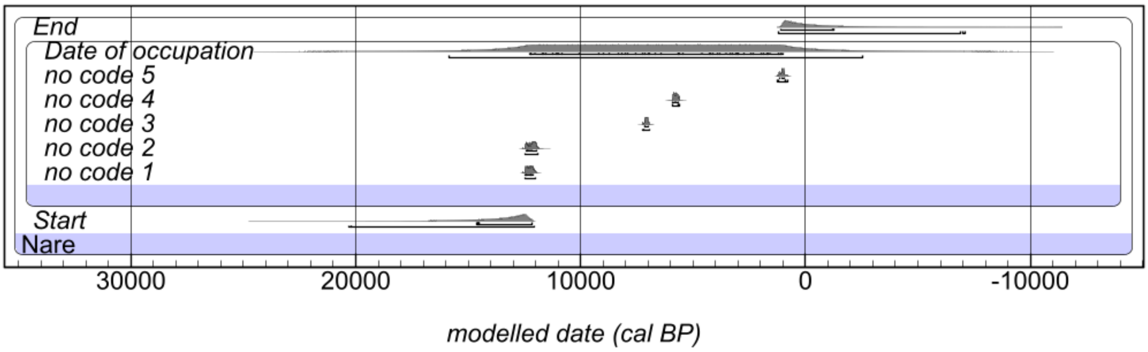


**Supplementary Figure 78.** Bayesian age model for Nare. Brackets beneath each age estimate show 68.3% and 95.4% CI. Outlier analysis output is noted as 'O:posterior probability/prior probability'.

```
Plot()
{
 Curve("IntCal20","intcal20.14c");
 Curve("SHCal20","shcal20.14c");
 Mix_Curve ("Mixed","IntCal20","SHCal20",U(0,100));
 Sequence("Nare")
 {
  Boundary("Start");
  Phase("")
  {
   R_Date("no code 1",10400,40);
   R_Date("no code 2",10350,60);
   R_Date("no code 3",6200,40);
   R_Date("no code 4",5040,60);
   R_Date("no code 5",1120,70);
   Date("Date of occupation");
  };
  Boundary("End");
 };
};
```

2.5.4. San Juan de Bedout (05PBE014)

San Juan de Bedout is an open-air site located in Colombia (6.5, -74.5), containing lithic technology[17,220]. There are two geological horizons, A and B, with the latter showing the earliest evidence of human occupation and a single radiocarbon age of 12595-11832 cal BP (10350 ± 90 BP; at 40 cm).

### 2.5.5. Serranía La Lindosa

Serranía La Lindosa is a 20 $km^2$ rocky outcrop in Colombia (approximately 2.5, -72.9) where multiple rockshelters (Cerro Azul, Cerro Montoya and Limoncillos) containing lithic technology and rock pictographs are located. The only site with >1 dates, Cerro Azul (2.5298, -72.8664) has a stratigraphy of seven strata (see Fig. 4 in Morcote-Ríos et al.[221], with stratum II containing the earliest human evidence. Strata assignment for each radiocarbon date is unclear, with only the two earliest dates (Beta-492721 and -489157; geological) noted as belonging to stratum II.

A single-phase Bayesian model using the only two ACR-YD-aged cultural dates for Cerro Azul estimates the start of cultural occupation at 13965-11820 cal BP (see Supplementary Figure 79 and OxCal code). Dates for Cerro Montoya and Limoncillos were entered into the modelling as single ages.

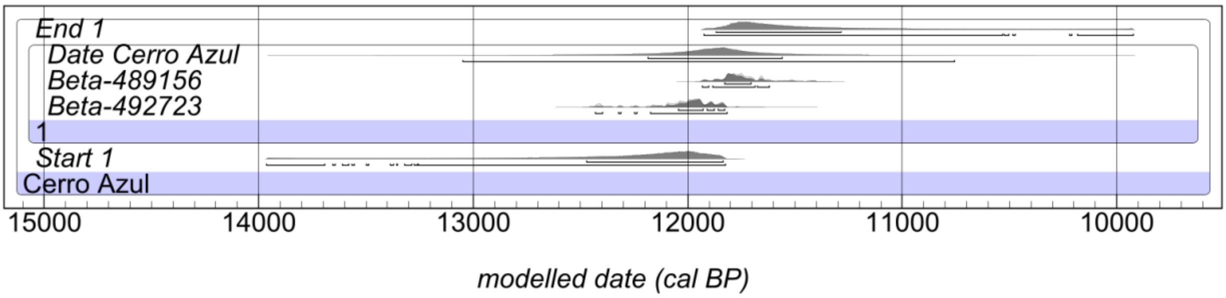


**Supplementary Figure 79.** Bayesian age model for Cerro Azul. Brackets beneath each age estimate show 68.3% and 95.4% CI. Outlier analysis output is noted as ‘O:posterior probability/prior probability’.

```
Plot()
{
 Sequence("Cerro Azul")
 {
  Boundary("Start 1");
  Phase("1")
  {
   R_Date("Beta-492723", 10280, 40);
   R_Date("Beta-489156", 10130, 30);
   Date("Date Cerro Azul");
  };
  Boundary("End 1");
 };
};
```

### 2.5.6. Tequendama

Tequendama is a locality of rockshelter sites (1-2) located in Colombia (4.533, -74.275; 2,570 m.a.s.l.), containing a lithic technology called ‘Tequendamiense’ and faunal remains[7,213,220,222–224]. There are nine stratigraphic levels (see Fig. 17 in Ch. 3 of Correal Urrego and van der Hammen[9]), with stratum 5B showing the earliest cultural evidence. As argued in Aceituno and Rojas-Mora[7], differences between Tequendamiense and Abriense technologies include the ‘use of allochthonous materials and the presence of scrapers, thinning flakes and a projectile point fragment in the former’. Both are viewed to represent evidence of hunting and butchering activities.

Bayesian modelling estimates the start of stratum 5-7 at 13580-12525 cal BP, 11695-10975 cal BP and 11090-7950 cal BP, respectively (see Supplementary Figure 80 and OxCal code). There are only two outliers at 60% posterior probability (GrN-7478 and an age without lab code at 6080 BP) in

stratum 7. Both are likely understimates caused by vertical mixing. Following stratum 7, cultural activity is evidenced in stratum 8, which is estimated to have started at 8165-6545 cal BP.

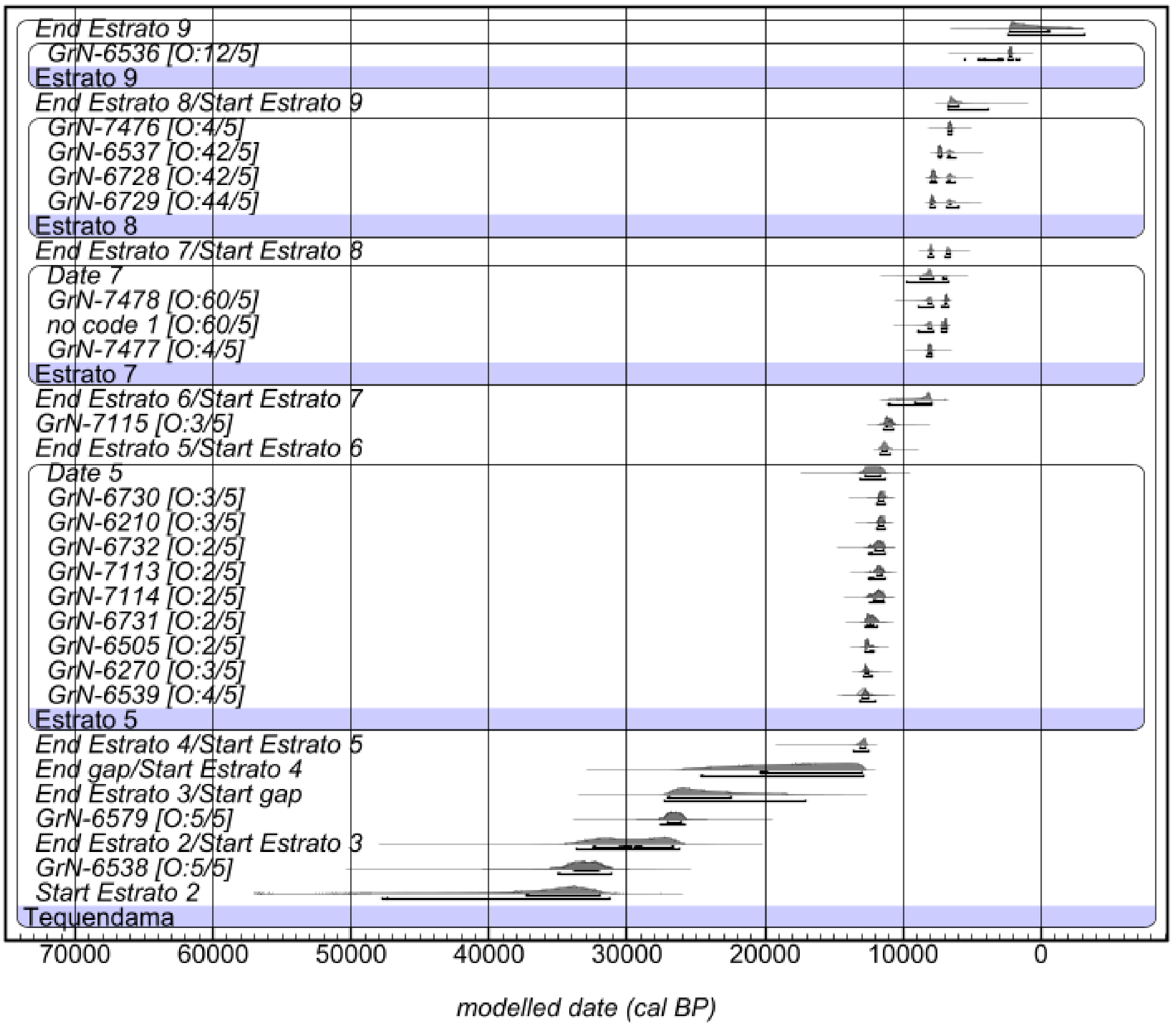


**Supplementary Figure 80.** Bayesian age model for Tequendama. Brackets beneath each age estimate show 68.3% and 95.4% CI. Outlier analysis output is noted as ‘O:posterior probability/prior probability’.

```
Plot()
{
 Outlier_Model("General",T(5),U(0,4),"t");
 Sequence("Tequendama")
 {
  Boundary("Start Estrato 2");
  R_Date("GrN-6538",28890,840)
  {
   Outlier("General", 0.05);
   site="TEQ1";
   sample="Col'172";
   estrato="2";
   material="charcoal";
  };
  Boundary("End Estrato 2/Start Estrato 3");
  R_Date("GrN-6579",22250,470)
  {
   Outlier("General", 0.05);
   site="TEQ1";
   sample="Col'178";
   estrato="3";
   material="charcoal";
  };
  Boundary("End Estrato 3/Start gap");
  Interval("Duration of gap");
  Boundary("End gap/Start Estrato 4");
  Boundary("End Estrato 4/Start Estrato 5");
  Phase("Estrato 5")
  {
   R_Date("GrN-6539",10920,260)
   {
    Outlier("General", 0.05);
    site="TEQ1";
    sample="Col'170";
    estrato="5 a/b";
    material="charcoal";
   };
   R_Date("GrN-6270",10730,105)
   {
    Outlier("General", 0.05);
    site="TEQ1";
    sample="Col'166";
    estrato="5b";
    material="charcoal";
   };
   R_Date("GrN-6505",10590,90)
   {
    Outlier("General", 0.05);
    site="TEQ1";
    sample="Col'167";
    estrato="5b";
    material="charcoal";
   };
   R_Date("GrN-6731",10460,130)
   {
    Outlier("General", 0.05);
    site="TEQ1";
    sample="Col'169";
    estrato="5b";
    material="charcoal";
   };
   R_Date("GrN-7114",10150,150)
   {
    Outlier("General", 0.05);
    site="TEQ1";
    sample="Col'175";
    estrato="5b o 5/6";
    material="charcoal";
   };
   R_Date("GrN-7113",10140,100)
   {
    Outlier("General", 0.05);
    site="TEQ1";
    sample="Col'168";
    estrato="5b o 5/6";
    material="charcoal";
   };
   R_Date("GrN-6732",10130,150)
   {
    Outlier("General", 0.05);
    site="TEQ1";
    sample="Col'176";
    estrato="5b o 5/6";
    material="charcoal";
   };
   R_Date("GrN-6210",10025,95)
   {
    Outlier("General", 0.05);
    site="TEQ1";
    sample="Col'165";
    estrato="5b o 5/6";
    material="charcoal";
   };
   R_Date("GrN-6730",9990,100)
   {
    Outlier("General", 0.05);
    site="TEQ1";
    sample="Col'174";
    estrato="5b o 5/6";
    material="charcoal";
   };
   Interval("Duration 5");
   Date("Date 5");
  };
  Boundary("End Estrato 5/Start Estrato 6");
  R_Date("GrN-7115",9740,135)
  {
   Outlier("General", 0.05);
   site="TEQ1";
   sample="Col'171";
   estrato="6";
   material="charcoal";
  };
  Boundary("End Estrato 6/Start Estrato 7");
  Phase("Estrato 7")
  {
   R_Date("GrN-7477",7235,60)
   {
    Outlier("General", 0.05);
    site="TEQ1";
    sample="Col'181";
    estrato="7";
    entierro="12";
    material="human bone";
   };
   R_Date("no code 1",6080,40)
   {
    Outlier("General", 0.05);
    sample="Col AAA";
    estrato="7A";
    material="human bone";
   };
   R_Date("GrN-7478",6020,45)
   {
    Outlier("General", 0.05);
    site="TEQ1";
    sample="Col'182";
    estrato="7";
    material="human bone";
   };
   Date("Date 7");
   Interval("Duration 7");
  };
  Boundary("End Estrato 7/Start Estrato 8");
  Phase("Estrato 8")
  {
   R_Date("GrN-6729",7090,75)
   {
    Outlier("General", 0.05);
    site="TEQ1";
    sample="Col'164";
    estrato="8a";
    material="charcoal";
   };
   R_Date("GrN-6728",6990,110)
   {
    Outlier("General", 0.05);
    site="TEQ1";
    sample="Col'163";
    estrato="8a";
    material="charcoal";
   };
   R_Date("GrN-6537",6395,70)
   {
    Outlier("General", 0.05);
    site="TEQ1";
    sample="Col'161";
    estrato="8 a/b";
    material="charcoal";
   };
   R_Date("GrN-7476",5805,50)
   {
    Outlier("General", 0.05);
    site="TEQ1";
    sample="Col'180";
    estrato="8";
    entierro="7";
    material="human bone";
   };
  };
  Boundary("End Estrato 8/Start Estrato 9");
  Phase("Estrato 9")
  {
   R_Date("GrN-6536",2225,35)
   {
    Outlier("General", 0.05);
    site="TEQ1";
    sample="Col'159";
    estrato="9";
    material="charcoal";
   };
  };
  Boundary("End Estrato 9");
 };
 Difference("Difference 5 and 6","End Estrato 4/Start Estrato 5","End Estrato 5/Start Estrato 6");
 Difference("Difference 6 and 7","End Estrato 5/Start Estrato 6","End Estrato 6/Start Estrato 7");
 Difference("Difference 5 and 7","End Estrato 4/Start Estrato 5","End Estrato 6/Start Estrato 7");
 Sequence()
 {
  Boundary("=End Estrato 5/Start Estrato 6");
  Interval("Duration 6");
  Date("Date 6");
  Boundary("=End Estrato 6/Start Estrato 7");
 };
};
```

## 2.5.7. Tibitó 1 & 2

Tibitó 1 & 2 are open-air sites found in the same locality (600 m from each other) within Colombia (4.98, -73.98), containing Abriense lithic technology and extinct faunal remains [*Notiomastodon platensis*, *Equus* (*Amerhippus*) *lasallei*, and *Cuvieronius hyodon*][213,216,219,224,225]. There are seven strata, with stratum 3 containing the earliest human evidence. There is a single date at the site, 13805-

13342 cal BP (GrN-9375; 11740 ± 110 BP), which was obtained on faunal bone remains within stratum 3-A.

## *2.6. Peru*

### 2.6.1. Cuncaicha

Cuncaicha is a high-altitude (4,480 masl) rockshelter located in Peru (-15.373, -72.618)[226–228]. Described as a lithic workshop, the site includes projectile point technology and faunal remains. Cuncaicha contains five distinct strata, with stratum 5 corresponding to the terminal Pleistocene (see Fig. S3 in Rademaker et al.[226]). Nearby (7 km), archaeologists found a second site named Pucuncho (open-air workshop at 4,355 masl). Here, two fluted fishtail projectile point bases made of local materials (andesite and obsidian) were found. There are no direct dates for the second archaeological site. In 2018, Rademaker and colleagues published data on a series of human burials found at the site, dated to the Holocene[228–230].

Using data from Rademaker et al.[226] (excluding Holocene-aged human burials), Bayesian modelling estimates the start of the terminal Pleistocene component at 12065-11775 cal BP, with outliers (see Supplementary Figure 81 and OxCal code). The replicates Beta-297423 and AA-94254 fail a $\chi^2$ test and, as such, are significantly different ($\chi^2$ = 9.492; df = 1; p = 3.8). This likely denotes pretreatment issues, with the oldest, ultrafiltered collagen date (AA 94254) probably being more reliable. When combined, however, the age is not outlier within the sequence. The 12065-11775 cal BP estimate is comparable to that presented for the same period by Meinekat et al.[227], at 12275–11780 cal BP. Following the terminal Pleistocene component, cultural activity is evidenced during the early Holocene event, which is estimated to have started at 9645-9315 cal BP.

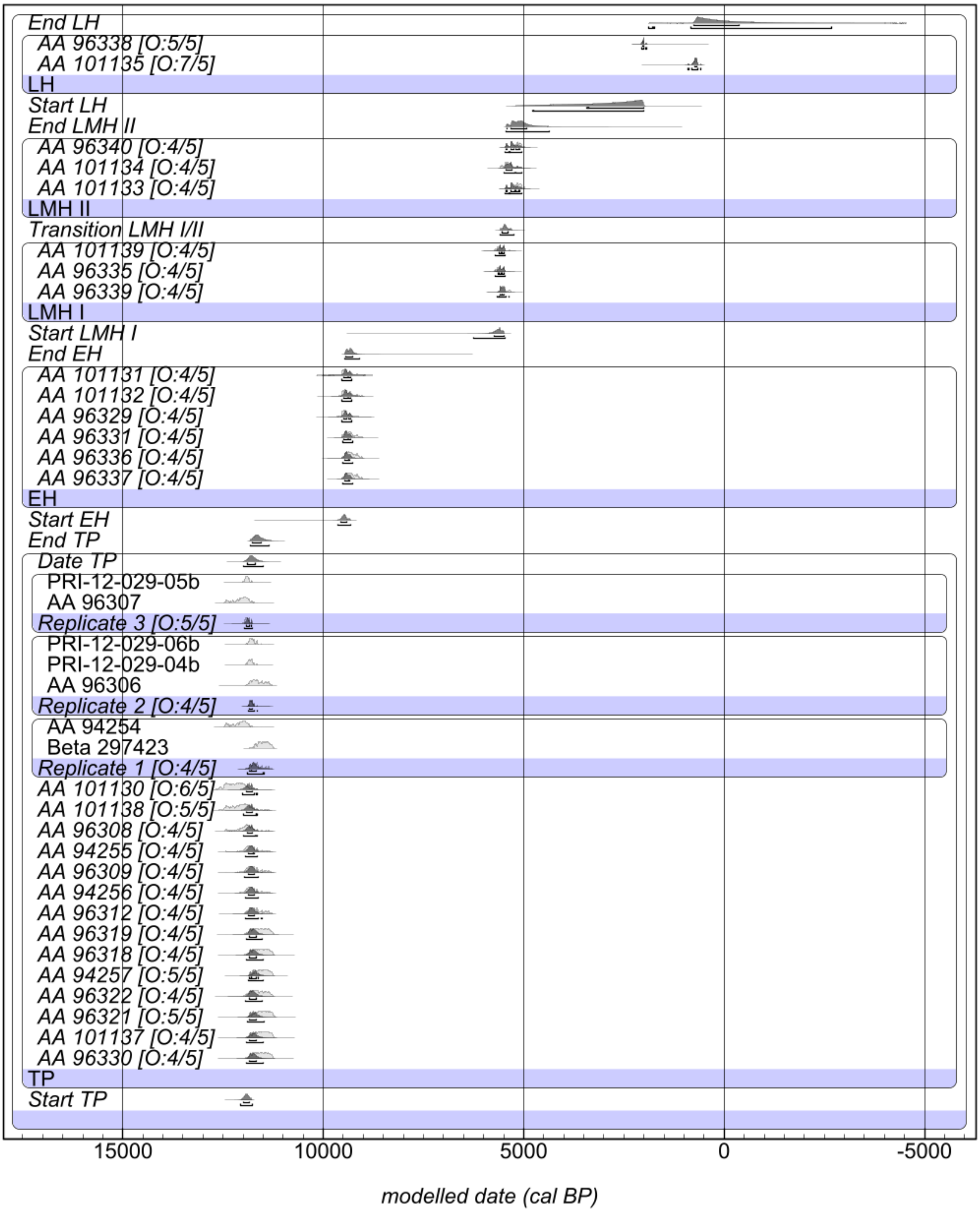


**Supplementary Figure 81.** Bayesian age model for Cuncaicha. Brackets beneath each age estimate show 68.3% and 95.4% CI. Outlier analysis output is noted as 'O:posterior probability/prior probability'.

```
Plot()
{
 Outlier_Model("General",T(5),U(0,4),"t");
 Outlier_Model("SSimple",N(0,2),0,"s");
 Curve("SHCal20","shcal20.14c");
 Sequence()
 {
  Boundary("Start TP");
  Phase("TP")
  {
   R_Date("AA 96330",10086,97)
   {
    Outlier("General", 0.05);
    context=Component TP, U.1-2, L.6;
   };
   R_Date("AA 101137",10060,100)
   {
    Outlier("General", 0.05);
    context=Component TP, U.7, L.8, F.12-3;
   };
   R_Date("AA 96321",10034,97)
   {
    Outlier("General", 0.05);
    context=Component TP, U.2, L.8b;
   };
   R_Date("AA 96322",10127,98)
   {
    Outlier("General", 0.05);
    context=Component TP, U.2, L.8b;
   };
   R_Date("AA 94257",10055,67)
   {
    Outlier("General", 0.05);
    context=Component TP, U.2, L.9;
   };
   R_Date("AA 96318",10084,99)
   {
    Outlier("General", 0.05);
    context=Component TP, U.2, L.9;
   };
   R_Date("AA 96319",10100,99)
   {
    Outlier("General", 0.05);
    context=Component TP, U.2, L.9;
   };
   R_Date("AA 96312",10163,71)
   {
    Outlier("General", 0.05);
    context=Component TP, U.2, L.11;
   };
   R_Date("AA 94256",10200,69)
   {
    Outlier("General", 0.05);
    context=Component TP, U.2, L.11;
   };
   R_Date("AA 96309",10189,77)
   {
    Outlier("General", 0.05);
    context=Component TP, U.2, L.12;
   };
   R_Date("AA 94255",10211,69)
   {
    Outlier("General", 0.05);
    context=Component TP, U.2, L.12;
   };
   R_Date("AA 96308",10260,72)
   {
    Outlier("General", 0.05);
    context=Component TP, U.2, L.13;
   };
   R_Date("AA 101138",10310,100)
   {
    Outlier("General", 0.05);
    context=Component TP, U.7, L.10, F.12-4;
   };
   R_Date("AA 101130",10380,100)
   {
    Outlier("General", 0.05);
    context=Component TP, U.7. L.10, F.12-4;
   };
   R_Combine("Replicate 1")
   {
    R_Date("Beta 297423",10050,50)
    {
     Outlier("SSimple", 0.05);
     context=Component TP, U.2, L.13;
     notes=duplicate of AA 94254;
    };
    R_Date("AA 94254",10321,73)
    {
     Outlier("SSimple", 0.05);
     context=Component TP, U.2, L.13;
     notes=duplicate of Beta 297423;
    };
    Outlier("General", 0.05);
   };
   R_Combine("Replicate 2")
   {
    R_Date("AA 96306",10132,71)
    {
     Outlier("SSimple", 0.05);
     context=Component TP, U.2, L.13;
     notes=replicate of PRI-12-029-04b and PRI-12-029-06b;
    };
    R_Date("PRI-12-029-04b",10205,35)
    {
     Outlier("SSimple", 0.05);
     context=Component TP, U.2, L.13;
     notes=replicate of 96306 and PRI-12-029-06b;
    };
    R_Date("PRI-12-029-06b",10180,40)
    {
     Outlier("SSimple", 0.05);
     context=Component TP, U.2, L.13;
     notes=replicate of PRI-12-029-04b and AA 96306;
    };
    Outlier("General", 0.05);
   };
   R_Combine("Replicate 3")
   {
    R_Date("AA 96307",10306,72)
    {
     Outlier("SSimple", 0.05);
     context=Component TP, U.2, L.13;
     notes=duplicate of PRI-12-029-05b;
    };
    R_Date("PRI-12-029-05b",10265,35)
    {
     Outlier("SSimple", 0.05);
     context=Component TP, U.2, L.13;
     notes=duplicate of AA 96307;
    };
    Outlier("General", 0.05);
   };
   Interval("Duration of TP");
   Interval("Duration TP");
   Date("Date TP");
  };
  Boundary("End TP");
  Boundary("Start EH");
  Phase("EH")
  {
   R_Date("AA 96337",8363,82)
   {
    Outlier("General", 0.05);
    context=Component EH, U.1, L.4b;
    notes=;
   };
   R_Date("AA 96336",8361,82)
   {
    Outlier("General", 0.05);
    context=Component EH, U.2, L.4;
   };
   R_Date("AA 96331",8404,82)
   {
    Outlier("General", 0.05);
    context=Component EH, U.1-2, L.6;
   };
   R_Date("AA 96329",8483,83)
   {
    Outlier("General", 0.05);
    context=Component EH, U.1-2, L.6;
   };
   R_Date("AA 101132",8454,84)
   {
    Outlier("General", 0.05);
    context=Component EH, U.7. L.7, F.12-2;
   };
   R_Date("AA 101131",8461,85)
   {
    Outlier("General", 0.05);
    context=Component EH, U.7. L.7, F.12-2;
   };
  };
  Boundary("End EH");
  Boundary("Start LMH I");
  Phase("LMH I")
  {
   R_Date("AA 96339",4826,58)
   {
    Outlier("General", 0.05);
    context=Component LMH I, U.2, L.3;
   };
   R_Date("AA 96335",4898,66)
   {
    Outlier("General", 0.05);
    context=Component LMH I, U.2, L.4;
   };
   R_Date("AA 101139",4890,66)
   {
    Outlier("General", 0.05);
    context=Component LMH I, U.7. L.5;
    notes=;
   };
  };
  Boundary("Transition LMH I/II");
  Phase("LMH II")
  {
   R_Date("AA 101133",4584,59)
   {
    Outlier("General", 0.05);
    context=Component LMH II, U.6, L.4-5, F. 12-1;
   };
   R_Date("AA 101134",4683,73)
   {
    Outlier("General", 0.05);
    context=Component LMH II, U.6, L.4-5, F. 12-1;
   };
   R_Date("AA 96340",4599,57)
   {
    Outlier("General", 0.05);
    context=Component LMH II, U.2. L.3;
   };
  };
  Boundary("End LMH II");
  Boundary("Start LH");
  Phase("LH")
  {
   R_Date("AA 101135",841,45)
   {
    Outlier("General", 0.05);
    context=Component LH II, U.7, L.3;
   };
   R_Date("AA 96338",2098,4)
   {
    Outlier("General", 0.05);
    context=Component LH I, U.2, L.3;
   };
  };
  Boundary("End LH");
 };
};
```

2.6.2. Guitarrero Cave

Guitarrero Cave is a high-elevation (2,580 m) cave site located in Peru (-9.20069, -77.7098), containing lithic technology (bifacial) and fibre-based artefacts like cordage and textiles[231–237]. Lynch[233] defined two late Pleistocene/early Holocene cultural complexes at the site (I & II), but further analysis by Jolie et al.[231] deemed these to represent a single group.

A single-phase Bayesian model including direct ages on twined textiles and cordage from Complex II obtained by Joile et al. (2011) estimates the start at 12685-11755 cal BP (see Supplementary Figure 82 and OxCal code). AA-81780 was excluded as a clear outlier at 2210 38 BP—corroborating natural and cultural disturbances noted by archaeologists[231]. An alternate multi-phase model was created using previously published (pre Jolie et al.[231]) measurements, combining Complex I and II ages into a single `Phase` (given conclusions by Jolie et al[231]). This estimates the start of Complexes I and II to 14865-11885 cal BP with five outliers, only one of these being within the phase in question [GX-1859, 'charcoal (pooled?)'; a likely overestimate; see Supplementary Figure 83 and OxCal code]. These further denote vertical mixing observed at the site. Start estimates for both models are statistically comparable, however (Supplementary Figure 84). Following I and II, cultural activity is evidenced in Complex III, which is estimated to have started at 8980-2005 cal BP (bimodal, with the highest distribution centred at around 2100 cal BP).

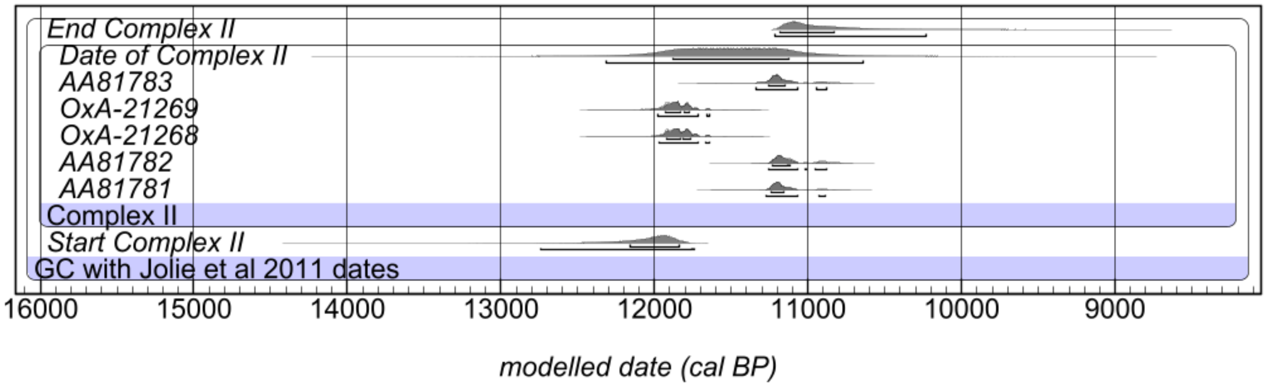


**Supplementary Figure 82.** Bayesian age model for Guitarrero Cave (using direct ages on twined textiles and cordage from Complex II). Brackets beneath each age estimate show 68.3% and 95.4% CI.

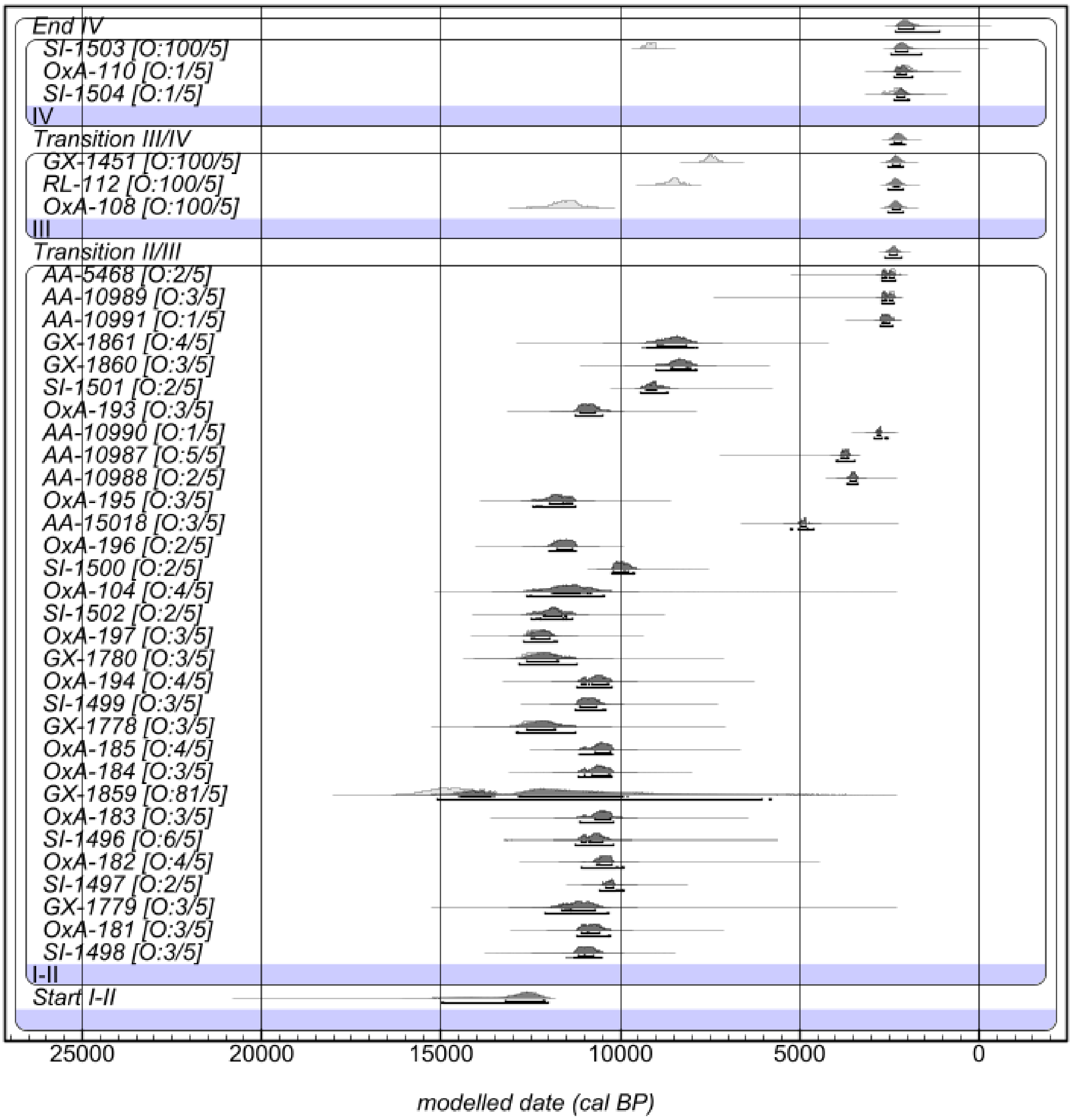


**Supplementary Figure 83.** Bayesian age model for Guitarrero Cave (using pre-Jolie et al.[231] data). Brackets beneath each age estimate show 68.3% and 95.4% CI. Outlier analysis output is noted as 'O:posterior probability/prior probability'.

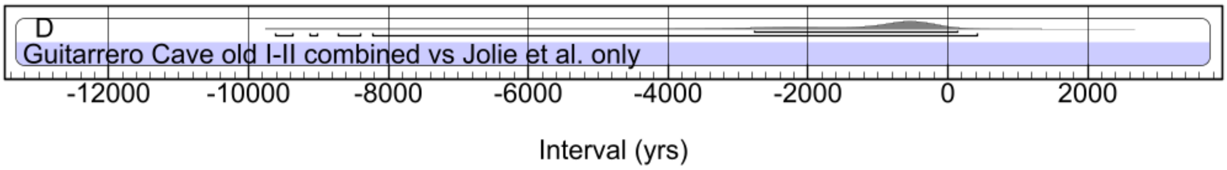


**Supplementary Figure 84.** Probability density function for the difference ('D') between the start of cultural activity at Guitarrero Cave direct ages on twined textiles and cordage from Complex II (Supplementary Figure 82) vs pre-Jolie et al.[231] data (Supplementary Figure 83). These results suggest that there is no significant difference between the modelled outputs, as the distributions include zero at 95.4% CI (black bracket beneath).

```
Plot()
{
 Curve("SHCal20","shcal20.14c");
 Sequence("GC with Jolie et al 2011 dates")
 {
  Boundary("Start Complex II");
  Phase("Complex II")
  {
   R_Date("AA81781",9797,59)
   {
    context="Complex II, square C6, unit 156";
    material="cordage/textile";
   };
   R_Date("AA81782",9767,58)
   {
    context="Complex II, square C6, unit 159";
    material="cordage/textile";
   };
   R_Date("OxA-21268",10230,45)
   {
    context="Complex II, square C6, unit 159A";
    material="cordage/textile";
   };
   R_Date("OxA-21269",10240,45)
   {
    context="Complex II, square C6, unit 159B";
    material="cordage/textile";
   };
   R_Date("AA81783",9813,70)
   {
    context="Complex II, square C6, unit 159B";
    material="cordage/textile";
   };
   Interval("Duration of Complex II");
   Date("Date of Complex II");
  };
  Boundary("End Complex II");
 };
 //excluded date
 // R_Date("AA81780",2210,38)
 // {
 // context="Complex II, square C5, unit 124";
 // material="cordage/textile";
 // };
 //};
};


Plot()
{
 Outlier_Model("General",T(5),U(0,4),"t");
 Curve("SHCal20","shcal20.14c");
 Sequence()
 {
  Boundary("Start I-II");
  Phase("I-II")
  {
   R_Date("SI-1498",9660,150)
   {
    Outlier("General", 0.05);
    context="Complex I, square B1/A2, unit 26";
    material="charcoal (pooled?)";
   };
   R_Date("OxA-181",9520,150)
   {
    Outlier("General", 0.05);
    context="Complex I, square B1/A2, unit 26";
    material="charcoal";
   };
   R_Date("GX-1779",9790,240)
   {
    Outlier("General", 0.05);
    context="Complex I, square B1/A2, unit 28";
    material="charcoal (pooled?)";
   };
   R_Date("SI-1497",9140,90)
   {
    Outlier("General", 0.05);
    context="Complex I, square B2 N1/2, unit 59";
    material="charcoal (pooled?)";
   };
   R_Date("OxA-182",9280,150)
   {
    Outlier("General", 0.05);
    context="Complex I, square B2 N1/2, unit 60";
    material="charcoal";
   };
   R_Date("SI-1496",9475,130)
   {
    Outlier("General", 0.05);
    context="Complex I, square B2 N1/2, unit 62";
    material="charcoal (pooled?)";
   };
   R_Date("OxA-183",9340,150)
   {
    Outlier("General", 0.05);
    context="Complex I, square B2 N1/2, unit 62";
    material="charcoal";
   };
   R_Date("GX-1859",12560,360)
   {
    Outlier("General", 0.05);
    context="Complex I, square B2 N1/2, unit 63";
    material="charcoal (pooled?)";
   };
   R_Date("OxA-184",9400,150)
   {
    Outlier("General", 0.05);
    context="Complex I, square B2 N1/2, unit 63";
    material="charcoal";
   };
   R_Date("OxA-185",9350,150)
   {
    Outlier("General", 0.05);
    context="Complex I, square B2 N1/2, unit 64";
    material="charcoal";
   };
   R_Date("GX-1778",10535,290)
   {
    Outlier("General", 0.05);
    context="Complex IIa, square B1/A2, unit  22";
    material="charcoal (pooled?)";
   };
   R_Date("SI-1499",9580,135)
   {
    Outlier("General", 0.05);
    context="Complex IIa, square B1/A2, unit 22";
    material="charcoal (pooled?)";
   };
   R_Date("OxA-194",9430,150)
   {
    Outlier("General", 0.05);
    context="Complex IIa, square B1/A2, unit 22";
    material="charcoal";
   };
   R_Date("GX-1780",10475,300)
   {
    Outlier("General", 0.05);
    context="Complex IIa, square C6, unit  159";
    material="charcoal (pooled?)";
   };
   R_Date("OxA-197",10445,130)
   {
    Outlier("General", 0.05);
    context="Complex IIa, square C6, unit  159";
    material="cordage";
   };
   R_Date("SI-1502",10240,110)
   {
    Outlier("General", 0.05);
    context="Complex IIa, square C6, unit 159";
    material="charcoal (pooled?)";
   };
   R_Date("OxA-104",9930,300)
   {
    Outlier("General", 0.05);
    context="Complex IIb, square C5, unit 123";
    material="wood batten";
   };
   R_Date("SI-1500",8910,90)
   {
    Outlier("General", 0.05);
    context="Complex IIc, square B1/A2, unit 20";
    material="charcoal (pooled?)";
   };
   R_Date("OxA-196",10085,120)
   {
    Outlier("General", 0.05);
    context="Complex IIc, square C5, unit 122";
    material="cordage";
   };
   R_Date("AA-15018",4337,55)
   {
    Outlier("General", 0.05);
    context="Complex IId, square B1/A2, unit 19";
    material="noncarbonized seed";
   };
   R_Date("OxA-195",10180,130)
   {
    Outlier("General", 0.05);
    context="Complex IId, square C6, unit 150";
    material="bipointed wood dowel";
   };
   R_Date("AA-10988",3325,55)
   {
    Outlier("General", 0.05);
    context="Complex IId,( square, B5, unit107)";
    material="noncarbonized seed";
   };
   R_Date("AA-10987",3495,50)
   {
    Outlier("General", 0.05);
    context="Complex IId,( square, B5, unit, 107)";
    material="noncarbonized seed";
   };
   R_Date("AA-10990",2695,55)
   {
    Outlier("General", 0.05);
    context="Complex IId,( square, C6,, unit, 146)";
    material="noncarbonized seed";
   };
   R_Date("OxA-193",9600,130)
   {
    Outlier("General", 0.05);
    context="Complex IIe, square B1/A2, unit 18";
    material="charcoal";
   };
   R_Date("SI-1501",8175,95)
   {
    Outlier("General", 0.05);
    context="Complex IIe, square B1/A2, unit 18";
    material="charcoal (pooled?)";
   };
   R_Date("GX-1860",7575,220)
   {
    Outlier("General", 0.05);
    context="Complex IIe, square B1/A2, unit 18";
    material="charcoal (pooled?)";
   };
   R_Date("GX-1861",7680,280)
   {
    Outlier("General", 0.05);
    context="Complex IIe, square C6, unit 146";
    material="charcoal (pooled?)";
   };
   R_Date("AA-10991",2540,50)
   {
    Outlier("General", 0.05);
    context="Complex IIe, square C6, unit 146";
    material="noncarbonized seed";
   };
   R_Date("AA-10989",2455,50)
   {
    Outlier("General", 0.05);
    context="Complex IIe, square, C6, unit, 144)";
    material="noncarbonized seed";
   };
   R_Date("AA-5468",2430,60)
   {
    Outlier("General", 0.05);
    context="Complex IIe, square, C6, unit, 144)";
    material="noncarbonized seed";
   };
   Interval("Duration of I-II");
  };
  Boundary("Transition II/III");
  Phase("III")
  {
   R_Date("OxA-108",10000,200)
   {
    Outlier("General", 0.05);
    context="Complex III, square B2 S1/2, unit 35";
```

```
   material="wood dowel";
  };
  R_Date("RL-112",7730,150)
  {
   Outlier("General", 0.05);
   context="Complex III, square B3, unit 82";
   material="pooled charcoal";
  };
  R_Date("GX-1451",6610,160)
  {
   Outlier("General", 0.05);
   context="Complex III, square, B6, unit, 132)";
   material="pooled charcoal";
  };
 };
 Boundary("Transition III/IV");
 Phase("IV")
 {
  R_Date("SI-1504",2315,125)
  {
   Outlier("General", 0.05);
   context="Complex IV, square B2 N1/2, unit 47";
   material="wood firedrill hearth";
  };
  R_Date("OxA-110",2150,150)
  {
   Outlier("General", 0.05);
   context="Complex IV, square B2 N1/2, unit 47";
   material="wood firedrill hearth";
  };
  R_Date("SI-1503",8225,90)
  {
   Outlier("General", 0.05);
   context="Complex IV, square B2 N1/2, unit 47/48";
   material="pooled charcoal";
  };
 };
 Boundary("End IV");
 };
};
```

## 2.6.3. Huaca Prieta

Huaca Prieta is a large stone and earthen mound located in Peru (-7.9241, -79.3069), containing lithic technology (unifacial), faunal and malachological remains at pre-mound components[238–243]. Late Pleistocene/early Holocene cultural evidence is within units 15/21, 9, 12, (Test Pit) 22, HP-3 and 2[238,239] (see Fig. 2 in Dillehay et al.[239]). Near Huaca Prieta, the sites of Paredones (600 m north; Unit 12) and Unit 16 (50 m north) also contain early cultural material[239].

Following Dillehay et al[239], a Bayesian model was built using all measurements (n=16) from early Holocene/late Pleistocene layers at the Huaca Prieta and associated sites. This estimates the start of cultural activity in the area at 16695-15070 cal BP, with two outliers (AA-86930 and AA-84168) within a later Holocene component (see Supplementary Figure 85 and OxCal code). The age range of this component is estimated at 15757-9994 cal BP, overlapping the ACR-YD period. Following the late Pleistocene/early Holocene, cultural activity is evidenced at a Holocene layer, which is estimated to have started at 8860-7935 cal BP.

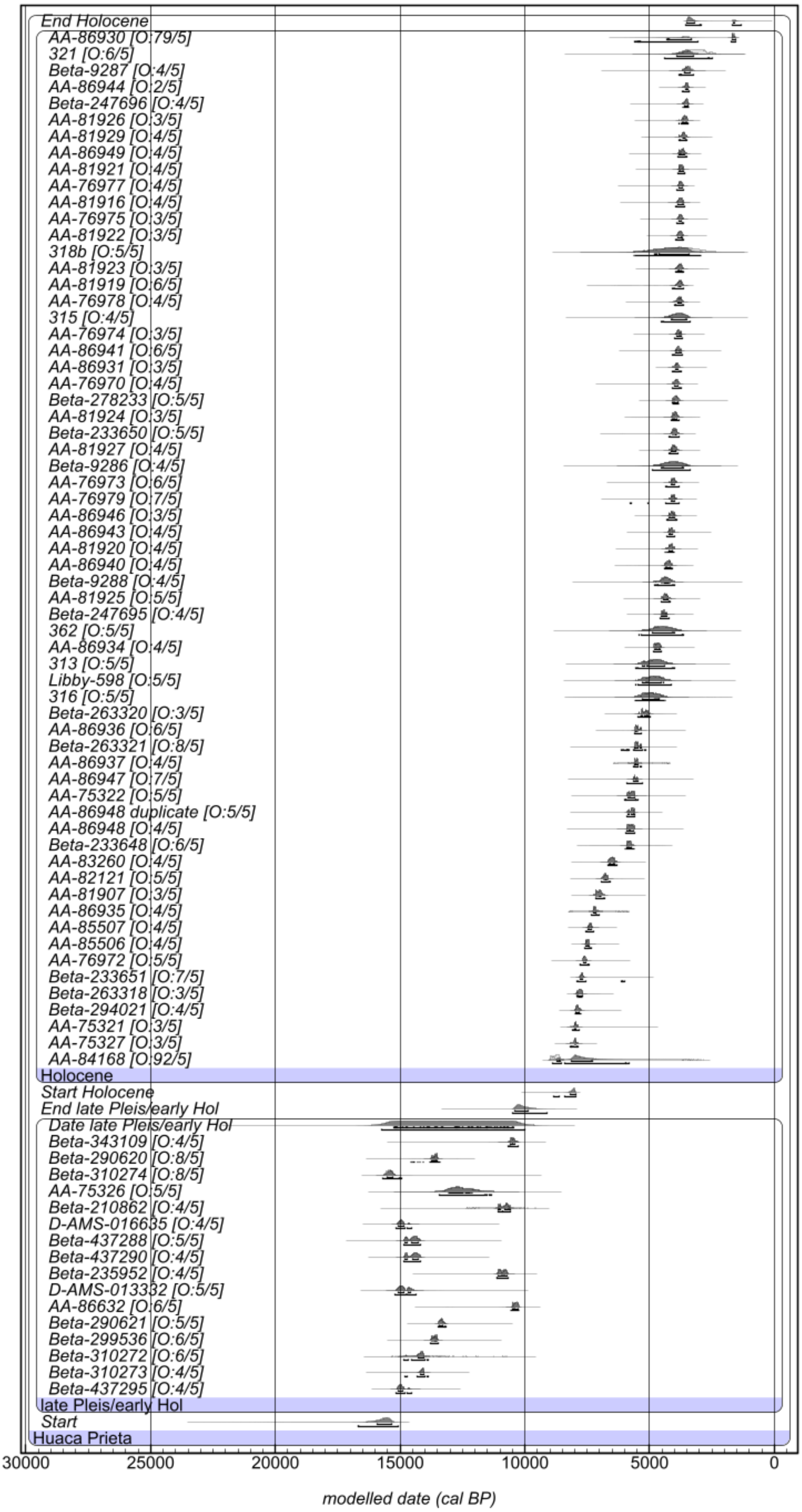


**Supplementary Figure 85.** Bayesian age model for Huaca Prieta. Brackets beneath each age estimate show 68.3% and 95.4% CI. Outlier analysis output is noted as 'O:posterior probability/prior probability'.

```
Plot()
{
 Outlier_Model("General",T(5),U(0,4),"t");
 Curve("SHCal20","shcal20.14c");
 Sequence("Huaca Prieta")
 {
  Boundary("Start");
  Phase("late Pleis/early Hol")
  {
   R_Date("Beta-437295",12610,40)
   {
    Outlier("General", 0.05);
   };
   R_Date("Beta-310273",12240,50)
   {
    Outlier("General", 0.05);
   };
   R_Date("Beta-310272",12280,60)
   {
    Outlier("General", 0.05);
   };
   R_Date("Beta-299536",11800,50)
   {
    Outlier("General", 0.05);
   };
   R_Date("Beta-290621",11500,50)
   {
    Outlier("General", 0.05);
   };
   R_Date("AA-86632",9230,40)
   {
    Outlier("General", 0.05);
   };
   R_Date("D-AMS-013332",12594,62)
   {
    Outlier("General", 0.05);
   };
   R_Date("Beta-235952",9580,40)
   {
    Outlier("General", 0.05);
   };
   R_Date("Beta-437290",12410,40)
   {
    Outlier("General", 0.05);
   };
   R_Date("Beta-437288",12420,40)
   {
    Outlier("General", 0.05);
   };
   R_Date("D-AMS-016635",12602,35)
   {
    Outlier("General", 0.05);
      };
   R_Date("Beta-210862",9530,50)
   {
    Outlier("General", 0.05);
   };
   R_Date("AA-75326",10770,340)
   {
    Outlier("General", 0.05);
   };
   R_Date("Beta-310274",12950,50)
   {
    Outlier("General", 0.05);
   };
   R_Date("Beta-290620",11780,50)
   {
    Outlier("General", 0.05);
   };
   R_Date("Beta-343109",9330,40)
   {
    Outlier("General", 0.05);
   };
   Date("Date late Pleis/early Hol");
   Interval("Duration late Pleis/early Hol");
  };
  Boundary("End late Pleis/early Hol");
  Interval("Duration transition");
  Boundary("Start Holocene");
  Phase("Holocene")
  {
   R_Date("AA-84168",7956,50)
   {
    Outlier("General", 0.05);
   };
   R_Date("AA-75327",7226,44)
   {
    Outlier("General", 0.05);
   };
   R_Date("AA-75321",7195,45)
   {
    Outlier("General", 0.05);
   };
   R_Date("Beta-294021",7110,50)
   {
    Outlier("General", 0.05);
   };
   R_Date("Beta-263318",7000,50)
   {
    Outlier("General", 0.05);
   };
   R_Date("Beta-233651",6920,30)
   {
    Outlier("General", 0.05);
   };
   R_Date("AA-76972",6797,48)
   {
    Outlier("General", 0.05);
   };
   R_Date("AA-85506",6641,49)
   {
    Outlier("General", 0.05);
   };
   R_Date("AA-85507",6522,54)
   {
    Outlier("General", 0.05);
   };
   R_Date("AA-86935",6310,33)
   {
    Outlier("General", 0.05);
   };
   R_Date("AA-81907",6170,45)
   {
    Outlier("General", 0.05);
   };
   R_Date("AA-82121",5980,40)
   {
    Outlier("General", 0.05);
   };
   R_Date("AA-83260",5750,60)
   {
    Outlier("General", 0.05);
   };
   R_Date("Beta-233648",5110,40)
   {
    Outlier("General", 0.05);
   };
   R_Date("AA-86948",5059,72)
   {
    Outlier("General", 0.05);
   };
   R_Date("AA-86948 duplicate",5020,35)
   {
    Outlier("General", 0.05);
   };
   R_Date("AA-75322",5018,86)
   {
    Outlier("General", 0.05);
   };
   R_Date("AA-86947",4898,49)
   {
    Outlier("General", 0.05);
   };
   R_Date("AA-86937",4849,31)
   {
    Outlier("General", 0.05);
   };
   R_Date("Beta-263321",4790,40)
   {
    Outlier("General", 0.05);
   };
   R_Date("AA-86936",4783,31)
   {
    Outlier("General", 0.05);
   };
   R_Date("Beta-263320",4590,40)
   {
    Outlier("General", 0.05);
   };
   R_Date("316",4380,220)
   {
    Outlier("General", 0.05);
   };
   R_Date("Libby-598",4298,230)
   {
    Outlier("General", 0.05);
   };
   R_Date("313",4257,250)
   {
    Outlier("General", 0.05);
   };
   R_Date("AA-86934",4181,34)
   {
    Outlier("General", 0.05);
   };
   R_Date("362",4044,300)
   {
    Outlier("General", 0.05);
   };
   R_Date("Beta-247695",4000,40)
   {
    Outlier("General", 0.05);
   };
   R_Date("AA-81925",3964,41)
   {
    Outlier("General", 0.05);
   };
   R_Date("Beta-9288",3960,100)
   {
    Outlier("General", 0.05);
   };
   R_Date("AA-86940",3875,30)
   {
    Outlier("General", 0.05);
   };
   R_Date("AA-81920",3810,41)
   {
    Outlier("General", 0.05);
   };
   R_Date("AA-86943",3806,28)
   {
    Outlier("General", 0.05);
   };
   R_Date("AA-86946",3783,41)
   {
    Outlier("General", 0.05);
   };
   R_Date("AA-76979",3758,40)
   {
    Outlier("General", 0.05);
   };
   R_Date("AA-76973",3748,40)
   {
    Outlier("General", 0.05);
   };
   R_Date("Beta-9286",3730,300)
   {
    Outlier("General", 0.05);
   };
   R_Date("AA-81927",3728,40)
   {
    Outlier("General", 0.05);
   };
   R_Date("Beta-233650",3700,40)
   {
    Outlier("General", 0.05);
   };
   R_Date("AA-81924",3687,40)
   {
    Outlier("General", 0.05);
   };
   R_Date("Beta-278233",3660,40)
   {
    Outlier("General", 0.05);
   };
   R_Date("AA-76970",3649,36)
   {
    Outlier("General", 0.05);
   };
   R_Date("AA-86931",3638,29)
   {
    Outlier("General", 0.05);
```

```
};
R_Date("AA-86941",3599,29)
{
Outlier("General", 0.05);
};
R_Date("AA-76974",3588,36)
{
Outlier("General", 0.05);
};
R_Date("315",3572,220)
{
Outlier("General", 0.05);
};
R_Date("AA-76978",3567,40)
{
Outlier("General", 0.05);
};
R_Date("AA-81919",3557,40)
{
Outlier("General", 0.05);
};
R_Date("AA-81923",3556,44)
{
Outlier("General", 0.05);
};
R_Date("318b",3550,600)
{
Outlier("General", 0.05);
};
R_Date("AA-81922",3547,40)
{
Outlier("General", 0.05);
};
R_Date("AA-76975",3535,35)
{
Outlier("General", 0.05);
};
R_Date("AA-81916",3534,53)
{
Outlier("General", 0.05);
};
R_Date("AA-76977",3530,36)
{
Outlier("General", 0.05);
};
R_Date("AA-81921",3508,40)
{
Outlier("General", 0.05);
};
R_Date("AA-86949",3467,39)
{
Outlier("General", 0.05);
};
R_Date("AA-81929",3441,39)
{
Outlier("General", 0.05);
};
R_Date("AA-81926",3394,40)
{
Outlier("General", 0.05);
};
R_Date("Beta-247696",3350,40)
{
Outlier("General", 0.05);
};
R_Date("AA-86944",3334,38)
{
Outlier("General", 0.05);
};
R_Date("Beta-9287",3270,100)
{
Outlier("General", 0.05);
};
R_Date("321",2966,340)
{
Outlier("General", 0.05);
};
R_Date("AA-86930",1760,29)
{
Outlier("General", 0.05);
};
};
Boundary("End Holocene");
};
};
```

## 2.6.5. Pampa de los Fósiles and Ascope

Pampa de los Fósiles and Ascope are open-air, surface sites located near each other in Peru (approximately -7.60, -79.29), containing lithic technology of the Paiján tradition[34–36]. As such, a single-phase Bayesian model containing eleven charcoal dates associated with this tradition was built. This estimates the start of cultural activity at 13500-11695 cal BP (see Supplementary Figure 86 and OxCal code).

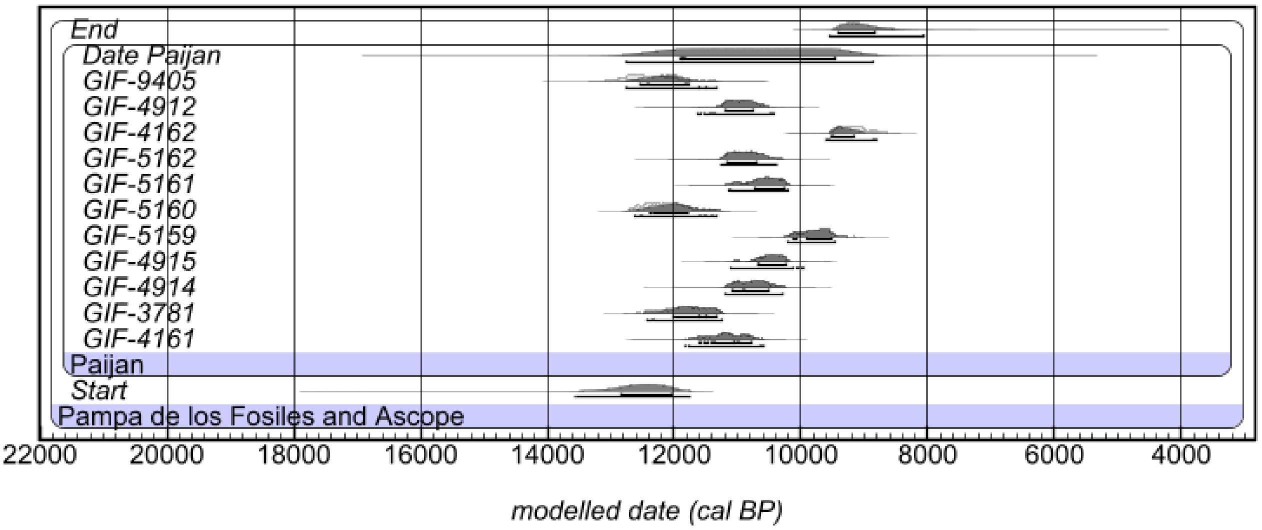


**Supplementary Figure 86.** Bayesian age model for Pampa de los Fósiles and Ascope. Brackets beneath each age estimate show 68.3% and 95.4% CI. Outlier analysis output is noted as ‘O:posterior probability/prior probability’.

```
Plot()
{
 Curve("SHCal20","shcal20.14c");
 Sequence("Pampa de los Fosiles and Ascope")
 {
  Boundary("Start");
  Phase("Paijan")
  {
   R_Date("GIF-4161",9810,180)
   {
    site="Pampa de los Fósiles";
   };
   R_Date("GIF-3781",10200,180)
   {
    site="Pampa de los Fósiles";
   };
   R_Date("GIF-4914",9490,170)
   {
    site="Pampa de los Fósiles";
   };
   R_Date("GIF-4915",9300,170)
   {
    site="Pampa de los Fósiles";
   };
   R_Date("GIF-5159",8730,160)
   {
    site="Pampa de los Fósiles";
   };
   R_Date("GIF-5160",10380,170)
   {
    site="Pampa de los Fósiles";
   };
   R_Date("GIF-5161",9360,170)
   {
    site="Pampa de los Fósiles";
   };
   R_Date("GIF-5162",9600,170)
   {
    site="Pampa de los Fósiles";
   };
   R_Date("GIF-4162",8260,160)
   {
    site="Pampa de los Fósiles";
   };
   R_Date("GIF-4912",9670,170)
   {
    site="Ascope";
   };
   R_Date("GIF-9405",10640,260)
   {
    site="Pampa de los Fósiles";
   };
   Date("Date Paijan");
   Interval("Duration Paijan");
  };
  Boundary("End");
 };
};
```

2.6.6. Quebrada Jaguay (QJ-280)

Quebrada Jaguay (QJ-280) is an open-air site located in Peru (-16.527, -72.856), containing lithic technology, fiber-based artefacts, and faunal remains[244–250]. The site is divided into four sectors (I-IV) and contains late Pleistocene (Jaguay Phase) and early Holocene (Machas Phase). Sector I, located on a high terrace, contains four stratigraphic levels with 4c presenting the earliest evidence of human occupation (see Fig. 3, Jones et al.[247]).

Bayesian modelling using Sector I data estimates the start of level 4c at 14595-12355 cal BP, 3 at 12480-11275 cal BP, and 2 at 11195-10280 cal BP (see Supplementary Figure 87 and OxCal code). There are no outliers. Following level 2, cultural activity is evidenced at level 1, which is estimated to have started at 8780-8375 cal BP.

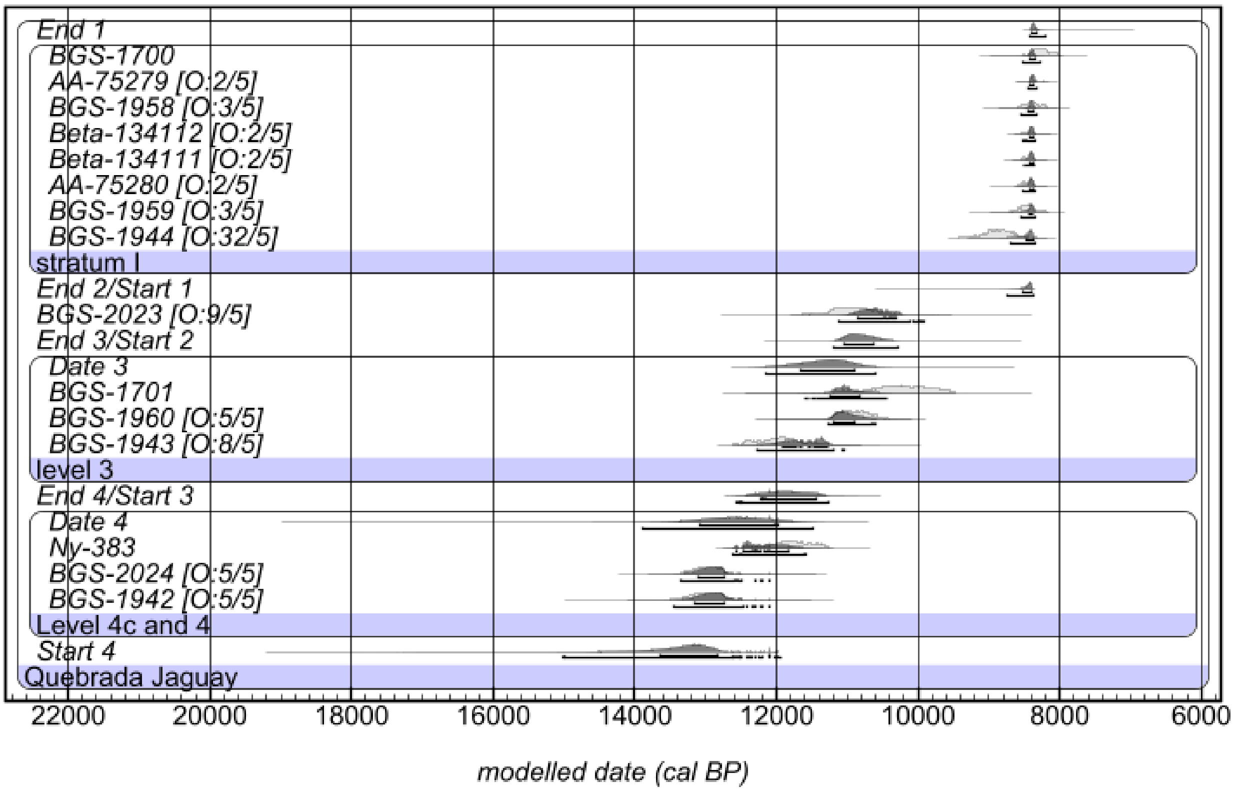


**Supplementary Figure 87.** Bayesian age model for Quebrada Jaguay. Brackets beneath each age estimate show 68.3% and 95.4% CI. Outlier analysis output is noted as ‘O:posterior probability/prior probability’.

```
Plot()
{
 Curve("SHCal20","shcal20.14c");
 Outlier_Model("General",T(5),U(0,4),"t");
 Sequence("Quebrada Jaguay")
 {
  Boundary("Start 4");
  Phase("Level 4c and 4")
  {
   R_Date("BGS-1942",11105,260)
   {
    Outlier("General",0.05);
    context="Sector I, stratum I-2-D level 4c";
    material="charcoal";
   };
   R_Date("BGS-2024",11088,220)
   {
    Outlier("General",0.05);
    context="Sector I, stratum I-2-B level 4c";
    material="charcoal";
   };
   R_Date("Ny-383",10200,140)
   {
    context="Sector I, stratum 1970 layer 4";
    material="charcoal";
   };
   Date("Date 4");
   Interval("Duration 4");
  };
  Boundary("End 4/Start 3");
  Phase("level 3")
  {
   R_Date("BGS-1943",10274,125)
   {
    Outlier("General",0.05);
    context="Sector I, stratum I-2-D level 3b";
    material="charcoal";
   };
   R_Date("BGS-1960",9597,135)
   {
    Outlier("General",0.05);
    context="Sector I, stratum I-3-B feature 9";
    material="charcoal";
   };
   R_Date("BGS-1701",9120,300)
   {
    context="Sector I, stratum 1992 level 3";
    material="charcoal";
   };
   Date("Date 3");
   Interval("Duration 3");
  };
  Boundary("End 3/Start 2");
  R_Date("BGS-2023",9657,220)
  {
   Outlier("General",0.05);
   context="Sector I, stratum I-2-B level 2a";
   material="charcoal";
  };
  Boundary("End 2/Start 1");
  Phase("stratum I")
  {
   R_Date("BGS-1944",8053,115)
   {
    Outlier("General",0.05);
    context="Sector I, stratum I-3-B level 1f";
    material="charcoal";
   };
   R_Date("BGS-1959",7690,100)
   {
    Outlier("General",0.05);
    context="Sector I, stratum I-3-B level 1b";
    material="charcoal";
   };
   R_Date("AA-75280",7670,56)
   {
    Outlier("General",0.05);
    context="Sector I, stratum I-3-B level 1a2";
    material="plant remains";
   };
   R_Date("Beta-134111",7660,50)
   {
    Outlier("General",0.05);
    context="Sector I, stratum I-3-B level 1d";
    material="gourd fragment";
   };
   R_Date("Beta-134112",7650,50)
   {
    Outlier("General",0.05);
    context=Sector I, stratum I-3-B level 1c;
    material=gourd fragment;
   };
   R_Date("BGS-1958",7620,100)
   {
    Outlier("General",0.05);
    context="Sector I, stratum I-3-B level 1e";
    material="charcoal";
   };
   R_Date("AA-75279",7599,42)
   {
    Outlier("General",0.05);
    context="Sector I, stratum I-3-B level 1a2";
    material="plant remains";
   };
   R_Date("BGS-1700",7500,130)
   {
    context="Sector I, stratum 1992 level 1b";
    material="charcoal";
   };
  };
  Boundary("End 1");
 };
 Difference("Difference 4 and 3","Start 4","End 4/Start 3");
 Difference("Difference 3 and 2","End 4/Start 3","End 3/Start 2");
 Difference("Difference 4 and 2","Start 4","End 3/Start 2");
 Sequence()
 {
  Boundary("=End 3/Start 2");
  Interval("Duration 2");
  Date("Date 2");
  Boundary("=End 2/Start 1");
 };
};
```

## 2.6.7. Quebrada Tacahuay

Quebrada Tacahuay is an open-air site located in Peru (-17.13, -71.01), containing lithic technology (unifacial) and faunal remains[244,251–253]. There are nine stratigraphic units as defined by sedimentological and cultural evidence, with 8B lower containing the earliest cultural occupation (see Figs. 2, 6 and Table 1 in DeFrance and Álvarez[253]). Underlying units 8C-9 are culturally sterile, whilst overlying unit 7, consisting of flooding debris from an ENSO event, sealed the early human signal[253].

Bayesian modelling estimates the start of unit 8 at 12915-12515 cal BP and unit 5 at 12280-11300 cal BP (see Supplementary Figure 88 and OxCal code). There are no outliers. Following unit 8, cultural activity is evidenced at unit 4, which is estimated to have started at 11295-9715 cal BP (or 10815-9955 at 68.3% CI).

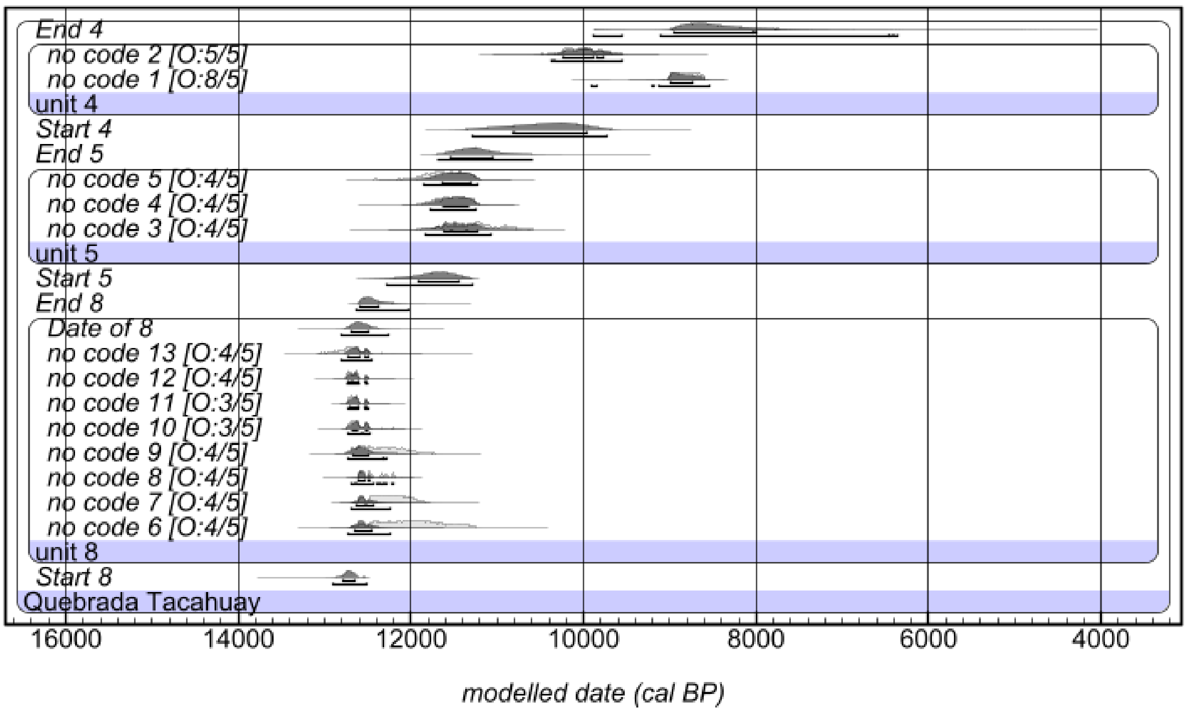


**Supplementary Figure 88.** Bayesian age model for Quebrada Tacahuay. Brackets beneath each age estimate show 68.3% and 95.4% CI. Outlier analysis output is noted as 'O:posterior probability/prior probability'.

```
Plot()
{
 Curve("SHCal20","shcal20.14c");
 Outlier_Model("General",T(5),U(0,4),"t");
 Sequence("Quebrada Tacahuay")
 {
  Boundary("Start 8");
  Phase("unit 8")
  {
   R_Date("no code 6",10290,200)
   {
    Outlier("General", 0.05);
    context="Perfil 1A-Unidad 8";
    material="charcoal";
   };
   R_Date("no code 7",10420,110)
   {
    Outlier("General", 0.05);
    context="Perfil 1A-Unidad 8";
    material="charcoal";
   };
   R_Date("no code 8",10510,50)
   {
    Outlier("General", 0.05);
    context="Perfil 3B-Unidad 8";
    material="charcoal";
   };
   R_Date("no code 9",10530,140)
   {
    Outlier("General", 0.05);
    context="Perfil 3A-Unidad 8";
    material="charcoal";
   };
   R_Date("no code 10",10660,80)
   {
    Outlier("General", 0.05);
    context="Bloque 3 Unidad 8B bajo";
    material=charcoal;
   };
   R_Date("no code 11",10690,60)
   {
    Outlier("General", 0.05);
    context="Perfil 1A Unidad 8 (1998)";
    material="charcoal";
   };
   R_Date("no code 12",10750,80)
   {
    Outlier("General", 0.05);
    context="Perfil 1-Unidad 8";
    material="charcoal";
   };
   R_Date("no code 13",10770,150)
   {
    Outlier("General", 0.05);
    context="Perfil 1-Unidad 8";
    material="charcoal";
   };
   Interval("Duration of 8");
   Date("Date of 8");
  };
  Boundary("End 8");
  Boundary("Start 5");
  Phase("unit 5")
  {
   R_Date("no code 3",9850,150)
   {
    Outlier("General", 0.05);
    context="Bloque 3 Unidad 5B";
    material="charcoal";
   };
   R_Date("no code 4",10050,90)
   {
    Outlier("General", 0.05);
    context="Perfil 3C Unidad 5B";
    material="charcoal";
   };
   R_Date("no code 5",10090,130)
   {
    Outlier("General", 0.05);
    context="Perfil 3B-Unidad 5";
    material="charcoal";
   };
  };
  Boundary("End 5");
  Boundary("Start 4");
  Phase("unit 4")
  {
   R_Date("no code 1",7990,80)
   {
    Outlier("General", 0.05);
    context="Perfil 4 Unidad 4c3";
    material="charcoal";
   };
   R_Date("no code 2",9010,140)
   {
    Outlier("General", 0.05);
    context="Bloque 2 Unidad 4";
    material="charcoal";
   };
  };
  Boundary("End 4");
 };
};
```

## 2.6.8. The Ring Site

The Ring Site is an open-air shell ring on top of middens located in Peru (-17.13, -71.01), containing lithic technology and faunal remains[254]. There are a number of units, including the originally excavated (in 1983) Units A and B, which fall into three Groups of stratigraphic sequences (with

groups 1 and 2 having correlated levels). Unit C, which is representative of Group 1 (also including Units B, D, L, and M), consists of 15 levels. Level 16 in Unit B is the most basal level that has been radiocarbon dated. All samples sent for measurement are the product of flotation.

Excluding the radiocarbon date in TP1 (PITT-0142)—given the tenuous correlation between this and the other units—a single-sequence Bayesian model estimates the start of occupation at 19990-11275 cal BP (or 13565-11480 cal BP at 68.3% CI), with no outliers (see Supplementary Figure 89 and OxCal code). A ΔR of 40±126 was applied to the marine gastropod age. This was calculated using Merino-Campos et al.[205] data for the north upwelling coastal region (18-30°S) of Chile (a nearby latitude range), but updated using the Marine20 curve[206] through calib.org[207] (with the collection date set to the first year of the quoted range).

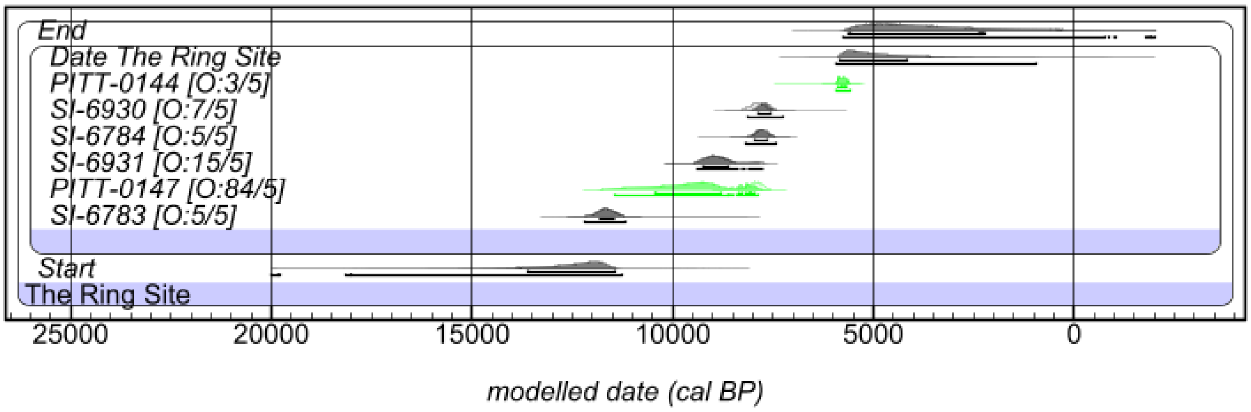


**Supplementary Figure 89.** Bayesian age model for The Ring Site. Brackets beneath each age estimate show 68.3% and 95.4% CI. Green distributions represent atmospheric measurements, with those in grey marine ages. measurements.Outlier analysis output is noted as 'O:posterior probability/prior probability'.

```
// Delta_R values updated for Marine20
Plot()
{
 Outlier_Model("General",T(5),U(0,4),"t");
 Sequence("The Ring Site")
 {
  Boundary("Start");
  Sequence("")
  {
   Curve("Marine20","marine20.14c");
   Delta_R("north upwelling region", 40, 126);
   R_Date("SI-6783",10575,105)
   {
    Outlier("General", 0.05);
    material="marine shell";
    context="Unit B, Level 16";
   };
   Curve("SHCal20","shcal20.14c");
   R_Date("PITT-0147",7155,180)
   {
    Outlier("General", 0.05);
    material="floated charcoal (small sample)";
    context="Unit M, Level 12 (between Unit B's 15 and 16)";
   };
   Curve("=Marine20");
   Delta_R("=north upwelling region");
   R_Date("SI-6931",8755,120)
   {
    Outlier("General", 0.05);
    material="marine shell";
    context="Unit B. Level 7";
   };
   R_Date("SI-6784",7675,60)
   {
    Outlier("General", 0.05);
    material="marine shell";
    context="Unit B, Level 6";
   };
   R_Date("SI-6930",7810,105)
   {
    Outlier("General", 0.05);
    material="marine shell";
    context="Unit B, Level 5";
   };
   Curve("=SHCal20");
   R_Date("PITT-0144",5060,65)
   {
    Outlier("General", 0.05);
    material="charcoal";
    context="Unit J, Level 8 (base of shell ring)";
   };
   Date("Date The Ring Site");
   Interval("Duration The Ring Site");
  };
  Boundary("End");
 };
};
```

*2.7. Uruguay*

2.7.1. K87 (Arroyo del Tigre)

K87 (Arroyo del Tigre) is an open-air site located in Uruguay (coordinates approximated at -30.4, -57.8), containing ceramics and lithic technology (including Fishtail and Tigre)[24,255–257]. No faunal remains were recovered at the site. There are six stratigraphic units as defined by sedimentological and cultural evidence (see Fig. 4 in Suárez et al.[255]). The earliest bifacial lithics are found at the interface of SU1 and SU2, continuing through SU2 and to the base of SU3. Pottery and unifacial lithic tools are found within SU4. A Fishtail point was found at the base of SU2 (associated to ages UCIAMS-125379 and -125393), whilst Tigre points were discovered mid-SU2, in level 11B (associated to ages UCIAMS-145431, -145435 and -125385).

Bayesian modelling of K87 estimates the start of SU1-SU2 interface at 14000-13175 cal BP, SU2 base (Fishtail) at 13175-12795 cal BP, and SU2 middle at 12565-11840 cal BP (Tigre), with no overlap between Fishtail and Tigre components at 95% CI (see Supplementary Figure 90 and OxCal code). There is one outlier, UCIAMS-125397 (soil organic matter), located within SU4. Given a reported low carbon yield, it is possible that this age is discrepant due to modern carbon contamination. Following SU2 middle, cultural activity is evidenced at SU2 upper, which is estimated to have started at 10850-9445 cal BP.

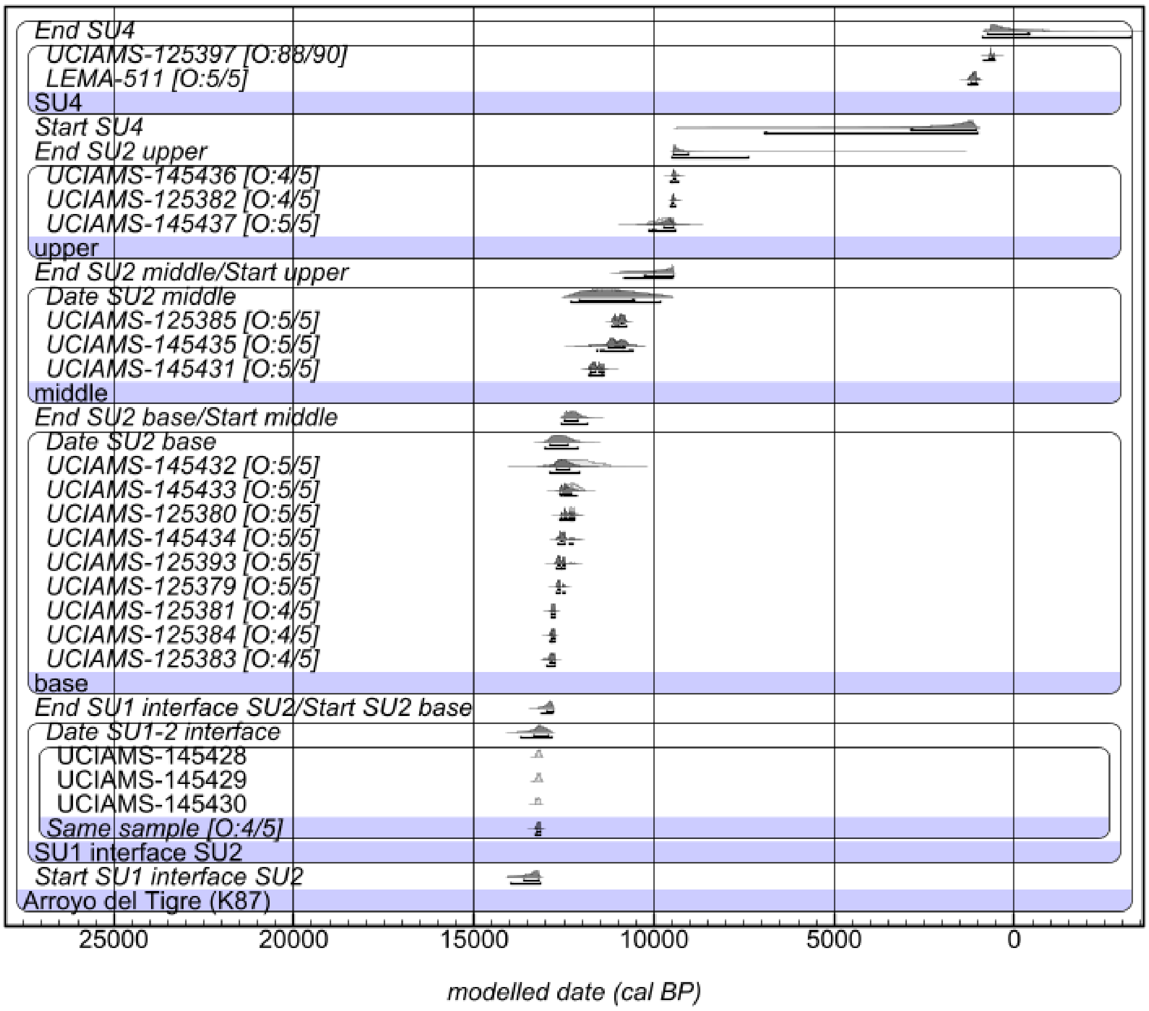


**Supplementary Figure 90.** Bayesian age model for K87. Brackets beneath each age estimate show 68.3% and 95.4% CI. Outlier analysis output is noted as 'O:posterior probability/prior probability'.

```
Plot()
{
 Outlier_Model("SSimple",N(0,2),0,"s");
 Outlier_Model("General",T(5),U(0,4),"t");
 Sequence("Arroyo del Tigre (K87)")
 {
  Boundary("Start SU1 interface SU2");
  Phase("SU1 interface SU2")
  {
   R_Combine("Same sample")
   {
    R_Date("UCIAMS-145430", 11355, 30)
    {
     Outlier("SSimple", 0.05);
    };
    R_Date("UCIAMS-145429", 11320, 30)
    {
     Outlier("SSimple", 0.05);
    };
    R_Date("UCIAMS-145428", 11315, 30)
    {
     Outlier("SSimple", 0.05);
    };
    Outlier("General", 0.05);
   };
   Date("Date SU1-2 interface");
   Interval("Duration SU1-2 interface");
  };
  Boundary("End SU1 interface SU2/Start SU2 base");
  Phase("base")
  {
   R_Date("UCIAMS-125383", 10955, 50)
   {
    Outlier("General", 0.05);
   };
   R_Date("UCIAMS-125384", 10930, 20)
   {
    Outlier("General", 0.05);
   };
   R_Date("UCIAMS-125381", 10905, 20)
   {
    Outlier("General", 0.05);
   };
   R_Date("UCIAMS-125379", 10595, 25)
   {
    Outlier("General", 0.05);
   };
   R_Date("UCIAMS-125393", 10580, 50)
   {
    Outlier("General", 0.05);
   };
   R_Date("UCIAMS-145434", 10510, 45)
   {
    Outlier("General", 0.05);
   };
   R_Date("UCIAMS-125380", 10425, 20)
   {
    Outlier("General", 0.05);
   };
   R_Date("UCIAMS-145433", 10410, 60)
   {
    Outlier("General", 0.05);
   };
   R_Date("UCIAMS-145432", 10400, 300)
   {
    Outlier("General", 0.05);
   };
   Date("Date SU2 base");
   Interval("Duration SU2 base");
  };
  Boundary("End SU2 base/Start middle");
  Phase("middle")
  {
   R_Date("UCIAMS-145431", 10075, 30)
   {
    Outlier("General", 0.05);
   };
   R_Date("UCIAMS-145435", 9710, 130)
   {
    Outlier("General", 0.05);
   };
   R_Date("UCIAMS-125385", 9615, 20)
   {
    Outlier("General", 0.05);
   };
   Date("Date SU2 middle");
   Interval("Duration SU2 middle");
  };
  Boundary("End SU2 middle/Start upper");
  Phase("upper")
  {
   R_Date("UCIAMS-145437", 8690, 150)
   {
    Outlier("General", 0.05);
   };
   R_Date("UCIAMS-125382", 8425, 15)
   {
    Outlier("General", 0.05);
   };
   R_Date("UCIAMS-145436", 8405, 25)
   {
    Outlier("General", 0.05);
   };
  };
  Boundary("End SU2 upper");
  Interval("Gap SU2 to 4");
  Boundary("Start SU4");
  Phase("SU4")
  {
   R_Date("LEMA-511", 1210, 40)
   {
    Outlier("General", 0.05);
   };
   R_Date("UCIAMS-125397", 685, 15)
   {
    Outlier("General", 0.9);
   };
  };
  Boundary("End SU4");
 };
 Difference("Difference SU1-2 and SU2 base","Start SU1 interface SU2","End SU1 interface SU2/Start SU2 base");
 Difference("Difference SU2 base and middle","End SU1 interface SU2/Start SU2 base","End SU2 base/Start middle");
};
```

## 2.7.2. Pay Paso

Pay Paso is a locality with multiple open-air archaeological sites/components located in Uruguay (-30.2685, -57.460639), containing lithic technology and extinct fauna (*Equus sp*. and *Glyptodon sp*. in Pay Paso 1)[20,24,256,258,259]. There are three cultural components, as defined by stratigraphic, cultural and chronometric evidence in Pay Paso 1 (see Fig. 2 in Suarez[24]). Component 1 is located at the base of the profile in stratigraphic unit U2a, at a depth of 5.7 m. The dating of this component is in line ages reported for Fishtail points found elsewhere. Component 2 is found in stratigraphic unit U2c and contains Tigre complex artefacts, whilst component 3 is in stratigraphic unit U2d and contains Pay Paso complex bifacial points.

Bayesian modelling of Pay Paso 1 estimates the start of components 1-3 at 12890-12750 cal BP (Fishtail), 11945-11740 cal BP (Tigre) and 11070-10710 cal BP (Pay Paso), respectively, with no overlap between the three (and each complex) at 95% CI (see Supplementary Figure 91 and OxCal code). There are no outliers.

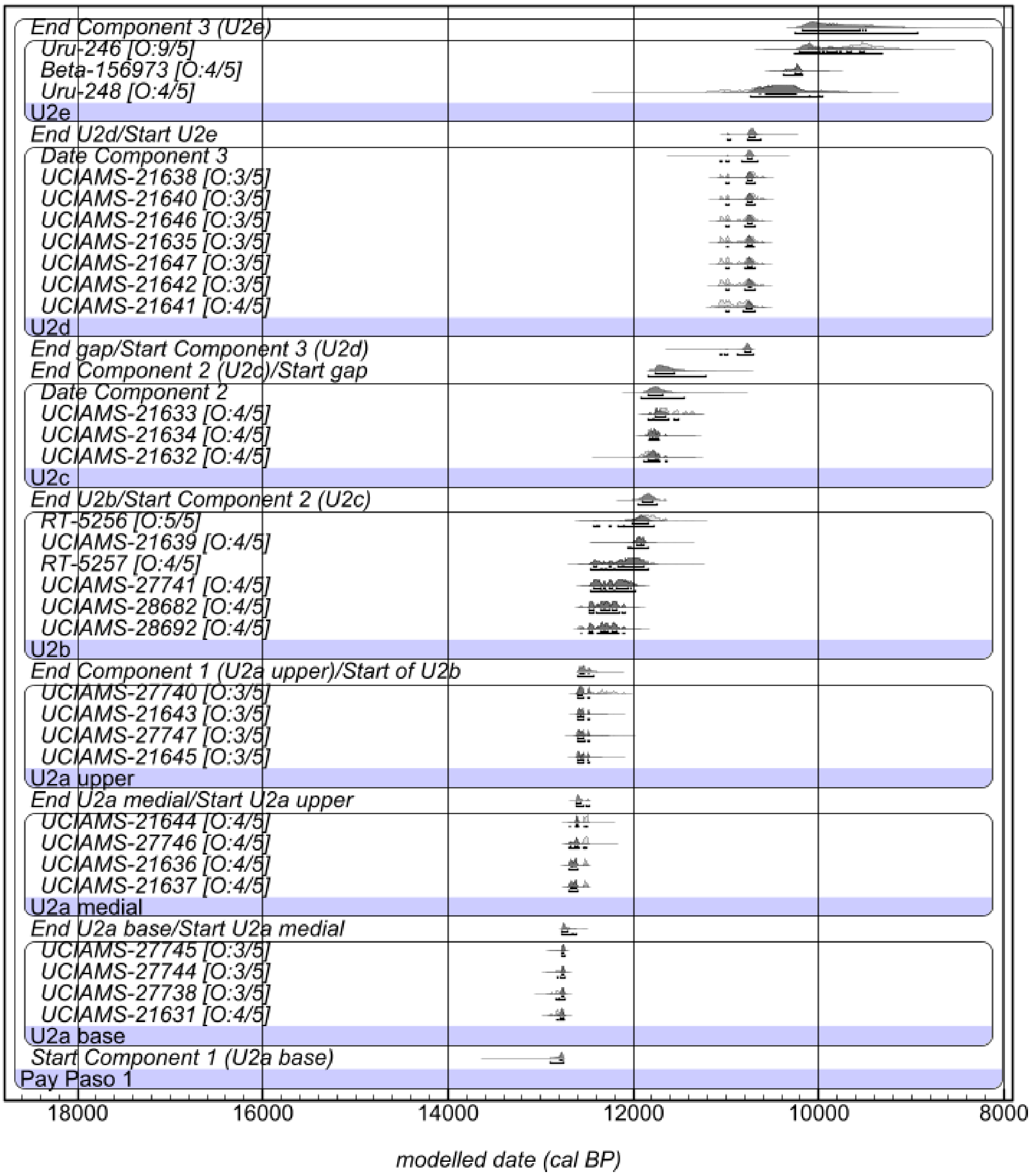


**Supplementary Figure 91.** Bayesian age model for Pay Paso 1. Brackets beneath each age estimate show 68.3% and 95.4% CI. Outlier analysis output is noted as 'O:posterior probability/prior probability'.

```
Plot()
{
 Outlier_Model("General",T(5),U(0,4),"t");
 Curve("SHCal20","shcal20.14c");
 Sequence("Pay Paso 1")
 {
  Boundary("Start Component 1 (U2a base)");
  Phase("U2a base")
  {
   R_Date("UCIAMS-21631", 10930, 20)
   {
    Outlier("General", 0.05);
   };
   R_Date("UCIAMS-27738", 10910, 30)
   {
    Outlier("General", 0.05);
   };
   R_Date("UCIAMS-27744", 10895, 30)
   {
    Outlier("General", 0.05);
   };
   R_Date("UCIAMS-27745", 10880, 25)
   {
    Outlier("General", 0.05);
   };
  };
  Boundary("End U2a base/Start U2a medial");
  Phase("U2a medial")
  {
   R_Date("UCIAMS-21637", 10630, 25)
   {
    Outlier("General", 0.05);
   };
   R_Date("UCIAMS-21636", 10630, 25)
   {
    Outlier("General", 0.05);
   };
   R_Date("UCIAMS-27746", 10595, 30)
   {
    Outlier("General", 0.05);
   };
   R_Date("UCIAMS-21644", 10580, 20)
   {
    Outlier("General", 0.05);
   };
  };
  Boundary("End U2a medial/Start U2a upper");
  Phase("U2a upper")
  {
   R_Date("UCIAMS-21645", 10550, 20)
   {
    Outlier("General", 0.05);
   };
   R_Date("UCIAMS-27747", 10540, 35)
   {
    Outlier("General", 0.05);
   };
   R_Date("UCIAMS-21643", 10520, 20)
   {
    Outlier("General", 0.05);
   };
   R_Date("UCIAMS-27740", 10500, 25)
   {
    Outlier("General", 0.05);
   };
  };
  Boundary("End Component 1 (U2a upper)/Start of U2b");
  Phase("U2b")
  {
   R_Date("UCIAMS-28692", 10465, 30)
   {
    Outlier("General", 0.05);
   };
   R_Date("UCIAMS-28682", 10450, 25)
   {
    Outlier("General", 0.05);
   };
   R_Date("UCIAMS-27741", 10390, 30)
   {
    Outlier("General", 0.05);
   };
   R_Date("RT-5257", 10320, 70)
   {
    Outlier("General", 0.05);
   };
   R_Date("UCIAMS-21639", 10285, 25)
   {
    Outlier("General", 0.05);
   };
   R_Date("RT-5256", 10225, 70)
   {
    Outlier("General", 0.05);
   };
  };
  Boundary("End U2b/Start Component 2 (U2c)");
  Phase("U2c")
  {
   R_Date("UCIAMS-21632", 10205, 35)
   {
    Outlier("General", 0.05);
   };
   R_Date("UCIAMS-21634", 10180, 20)
   {
    Outlier("General", 0.05);
   };
   R_Date("UCIAMS-21633", 10115, 25)
   {
    Outlier("General", 0.05);
   };
   Interval("Duration Component 2");
   Date("Date Component 2");
  };
  Boundary("End Component 2 (U2c)/Start gap");
  Boundary("End gap/Start Component 3 (U2d)");
  Phase("U2d")
  {
   R_Date("UCIAMS-21641", 9585, 25)
   {
    Outlier("General", 0.05);
   };
   R_Date("UCIAMS-21642", 9555, 25)
   {
    Outlier("General", 0.05);
   };
   R_Date("UCIAMS-21647", 9550, 20)
   {
    Outlier("General", 0.05);
   };
   R_Date("UCIAMS-21635", 9545, 20)
   {
    Outlier("General", 0.05);
   };
   R_Date("UCIAMS-21646", 9545, 20)
   {
    Outlier("General", 0.05);
   };
   R_Date("UCIAMS-21640", 9525, 20)
   {
    Outlier("General", 0.05);
   };
   R_Date("UCIAMS-21638", 9525, 20)
   {
    Outlier("General", 0.05);
   };
   Interval("Duration Component 3");
   Date("Date Component 3");
  };
  Boundary("End U2d/Start U2e");
  Phase("U2e")
  {
   R_Date("Uru-248", 9280, 200)
   {
    Outlier("General", 0.05);
   };
   R_Date("Beta-156973", 9120, 40)
   {
    Outlier("General", 0.05);
   };
   R_Date("Uru-246", 8570, 150)
   {
    Outlier("General", 0.05);
   };
  };
  Boundary("End Component 3 (U2e)");
 };
 Difference("Difference 1 and 2","Start Component 1 (U2a base)","End U2b/Start Component 2 (U2c)");
 Difference("Difference 2 and 3","End U2b/Start Component 2 (U2c)","End gap/Start Component 3 (U2d)");
 Difference("Difference 1 and 3","Start Component 1 (U2a base)","End gap/Start Component 3 (U2d)");
 Sequence()
 {
  Boundary("=Start Component 1 (U2a base)");
  Interval("Duration of Component 1");
  Date("Date of Component 1");
  Boundary("=End Component 1 (U2a upper)/Start of U2b");
 };
};
```

## *2.8. Venezuela*

### 2.8.1. El Vano

El Vano is an open-air site located in Venezuela (9.62, -70.00), containing El Jobo technology in association with *Eremotherium rusconni* and cf. *Glyptotherium* sp. *remains*[260–262]. There are two stratigraphic units (AAg and ARg) as defined by sedimentological and cultural evidence, with ARg showing the earliest evidence of human activity (El Jobo projectile points). Faunal bones were found in AAg[261]. There are three radiocarbon dates for El Vano: Beta-95601 (bone apatite; 7400 ± 50 BP), -95602 (bone protein, likely collagen; 10710 ± 60 BP), -95603 (sediment; 8360 ± 60 BP). Given that sediment and bone apatite dating is problematic[263–267], Beta-95602 (12760-12615 cal BP) is likely the most reliable measurement.

In 2022, the author attempted to radiocarbon date *Eremotherium* sp. and *Glyptotherium* sp. remains for the site. Unfortunately, no measurements were produced due to poor collagen preservation.

### 2.8.2. Taima-Taima

Taima-Taima is an open-air site (water hole) located in Venezuela (11.43, -69.75), containing extinct faunal remains (juvenile *Notiomastodon platensis*; some elements showing cut marks) in close association with El Jobo technology (the midsection of an el Jobo point was found within the cavity of the right pubis)[13,268–275]. There are four stratigraphic levels at the site (see Fig. 3 in Bryan et al.[13]). The mastodon skeleton was found within the water saturated grey clayey sand of unit I, with additional *N. platensis*, *Glyptodon* sp., *Equus* sp., *Glossotherium* sp., and *Arctodus* sp. bones located in the lower section. Radiocarbon dates for the site include twigs hypothesised to be *N. Platensis* stomach/intestine contents[13].

In 2022, the author attempted to radiocarbon date *Notiomastodon* sp., *Glyptotherium* sp. and *Eremotherium* sp., and unidentified faunal remains for the site. Unfortunately, no measurements were produced due to poor collagen preservation.

Bayesian modelling estimates the start of unit I at 18255-16750 cal BP (or 17670-16980 at 62.8% CI), with one outlier (bone carbonate age for sample IVIC-191-B, noted as no code 19; see Supplementary Figure 92 and OxCal code). The age range for unit I is estimated at 17650-13060 cal BP, overlapping the ACR-YD period. The bone carbonate age is likely an underestimate, which is in line with bone carbonate uptaking younger carbon[276] and thus being unreliable. An age produced on lignite (no laboratory code) was not included in the model, as a clear outlier (overestimate) at 36690±2050 BP.

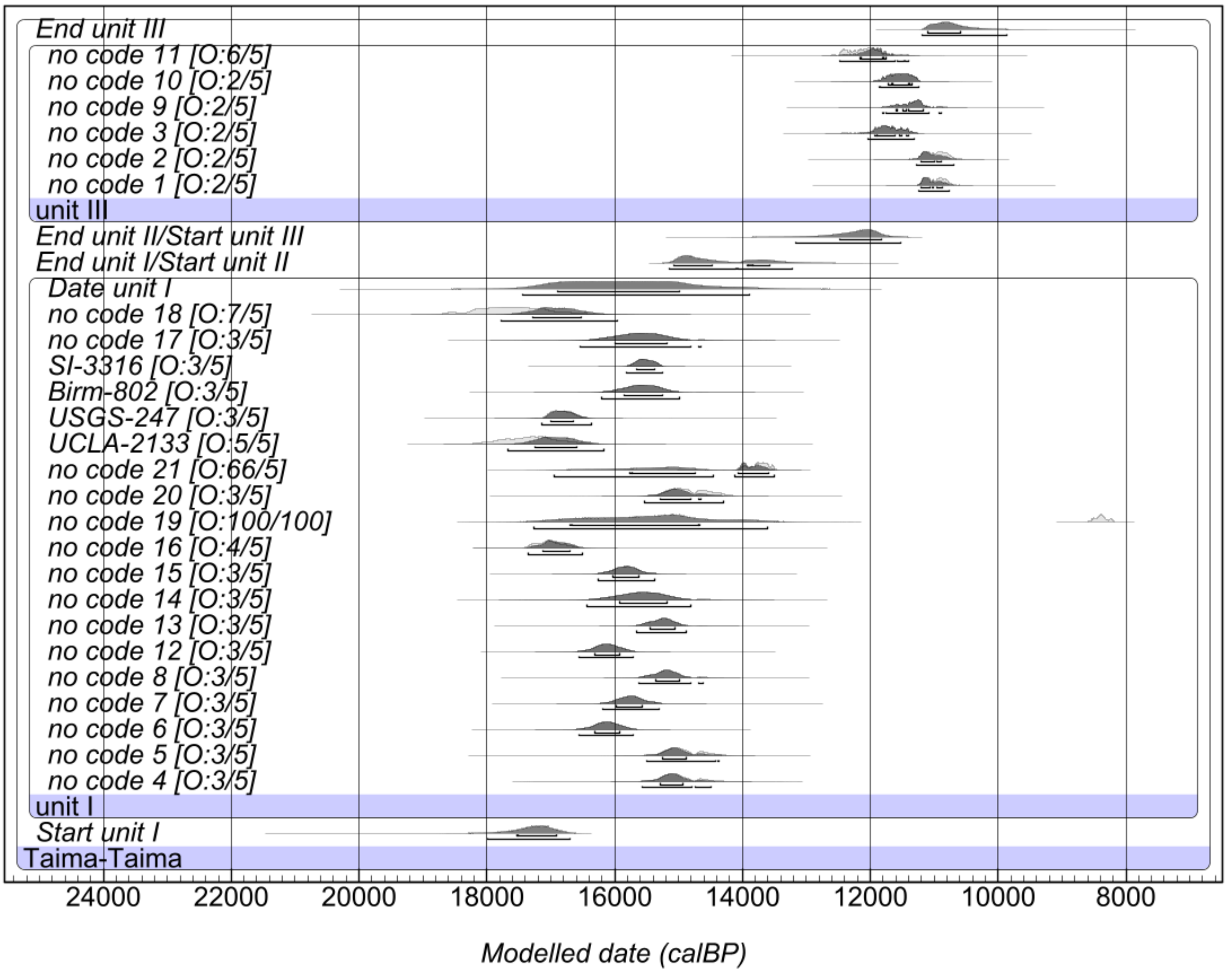


**Supplementary Figure 92.** Bayesian age model for Taima-Taima. Brackets beneath each age estimate show 68.3% and 95.4% CI. Outlier analysis output is noted as 'O:posterior probability/prior probability'.

```
Plot()
{
 Outlier_Model("General",T(5),U(0,4),"t");
 Sequence("Taima-Taima")
 {
  Boundary("Start unit I");
  Phase("unit I")
  {
   R_Date("no code 4",12660,120)
   {
    Outlier("General", 0.05);
    material="grey sand";
   };
   R_Date("no code 5",12620,120)
   {
    Outlier("General", 0.05);
    material="grey sand";
   };
   R_Date("no code 6",13390,130)
   {
    Outlier("General", 0.05);
    material="grey sand";
   };
   R_Date("no code 7",13130,130)
   {
    Outlier("General", 0.05);
    material="grey sand";
   };
   R_Date("no code 8",12730,120)
   {
    Outlier("General", 0.05);
    material="grey sand";
   };
   R_Date("no code 12",13390,130)
   {
    Outlier("General", 0.05);
    material="grey sand";
   };
   R_Date("no code 13",12770,120)
   {
    Outlier("General", 0.05);
    material="grey sand";
   };
   R_Date("no code 14",12990,260)
   {
    Outlier("General", 0.05);
    material="grey sand";
   };
   R_Date("no code 15",13180,130)
   {
    Outlier("General", 0.05);
    material="grey sand";
   };
   R_Date("no code 16",14010,140)
   {
    Outlier("General", 0.05);
    material="grey sand";
   };
   R_Date("no code 19",7590,100)
   {
    Outlier("General", 1);
    material="bone carbonate";
   };
   R_Date("no code 20",12580,150)
   {
    Outlier("General", 0.05);
    material="mixed grey sand";
   };
   R_Date("no code 21",11860,130)
   {
    Outlier("General", 0.05);
    material="wood";
   };
   R_Date("UCLA-2133",14200,300)
   {
    Outlier("General", 0.05);
    material="twig";
   };
   R_Date("USGS-247",13880,120)
   {
    Outlier("General", 0.05);
    material="twig";
   };
   R_Date("Birm-802",13000,200)
   {
    Outlier("General", 0.05);
    material="twig";
   };
   R_Date("SI-3316",12980,85)
   {
    Outlier("General", 0.05);
    material="twig";
   };
   R_Date("no code 17",13010,280)
   {
    Outlier("General", 0.05);
    material="bone non-carbonate carbon";
   };
   R_Date("no code 18",14440,435)
   {
    Outlier("General", 0.05);
    material="bone non-carbonate carbon";
   };
   Date("Date unit I");
   Interval("Duration unit I");
  };
  Boundary("End unit I/Start unit II");
  Boundary("End unit II/Start unit III");
  Phase("unit III")
  {
   R_Date("no code 1",9650,80)
   {
    Outlier("General", 0.05);
    material="black clay";
   };
   R_Date("no code 2",9650,110)
   {
    Outlier("General", 0.05);
    material="black clay";
   };
   R_Date("no code 3",10140,90)
   {
    Outlier("General", 0.05);
    material="black clay";
   };
   R_Date("no code 9",9860,110)
   {
    Outlier("General", 0.05);
    material="black clay";
   };
   R_Date("no code 10",10030,90)
   {
    Outlier("General", 0.05);
    material="black clay";
   };
   R_Date("no code 11",10290,90)
   {
    Outlier("General", 0.05);
    material="black clay";
   };
  };
  Boundary("End unit III");
 };
};
//excluded date
//R_Date("no code 22",36690,2050)
//material=lignite;
```

## 3. OxCal code (not already in previous sections; .prior files in Source data folder)

### *Figure 2*

```
Date(Prior("southern Andes","southernAndes_S.prior"))
{
 color="#0E4A7C";
};
Date(Prior("central Andes","centralAndes_S.prior"))
{
 color="#0E4A7C";
};
Date(Prior("poleward 40S s. Andes","poleward40_S.prior"))
{
 color="#0E4A7C";
};
Date(Prior("northern Andes","northernAndes_S.prior"))
{
 color="#0E4A7C";
};
Date(Prior("Paraguay-Parana Lowlands","Paraguaylow_S.prior"))
{
 color="#0E4A7C";
};
Date(Prior("Patagonian Plateau","Patagonian_S.prior"))
{
 color="#0E4A7C";
};
Date(Prior("Brazilian Highlands","Brazilianhigh_S.prior"))
{
 color="#0E4A7C";
};
Date(Prior("Orinoco Lowlands","orinoco_S.prior"))
{
 color="#0E4A7C";
};
Date(Prior("Amazonian Lowlands","amazonian_S.prior"))
{
 color="#0E4A7C";
};
Line( );
Date(Prior("El Jobo","El_Jobo_S.prior"))
{
 color="#0F8A98";
};
Date(Prior("uniface/biface","uniface_tech_S.prior"))
{
 color="#0F8A98";
};
Date(Prior("Abriense","Abriense_tech_S.prior"))
{
 color="#0F8A98";
};
Date(Prior("Paijan","Paijan_tech_S.prior"))
{
 color="#0F8A98";
};
Date(Prior("projectile point","projectile_tech_S.prior"))
{
 color="#0F8A98";
};
Date(Prior("Tequendamiense","Teq_tech_S.prior"))
{
 color="#0F8A98";
};
Date(Prior("Fishtail","Fishtail_tech_S.prior"))
{
 color="#0F8A98";
};
Date(Prior("Itaparica","Itaparica_tech_S.prior"))
{
 color="#0F8A98";
};
Date(Prior("Tigre","Tigre_tech_S.prior"))
{
 color="#0F8A98";
};
Date(Prior("Huentelauquen","Huente_tech_S.prior"))
{
 color="#0F8A98";
};
Date(Prior("Pay Paso","PP_S_3.prior"))
{
 color="#0F8A98";
};
Line( );
Date(Prior("high altitude","high_alt_S.prior"))
{
 color="#C9A700";
};
Line( );
Date(Prior("megafauna kill/scavenge","megafauna_S.prior"))
{
 color="#D62728";
};
```

### *Figure 3*

```
KDE_Model("summarised distribution")
{
 Date(Prior("Abrigo los Pinos (Date)","AlP_D.prior"))
 {
  site="Abrigo los Pinos";
  latitude=-37.4;
  longitude=-58.9;
  country="Argentina";
  type="rockshelter";
  high altitude="n";
  province="Paraguay-Parana Lowlands";
  technology="Fishtail";
  megafauna kill/scavenge="n";
 };
 Date(Prior("Agua de la Cueva (Date 2b)","AdC_2b_D.prior"))
 {
  site="Agua de la Cueva";
  latitude=-32.6169;
  longitude=-69.1636;
  country="Argentina";
  type="rockshelter";
  high altitude="y";
  province="southern Andes";
  technology="uniface/biface";
  megafauna kill/scavenge="n";
 };
 Date(Prior("Agua de la Cueva (Date 2c)","AdC_2c_D.prior"))
 {
  site="Agua de la Cueva";
  latitude=-32.6169;
  longitude=-69.1636;
  country="Argentina";
  type="rockshelter";
  high altitude="y";
  province="southern Andes";
  technology="uniface/biface";
  megafauna kill/scavenge="n";
 };
 Date(Prior("Alero 224A (Date)","A224A_D.prior"))
 {
  site="Alero 224A";
  latitude=-25.2;
  longitude=-70.4;
  country="Chile";
  type="rockshelter";
  high altitude="n";
  province="southern Andes";
  technology="Huentelauquen";
  megafauna kill/scavenge="n";
 };
 Date(Prior("Alero 225 (Date)","A225_D.prior"))
 {
  site="Alero 225";
  latitude=-25.2;
  longitude=-70.4;
  country="Chile";
  type="rockshelter";
  high altitude="n";
  province="southern Andes";
  technology="Huentelauquen";
  megafauna kill/scavenge="n";
 };
 Date(Prior("Alero Cascabeles 226/5 (Date)","AC_226_D.prior"))
 {
  site="Alero Cascabeles 226/5";
  latitude=-25.2;
  longitude=-70.4;
  country="Chile";
  type="rockshelter";
  high altitude="n";
  province="southern Andes";
  technology="Huentelauquen";
  megafauna kill/scavenge="n";
 };
 Date(Prior("Arroyo de Frías MLP 5582 (Date)","AdF_D.prior"))
 {
  site="Arroyo de Frías MLP 5582";
  latitude=-34.65;
  longitude=-59.4307;
  country="Argentina";
  type="open-air";
  high altitude="n";
  province="Paraguay-Parana Lowlands";
  technology="uniface/biface";
  megafauna kill/scavenge="n";
 };
 Date(Prior("Arroyo del Tigre K87 (Date SU1-SU2 interface)","K87_D_1_2.prior"))
 {
  site="Arroyo del Tigre K87";
  latitude=-30.4;
  longitude=-57.8;
  country="Uruguay";
  type="open-air";
  high altitude="n";
  province="Paraguay-Parana Lowlands";
  technology="uniface/biface";
  megafauna kill/scavenge="n";
 };
 Date(Prior("Arroyo del Tigre K87 (Date SU2 base)","K87_D_base.prior"))
 {
  site="Arroyo del Tigre K87";
  latitude=-30.4;
  longitude=-57.8;
  country="Uruguay";
  type="open-air";
  high altitude="n";
  province="Paraguay-Parana Lowlands";
  technology="Fishtail";
  megafauna kill/scavenge="n";
 };
 Date(Prior("Arroyo del Tigre K87 (Date SU2 middle)","K87_D_mid.prior"))
 {
  site="Arroyo del Tigre K87";
  latitude=-30.4;
  longitude=-57.8;
  country="Uruguay";
  type="open-air";
  high altitude="n";
  province="Paraguay-Parana Lowlands";
  technology="Tigre";
  megafauna kill/scavenge="n";
 };
 Date(Prior("Arroyo Seco 2 (Date unit S)","AS2_D_unitS.prior"))
 {
  site="Arroyo Seco 2";
  latitude=-38.360556;
  longitude=-60.244167;
  country="Argentina";
  type="open-air";
  high altitude="n";
  province="Paraguay-Parana Lowlands";
  technology="uniface/biface";
  megafauna kill/scavenge="y";
```

```
};
Date(Prior("Arroyo Seco 2 (Date unit Y)","AS2_D_unitY.prior"))
{
 site="Arroyo Seco 2";
 latitude=-38.360556;
 longitude=-60.244167;
 country="Argentina";
 type="open-air";
 high altitude="n";
 province="Paraguay-Parana Lowlands";
 technology="uniface/biface";
 megafauna kill/scavenge="y";
};
Date(Prior("Boqueirão do Sitio da Pedra Furada (Date ST1)","PF_D_ST1.prior"))
{
 site="Boqueirão do Sitio da Pedra Furada";
 latitude=-8.85;
 longitude=-42.55;
 country="Brazil";
 type="rockshelter";
 high altitude="n";
 province="Brazilian Highlands";
 technology="Itaparica";
 megafauna kill/scavenge="n";
};
Date(Prior("Toca da Janela da Barra do Antonião (Date C4)","TDJ_D_C4.prior"))
{
 site="Toca da Janela da Barra do Antonião";
 latitude=-8.802222;
 longitude=-42.433056;
 country="Brazil";
 type="rockshelter";
 high altitude="n";
 province="Brazilian Highlands";
 technology="uniface/biface";
 megafauna kill/scavenge="n";
};
Date(Prior("Toca da Janela da Barra do Antonião (Date C3)","TDJ_D_C3.prior"))
{
 site="Toca da Janela da Barra do Antonião";
 latitude=-8.802222;
 longitude=-42.433056;
 country="Brazil";
 type="rockshelter";
 high altitude="n";
 province="Brazilian Highlands";
 technology="uniface/biface";
 megafauna kill/scavenge="n";
};
Date(Prior("Campo Laborde (Date S1 all)","CL_D_1_all.prior"))
{
 site="Campo Laborde";
 latitude=-37.1;
 longitude=-60.38;
 country="Argentina";
 type="open-air";
 high altitude="n";
 province="Paraguay-Parana Lowlands";
 technology="uniface/biface";
 megafauna kill/scavenge="y";
};
Date(Prior("Campo Laborde (Date S1 XAD only)","CL_D_1_XAD.prior"))
{
 site="Campo Laborde";
 latitude=-37.1;
 longitude=-60.38;
 country="Argentina";
 type="open-air";
 high altitude="n";
 province="Paraguay-Parana Lowlands";
 technology="uniface/biface";
 megafauna kill/scavenge="y";
};
Date(Prior("Casa del Minero 1 (Date SU3c inferior)","CM1_D_SU3c.prior"))
{
 site="Casa del Minero 1";
 latitude=-48.575833;
 longitude=-68.9125;
 country="Argentina";
 type="cave";
 high altitude="n";
 province="Patagonian Plateau";
 technology="uniface/biface";
 megafauna kill/scavenge="y";
};
Date(Prior("Casa del Minero 1 (Date SU4)","CM1_D_SU4.prior"))
{
 site="Casa del Minero 1";
 latitude=-48.575833;
 longitude=-68.9125;
 country="Argentina";
 type="cave";
 high altitude="n";
 province="Patagonian Plateau";
 technology="uniface/biface";
 megafauna kill/scavenge="y";
};
Date(Prior("Caverna da Pedra Pintada (Date early period)","CPP_D_early.prior"))
{
 site="Caverna da Pedra Pintada";
 latitude=-1.1;
 longitude=-54.06;
 country="Brazil";
 type="cave";
 high altitude="n";
 province="Amazonian Lowlands";
 technology="uniface/biface";
 megafauna kill/scavenge="n";
};
Date(Prior("Caverna da Pedra Pintada (Date inital period)","CPP_D_initial.prior"))
{
 site="Caverna da Pedra Pintada";
 latitude=-1.1;
 longitude=-54.06;
 country="Brazil";
 type="cave";
 high altitude="n";
 province="Amazonian Lowlands";
 technology="uniface/biface";
 megafauna kill/scavenge="n";
};
Date(Prior("Caverna da Pedra Pintada (Date late period)","CPP_D_late.prior"))
{
 site="Caverna da Pedra Pintada";
 latitude=-1.1;
 longitude=-54.06;
 country="Brazil";
 type="cave";
 high altitude="n";
 province="Amazonian Lowlands";
 technology="uniface/biface";
 megafauna kill/scavenge="n";
};
Date(Prior("Cerro el Sombrero Abrigo 1 (Date)","CSA1_D.prior"))
{
 site="Cerro el Sombrero Abrigo 1";
 latitude=-37.8166;
 longitude=-58.5666;
 country="Argentina";
 type="rockshelter";
 high altitude="n";
 province="Paraguay-Parana Lowlands";
 technology="Fishtail";
 megafauna kill/scavenge="n";
};
Date(Prior("Cerro La China I-III (Date)","CLC_D.prior"))
{
 site="Cerro La China I-III";
 latitude=-37.95;
 longitude=-58.6166;
 country="Argentina";
 type="rockshelter";
 high altitude="n";
 province="Paraguay-Parana Lowlands";
 technology="Fishtail";
 megafauna kill/scavenge="n";
};
Date(Prior("Cerro Tres Tetas (Date layer 5 lower)","C3T_D_layer5lower.prior"))
{
 site="Cerro Tres Tetas";
 latitude=-48.149444;
 longitude=-68.933333;
 country="Argentina";
 type="open-air";
 high altitude="n";
 province="Patagonian Plateau";
 technology="uniface/biface";
 megafauna kill/scavenge="n";
};
Date(Prior("Chipana 1 (Date)","Chipana1_D.prior"))
{
 site="Chipana 1";
 latitude=-21.2;
 longitude=-69.4;
 country="Chile";
 type="open-air";
 high altitude="n";
 province="southern Andes";
 technology="uniface/biface";
 megafauna kill/scavenge="n";
};
Date(Prior("Cueva Bautista (Date level O)","CB_D_O.prior"))
{
 site="Cueva Bautista";
 latitude=-21.338;
 longitude=-67.574;
 country="Bolivia";
 type="rockshelter";
 high altitude="y";
 province="central Andes";
 technology="uniface/biface";
 megafauna kill/scavenge="n";
};
Date(Prior("Cueva Bautista (Date level P)","CB_D_P.prior"))
{
 site="Cueva Bautista";
 latitude=-21.338;
 longitude=-67.574;
 country="Bolivia";
 type="rockshelter";
 high altitude="y";
 province="central Andes";
 technology="uniface/biface";
 megafauna kill/scavenge="n";
};
Date(Prior("Cueva Burucuyá (Date)","CBuru_D.prior"))
{
 site="Cueva Burucuyá";
 latitude=-37.8;
 longitude=-58.1;
 country="Argentina";
 type="cave";
 high altitude="n";
 province="Paraguay-Parana Lowlands";
 technology="uniface/biface";
 megafauna kill/scavenge="n";
};
Date(Prior("Cueva de Fell (Date Period I)","CF_D_Fell.prior"))
{
 site="Cueva de Fell";
 latitude=-52.044444;
 longitude=-70.056389;
 country="Chile";
 type="rockshelter";
 high altitude="n";
 province="Patagonian Plateau";
 technology="Fishtail";
 megafauna kill/scavenge="y";
};
Date(Prior("Cueva de la Vieja (Date)","CdlV_D.prior"))
{
 site="Cueva de la Vieja";
 latitude=-42.274167;
 longitude=-71.54;
 country="Chile";
 type="cave";
 high altitude="n";
 province="southern Andes";
 technology="uniface/biface";
 megafauna kill/scavenge="y";
};
Date(Prior("Cueva del Medio (Date Fell I)","CM_D_Fell_I.prior"))
{
 site="Cueva del Medio";
 latitude=-51.576;
 longitude=-72.583;
 country="Chile";
 type="cave";
 high altitude="n";
 province="Patagonian Plateau";
 technology="Fishtail";
 megafauna kill/scavenge="y";
};
```

```
Date(Prior("Cueva del Medio (Date Fell III)","CM_D_Fell_III.prior"))
{
 site="Cueva del Medio";
 latitude=-51.576;
 longitude=-72.583;
 country="Chile";
 type="cave";
 high.altitude="n";
 province="Patagonian Plateau";
 technology="Fishtail";
 megafauna kill/scavenge="y";
};
Date(Prior("Cueva Tixi (Date)","CTixi_D.prior"))
{
 site="Cueva Tixi";
 latitude=-37.4;
 longitude=-58.9;
 country="Argentina";
 type="cave";
 high.altitude="n";
 province="Paraguay-Parana Lowlands";
 technology="projectile point";
 megafauna kill/scavenge="n";
};
Date(Prior("Cueva Túnel (Date)","CTunel_D.prior"))
{
 site="Cueva Túnel";
 latitude=-48.4575;
 longitude=-68.8725;
 country="Argentina";
 type="cave";
 high.altitude="n";
 province="Patagonian Plateau";
 technology="uniface/biface";
 megafauna kill/scavenge="n";
};
Date(Prior("Cueva Zoro (Date)","CZ_D.prior"))
{
 site="Cueva Zoro";
 latitude=-37.8166;
 longitude=-58.6333;
 country="Argentina";
 type="rockshelter";
 high.altitude="n";
 province="Paraguay-Parana Lowlands";
 technology="uniface/biface";
 megafauna kill/scavenge="n";
};
Date(Prior("Cuncaicha (Date TP component)","Cun_D_TP.prior"))
{
 site="Cuncaicha";
 latitude=-15.373;
 longitude=-72.618;
 country="Peru";
 type="rockshelter";
 high.altitude="y";
 province="central Andes";
 technology="projectile point";
 megafauna kill/scavenge="n";
};
Date(Prior("El Abra (Date unit C)","EA_D_C.prior"))
{
 site="El Abra";
 latitude=5.02;
 longitude=-73.937;
 country="Colombia";
 type="rockshelter";
 high.altitude="n";
 province="northern Andes";
 technology="Abriense";
 megafauna kill/scavenge="n";
};
Date(Prior("El Alto 3 (Date SU3)","EA3_SU3_D.prior"))
{
 site="El Alto 3";
 latitude=-31.3988;
 longitude=-64.7388;
 country="Argentina";
 type="rockshelter";
 high.altitude="n";
 province="southern Andes";
 technology="projectile point";
 megafauna kill/scavenge="n";
};
Date(Prior("El Alto 3 (Date SU4)","EA3_SU4_D.prior"))
{
 site="El Alto 3";
 latitude=-31.3988;
 longitude=-64.7388;
 country="Argentina";
 type="rockshelter";
 high.altitude="n";
 province="southern Andes";
 technology="projectile point";
 megafauna kill/scavenge="n";
};
Date(Prior("El Membrillo LV.105 (Date)","Membrillo_D.prior"))
{
 site="El Membrillo LV.105";
 latitude=-31.928333;
 longitude=-71.488333;
 country="Chile";
 type="open-air";
 high.altitude="n";
 province="southern Andes";
 technology="uniface/biface";
 megafauna kill/scavenge="y";
};
Date(Prior("El Obispo 1 (Date)","EO1_D.prior"))
{
 site="El Obispo 1";
 latitude=-26.5;
 longitude=-70.6;
 country="Chile";
 type="open-air";
 high.altitude="n";
 province="southern Andes";
 technology="Huentelauquen";
 megafauna kill/scavenge="n";
};
Date(Prior("GO-JA-01 (Date)","GOJA01_D.prior"))
{
 site="GO-JA-01";
 latitude=-18.28638889;
 longitude=-52.04777778;
 country="Brazil";
 type="rockshelter";
 high.altitude="n";
 province="Brazilian Highlands";
 technology="Itaparica";
 megafauna kill/scavenge="n";
};
Date(Prior("Gruta del Indio (Date Atuel IV)","GdI_IV_D.prior"))
{
 site="Gruta del Indio";
 latitude=-34.75;
 longitude=-68.3666;
 country="Argentina";
 type="cave";
 high.altitude="n";
 province="southern Andes";
 technology="uniface/biface";
 megafauna kill/scavenge="n";
};
Date(Prior("Guitarrero Cave (Date Complex I-II)","GC_D_I_II.prior"))
{
 site="Guitarrero Cave";
 latitude=-9.20069;
 longitude=-77.7098;
 country="Peru";
 type="cave";
 high.altitude="y";
 province="central Andes";
 technology="uniface/biface";
 megafauna kill/scavenge="n";
};
Date(Prior("Huaca Prieta (Date early Holocene/late Pleistocene)","HC_D_Pleis.prior"))
{
 site="Huaca Prieta";
 latitude=-7.9241;
 longitude=-79.3069;
 country="Peru";
 type="open-air";
 high.altitude="n";
 province="central Andes";
 technology="uniface/biface";
 megafauna kill/scavenge="n";
};
Date(Prior("Je-996 (Date)","Je_D.prior"))
{
 site="Je-996";
 latitude=-7.059;
 longitude=-79.397;
 country="Peru";
 type="open-air";
 high.altitude="n";
 province="central Andes";
 technology="Paiján and Fishtail";
 megafauna kill/scavenge="n";
};
Date(Prior("La Chimba 13 (Date)","LC13_D.prior"))
{
 site="La Chimba 13";
 latitude=-23.545556;
 longitude=-70.368889;
 country="Chile";
 type="open-air";
 high.altitude="n";
 province="southern Andes";
 technology="Huentelauquen";
 megafauna kill/scavenge="n";
};
Date(Prior("La Cueva 1 del Lago Sofía (Date Fell I)","CLS_D_Fell_I.prior"))
{
 site="La Cueva 1 del Lago Sofía";
 latitude=-51.53;
 longitude=-72.53;
 country="Chile";
 type="cave";
 high.altitude="n";
 province="Patagonian Plateau";
 technology="uniface/biface";
 megafauna kill/scavenge="y";
};
Date(Prior("La Gruta, Laguna 2, C1 (Date)","LG_D.prior"))
{
 site="La Gruta, Laguna 2, C1";
 latitude=-48.8261;
 longitude=-69.3961;
 country="Argentina";
 type="cave";
 high.altitude="n";
 province="Patagonian Plateau";
 technology="uniface/biface";
 megafauna kill/scavenge="n";
};
Date(Prior("La Palestina 2 05YON002-02 (Date horizon Bt)","LP2_D_Bt.prior"))
{
 site="La Palestina 2 05YON002-02";
 latitude=6.716;
 longitude=-74.383;
 country="Colombia";
 type="open-air";
 high.altitude="n";
 province="northern Andes";
 technology="uniface/biface";
 megafauna kill/scavenge="n";
};
Date(Prior("Lapa do Boquete (Date stratum 6)","LBo_D_6.prior"))
{
 site="Lapa do Boquete";
 latitude=-14.98;
 longitude=-44.44;
 country="Brazil";
 type="cave";
 high.altitude="n";
 province="Brazilian Highlands";
 technology="Itaparica";
 megafauna kill/scavenge="n";
};
Date(Prior("Lapa do Boquete (Date stratum 7)","LBo_D_7.prior"))
{
 site="Lapa do Boquete";
 latitude=-14.98;
 longitude=-44.44;
 country="Brazil";
 type="cave";
 high.altitude="n";
 province="Brazilian Highlands";
 technology="Itaparica";
 megafauna kill/scavenge="n";
};
Date(Prior("Lapa do Boquete (Date stratum 8)","LBo_D_8.prior"))
```

```
{
 site="Lapa do Boquete";
 latitude=-14.98;
 longitude=-44.44;
 country="Brazil";
 type="cave";
 high altitude="n";
 province="Brazilian Highlands";
 technology="Itaparica";
 megafauna kill/scavenge="n";
};
Date(Prior("Lapa do Caboblo (Date)","LdCabo_D.prior"))
{
 site="Lapa do Caboblo";
 latitude=-18.28527778;
 longitude=-43.85388889;
 country="Brazil";
 type="rockshelter/cave";
 high altitude="n";
 province="Brazilian Highlands";
 technology="uniface/biface";
 megafauna kill/scavenge="n";
};
Date(Prior("Lapa dos Bichos (Date stratum 7)","LBi_D_7.prior"))
{
 site="Lapa dos Bichos";
 latitude=-14.98;
 longitude=-44.44;
 country="Brazil";
 type="cave";
 high altitude="n";
 province="Brazilian Highlands";
 technology="Itaparica";
 megafauna kill/scavenge="n";
};
Date(Prior("Lapa dos Bichos (Date stratum 8)","LBi_D_8.prior"))
{
 site="Lapa dos Bichos";
 latitude=-14.98;
 longitude=-44.44;
 country="Brazil";
 type="cave";
 high altitude="n";
 province="Brazilian Highlands";
 technology="Itaparica";
 megafauna kill/scavenge="n";
};
Date(Prior("Los Médanos 2 (Date)","LM2_D.prior"))
{
 site="Los Médanos 2";
 latitude=-26.3;
 longitude=-70.6;
 country="Chile";
 type="open-air";
 high altitude="n";
 province="southern Andes";
 technology="Huentelauquen";
 megafauna kill/scavenge="n";
};
Date(Prior("Los Rieles (Date paleo-dune)","LR_D_dune.prior"))
{
 site="Los Rieles";
 latitude=-31.93;
 longitude=-71.52;
 country="Chile";
 type="open-air";
 high altitude="n";
 province="southern Andes";
 technology="uniface/biface";
 megafauna kill/scavenge="n";
};
Date(Prior("Monte Verde II (Date MV-6)","MVII_D_MV6.prior"))
{
 site="Monte Verde II";
 latitude=-41.51;
 longitude=-73.2;
 country="Chile";
 type="open-air";
 high altitude="n";
 province="southern Andes";
 technology="uniface/biface";
 megafauna kill/scavenge="y";
};
Date(Prior("Monte Verde II (Date MV-7)","MVII_D_MV7.prior"))
{
 site="Monte Verde II";
 latitude=-41.51;
 longitude=-73.2;
 country="Chile";
 type="open-air";
 high altitude="n";
 province="southern Andes";
 technology="uniface/biface";
 megafauna kill/scavenge="y";
};
Date(Prior("MS.PA.02 Casa da Pedra (Date)","MSPA_D.prior"))
{
 site="MS.PA.02 Casa da Pedra";
 latitude=-19.58333333;
 longitude=-52.66666667;
 country="Brazil";
 type="cave";
 high altitude="n";
 province="Brazilian Highlands";
 technology="uniface/biface";
 megafauna kill/scavenge="n";
};
Date(Prior("MT-SL-31-Morro da Janela (Date)","MTLS21_D.prior"))
{
 site="MT-SL-31-Morro da Janela";
 latitude=-15.92166667;
 longitude=-54.37833333;
 country="Brazil";
 type="rockshelter";
 high altitude="n";
 province="Brazilian Highlands";
 technology="uniface/biface";
 megafauna kill/scavenge="n";
};
Date(Prior("Nare 005PNA005 (Date horizon AB)","Nare_D.prior"))
{
 site="Nare 005PNA005";
 latitude=6.35;
 longitude=-74.1;
 country="Colombia";
 type="open-air";
 high altitude="n";
 province="northern Andes";
 technology="uniface/biface";
 megafauna kill/scavenge="n";
};
Date(Prior("Pampa de los Fósiles and Ascope (Date Paiján)","Paijan_D.prior"))
{
 site="Pampa de los Fósiles and Ascope";
 latitude=-7.71;
 longitude=-79.1;
 country="Peru";
 type="open-air";
 high altitude="n";
 province="central Andes";
 technology="Paijan";
 megafauna kill/scavenge="n";
};
Date(Prior("Pampa Ramaditas 5 (Date)","PR5_D.prior"))
{
 site="Pampa Ramaditas 5";
 latitude=-21.2;
 longitude=-69.4;
 country="Chile";
 type="open-air";
 high altitude="n";
 province="southern Andes";
 technology="projectile point";
 megafauna kill/scavenge="n";
};
Date(Prior("Pampa Ramaditas 7 (Date)","PR7_D.prior"))
{
 site="Pampa Ramaditas 7";
 latitude=-21.2;
 longitude=-69.4;
 country="Chile";
 type="open-air";
 high altitude="n";
 province="southern Andes";
 technology="uniface/biface";
 megafauna kill/scavenge="n";
};
Date(Prior("Paso Otero 5 (Date)","PO5_D.prior"))
{
 site="Paso Otero 5";
 latitude=-38.2022;
 longitude=-59.1327;
 country="Argentina";
 type="open-air";
 high altitude="n";
 province="Paraguay-Parana Lowlands";
 technology="Fishtail";
 megafauna kill/scavenge="y";
};
Date(Prior("Pay Paso 1 (Date component 1)","PP_D_1.prior"))
{
 site="Pay Paso 1";
 latitude=-30.2685;
 longitude=-57.460639;
 country="Uruguay";
 type="open-air";
 high altitude="n";
 province="Paraguay-Parana Lowlands";
 technology="Fishtail";
 megafauna kill/scavenge="y";
};
Date(Prior("Pay Paso 1 (Date component 2)","PP_D_2.prior"))
{
 site="Pay Paso 1";
 latitude=-30.2685;
 longitude=-57.460639;
 country="Uruguay";
 type="open-air";
 high altitude="n";
 province="Paraguay-Parana Lowlands";
 technology="Tigre";
 megafauna kill/scavenge="y";
};
Date(Prior("Pay Paso 1 (Date component 3)","PP_D_3.prior"))
{
 site="Pay Paso 1";
 latitude=-30.2685;
 longitude=-57.460639;
 country="Uruguay";
 type="open-air";
 high altitude="n";
 province="Paraguay-Parana Lowlands";
 technology="Pay Paso";
 megafauna kill/scavenge="y";
};
Date(Prior("Peñas de las Trampas 1.1 (Date)","PdlT1_D.prior"))
{
 site="Peñas de las Trampas 1.1";
 latitude=-26.0175;
 longitude=-67.35;
 country="Argentina";
 type="rockshelter";
 high altitude="y";
 province="southern Andes";
 technology="uniface/biface";
 megafauna kill/scavenge="n";
};
Date(Prior("Piedra Museo AEP-1 (Date SU4)","PM_D_SU4.prior"))
{
 site="Piedra Museo AEP-1";
 latitude=-47.895;
 longitude=-67.867778;
 country="Argentina";
 type="rockshelter";
 high altitude="n";
 province="Patagonian Plateau";
 technology="uniface/biface";
 megafauna kill/scavenge="y";
};
Date(Prior("Piedra Museo AEP-1 (Date SU5)","PM_D_SU5.prior"))
{
 site="Piedra Museo AEP-1";
 latitude=-47.895;
 longitude=-67.867778;
 country="Argentina";
 type="rockshelter";
 high altitude="n";
 province="Patagonian Plateau";
 technology="Fishtail";
 megafauna kill/scavenge="y";
};
Date(Prior("Piedra Museo AEP-1 (Date SU6)","PM_D_SU6.prior"))
{
```

```
site="Piedra Museo AEP-1";
latitude=-47.895;
longitude=-67.867778;
country="Argentina";
type="rockshelter";
high.altitude="n";
province="Patagonian Plateau";
technology="uniface/biface";
megafauna kill/scavenge="y";
};
Date(Prior("Pilauco (Date PB-7)","Pil_PB7_D.prior"))
{
site="Pilauco";
latitude=-40.566667;
longitude=-73.116667;
country="Chile";
type="open-air";
high.altitude="n";
province="southern Andes";
technology="uniface/biface";
megafauna kill/scavenge="n";
};
Date(Prior("Pilauco (Date PB-8)","Pil_PB8_D.prior"))
{
site="Pilauco";
latitude=-40.566667;
longitude=-73.116667;
country="Chile";
type="open-air";
high.altitude="n";
province="southern Andes";
technology="uniface/biface";
megafauna kill/scavenge="n";
};
Date(Prior("Pintoscayoc 1 (Date)","Pinto1_D.prior"))
{
site="Pintoscayoc 1";
latitude=-22.9444;
longitude=-65.4182;
country="Argentina";
type="rockshelter";
high.altitude="y";
province="southern Andes";
technology="projectile point";
megafauna kill/scavenge="n";
};
Date(Prior("Piuquenes (Date)","Piuquenes_D.prior"))
{
site="Piuquenes";
latitude=-32.266667;
longitude=-70.266667;
country="Chile";
type="rockshelter";
high.altitude="n";
province="southern Andes";
technology="projectile point";
megafauna kill/scavenge="n";
};
Date(Prior("Punta Ñagué (Date lower component)","PN_D_lower.prior"))
{
site="Punta Ñagué";
latitude=-31.85;
longitude=-71.52;
country="Chile";
type="open-air";
high.altitude="n";
province="southern Andes";
technology="Huentelauquen";
megafauna kill/scavenge="n";
};
Date(Prior("Punta Ñagué (Date middle component)","PN_D_middle.prior"))
{
site="Punta Ñagué";
latitude=-31.85;
longitude=-71.52;
country="Chile";
type="open-air";
high.altitude="n";
province="southern Andes";
technology="uniface/biface";
megafauna kill/scavenge="n";
};
Date(Prior("Punta Purgatorio (Date)","PPurga_D.prior"))
{
site="Punta Purgatorio";
latitude=-31.963333;
longitude=-71.499444;
country="Chile";
type="open-air";
high.altitude="n";
province="southern Andes";
technology="uniface/biface";
megafauna kill/scavenge="n";
};
Date(Prior("Quebrada Jaguay QJ-280 (Date level 2)","QJ_D_2.prior"))
{
site="Quebrada Jaguay QJ-280";
latitude=-16.527;
longitude=-72.856;
country="Peru";
type="open-air";
high.altitude="n";
province="central Andes";
technology="uniface/biface";
megafauna kill/scavenge="n";
};
Date(Prior("Quebrada Jaguay QJ-280 (Date level 3)","QJ_D_3.prior"))
{
site="Quebrada Jaguay QJ-280";
latitude=-16.527;
longitude=-72.856;
country="Peru";
type="open-air";
high.altitude="n";
province="central Andes";
technology="uniface/biface";
megafauna kill/scavenge="n";
};
Date(Prior("Quebrada Jaguay QJ-280 (Date level 4c)","QJ_D_4.prior"))
{
site="Quebrada Jaguay QJ-280";
latitude=-16.527;
longitude=-72.856;
country="Peru";
type="open-air";
high.altitude="n";
province="central Andes";
technology="uniface/biface";
megafauna kill/scavenge="n";
};
Date(Prior("Quebrada Maní 12 (Date stratum 1)","QM12_D_1.prior"))
{
site="Quebrada Maní 12";
latitude=-21.2;
longitude=-69.4;
country="Chile";
type="open-air";
high.altitude="n";
province="southern Andes";
technology="uniface/biface";
megafauna kill/scavenge="n";
};
Date(Prior("Quebrada Maní 12 (Date stratum 2)","QM12_D_2.prior"))
{
site="Quebrada Maní 12";
latitude=-21.2;
longitude=-69.4;
country="Chile";
type="open-air";
high.altitude="n";
province="southern Andes";
technology="uniface/biface";
megafauna kill/scavenge="n";
};
Date(Prior("Quebrada Maní 12 (Date stratum 3)","QM12_D_3.prior"))
{
site="Quebrada Maní 12";
latitude=-21.2;
longitude=-69.4;
country="Chile";
type="open-air";
high.altitude="n";
province="southern Andes";
technology="uniface/biface";
megafauna kill/scavenge="n";
};
Date(Prior("Quebrada Maní 12 (Date stratum 4)","QM12_D_4.prior"))
{
site="Quebrada Maní 12";
latitude=-21.2;
longitude=-69.4;
country="Chile";
type="open-air";
high.altitude="n";
province="southern Andes";
technology="uniface/biface";
megafauna kill/scavenge="n";
};
Date(Prior("Quebrada Maní 32 (Date occupation)","QM32_D.prior"))
{
site="Quebrada Maní 32";
latitude=-21.2;
longitude=-69.4;
country="Chile";
type="open-air";
high.altitude="n";
province="southern Andes";
technology="uniface/biface";
megafauna kill/scavenge="n";
};
Date(Prior("Quebrada Maní 35 (Date)","QM35_D.prior"))
{
site="Quebrada Maní 35";
latitude=-21.2;
longitude=-69.4;
country="Chile";
type="open-air";
high.altitude="n";
province="southern Andes";
technology="projectile point";
megafauna kill/scavenge="n";
};
Date(Prior("Quebrada Santa Julia LV. 221 (Date stratum 37)","QSJ_D_37.prior"))
{
site="Quebrada Santa Julia LV. 221";
latitude=-31.836917;
longitude=-71.488278;
country="Chile";
type="open-air";
high.altitude="n";
province="southern Andes";
technology="projectile point";
megafauna kill/scavenge="y";
};
Date(Prior("Quebrada Tacahuay (Date unit 5)","QT_D_5.prior"))
{
site="Quebrada Tacahuay";
latitude=-17.13;
longitude=-71.01;
country="Peru";
type="open-air";
high.altitude="n";
province="central Andes";
technology="uniface/biface";
megafauna kill/scavenge="n";
};
Date(Prior("Quebrada Tacahuay (Date unit 8)","QT_D_8.prior"))
{
site="Quebrada Tacahuay";
latitude=-17.13;
longitude=-71.01;
country="Peru";
type="open-air";
high.altitude="n";
province="central Andes";
technology="uniface/biface";
megafauna kill/scavenge="n";
};
Date(Prior("Quereo (Date level II)","Q_D_QuereoII.prior"))
{
site="Quereo";
latitude=-31.93;
longitude=-71.31;
country="Chile";
type="open-air";
high.altitude="n";
province="southern Andes";
technology="uniface/biface";
megafauna kill/scavenge="y";
};
Date(Prior("Ring Site (Date)","TRS_D.prior"))
{
site="Ring Site";
```

```
latitude=-17.667;
longitude=-71.333;
country="Peru";
type="open-air";
high altitude="n";
province="central Andes";
technology="uniface/biface";
megafauna kill/scavenge="n";
};
Date(Prior("RS-I-69 Laranjito (Date)","RSI_D.prior"))
{
site="RS-I-69 Laranjito";
latitude=-29.61333333;
longitude=-56.93;
country="Brazil";
type="open-air";
high altitude="n";
province="Brazilian Highlands";
technology="projectile point";
megafauna kill/scavenge="n";
};
Date(Prior("Salar de Punta Negra 1 (Date)","SPN1_D.prior"))
{
site="Salar de Punta Negra 1";
latitude=-24.466667;
longitude=-68.883333;
country="Chile";
type="open-air";
high altitude="y";
province="southern Andes";
technology="projectile point";
megafauna kill/scavenge="n";
};
Date(Prior("San Isidro (Date)","SIsi_D.prior"))
{
site="San Isidro";
latitude=2.4;
longitude=-76.5;
country="Colombia";
type="open-air";
high altitude="n";
province="northern Andes";
technology="uniface/biface";
megafauna kill/scavenge="n";
};
Date(Prior("San Lorenzo 1 (Date)","SLoren1_D.prior"))
{
site="San Lorenzo 1";
latitude=-23.4;
longitude=-67.9;
country="Chile";
type="cave";
high altitude="y";
province="southern Andes";
technology="uniface/biface";
megafauna kill/scavenge="n";
};
Date(Prior("San Ramón 15 (Date)","SR15_D.prior"))
{
site="San Ramón 15";
latitude=-25.3;
longitude=-70.4;
country="Chile";
type="open-air";
high altitude="n";
province="southern Andes";
technology="Huentelauquen";
megafauna kill/scavenge="n";
};
Date(Prior("Santa Elina (Date Unit II)","SE_D_II.prior"))
{
site="Santa Elina";
latitude=-15.4577778;
longitude=-56.7925;
country="Brazil";
type="rockshelter";
high altitude="n";
province="Brazilian Highlands";
technology="Itaparica";
megafauna kill/scavenge="y";
};
Date(Prior("Santana do Riacho (Date burial/cultural activity)","SR_D_humans.prior"))
{
site="Santana do Riacho";
latitude=-19.2;
longitude=-43.7;
country="Brazil";
type="rockshelter";
high altitude="n";
province="Brazilian Highlands";
technology="uniface/biface";
megafauna kill/scavenge="n";
};
Date(Prior("Tagua Tagua 1 & 2 (Date Paleoindian level)","TT_D_Pleis.prior"))
{
site="Tagua Tagua 1 & 2";
latitude=-34.4;
longitude=-71.2;
country="Chile";
type="open-air";
high altitude="n";
province="southern Andes";
technology="Fishtail";
megafauna kill/scavenge="y";
};
Date(Prior("Tagua Tagua 3 (date L4b facies)","TT4_D.prior"))
{
site="Tagua Tagua 3";
latitude=-34.4;
longitude=-71.2;
country="Chile";
type="open-air";
high altitude="n";
province="southern Andes";
technology="uniface/biface";
megafauna kill/scavenge="y";
};
Date(Prior("Cueva de la Vieja (Date)","CdlV_D.prior"))
{
site="Cueva de la Vieja";
latitude=-45.2742;
longitude=-71.54;
country="Chile";
type="cave";
high altitude="n";
province="southern Andes";
technology="uniface/biface";
megafauna kill/scavenge="n";
};
Date(Prior("Tequendama (Date stratum 5)","Teq_D_5.prior"))
{
site="Tequendama";
latitude=4.533;
longitude=-74.275;
country="Colombia";
type="rockshelter";
high altitude="n";
province="northern Andes";
technology="Tequendamiense";
megafauna kill/scavenge="y";
};
Date(Prior("Tequendama (Date stratum 6)","Teq_D_6.prior"))
{
site="Tequendama";
latitude=4.533;
longitude=-74.275;
country="Colombia";
type="rockshelter";
high altitude="n";
province="northern Andes";
technology="Tequendamiense";
megafauna kill/scavenge="y";
};
Date(Prior("Tequendama (Date stratum 7)","Teq_D_7.prior"))
{
site="Tequendama";
latitude=4.533;
longitude=-74.275;
country="Colombia";
type="rockshelter";
high altitude="n";
province="northern Andes";
technology="Tequendamiense";
megafauna kill/scavenge="y";
};
Date(Prior("Toca do Sítio do Meio (Date D upper)","TSM_D_upper.prior"))
{
site="Toca do Sítio do Meio";
latitude=-8.839167;
longitude=-42.563889;
country="Brazil";
type="rockshelter";
high altitude="n";
province="Brazilian Highlands";
technology="Itaparica";
megafauna kill/scavenge="n";
};
Date(Prior("Tres Arroyos 1 (Date level Va)","TA1_D_Va.prior"))
{
site="Tres Arroyos 1";
latitude=-53.383;
longitude=-68.783;
country="Chile";
type="rockshelter";
high altitude="n";
province="Patagonian Plateau";
technology="uniface/biface";
megafauna kill/scavenge="y";
};
Date(Prior("Tuina 1 (Date)","Tuina1_D.prior"))
{
site="Tuina 1";
latitude=-22.8;
longitude=-68.3;
country="Chile";
type="rockshelter";
high altitude="y";
province="southern Andes";
technology="uniface/biface";
megafauna kill/scavenge="n";
};
Date(Prior("Tulan 109 (Date)","Tulan9_D.prior"))
{
site="Tulan 109";
latitude=-23.6;
longitude=-67.9;
country="Chile";
type="cave";
high altitude="y";
province="southern Andes";
technology="uniface/biface";
megafauna kill/scavenge="n";
};
Date(Prior("Vale da Pedra Furada (Date horizon C2)","VPF_D_C2.prior"))
{
site="Vale da Pedra Furada";
latitude=-8.85;
longitude=-42.55;
country="Brazil";
type="open-air";
high altitude="n";
province="Brazilian Highlands";
technology="uniface/biface";
megafauna kill/scavenge="n";
};
Date(Prior("Vale da Pedra Furada (Date horizon C3)","VPF_D_C3.prior"))
{
site="Vale da Pedra Furada";
latitude=-8.85;
longitude=-42.55;
country="Brazil";
type="open-air";
high altitude="n";
province="Brazilian Highlands";
technology="uniface/biface";
megafauna kill/scavenge="n";
};
Date(Prior("Valiente CT14 (Date)","Valiente_D.prior"))
{
site="Valiente CT14";
latitude=-32.028333;
longitude=-71.160833;
country="Chile";
type="open-air";
high altitude="n";
province="southern Andes";
technology="Fishtail";
megafauna kill/scavenge="n";
};
Date(Prior("Taima-Taima (Date Unit 1)","TT_U1_D.prior"))
{
site="Taima-Taima";
latitude=11.43;
```

```
longitude=-69.75;
country="Venezuela";
type="open-air";
high altitude="n";
province="northern Andes";
technology="El Jobo";
megafauna kill/scavenge="y";
};
R_Date("Cuba (Date)",10619,66)
{
site="Cuba ";
material="charcoal";
latitude="5.197776";
longitude="-75.676397";
country="Colombia"type="open-air";
high altitude="n";
technology="uniface/biface";
megafauna kill/scavenge="n";
code="AA-102510";
};
R_Date("El Guatin (Date)",10130,50)
{
site="El Guatin";
material="charcoal";
latitude="4.75";
longitude="-75.7";
country="Colombia"type="open-air";
high altitude="n";
technology="uniface/biface";
megafauna kill/scavenge="n";
code="Beta-325213";
};
R_Date("El Jazmin (Date)",10120,70)
{
site="El Jazmin";
material="charcoal";
latitude="3.8";
longitude="-76.2";
country="Colombia"type="open-air";
high altitude="n";
technology="uniface/biface";
megafauna kill/scavenge="n";
code="unreported";
};
R_Date("La Mikela (Date)",10376,70)
{
site="La Mikela";
material="charcoal";
latitude="4.781956";
longitude="-75.68447";
country="Colombia"type="open-air";
high altitude="n";
technology="uniface/biface";
megafauna kill/scavenge="n";
code="AA-98939";
};
R_Date("La Morena (Date)",10090,60)
{
site="La Morena";
material="unreported";
latitude="6.2";
longitude="-75.5";
country="Colombia"type="open-air";
high altitude="n";
technology="uniface/biface";
megafauna kill/scavenge="n";
code="unreported";
};
R_Date("PIII0I-52 (Date)",10260,50)
{
site="PIII0I-52";
material="unreported";
latitude="6.8";
longitude="-75.1";
country="Colombia"type="open-air";
high altitude="n";
technology="uniface/biface";
megafauna kill/scavenge="n";
code="unreported";
};
R_Date("San Juan de Bedout 05PBE014 (Date)",10350,90)
{
site="San Juan de Bedout 05PBE014";
material="";
latitude="6.5";
longitude="-74.5";
country="Colombia"type="open-air";
high altitude="n";
technology="uniface/biface";
megafauna kill/scavenge="n";
code="no code San Juan";
};
R_Date("Tibitó 2 (Date)",11740,110)
{
site="Tibitó 2";
material="";
latitude="4.98";
longitude="-73.98";
country="Colombia"type="open-air";
high altitude="n";
technology="Abriense";
megafauna kill/scavenge="y";
code="GrN-9375";
};
R_Date("El Vano (Date)",10710,60)
{
site="El Vano";
material="";
latitude="-18.31";
longitude="-52.04";
country="Venezuela"type="rockshelter";
high altitude="n";
technology="Itaparica";
megafauna kill/scavenge="n";
code="Beta-95602";
};
Date(Prior("Cerro Azul (Date)","CA_D.prior"))
{
site="Cerro Azul";
latitude=2.5298;
longitude=-72.8664;
country="Colombia";
type="rockshelter";
high altitude="n";
province="Orinoco Lowlands";
technology="uniface/biface";
megafauna kill/scavenge="n";
};
R_Date("Cerro Montoya (Date)",10340,40)
{
site="Cerro Montoya";
material="seed";
latitude="2.5644";
longitude="-72.874";
country="Colombia"type="rockshelter";
high altitude="n";
province=Orinoco Lowlands;
technology="uniface/biface";
megafauna kill/scavenge="n";
code="Beta-509123";
};
R_Date("Limoncillos (Date)",10560,30)
{
site="Limoncillos";
material="seed";
latitude="2.5644";
longitude="-72.874";
country="Colombia"type="rockshelter";
high altitude="n";
province=Orinoco Lowlands;
technology="uniface/biface";
megafauna kill/scavenge="n";
code="Beta-509123 dup";
};
Curve("SHCal20","shcal20.14c");
R_Date("Amalia Sitio 2 (Date)",10425,75)
{
site="Amalia Sitio 2";
material="";
latitude="-37.8";
longitude="-58.10";
country="Argentina"type="rockshelter";
high altitude="n";
technology="Fishtail";
megafauna kill/scavenge="n";
code="AA-35499";
};
R_Date("Cueva El Abra (Date)",10270,200)
{
site="Cueva El Abra";
material="";
latitude="-37.4";
longitude="-58.90";
country="Argentina"type="cave";
high altitude="n";
technology="uniface/biface";
megafauna kill/scavenge="n";
code="AA-94641";
};
R_Date("Cueva La Brava (Date)",10178,54)
{
site="Cueva La Brava";
material="";
latitude="-37.8";
longitude="-58.1";
country="Argentina"type="cave";
high altitude="n";
technology="uniface/biface";
megafauna kill/scavenge="n";
code="AA-9463";
};
R_Date("El Trébol (Date)",10570,130)
{
site="El Trébol";
material="";
latitude="-41.07";
longitude="-71.49";
country="Argentina"type="cave";
high altitude="n";
technology="uniface/biface";
megafauna kill/scavenge="n";
code="AA-65707";
};
R_Date("Inca Cueva 4 (Date)",10620,140)
{
site="Inca Cueva 4";
material="";
latitude="-7.06";
longitude="-79.40";
country="Peru"type="open-air";
high altitude="n";
technology="Paiján";
megafauna kill/scavenge="n";
code="LP-137";
};
R_Date("La Cueva de Yavi (Date)",10450,55)
{
site="La Cueva de Yavi";
material="";
latitude="-9.75";
longitude="-48.37";
country="Brazil"type="open-air";
high altitude="n";
technology="uniface/biface";
megafauna kill/scavenge="n";
code="CSIC-1101";
};
R_Date("León Huasi (Date)",10550,300)
{
site="León Huasi";
material="";
latitude="-26.3";
longitude="-70.6";
country="Chile"type="open-air";
high altitude="n";
technology="Huentelauquén";
megafauna kill/scavenge="n";
code="GAK-13402";
};
R_Date("Los Toldos (Date)",12600,600)
{
site="Los Toldos";
material="";
latitude="-47.9";
longitude="-67.87";
country="Argentina"type="cave";
high altitude="n";
technology="uniface/biface";
megafauna kill/scavenge="n";
code="no code Los Toldos";
};
R_Date("Capivara 5 (Date)",10050,80)
{
```

```
site="Capivara 5";
material="";
latitude="-10.14";
longitude="-48.44";
country="Brazil";type="open-air";
high altitude="n";
technology="uniface/biface";
megafauna kill/scavenge="n";
code="Beta-179196";
};
R_Date("Coqueirinho (Date)",10460,60)
{
site="Coqueirinho";
material="";
latitude="-19.54";
longitude="-43.95";
country="Brazil";type="open-air";
high altitude="n";
technology="uniface/biface";
megafauna kill/scavenge="n";
code="Beta-237346";
};
R_Date("GO-JA-02 (Date)",10120,80)
{
site="GO-JA-02";
material="";
latitude="-18.45";
longitude="-52.01";
country="Brazil";type="rockshelter";
high altitude="n";
technology="Itaparica";
megafauna kill/scavenge="n";
code="SI-3108";
};
R_Date("GO-JA-14 (Date)",10740,85)
{
site="GO-JA-14";
material="";
latitude="-14.48";
longitude="-49.47";
country="Brazil";type="rockshelter";
high altitude="n";
technology="Itaparica";
megafauna kill/scavenge="n";
code="SI-3111";
};
R_Date("GO-NI-49 (Date)",10750,300)
{
site="GO-NI-49";
material="";
latitude="-23.00";
longitude="-65.45";
country="Argentina";type="rockshelter";
high altitude="y";
technology="uniface/biface";
megafauna kill/scavenge="n";
code="SI-2769";
};
R_Date("Lajeado 18 (Date)",10300,60)
{
site="Lajeado 18";
material="";
latitude="-19.52";
longitude="-44.07";
country="Brazil";type="rockshelter/cave";
high altitude="n";
technology="uniface/biface";
megafauna kill/scavenge="n";
code="Beta-179198";
};
R_Date("Lapa das Boleiras (Date)",10150,130)
{
site="Lapa das Boleiras";
material="";
latitude="-18.3";
longitude="-43.74";
country="Brazil";type="rockshelter/cave";
high altitude="n";
technology="uniface/biface";
megafauna kill/scavenge="n";
code="Beta-168451";
};
R_Date("Lapa do Peixe Gordo (Date)",10210,60)
{
site="Lapa do Peixe Gordo";
material="";
latitude="-28.6";
longitude="-65.6";
country="Argentina";type="rockshelter";
high altitude="n";
technology="uniface/biface";
megafauna kill/scavenge="n";
code="Beta-233762";
};
R_Date("MG-RP-6 Lapa do Gentio (Date)",10190,120)
{
site="MG-RP-6 Lapa do Gentio";
material="";
latitude="-16.26";
longitude="-46.05";
country="Brazil";type="rockshelter";
high altitude="n";
technology="Itaparica";
megafauna kill/scavenge="n";
code="SI-6837";
};
R_Date("Miracema do Tocantins 1 (Date)",10530,90)
{
site="Miracema do Tocantins 1";
material="";
latitude="-9.64";
longitude="-48.41";
country="Brazil";type="open-air";
high altitude="n";
technology="uniface/biface";
megafauna kill/scavenge="n";
code="Beta-190080";
};
R_Date("RS-I-66 Milton Almeida (Date)",10810,275)
{
site="RS-I-66 Milton Almeida";
material="";
latitude="-40.3";
longitude="-73.1";
country="Chile";type="open-air";
high altitude="n";
technology="uniface/biface";
megafauna kill/scavenge="n";
code="SI-2722";
};
R_Date("Sitio Toca dos Coqueiros (Date)",10640,50)
{
site="Sitio Toca dos Coqueiros";
material="";
latitude="-29.72";
longitude="-56.66";
country="Brazil";type="open-air";
high altitude="n";
technology="projectile point";
megafauna kill/scavenge="n";
code="Beta-104571";
};
R_Date("Toca da Boa Vista I (Date)",10530,110)
{
site="Toca da Boa Vista I";
material="";
latitude="-8.63";
longitude="-42.36";
country="Brazil";type="rockshelter/cave";
high altitude="n";
technology="uniface/biface";
megafauna kill/scavenge="n";
code="Beta-32971";
};
R_Date("Toca da Lagoa de Cima IX (Date)",10480,50)
{
site="Toca da Lagoa de Cima IX";
material="";
latitude="-8.7";
longitude="-42.6";
country="Brazil";type="rockshelter/cave";
high altitude="n";
technology="Itaparica";
megafauna kill/scavenge="n";
code="Beta-233909";
};
R_Date("Toca de Cima do Pilao (Date)",10390,80)
{
site="Toca de Cima do Pilao";
material="";
latitude="-8.87";
longitude="-42.59";
country="Brazil";type="rockshelter/cave";
high altitude="n";
technology="Itaparica";
megafauna kill/scavenge="n";
code="Beta-27345";
};
R_Date("Toca do Elias (Date)",10270,35)
{
site="Toca do Elias";
material="";
latitude="-8.84";
longitude="-42.56";
country="Brazil";type="rockshelter/cave";
high altitude="n";
technology="Itaparica";
megafauna kill/scavenge="n";
code="CAMS-95865";
};
R_Date("Toca do João Leite (Date)",10800,70)
{
site="Toca do João Leite";
material="";
latitude="-8.74";
longitude="-42.74";
country="Brazil";type="rockshelter/cave";
high altitude="n";
technology="Itaparica";
megafauna kill/scavenge="n";
code="Beta-220088";
};
R_Date("Alero Marifilo 1 (Date)",10190,120)
{
site="Alero Marifilo 1";
material="";
latitude="-39.51";
longitude="-72.06";
country="Chile";type="rockshelter";
high altitude="n";
technology="uniface/biface";
megafauna kill/scavenge="n";
code="Beta-164475";
};
R_Date("El Chueco 1 CIS 042 (Date)",10010,60)
{
site="El Chueco 1 CIS 042";
material="";
latitude="-44.4";
longitude="-71.30";
country="Chile";type="cave";
high altitude="n";
technology="uniface/biface";
megafauna kill/scavenge="n";
code="Beta-227703";
};
R_Date("Quebrada Saltillo (Date)",10260,60)
{
site="Quebrada Saltillo";
material="";
latitude="-6.87";
longitude="-79.42";
country="Peru";type="open-air";
high altitude="n";
technology="Paiján";
megafauna kill/scavenge="n";
code="Beta-191578";
};
R_Date("Río Bueno (Date)",10400,90)
{
site="Río Bueno";
material="";
latitude="-24.3";
longitude="-68.9";
country="Chile";type="open-air";
high altitude="y";
technology="projectile point";
megafauna kill/scavenge="n";
code="No code Río Bueno";
};
R_Date("Tuina 5 (Date)",10060,70)
{
site="Tuina 5";
```

```
  material="";
  latitude="-22.8";
  longitude="-68.3";
  country="Chile" type="cave";
  high altitude="y";
  technology="uniface/biface";
  megafauna kill/scavenge="n";
  code="Beta-107120";
 };
 R_Date("El Inga (Date)",10410,35)
 {
  site="El Inga";
  material="charcoal";
  latitude="-0.05";
  longitude="-78.55";
  country="Ecuador" type="open-air";
  high altitude="y";
  technology="Fishtal";
  megafauna kill/scavenge="n";
  code="PRI-13-029";
 };
 R_Date("CA-09-57/CA-09-55-2 (Date)",10360,100)
 {
  site="CA-09-57/CA-09-55-2";
  material="";
  latitude="-6.95";
  longitude="-79.24";
  country="Peru" type="open-air";
  high altitude="n";
  technology="Paiján";
  megafauna kill/scavenge="n";
  code="Beta-154141";
 };
 R_Date("CA-09-89 (Date)",11650,180)
 {
  site="CA-09-89";
  material="";
  latitude="-6.95";
  longitude="-79.24";
  country="Peru" type="open-air";
  high altitude="n";
  technology="Paiján";
  megafauna kill/scavenge="n";
  code="Beta-20887";
 };
 R_Date("Cerro Chivateros PV-46-27 (Date)",10430,160)
 {
  site="Cerro Chivateros PV-46-27";
  material="";
  latitude="-11.94";
  longitude="-77.13";
  country="Peru" type="open-air";
  high altitude="n";
  technology="uniface/biface";
  megafauna kill/scavenge="n";
  code="UCLA-683";
 };
 R_Date("Je-1002 (Date)",11014,64)
 {
  site="Je-1002";
  material="";
  latitude="-7.07";
  longitude="-79.41";
  country="Peru" type="open-air";
  high altitude="n";
  technology="Paiján";
  megafauna kill/scavenge="n";
  code="AA-57942";
 };
 R_Date("Je-439 (Date)",10056,67)
 {
  site="Je-439";
  material="";
  latitude="-22.12";
  longitude="-65.47";
  country="Argentina"type="cave";
  high altitude="n";
  technology="uniface/biface";
  megafauna kill/scavenge="n";
  code="AA-57950";
 };
 R_Date("PV-19-57-2 (Date)",10260,90)
 {
  site="PV-19-57-2";
  material="";
  latitude="-31.96";
  longitude="-71.5";
  country="Chile" type="open-air";
  high altitude="n";
  technology="uniface/biface";
  megafauna kill/scavenge="n";
  code="Beta-154128";
 };
 R_Date("PV-19-96-1 (Date)",10560,60)
 {
  site="PV-19-96-1";
  material="";
  latitude="-7.17";
  longitude="-79.52";
  country="Peru" type="open-air";
  high altitude="n";
  technology="Paiján";
  megafauna kill/scavenge="n";
  code="Beta-154123";
 };
 R_Date("Tres Ventanas 12b-VI-450 (Date)",10030,170)
 {
  site="Tres Ventanas 12b-VI-450";
  material="";
  latitude="-12.21";
  longitude="-76.38";
  country="Peru" type="open-air";
  high altitude="n";
  technology="Paiján";
  megafauna kill/scavenge="n";
  code="I-3091";
 };
 R_Date("Urupez II (Date)",11690,80)
 {
  site="Urupez II";
  material="";
  latitude="-34.83";
  longitude="-55.32";
  country="Uruguay"type="open-air";
  high altitude="n";
  technology="Fishtal";
  megafauna kill/scavenge="n";
  code="Beta-211398";
 };
 R_Date("Lapa do Dragao (Date)",11000,300)
 {
  site="Lapa do Dragao";
  material="";
  latitude="-14.4167";
  longitude="-44.4";
  country="Brazil" type="rockshelter";
  high altitude="n";
  province=Brazilian Highlands;
  technology="uniface/biface";
  megafauna kill/scavenge="n";
  code="CDTN-1007";
 };
};
```

*Figure 4*

```
Plot()
{
 Date(Prior("megafauna kill/scavenge","megafauna_S.prior"))
 {
  color="#9467bd";
 };
 KDE_Plot("KDE plot of LADs")
 {
  Curve("SHCal20","shcal20.14c");
  R_Date("Cuvieronis hyodon",11740,110);
  R_Date("Macrauchenia patachonica",11665,50);
  R_Date("Hemiauchenia",11320,90);
  R_Date("Eremotherium",10710,60);
  R_Date("Glossotherium",10500,90);
  R_Date("Megatherium americanum",10440,100);
  R_Date("Paleolama",10310,30);
  R_Date("Equus",10290,130);
  R_Date("Mylodon darwini",9700,100);
  R_Date("Notiomastodon platensis",9100,300);
  R_Date("Hippidion saldiasi",8850,80);
 };
};
```

## References


1. Boëda, E. *et al.* The chiquihuite cave, a real novelty? Observations about the still-ignored south American prehistory. *PaleoAmerica* **7**, 1–7 (2021).
2. Prates, L., Politis, G. G. & Perez, S. I. Rapid radiation of humans in South America after the last glacial maximum: A radiocarbon-based study. *PLoS One* **15**, e0236023 (2020).
3. Ugalde, P. C. *et al.* The first peoples of the Atacama Desert lived among the trees: A 11,600- to 11,200-year-old grove and congregation site. *Proc. Natl. Acad. Sci. U. S. A.* **121**, e2320506121 (2024).
4. Liu, Z. *et al.* Transient simulation of last deglaciation with a new mechanism for Bølling-Allerød warming. *Science* **325**, 310–314 (2009).
5. He, F. *Simulating transient climate evolution of the last deglaciation with CCSM 3*. (University of Wisconsin Madison, Madison, WC, USA, 2011).
6. GLOBAL CH.E.C. *PaleoView*. (2020).
7. Aceituno, F. J. & Rojas-Mora, S. Lithic technology studies in Colombia during the late Pleistocene and early Holocene. *Chungará (Arica)* 0–0 (2015).
8. Muttillo, B., Lembo, G., Rufo, E., Peretto, C. & Lleras Pérez, R. Revisiting the oldest known lithic assemblages of Colombia: A review of data from El Abra and Tibitó (Cundiboyacense Plateau, Eastern Cordillera, Colombia). *Journal of Archaeological Science: Reports* **13**, 455–465 (2017).
9. Correal, G. & Van der Hammen, T. Investigaciones arqueológicas en los abrigos rocosos del Tequendama. *Biblioteca Banco Popular, Bogotá* (1977).
10. Nieuwenhuis, C. J. Traces on tropical tools; a functional study of preceramic sites in Colombia. (University of Leiden, Netherlands, 2002).
11. Correal Urrego, G. Aclaraciones al texto 'Traces on tropical tools. A functional study of chert artefacts from preceramic sites in Colombia' (Nieuwenhuis, Channah José, 2002). *Maguaré* (2003).
12. Rouse, I. A lithic industry of Paleo-Indian type in Venezuela. *Am. Antiq.* **22**, 172–179 (1956).

13. Bryan, A. L., Casamiquela, R. M., Cruxent, J. M., Gruhn, R. & Ochsenius, C. An El Jobo Mastodon Kill at Taima-taima, Venezuela. *Science* **200**, 1275–1277 (1978).
14. Bird, J. B. *Travels and Archaeology in South Chile*. (University of Iowa Press, Unites States of America, 1988).
15. Bird, J. A comparison of south Chilean and Ecuadorian 'fishtail' projectile points. *Pap. Kroeber Anthropol. Soc.* **40**, 52–71 (1969).
16. Mazzanti, D. Secuencia arqueológica del sitio 2 de la localidad arqueológica Amalia (Provincia de Buenos Aires). *Del Mar a los Salitrales: Diez mil años de historia pampeana en el umbral del tercer milenio* 327–339 (2002).
17. López, C. E. Landscape development and the evidence for early human occupation in the inter-Andean tropical lowlands of the Magdalena River, Colombia. (Temple University, 2004).
18. Mazzanti, D., Martínez, G. & Quintana, C. Early settlements in eastern Tandilia, Buenos Aires Province, Argentina: archaeological contexts and site-formation processes. *Late Pleistocene Peopling of Latin America* 99–103 (2012).
19. Prates, L., Politis, G. & Steele, J. Radiocarbon chronology of the early human occupation of Argentina. *Quat. Int.* **301**, 104–122 (2013).
20. Suaréz, R. *Arqueología durante la Transición Pleistoceno-Holoceno en Uruguay: Componentes Paleoindios, Organización de la Tecnología Lítica y Movilidad de los Primeros Americanos: Componentes Paleoindios, Organización de la Tecnología Lítica y Movilidad de los Primeros Americanos*. (BAR Publishing, Oxford, England, 2011). doi:10.30861/9781407307787.
21. Meneghin, U. Un nuevo registro radiocarbónico (C14) en el yacimiento Urupez II, Maldonado, Uruguay. *Orígenes (Montevideo)* **5**, 1–7 (2006).
22. Loponte, D., Carbonera, M. & Silvestre, R. Fishtail projectile points from south America: The Brazilian record. *Archaeol. Discov.* **03**, 85–103 (2015).
23. Flegenheimer, N., Miotti, L. & Mazzia, N. Rethinking early objects and landscapes in the

Southern Cone: Fishtail-point concentrations in the Pampas and northern Patagonia. in *Paleoamerican Odyssey* (eds. Graf, K. E., Ketron, C. V. & Waters, M. R.) 259–376 (Texas A&M University Press, College Station, TX, 2013).

24. Suárez, R. The Paleoamerican Occupation of the Plains of Uruguay: Technology, Adaptations, and Mobility. *PaleoAmerica* **1**, 88–104 (2015).
25. Llagostera, A., Weisner, R., Castillo, G., Cervellino, M. & Costa-Junqueira, M. El Complejo Huentelauquén bajo una perspectiva macroespacial y multidisciplinaria. in *Actas del XIV Congreso Nacional de Arqueología Chilena* (ed. de Arqueología, S. C.) vol. 1 461–482 (Sociedad Chilena de Arqueología , 2000).
26. Salazar, D. *et al.* CRONOLOGÍA Y ORGANIZACIÓN ECONÓMICA DE LAS POBLACIONES ARCAICAS DE LA COSTA DE TALTAL. *Estud. atacam.* 07–46 (2015).
27. Salazar, D. *et al.* Nuevos sitios correspondientes al Complejo Cultural Huentelauquén en la costa de Taltal. *Taltalia* **5-6**, 9–19 (2013).
28. Llagostera, A. Early occupations and the emergence of fishermen on the pacific coast of south America. *Andean past* **3**, 9 (1992).
29. Salazar, D. *et al.* Early Evidence (ca. 12,000 BP) for Iron Oxide Mining on the Pacific Coast of South America. *Current Anthropology* **52**, 463–475 (2011).
30. Lourdeau, A. Industries lithiques du centre et du nord-est du Brésil pendant la transition Pléistocène–Holocène et l'Holocène ancien : la question du Technocomplexe Itaparica. *Anthropologie* **120**, 1–34 (2016).
31. Lourdeau, A. Lithic Technology and Prehistoric Settlement in Central and Northeast Brazil: Definition and Spatial Distribution of the Itaparica Technocomplex. *PaleoAmerica* **1**, 52–67 (2015).
32. Lourdeau, A. The Itaparica technocomplex: the first conspicuous settlement of central and northeastern Brazil from a technological perspective. *Southbound: Late Pleistocene peopling of Latin America. Current Research in the Pleistocene, Special Edition, Center for Study of the*

*First American, College Station, Texas* 53–56 (2012).

33. Kipnis, R. Foraging Societies of Eastern Central Brazil: An Evolutionary Ecological Study of Subsistence Strategies During the Terminal Pleistocene and Early/middle Holocene. (University of Michigan, 2002).
34. Chauchat, C. Limaces and Unifaces in the Paiján Industry, Peru, and the Early Prehistory of America. *Lithic Technology* **47**, 231–242 (2022).
35. Chauchat, C. El sitio pajanense Ascope 12 en la Quebrada de la Camotera y el grupo de Ascope, La Libertad, Perú. *Ñawpa Pacha* **32**, 1–42 (2012).
36. Pelegrin, J. & Chauchat, C. Tecnologia y funcion de las puntas de Paijan: El aporte de la experimentacion. *Latin American Antiquity* **4**, 367–382 (1993).
37. Dillehay, T. D. *From Foraging to Farming in the Andes: New Perspectives on Food Production and Social Organization*. (Cambridge University Press, 2011).
38. Chauchat, C. & Briceño, J. Paiján and fishtail points from Quebrada Santa María, North Coast of Perú. *Current Research in the Pleistocene*.
39. García, A., Zárate, M. & Paez, M. M. The Pleistocene/Holocene transition and human occupation in the Central Andes of Argentina: Agua de la Cueva locality. *Quat. Int.* **53-54**, 43–52 (1999).
40. Gil, A. F., Neme, G., Otaola, C. & García, A. Registro Arqueofaunistico en los Andes Meridionales Entre 11,000 Y 5000 Años A.P.: Evidencias en Agua de la Cueva-Sector Sur (Mendoza, Argentina). *Lat. Am. Antiq.* **22**, 595–617 (2011).
41. Lucero, G., Cortegoso, V. & Castro, S. Cazadores-Recolectores del Holoceno temprano: explotación de recursos líticos en el sitio Agua de la Cueva Sector Norte. in *Anales de arqueología y etnología* vol. 61 185–215 (academia.edu, 2006).
42. García, A. Bioturbación por Ctenomys mendocinus en el alero Agua de la Cueva. *Anales de Arqueología y Etnología* (2005).
43. Politis, G., Tonni, E. P., Fidalgo, F., Salemme, M. & Meo Guzmán, L. Man and Pleistocene

megamammals in the Argentine Pampa: site 2 at Arroyo Seco. *Current Research in the Pleistocene* **4**, 159–161 (1987).

44. Politis, G. G., Gutiérrez, M. A., Rafuse, D. J. & Blasi, A. The Arrival of Homo sapiens into the Southern Cone at 14,000 Years Ago. *PLoS One* **11**, e0162870 (2016).
45. Steele, J. & Politis, G. AMS 14C dating of early human occupation of southern South America. *J. Archaeol. Sci.* **36**, 419–429 (2009).
46. Politis, G. G., Barrientos, G. & Scabuzzo, C. *Estado Actual de Las Investigaciones En El Sitio Arroyo Seco 2 (Partido de Tres Arroyos, Provincia de Buenos Aires, Argentina)*. (INCUAPA-CONICET, Argentina, 2012).
47. Rafuse, D. J. Early to Middle Holocene subsistence strategies in the Pampas region: Evidence from the Arroyo Seco 2 site. *Journal of Archaeological Science: Reports* **12**, 673–683 (2017).
48. Politis, G. G., Barrientos, G. & Scabuzzo, C. Los entierros humanos de Arroyo Seco 2. in *Estado Actual de la Investigaciones en el sitio Arroyo Seco 2 (Partido de Tres Arroyos, provincia de Buenos Aires, Argentina)* (eds. Politis, G. G., Barrientos, G. & Scabuzzo, C.) (INCUAPA-CONICET, 2012).
49. Messineo, P. G. Lithic technology at Campo Laborde, an early-Holocene megamammal hunting site in the Pampean region (Argentina). (2012).
50. Messineo, P. G. & Pal, N. M. Techno-morphological and use-wear analysis on lithic and bone tools from Campo Laborde Site (Pampean Region, Argentina). (2011).
51. Messineo, P. G., Favier Dubois, C. M., Politis, G. G. & Vitale, P. Site formation process and megamammal bone radiocarbon dates in Campo Laborde (Pampas of Argentina): Contribution towards a research methodology. *Quat. Int.* **586**, 53–65 (2021).
52. Messineo, P. G. & Politis, G. G. New radiocarbon dates from the Campo Laborde site (Pampean Region, Argentina) support the Holocene survival of giant ground sloth and glyptodonts. *Current Research in the Pleistocene* **26**, 5–9 (2009).
53. Politis, G. G. & Messineo, P. G. The Campo Laborde site: New evidence for the Holocene

survival of Pleistocene megafauna in the Argentine Pampas. *Quat. Int.* **191**, 98–114 (2008).

54. Politis, G. G., Messineo, P. G., Stafford, T. W., Jr & Lindsey, E. L. Campo Laborde: A Late Pleistocene giant ground sloth kill and butchering site in the Pampas. *Sci Adv* **5**, eaau4546 (2019).
55. Paunero, R. S. *et al.* Análisis de la tecnología lítica del sitio Casa del Minero 1, Meseta Central de Santa Cruz. in *Arqueología de Fuego-Patagonia. Levantando piedras, desenterrando huesos... y develando arcanos* (eds. Morello, F., Prieto, A. & Bahamonde, G.) 589–600 (Centro de Estudios del Cuaternario Antártico (CEQUA), Punta Arenas, Chile, 2007).
56. Frank, A. D. Tratamiento y daño térmico de artefactos líticos en los componentes tempranos del sitio Casa del Minero 1, Santa Cruz, Argentina. *Chungará (Arica)* **44**, 25–37 (2012).
57. Paunero, R. *et al.* Sitio Casa del Minero 1, localidad arqueológica La María: nuevas evidencias sobre ocupación humana pleistocénica en Santa Cruz. in *XIV Congreso Nacional de Arqueología Argentina* (naturalis.fcnym.unlp.edu.ar, 2001).
58. Paunero, R. S. The presence of a Pleistocene colonizing culture in La Maria archaeological locality, Casa del Minero 1. in *Where the south winds blow: ancient evidence of Paleo South Americans* (eds. Miotti, L., Salemme, M. & Flegenheimer, N.) 127–132 (Center for the Study of the First Americans (CSFA), Texas A&M University, Texas, United States of America, 2003).
59. Cueto, M. E., Paunero, R. S. & Castro, A. S. La aplicación del análisis funcional sobre el conjunto artefactual lítico del componente temprano del sitio Casa del Minero 1 para la determinación de operaciones …. *XVIII Congreso Nacional de* (2012).
60. Flegenheimer, N. Excavaciones en el sitio 3, Cerro La China, Provincia de Buenos Aires. *Relaciones de la Sociedad Argentina de Antropología* (1987).
61. Zárate, M. & Flegenheimer, N. Geoarchaeology of the Cerro La China locality (Buenos Aires, Argentina): Site 2 and site 3. *Geoarchaeology* **6**, 273–294 (1991).
62. Flegenheimer, N. Recent research at localities cerro La China and cerro El Sombrero,

Argentina. *Current Research in the* (1987).

63. Paunero, R. S. El sitio Cueva 1 de la Localidad Arqueológica Cerro Tres Tetas (Estancia San Rafael, provincia de Santa Cruz, Argentina). in *Anales de Arqueología y Etnología* vol. 48 73–90 (1993).
64. Paunero, R. The Cerro Tres Tetas (C3T) locality in the central plateau of Santa Cruz, Argentina. in *Where the south winds blow: ancient evidence of Paleo South Americans* (eds. Miotti, L., Salemme, M. & Flegenheimer, N.) 133–140 (Center for the Study of the First Americans (CSFA), Texas A&M University, Texas, United States of America, 2003).
65. Paunero, R. S., Paunero, M. F. & Ramos, D. Artefactos óseos en componentes del Pleistoceno final de las Localidades La María y Cerro Tres Tetas, Santa Cruz, Argentina. *Primer Congreso Nacional de Zooarqueología* (2008).
66. Paunero, R. & Castro, A. Análisis lítico y funcionalidad del componente inferior del sitio Cueva 1, localidad arqueológica Cerro Tres Tetas, provincia de Santa Cruz, Argentina. in *Anales del Instituto de la Patagonia* (2001).
67. Paunero, R. S., Castro, A. & Reyes, M. Estudios Líticos del componente medio del sitio Cueva 1 de Cerro Tres Tetas, Santa Cruz, Argentina: Implicaciones para construir patrones de distribución artefactual y uso del microespacio. *Arqueología de Fuego Patagonia. Levantando piedras, desenterrando huesos… y develando arcanos* 613–622 (2007).
68. Paunero, R. Localidad Arqueológica Cerro Tres Tetas. *Guía de Campo de la Visita a las Localidades Arqueológicas* 89–100 (2000).
69. Paunero, R. S. Fogones, conjuntos líticos y funcionalidad en el componente pleistocénico del sitio Cueva 1 de Cerro Tres Tetas, provincia de Santa Cruz. in *Publicaciones del XIV Congreso Nacional de arqueología Argentina* vol. 2 419e428 (2009).
70. Frank, A. D. & Paunero, R. S. Análisis de la alteración térmica de los restos óseos procedentes del componente temprano de Cerro Tres Tetas (Meseta Central de Santa Cruz). Evidencia arqueológica y estudios experimentales. *Arqueología de Patagonia: una mirada desde el*

*último confín* **2**, 750–772 (2009).

71. Rivero, D. E. & Roldán, F. Initial peopling of Córdoba mountains, Argentina. First evidences from El Alto 3. *Center for the Study of the First Americans; Current Research in the Pleistocene* **22**, 12 (2005).
72. Rivero, D. E. Los primeros pobladores de las Sierras Centrales de Argentina. Las evidencias más antiguas del sitio 'El Alto 3' (Dpto. Punilla, Córdoba). **1**, 32–51 (2007).
73. Gil, A. F. Arqueología de La Payunia (Mendoza, Argentina): El poblamiento humano en los márgenes de la agricultura. (2006).
74. Long, A., Martin, P. S. & Lagiglia, H. A. Ground Sloth Extinction and Human Occupation at Gruta Del Indio, Argentina. *Radiocarbon* **40**, 693–700 (1997).
75. García, A. On the Coexistence of Man and Extinct Pleistocene Megafauna at Gruta Del Indio (Argentina). *Radiocarbon* **45**, 33–39 (2003).
76. D'antoni, H. Pollen analysis of Gruta del Indio. *Quaternary of South America and Antarctic* 83–104 (2020).
77. Martínez, G. 'Fish-tail' projectile points and megamammals: new evidence from Paso Otero 5 (Argentina). *Antiquity* **75**, 523–528 (2001).
78. Martínez, G., Gutiérrez, M. A. & Prado, J. New archaeological evidences from the late Pleistocene / early Holocene Paso Otero 5 site ( Pampean Region , Argentina ). *Current Research in the Pleistocene* **21**, 16–19 (2004).
79. Martínez, G. & Gutiérrez, M. Paso Otero 5: a summary of the interdisciplinary lines of evidence for reconstructing early human occupation and paleoenvironment in the Pampean region, Argentina. *Peuplements et Préhistoire de l'Amérique* 271–284 (2011).
80. Martínez, G., Gutiérrez, M. A. & Tonni, E. P. Paleoenvironments and faunal extinctions: Analysis of the archaeological assemblages at the Paso Otero locality (Argentina) during the Late Pleistocene–Early Holocene. *Quat. Int.* **299**, 53–63 (2013).
81. Miotti, L., Vázquez, M. & Hermo, D. Piedra Museo: un yamnagoo pleistocénico de los

colonizadores de la meseta de Santa Cruz. El estudio de la arqueofauna. *Soplando en el viento: Actas de las Terceras Jornadas de la Arqueología de la Patagonia* 113–136 (1999).

82. Miotti, L. Piedra Museo (Santa Cruz), nuevos datos para la ocupación Pleistocénica en Patagonia. *Actas de las Segundas Jornadas de la Arqueología de la Patagonia* 27–38 (1996).
83. Miotti, L., Salemme, M. & Rabassa, J. Radiocarbon chronology at Piedra Museo locality. in *Where the South Winds Blow: Ancient evidence of Paleo South Americans* (eds. Miotti, L., Salemme, M. & Flegenheimer, N.) 99–104 (Texas A & M University Press, College Station, TX, 2003).
84. Miotti, L. Piedra Museo locality: a special place in the New World. *Current Research in the Pleistocene* **12**, (1995).
85. Miotti, L. & Salemme, M. Hunting and butchering events at the Pleistocene/Holocene transition in Piedra Museo: an example of adaptation strategies of the first colonizers of Patagonia. *Paleoamerican origins: beyond Clovis* 209–220 (2005).
86. *Archaeology of Piedra Museo Locality: An Open Window to the Early Population of Patagonia*. (Springer Nature Switzerland AG, Switzerland, 2022).
87. Miotti, L., Mosquera, B., Salemme, M. & Rabassa, J. Radiocarbon chronology at the AEP-1 rockshelter in Piedra Museo locality: An update and discussion of the datings. in *Archaeology of Piedra Museo Locality* 111–125 (Springer International Publishing, Cham, 2022).
88. Politis, G. G., Johnson, E., Gutierrez, M. & Hartwell, W. T. Survival of the Pleistocene fauna: New radiocarbon dates on organic sediments from La Moderna (Pampean region, Argentina). *Americans. Center for the Study of …* (2003).
89. Politis, G. G. & Gutiérrez, M. A. Gliptodontes y Cazadores-Recolectores de la Region Pampeana (Argentina). *Latin American Antiquity* **9**, 111–134 (1998).
90. Gilson, S. & Bueno, L. New perspectives on old matters: A review of archaeological and chronometric data from Abrigo do Sol (Mato Grosso, Brazil). *Radiocarbon* **66**, 46–58 (2024).
91. Miller, E. Pesquisas arqueológicas paleoindígenas no Brasil ocidental. *Estud. atacameños* 39–

64 (1987).

92. Santos, G. M. *et al.* A revised chronology of the lowest occupation layer of Pedra Furada Rock Shelter, Piauí, Brazil: the Pleistocene peopling of the Americas. *Quat. Sci. Rev.* **22**, 2303–2310 (2003).

93. Meltzer, D. J., Adovasio, J. M. & Dillehay, T. D. On a Pleistocene human occupation at Pedra Furada, Brazil. *Antiquity* **68**, 695–714 (1994).

94. Bahn, P. G. Archaeology. 50,000-year-old Americans of Pedra Furada. *Nature* **362**, 114–115 (1993).

95. Guidon, N. *et al.* Pedra Furada, Brazil: Paleoindians, Paintings and Paradoxes, an interview. **3**, 42–52 (2002).

96. Guidon, N. On Stratigraphy and Chronology at Pedra Furada. *Curr. Anthropol.* **30**, 641–642 (1989).

97. Parenti, F. Problemática da pré-história do Pleistoceno Superior no Nordeste do Brasil: O abrigo da Pedra Furada em seu contexto regional. in *Anais da Conferência Internacional sobre Povoamento das Américas--Revista da Fundação Museu do Homem Americano* vol. 1 15–54 (1996).

98. Aimola, G., Andrade, C., Mota, L. & Parenti, F. Final Pleistocene and Early Holocene at Sitio do Meio, Piauí, Brazil: Stratigraphy and comparison with Pedra Furada. *Journal of Lithic Studies* **1**, 5–24 (2014).

99. Borrero, L. A. Human and natural agency: some comments on Pedra Furada. *Antiquity* **69**, 602–603 (1995).

100. Parenti, F. *Le gisement quaternaire de Pedra Furada (Piauí, Brésil): stratigraphie, chronologie, évolution culturelle*. (Editions Recherche sur les civilisations, 2001).

101. Parenti, F. Le gisement préhistorique du pléistocène supérieur de Pedra Furada (Piauí, Brésil). Considérations chronostratigraphiques et implications paléoanthropologiques. *Travaux et Documents des Laboratoires de Géologie de Lyon* **125**, 303–313 (1993).

102. Parenti, F. *et al.* Chronostratigraphie des gisements archéologiques et paléontologiques de Sao raimundo nonato (Piaui, Brésil): contribution à la connaissance du peuplement pléistocène de l'Amérique. in *14C et archeològie: 3éme Congrés International, Lyon 6-10 avril 1998* 327–332 (Société Préhistorique Française, 1999).

103. Parenti, F. Pedra Furada: A reappraisal of its artifacts, structures and stratigraphy. *Anthropologie* **127**, 103138 (2023).

104. Valladas, H. *et al.* TL age-estimates of burnt quartz pebbles from the Toca do Boqueirão da Pedra Furada (Piaui, Northeastern Brazil). *Quat. Sci. Rev.* **22**, 1257–1263 (2003).

105. Michab, M. *et al.* Luminescence dates for the Paleoindian site of Pedra Pintada, Brazil. *Quat. Sci. Rev.* **17**, 1041–1046 (1998).

106. Roosevelt, A. C. Relatório preliminar sobre o levantamento e escavações na Caverna da Pedra Pintada, Monte Alegre, Pará, Brasil. *Segunda etapa de campo do projeto arqueológico Santarém* (1991).

107. Roosevelt, A. C. *et al.* Paleoindian Cave Dwellers in the Amazon: The Peopling of the Americas. *Science* **272**, 373–384 (1996).

108. Roosevelt, A. C., Douglas, J. & Brown, L. The migrations and adaptations of the first Americans: Clovis and pre-Clovis viewed from South America. *The first Americans: the Pleistocene colonization of the New World* **27**, 159–236 (2002).

109. Rodet, M. J., Duarte-Talim, D., Pereira, E. & Moraes, C. New Data from Pedra Pintada Cave, Brazilian Amazon: Technological Analyses of the Lithic Industries in the Pleistocene–Holocene. *Latin American Antiquity* 1–21 (2023).

110. Pereira, E. da S. & Moraes, C. de P. A cronologia das pinturas rupestres da Caverna da Pedra Pintada, Monte Alegre, Pará: revisão histórica e novos dados. *Bol. Mus. Para. Emílio Goeldi. Ciênc. hum.* **14**, 327–342 (2019).

111. Fogaça, E. Instrumentos líticos unifaciais da transição Pleistoceno-Holoceno no Planalto Central do Brasil: Individualidade e especificidade dos objetos técnicos. *Canindé* **3**, 9–35

(2003).

112. Fogaça, E. A Tradição Itaparica e as indústrias líticas pré-cerâmicas da Lapa do Boquete (MG-Brasil). *Revista do Museu de Arqueologia e Etnologia* 145–158 (1995).

113. Prous, A. & Fogaça, E. Archaeology of the Pleistocene-Holocene boundary in Brazil. *Quat. Int.* **53-54**, 21–41 (1999).

114. Bueno, L., Dias, A. S. & Steele, J. The Late Pleistocene/Early Holocene archaeological record in Brazil: A geo-referenced database. *Quat. Int.* **301**, 74–93 (2013).

115. Fogaça, E., Sampaio, D. R. & Molina, L. A. Nas entrelinhas da tradição : os intrumentos de ocasião da Lapa do Boquete (Minas Gerais - Brasil). *rsab* **10**, 71–88 (1997).

116. Castro, S. S. A evolução do sítio arqueológico Lapa do Boquete na paisagem cárstica do Vale do Rio Peruaçu: Januária (MG). (1998).

117. Moura, M. T. A evolução do sítio arqueológico Lapa do Boquete na paisagem cárstica do Vale do Rio Peruaçu: Januária (MG). *São Paulo, SP: Universidade de São Paulo* (1997).

118. Prous, A. Fouilles de l’Abri du Boquete, Minas Gerais, Brésil. *Journal de la Société des américanistes* **77**, 77–109 (1991).

119. Feathers, J., Kipnis, R., Piló, L., Arroyo-Kalin, M. & Coblentz, D. How old is Luzia? Luminescence dating and stratigraphic integrity at Lapa Vermelha, Lagoa Santa, Brazil. *Geoarchaeology* **16**, (2010).

120. Neves, W. A., Powell, J. F., Prous, A., Ozolins, E. G. & Blum, M. Lapa vermelha IV Hominid 1: morphological affinities of the earliest known American. *Genet. Mol. Biol.* **22**, 461–469 (1999).

121. Fontugne, M. New Radiocarbon Ages of Luzia Woman, Lapa Vermelha IV Site, Lagoa Santa, Minas Gerais, Brazil. *Radiocarbon* **55**, 1187–1190 (2013).

122. Vialou, A. V. *Pré-história do Mato Grosso: Santa Elina*. (EdUSP, 2005).

123. Vialou, A. V. Santa Elina rockshelter, Brazil: Evidence of the coexistence of Man and Glossotherium. in *Where the South Winds Blow: Ancient evidence of Paleo South Americans*

(eds. Miotti, L., Salemme, M. & Flegenheimer, N.) 21–28 (Texas A & M University Press, College Station, TX, 2003).

124. Vilhena Vialou, A. *et al.* Decouverte de Mylodontinae dans un habitat prehistorique date du Mato Grosso (Bresil) l'abri rupestre de Santa Elina. *Comptes Rendus de l'Academie des Sciences-Serie II-Sciences de la Terre et des Planetes* **320**, 655–662 (1995).

125. Vialou, D., Benabdelhadi, M., Feathers, J., Fontugne, M. & Vialou, A. V. Peopling South America's centre: the late Pleistocene site of Santa Elina. *Antiquity* **91**, 865–884 (2017).

126. Pansani, T. R. *et al.* Evidence of artefacts made of giant sloth bones in central Brazil around the last glacial maximum. *Proc. Biol. Sci.* **290**, 20230316 (2023).

127. Pansani, T. R. *et al.* Radiocarbon dating and isotopic palaeoecology of Glossotherium phoenesis from the Late Pleistocene of the Santa Elina rock shelter, Central Brazil. *J. Quat. Sci.* **39**, 1186–1199 (2023).

128. Feathers, J. Datação por luminescência óptica estimulada. in *Pré-história do Mato Grosso: Vol. 1 Santa Elina* (ed. Vialou, A. V.) 55–59 (Editora da Universidade de São Paulo, São Paulo, Brazil, 2005).

129. Dias, A. S. & Jacobus, A. L. Quão antigo é o povoamento do sul do Brasil. *Revista do CEPA* **27**, 39–67 (2003).

130. Neves, W. A., Prous, A., González-José, R., Kipnis, R. & Powell, J. Early Holocene human skeletal remains from Santana do Riacho, Brazil: implications for the settlement of the New World. *J. Hum. Evol.* **45**, 19–42 (2003).

131. Araujo, A. G. M., Neves, W. A., Piló, L. B. & Atui, J. P. V. Holocene Dryness and Human Occupation in Brazil During the 'Archaic Gap'. *Quat. Res.* **64**, 298–307 (2005).

132. Kipnis, R. Early hunter-gatherers in the Americas: perspectives from central Brazil. *Antiquity* **72**, 581–592 (1998).

133. de Sousa, D. V. *et al.* Archaeoanthrosol formation and evolution of the 'Santana do Riacho' archaeological shelter: An old burial site in South America. *Geoarchaeology* **32**, 678–693

(2017).

134. Prous, A. As estruturas aparentes: os sepultamentos do Grande Abrigo de Santana do Riacho. Os sepultamentos da escavação n 1. *Arquivos do Museu de História natural da UFMG* (1992).

135. Parenti, F., Faure, M., Da Luz, F. & Guérin, C. Pleistocene faunas and lithic industries in the Antonião Rockshelter (Coronel José Dias, Piauí, Brazil): studying their association. *Current Research in the Pleistocene* **19**, 89–91 (2002).

136. Lahaye, C. *et al.* Another site, same old song: The Pleistocene-Holocene archaeological sequence of Toca da Janela da Barra do Antonião-North, Piauí, Brazil. *Quat. Geochronol.* **49**, 223–229 (2019).

137. Boëda, E. *et al.* New Data on a Pleistocene Archaeological Sequence in South America: Toca do Sítio do Meio, Piauí, Brazil. *PaleoAmerica* **2**, 286–302 (2016).

138. Guidon, N. & Andreatta, M. D. O sítio arqueológico Toca do Sítio do Meio (Piauí). *Clio* **3**, (1980).

139. Mota, L. & Scheel-Ybert, R. Antracologia no Parque Nacional Serra da Capivara (Piauí-Brasil): primeiros dados sobre a Toca do Boqueirão da Pedra Furada e a Toca do Sítio do Meio. *rsab* **32**, 197–224 (2019).

140. Guidon, N., Peyre, E., Guérin, C. & Coppens, Y. Resultados da datação de dentes humanos da Toca do Garrincho, Piauí, Brasil. *Clio* **14**, 75–86 (2000).

141. Peyre, A., Granat, J. & Guidon, N. Dents et crânes humains fossiles du Garrincho (Brésil) et peuplements anciens de l'Amérique. *Actes Societe francaise d'histoire de l'art dentaire* **14**, 32–37 (2009).

142. Peyre, E., Guérin, C., Guidon, N. & Coppens, Y. Des restes humains pléistocènes dans la grotte du Garrincho, Piauí, Brésil. *Comptes Rendus de l'Académie des Sciences-Series IIA-Earth and Planetary Science* **327**, 355–360 (1998).

143. Lahaye, C. *et al.* New insights into a late-Pleistocene human occupation in America: The Vale da Pedra Furada complete chronological study. *Quat. Geochronol.* **30, Part B**, 445–451

(2015).

144. Boëda, E. *et al.* A new late Pleistocene archaeological sequence in South America: the Vale da Pedra Furada (Piauí, Brazil). *Antiquity* **88**, 927–941 (2014).
145. Boëda, E. *et al.* 24.0 kyr cal BP stone artefact from Vale da Pedra Furada, Piauí, Brazil: Techno-functional analysis. *PLoS One* **16**, e0247965 (2021).
146. Parenti, F. Old and new on the same site: Putting Vale da Pedra Furada into a wider context. A comment to Lahaye et al. 2015. *Quat. Geochronol.* **30**, 48–53 (2015).
147. Capriles, J. M. *et al.* High-altitude adaptation and late Pleistocene foraging in the Bolivian Andes. *Journal of Archaeological Science: Reports* **6**, 463–474 (2016).
148. Capriles, J. M. & Albarracin-Jordan, J. The earliest human occupations in Bolivia: A review of the archaeological evidence. *Quat. Int.* **301**, 46–59 (2013).
149. Capriles, J. M. *et al.* Late Pleistocene Lithic Procurement and Geochemical Characterization of the Cerro Kaskio Obsidian Source in South-western Bolivia. *Archaeometry* **60**, 898–914 (2018).
150. Prieto, A. Cazadores Tempranos y Tardios en Cueva del Lago Sofia 1. *Ans. Inst. Pat. Ser. Cs. Soc.* **20**, 75–96 (1991).
151. Jackson, D. & Prieto, A. Estrategias tecnológicas y conjunto lítico del contexto paleoindio de Cueva de Lago Sofía 1, Ultima Esperanza, Magallanes. *Magallania* **33**, 115–120 (2005).
152. Waters, M. R., Amorosi, T. & Stafford, T. W. Redating Fell's Cave, Chile and the Chronological Placement of the Fishtail Projectile Point. *Am. Antiq.* **80**, 376–386 (2015).
153. Martin, F. M. Cueva Fell reinterpretada. *Chungará (Arica)* (2022).
154. Amorosi, T. & Prevosti, F. J. A preliminary review of the canid remains from Junius Bird's excavations at Fell's and Pali Aike caves, Magallanes, Chile. *Current Research in the Pleistocene* **25**, 25–27 (2008).
155. Clutton-Brock, J. The carnivore remains excavated at Fell's Cave in 1970. in *Travels and Archaeology in South Chile* (ed. Hyslop, J.) 188–195 (University of Iowa Press, Iowa, 1988).

156. Flegenheimer, N. & Cattáneo, R. Análisis comparativo de desechos de talla en contextos del Pleistoceno final/Holoceno temprano de Chile y Argentina. *Magallania* **41**, 171–192 (2013).

157. Nami, H. G. Technological observations on the Paleoindian artifacts from Fell's Cave, Magallanes, Chile. *Current Research in the Pleistocene* **15**, 81–83 (1998).

158. Scheinsohn, V. A hook on Patagonia: spearthrowers, bone hooks, and grips from Patagonia. *Cuadernos del Instituto Nacional de Antropología y Pensamiento Latinoamericano* **3**, 88–102 (2016).

159. Méndez, C. *et al.* The initial peopling of Central Western Patagonia (southernmost South America): Late Pleistocene through Holocene site context and archaeological assemblages from Cueva de la Vieja site. *Quat. Int.* **473**, 261–277 (2018).

160. Nami, H. G. & Nakamura, T. Cronología radiocarbónica con AMS sobre muestras de hueso procedentes del sitio Cueva del Medio (Última Esperanza, Chile). *An. Inst. Patagonia* **23**, (1995).

161. Nami, H. G. & Heusser, C. J. Cueva del Medio: A Paleoindian Site and Its Environmental Setting in Southern South America. *Archaeological Discovery* **03**, 62–71 (2015).

162. Nami, H. G. Cueva del Medio: perspectivas arqueológicas para la Patagonia Austral. *An. Inst. Patagonia* **17**, 71–106 (1987).

163. Nami, H. G. Resumen de las actividades y nuevos datos obtenidos en la quinta campaña de investigaciones arqueológicas en Ultima Esperanza, Chile. *Palimpsesto. Revista de Arqueología.* **2**, (1992).

164. Martin, F. M. *et al.* The Cave at the End of the World. Cueva del Medio and the Early Colonization of Southern South America. in *People and Culture in Ice Age Americas: New Dimensions in Paleoamerican Archaeology* (eds. Suarez, R. & Ardelean, C. F.) 1–12 (University of Utah Press, Salt Lake City, UT, 2019).

165. Martin, F. M. *et al.* Nuevas excavaciones en Cueva del Medio: Procesos de formación de la cueva y avances en los estudios de interacción entre cazadores-recolectores y fauna extinta

(Pleistoceno Final, Patagonia Meridional). *Magallania* **43**, 165–189 (2015).

166. Jackson, D., Méndez, C. & Aspillaga, E. Human Remains Directly Dated to the Pleistocene-Holocene Transition Support a Marine Diet for Early Settlers of the Pacific Coast of Chile. *The Journal of Island and Coastal Archaeology* **7**, 363–377 (2012).
167. Govan, E. & Parnell, A. *Simmr: Stable Isotope Mixing Models for R*. (2023).
168. Alfonso-Durruty, M., Troncoso, A., Larach, P., Becker, C. & Misarti, N. Maize (Zea mays) consumption in the southern andes (30°-31° S. Lat): Stable isotope evidence (2000 BCE-1540 CE). *Am. J. Phys. Anthropol.* **164**, 148–162 (2017).
169. Fernandes, R., Grootes, P., Nadeau, M.-J. & Nehlich, O. Quantitative diet reconstruction of a Neolithic population using a Bayesian mixing model (FRUITS): The case study of Ostorf (Germany). *Am. J. Phys. Anthropol.* **158**, 325–340 (2015).
170. Macario, K. D. *et al.* Marine reservoir effect on the Southeastern coast of Brazil: results from the Tarioba shellmound paired samples. *J. Environ. Radioact.* **143**, 14–19 (2015).
171. Carré, M., Jackson, D., Maldonado, A., Chase, B. M. & Sachs, J. P. Variability of 14C reservoir age and air–sea flux of CO2 in the Peru–Chile upwelling region during the past 12,000 years. *Quat. Res.* **85**, 87–93 (2016).
172. Santoro, C. M. *et al.* From the Pacific to the tropical forests: networks of social interaction in the Atacama Desert, late in the Pleistocene. *Chungará (Arica)* **51**, 5–25 (2019).
173. Dillehay, T. D. & Collins, M. B. Early cultural evidence from Monte Verde in Chile. *Nature* **332**, 150–152 (1988).
174. Dillehay, T. D. *Monte Verde, a Late Pleistocene Settlement in Chile: The Archaeological Context and Interpretation*. vol. 2 (Smithsonian Institution Press, 1997).
175. Meltzer, D. J. *et al.* On the Pleistocene Antiquity of Monte Verde, Southern Chile. *Am. Antiq.* **62**, 659–663 (1997).
176. Dillehay, T. D. *Monte Verde, A Late Pleistocene Settlement in Chile: Paleo-Environment and Site Context*. vol. 1 (Smithsonian Institution Press, Washington, 1997).

177. Dillehay, T. D. *et al.* New Archaeological Evidence for an Early Human Presence at Monte Verde, Chile. *PLoS One* **10**, e0141923 (2015).

178. Pino, M. & Dillehay, T. D. Monte Verde II: an assessment of new radiocarbon dates and their sedimentological context. *Antiquity* **97**, 524–540 (2023).

179. Pino, M., Chávez-Hoffmeister, M., Navarro-Harris, X. & Labarca, R. The late Pleistocene Pilauco site, Osorno, south-central Chile. *Quat. Int.* **299**, 3–12 (2013).

180. Moreno, K. *et al.* A late Pleistocene human footprint from the Pilauco archaeological site, northern Patagonia, Chile. *PLoS One* **14**, e0213572 (2019).

181. Navarro-Harris, X. *et al.* The procurement and use of knappable glassy volcanic raw material from the late Pleistocene Pilauco site, Chilean Northwestern Patagonia. *Geoarchaeology* **34**, 592–612 (2019).

182. Pino, M. & Astorga, G. A. *Pilauco: A Late Pleistocene Archaeo-Paleontological Site: Osorno, Northwestern Patagonia and Chile*. (Springer, 2020).

183. Béarez, P., Jackson, D. & Mollaret, N. Early Archaic Fishing (12,600–9,200 cal yr BP) in the Semiarid North Coast of Chile. *The Journal of Island and Coastal Archaeology* **10**, 133–148 (2015).

184. Jackson, D., Báez, P. & Seguel, R. Nuevas evidencias estratigráficas para el complejo Huentelauquén en la Provincia del Choapa, IV Región. *Revista Chilena de Antropología* **14**, (1997).

185. Méndez, C. & Nuevo-Delaunay, A. The Long-Term Relation Between Human Beings and Shellfish in the Semiarid Coast of Chile. in *South American Contributions to World Archaeology* (eds. Bonomo, M. & Archila, S.) 119–140 (Springer International Publishing, Cham, 2021).

186. Quintana, R. S. El sitio arqueológico Punta Ñagué: procesos de transformación y estrategias de conservación durante su excavación. *Conserva* **5**, 39–51 (2001).

187. Román B, A. & Jackson S, D. Dataciones por termoluminiscencia de rocas de fogones de

asentamientos Arcaicos, Provincia de Choapa. *Chungará (Arica)* **30**, 41–48 (1998).

188. Jackson, D. Datación radiocarbónica para una adaptación costera del arcaico temprano en el Norte Chico, comuna de Los Vilos. *Boletín de la Sociedad Chilena de Arqueología* **16**, 28–31 (1993).

189. Casamiquela, R., Montané, J. & Santana, R. Convivencia del hombre con el mastodonte en Chile central. *Noticiario Mensual del Museo Nacional de Historia Natural* **11**, 1–6 (1967).

190. Montané, J. Paleo-Indian remains from laguna de tagua tagua, central chile. *Science* **161**, 1137–1138 (1968).

191. Nuñez, L. *et al.* Cuenca de Taguatagua en Chile: el ambiente del Pleistoceno superior y ocupaciones humanas. *Revista Chilena de Historia Natural* **67**, 503–519 (1994).

192. Labarca, R. *et al.* Taguatagua 3: A new late Pleistocene settlement in a highly suitable lacustrine habitat in central Chile (34°S). *PLoS One* **19**, e0302465 (2024).

193. Jackson, D., Méndez, C., Núñez, L. & Jackson, D. Procesamiento de fauna extinta durante la transición Pleistoceno-Holoceno en el centro-norte de Chile. *Boletín de Arqueología PUCP* 315–336 (2011).

194. Montané, J. Primera fecha radiocarbónica de Tagua-Tagua. *Not. Mens. Mus. Nac. Hist. Nat.* **139**, (1968).

195. Labarca, R. *et al.* Taguatagua 1: New insights into the late Pleistocene fauna, paleoenvironment, and human subsistence in a unique lacustrine context in central Chile. *Quat. Sci. Rev.* **238**, 106282 (2020).

196. Miotti, L. & Salemme, M. C. When Patagonia was colonized: people mobility at high latitudes during Pleistocene/Holocene transition. *Quat. Int.* **109–110**, 95–111 (2003).

197. Massone, M. Fell 1 hunters' fire hearths in Magallanes area by the end of the Pleistocene. in *Where the South Winds Blow: Ancient evidence of Paleo South Americans* (eds. Miotti, L., Salemme, M. & Flegenheimer, N.) 153–159 (Texas A & M University Press, College Station, TX, 2003).

198. Massone, M. Los cazadores paleoindios de Tres Arroyos (Tierra del Fuego). in *Anales del Instituto de la Patagonia* (1987).

199. Borrero, L. A. Taphonomy of the Tres Arroyos 1 Rockshelter, Tierra del Fuego, Chile. *Quat. Int.* **109–110**, 87–93 (2003).

200. Latorre, C. *et al.* Late Pleistocene human occupation of the hyperarid core in the Atacama Desert, northern Chile. *Quat. Sci. Rev.* **77**, 19–30 (2013).

201. Santoro, C. M. *et al.* Ocupación humana pleistocénica en el desierto de Atacama: primeros resultados de la aplicación de un modelo predictivo de investigación interdisciplinaria. *Chungará (Arica)* **43**, 353–366 (2011).

202. Workman, T. R. *et al.* Landscape evolution and the environmental context of human occupation of the southern pampa del tamarugal, Atacama Desert, Chile. *Quat. Sci. Rev.* **243**, 106502 (2020).

203. Joly, D. *et al.* Late Pleistocene fuel management and human colonization of the Atacama Desert, northern Chile. *Latin American Antiquity* **28**, 144–160 (2017).

204. Herrera, K. A., Pelegrin, J., Gayo, E. & Santoro, C. M. Circulation of Objects and Raw Material in the Atacama Desert, Northern Chile by the End of the Pleistocene. *PaleoAmerica* **5**, 335–348 (2019).

205. Merino-Campos, V., De Pol-Holz, R., Southon, J., Latorre, C. & Collado-Fabbri, S. Marine Radiocarbon Reservoir Age Along the Chilean Continental Margin. *Radiocarbon* **61**, 195–210 (2019).

206. Heaton, T. J. *et al.* Marine20—The Marine Radiocarbon Age Calibration Curve (0–55,000 cal BP). *Radiocarbon* **62**, 779–820 (2020).

207. Reimer, R. W. & Reimer, P. J. An online application for ΔR calculation. *Radiocarbon* **59**, 1623–1627 (2017).

208. Jackson, D., Méndez, C., Seguel, R., Maldonado, A. & Vargas, G. Initial Occupation of the Pacific Coast of Chile during Late Pleistocene Times. *Curr. Anthropol.* **48**, 725–731 (2007).

209. Méndez, C., Jackson, D., Seguel, R. & Delaunay, A. N. Early high-quality lithic procurement in the semiarid north of Chile. *Current Research in the Pleistocene* **27**, 19–21 (2010).

210. Maldonado, A. *et al.* Early Holocene climate change and human occupation along the semiarid coast of north-central Chile. *J. Quat. Sci.* **25**, 985–988 (2010).

211. Núñez, L., Varela, J., Casamiquela, R. & Villagrán, C. Reconstruccion Multidisciplinaria de la Ocupacion Prehistorica de Quereo, Centro de Chile. *Latin American Antiquity* **5**, 99–118 (1994).

212. Jackson, D., Méndez, C. & De Souza, P. Poblamiento Paleoindio en el norte-centro de Chile: Evidencias, problemas y perspectivas de estudio. *Complutum* **15**, 165–176 (2004).

213. Aceituno, F. J., Loaiza, N., Delgado-Burbano, M. E. & Barrientos, G. The initial human settlement of Northwest South America during the Pleistocene/Holocene transition: Synthesis and perspectives. *Quat. Int.* **301**, 23–33 (2013).

214. Hurt, W. R., van der Hammen, T. & Urrego, G. C. Preceramic Sequences in the El Abra Rock-Shelters, Colombia. *Science* **175**, 1106–1108 (1972).

215. Correal, G. Apuntes sobre el medio ambiente pleistocénico y el hombre prehistórico en Colombia. *New evidence for the Pleistocene peopling of the Americas* 115–131 (1986).

216. Muttillo, B., Lleras Pérez, R., Rufo, E. & Lembo, G. Revisiting the lithic industries of El Abra sites (Sabana de Bogotá, Colombia, Northern South America). Implications for its significance and chronology. *Quat. Int.* **578**, 35–46 (2021).

217. Correal, G., Van Der Hammen, T. & Lerman, J. C. Artefactos líticos de abrigos rocosos en: El Abra, Colombia. *rev.colomb.antropol.* **14**, 11–46 (1969).

218. Hurt, W., van der Hammen, T. & Correal, G. *The El Abra Rockshelters, Sabana de Bogotá, Colombia, South America. Occasional Papers and Monographs No. 2*. (Indiana University, Bloomington, 1977).

219. Aceituno, F. J. & Loaiza, N. *Domesticación del bosque en el Cauca medio colombiano entre el Pleistoceno final y el Holoceno medio*. (BAR Publishing, Oxford, England, 2019).

doi:10.30861/9781407300900.

220. Aceituno-Bocanegra, F. J. & Uriarte, A. Mobility and human dispersion during the peopling of northwest south America between the late Pleistocene and the early Holocene. in *Suárez, Rafael; Ardelean, Ciprian F. (eds.). People and Culture in Ice Age Americas: New Dimensions in Paleoamerican Archaeology. University of Utah Press:* vol. 6 7–92 (University of Utah, 2021).
221. Morcote-Ríos, G., Aceituno, F. J., Iriarte, J., Robinson, M. & Chaparro-Cárdenas, J. L. Colonisation and early peopling of the Colombian Amazon during the Late Pleistocene and the Early Holocene: New evidence from La Serranía La Lindosa. *Quat. Int.* **578**, 5–19 (2021).
222. Correal Urrego, G. & van der Hammen, T. *Investigaciones arqueologicas en los abrigos rocosos del tequendama*. (Biblioteca Banco Popular, Bogotá, Colombia, 1977).
223. Triana-Vega, A. V. *et al.* Environmental reconstruction spanning the transition from hunter/gatherers to early farmers in Colombia: paleopedological and archaeological indicators from the pre-ceramic sites Tequendama and Aguazuque. *Quat. Int.* **516**, 175–189 (2019).
224. Delgado, M., Aceituno, F. J. & Barrientos, G. 14C data and the early colonization of Northwest South America: A critical assessment. *Quat. Int.* **363**, 55–64 (2015).
225. Correal Urrego, G. Restos de megafauna asociadas a artefactos en la sabana de bogotá. *Caldasia* **13**, 487–547 (1982).
226. Rademaker, K. *et al.* Paleoindian settlement of the high-altitude Peruvian Andes. *Science* **346**, 466–469 (2014).
227. Meinekat, S. A., Miller, C. E. & Rademaker, K. A site formation model for Cuncaicha rock shelter: Depositional and postdepositional processes at the high-altitude keysite in the Peruvian Andes. *Geoarchaeology* **37**, 304–331 (2022).
228. Rademaker, K. & Hodgins, G. Exploring the chronology of occupations and burials at Cuncaicha rockshelter, Peru. *New Perspectives on the Peopling of the Americas, Kerns Verlag, Tuebingen* 107–124 (2018).

229. Francken, M., Beier, J., Reyes-Centeno, H., Harvati, K. & Rademaker, K. The human skeletal remains from Cuncaicha rockshelter, Peru. in *New Perspectives on the Peopling of the Americas* (eds. Harvati, K., Jäger, G. & Reyes-Centeno, H.) 125–152 (Kerns Verlag, Tübingen, Germany, 2018).
230. Karakostis, F. A. *et al.* Biocultural evidence of precise manual activities in an Early Holocene individual of the high-altitude Peruvian Andes. *Am. J. Phys. Anthropol.* **174**, 35–48 (2021).
231. Jolie, E. A., Lynch, T. F., Geib, P. R. & Adovasio, J. M. Cordage, Textiles, and the Late Pleistocene Peopling of the Andes. *Curr. Anthropol.* **52**, 285–296 (2011).
232. Lynch, T. F. & Kennedy, K. A. Early human cultural and skeletal remains from guitarrero cave, northern peru. *Science* **169**, 1307–1309 (1970).
233. Lynch, T. F. *Guitarrero Cave: Early Man in the Andes*. (Academic Press, Inc., London, 1980).
234. Lynch, T. F., Gillespie, R., Gowlett, J. A. & Hedges, R. E. Chronology of guitarrero cave, peru. *Science* **229**, 864–867 (1985).
235. Adovasio, J. M. & Maslowski, R. F. Cordage, Basketry, and Textiles. in *Guitarrero Cave* (ed. Lynch, T. F.) 253–290 (Academic Press, 1980).
236. Adovasio, J. M. & Lynch, T. F. Preceramic Textiles and Cordage From Guitarrero Cave, Peru. *Am. Antiq.* **38**, 84–90 (1973).
237. Kaplan, L. & Lynch, T. F. Phaseolus (Fabaceae) in Archaeology: AMS Radiocarbon Dates and Their Significance for Pre-Colombian Agriculture. *Econ. Bot.* **53**, 261–272 (1999).
238. Dillehay, T. D. *et al.* A late pleistocene human presence at Huaca Prieta, Peru, and early Pacific Coastal adaptations. *Quat. Res.* **77**, 418–423 (2012).
239. Dillehay, T. D. *et al.* Simple technologies and diverse food strategies of the Late Pleistocene and Early Holocene at Huaca Prieta, Coastal Peru. *Sci Adv* **3**, e1602778 (2017).
240. Dillehay, T. D. *et al.* Chronology, mound-building and environment at Huaca Prieta, coastal Peru, from 13 700 to 4000 years ago. *Antiquity* **86**, 48–70 (2012).
241. Bird, J. B. Pre-ceramic art from Huaca Prieta, Chicama Valley. *Ñawpa Pacha: Journal of*

*Andean Archaeology* 29–34 (1963).

242. Grobman, A. *et al.* Preceramic maize from Paredones and Huaca Prieta, Peru. *Proc. Natl. Acad. Sci. U. S. A.* **109**, 1755–1759 (2012).

243. Dillehay, T. D. *Where the Land Meets the Sea: Fourteen Millennia of Human History at Huaca Prieta, Peru*. 741–780 (University of Texas Press, Texas, 2017).

244. Sandweiss, D. H. *et al.* Quebrada jaguay: early south american maritime adaptations. *Science* **281**, 1830–1832 (1998).

245. Rademaker, K., Bromley, G. R. M. & Sandweiss, D. H. Peru archaeological radiocarbon database, 13,000–7000 14C B.P. *Quat. Int.* **301**, 34–45 (2013).

246. McInnis, H. Subsistence and maritime adaptations at Quebrada Jaguay, Camana, Peru: a faunal analysis. (University of Maine, 1999).

247. Jones, K. B., Hodgins, G. W. L. & Sandweiss, D. H. Radiocarbon Chronometry of Site QJ-280, Quebrada Jaguay, a Terminal Pleistocene to Early Holocene Fishing Site in Southern Peru. *The Journal of Island and Coastal Archaeology* **14**, 82–100 (2017).

248. Tanner, B. R. Lithic Analysis of Chipped Stone Artifacts Recovered From Quebrada Jaguay, Peru. (The University of Maine, The University of Maine, 2001).

249. Reitz, E. J., McInnis, H. E., Sandweiss, D. H. & Others. Terminal Pleistocene and Early Holocene fishing strategies at Quebrada Jaguay and the Ring Site, southern Peru. *Journal of Archaeological Science: Reports* **8**, 447–453 (2016).

250. Jones, K. B., Hodgins, G. W. L., Etayo-Cadavid, M. F., Andrus, C. F. T. & Sandweiss, D. H. Centuries of Marine Radiocarbon Reservoir Age Variation Within Archaeological Mesodesma Donacium Shells from Southern Peru. *Radiocarbon* **52**, 1207–1214 (2010).

251. Keefer, D. K. *et al.* Early Maritime Economy and El Niño Events at Quebrada Tacahuay, Peru. *Science* **281**, 1833–1835 (1998).

252. de France, S. D. & Keefer, D. K. Quebrada Tacahuay, Southern Peru: A Late Pleistocene Site Preserved by a Debris Flow. *J. Field Archaeol.* **30**, 385–399 (2005).

253. DeFrance, S. D. & Álvarez, A. U. Quebrada Tacahuay: un sitio marítimo del Pleistoceno tardío en la costa sur del Perú. *Chungará (Arica)* **36**, 257–278 (2004).

254. Sandweiss, D. H., Richardson, J. B., III, Reitz, E. J., Hsu, J. T. & Feldman, R. A. Early maritime adaptations in the Andes: Preliminary studies at the Ring Site, Peru. *Ecology, Settlement, and History in the Osmore Drainage, Peru, Oxford: British Archaeology International Series* 35–84 (1989).

255. Suárez, R., Piñeiro, G. & Barceló, F. Living on the river edge: The Tigre site (K-87) new data and implications for the initial colonization of the Uruguay River basin. *Quat. Int.* (2017) doi:10.1016/j.quaint.2017.08.024.

256. López Mazz, J. M. Early human occupation of Uruguay: Radiocarbon database and archaeological implications. *Quat. Int.* **301**, 94–103 (2013).

257. Suárez, R. High resolution AMS 14C dates for late Pleistocene Fishtail technology from the Tigre site, Uruguay river basin, South America. *Quat. Sci. Rev.* (2019) doi:10.1016/j.quascirev.2019.04.009.

258. Suárez, R. & López, J. M. Archaeology of the Pleistocene–Holocene transition in Uruguay: an overview. *Quat. Int.* **109–110**, 65–76 (2003).

259. Suárez, R. Movilidad, acceso y uso de ágata traslúcida por los cazadores-recolectores tempranos durante la transición Pleistoceno-Holoceno en el Norte de Uruguay (ca. 11,000-8500 AP). *Latin American Antiquity* **22**, 359–384 (2011).

260. Jaimes, A. El Vano: El Jobo Traditions in Megathere Kill Site. *Current Research in the Pleistocene* **15**, 25–27 (1998).

261. Jaimes, A. Condiciones tafonómicas, huesos modificados y comportamiento humano en los sitios de matanza de El Vano (tradición El Jobo) y Lange/Ferguson (tradición Clovis). *Boletín de Antropología Americana* 159–184 (2005).

262. Quero, A. J. El Vano: una nueva localidad paleo-india en el noroccidente de Venezuela. *Maguaré* **17**, 46–64 (2003).

263. McGeehin, J. *et al.* Stepped-Combustion 14C Dating of Sediment: A Comparison with Established Techniques. *Radiocarbon* **43**, 255–261 (2001).

264. Miyairi, Y., Yoshida, K., Miyazaki, Y., Matsuzaki, H. & Kaneoka, I. Improved 14C dating of a tephra layer (AT tephra, Japan) using AMS on selected organic fractions. *Nucl. Instrum. Methods Phys. Res. B* **223-224**, 555–559 (2004).

265. Pessenda, L. C. R., Gouveia, S. E. M. & Aravena, R. Radiocarbon Dating of Total Soil Organic Matter and Humin Fraction and Its Comparison with 14C Ages of Fossil Charcoal. *Radiocarbon* **43**, 595–601 (2001).

266. Wang, Y., Amundson, R. & Trumbore, S. Radiocarbon Dating of Soil Organic Matter. *Quat. Res.* **45**, 282–288 (1996).

267. Zazzo, A. & Saliège, J.-F. Radiocarbon dating of biological apatites: A review. *Palaeogeogr. Palaeoclimatol. Palaeoecol.* **310**, 52–61 (2011).

268. Gruhn, R. & Bryan, A. L. The record of Pleistocene megafaunal extinction at Taima-Taima, northern Venezuela. *Pleistocene extinctions: A prehistoric revolution. University of Arizona Press, Tucson* 128–137 (1984).

269. Bryan, A. L. & Gruhn, R. The radiocarbon dates of Taima-Taima. in *Taima-Taima : A Late Pleistocene Paleo-Indian Kill Site in Northernmost South America— Final Reports of 1976 Excavations* (eds. Ochsenius, C. & Gruhn, R.) 53–58 (Programa CIPICS, Monografias Cientificas, Universidad Francisco de Miranda, Coro, Venezuela, 1979).

270. Ochsenius, C. & Gruhn, R. *Taima-Taima : A Late Pleistocene Paleo-Indian Kill Site in Northernmost South America—Final Reports of 1976 Excavations*. (Programa CIPICS, Monografias Cientificas, Universidad Francisco de Miranda, Coro, Venezuela, 1979).

271. Tuross, N. Albumin preservation in the Taima-taima mastodon skeleton. *Appl. Geochem.* **4**, 255–259 (1989).

272. Ranere, A. & López, C. Cultural diversity in late Pleistocene/early Holocene populations in Northwest South America and lower Central America. *International Journal of South*

*American* **1**, 25–31 (2007).

273. Tamers, M. A. Radiocarbon dating of kill sites. *Archaeometry* **14**, 21–26 (1972).

274. Bampi, H., Barberi, M. & Lima-Ribeiro, M. S. Megafauna kill sites in South America: A critical review. *Quat. Sci. Rev.* **298**, 107851 (2022).

275. Carlini, A. A. *et al.* Damaged glyptodontid skulls from Late Pleistocene sites of northwestern Venezuela: evidence of hunting by humans? *Swiss J. Palaeontol.* **141**, (2022).

276. Zazzo, A. Bone and enamel carbonate diagenesis: A radiocarbon prospective. *Palaeogeogr. Palaeoclimatol. Palaeoecol.* **416**, 168–178 (2014).
