# Supplementary material for: Climate influence on the early human occupation of South America during the late Pleistocene
Source: Nat Commun. 2025 Mar 21;16:2780. doi: 10.1038/s41467-025-58134-5 (PMC11928665; doi:10.1038/s41467-025-58134-5)
Supplement: Supplementary file 2 — Description of Additional Supplementary Files [file 41467_2025_58134_MOESM2_ESM.pdf]

Description of Additional Supplementary Files

Supplementary Data 1

Description - Archaeological and chronometric data for sites with ACR/YD-aged components in South America. Data points that were excluded from the analysis are in purple cells (explanations can be found in the notes column or in each site report within the Supplementary Information document). Coordinates follow the World Geodetic System (WGS84). z refers to depth in metres. The percent collagen is the yield of extracted collagen as a function of the starting weight of bone samples. C:N is the atomic weight ratio of carbon to nitrogen. %C and % N is the percentage of carbon and nitrogen in the combusted sample. BP refers to 'before present', with present for radiocarbon measurements set at AD 1950. Date error (±) is to 1σ. n/a refers to 'not applicable'.

Supplementary Data 2

Supplementary Data 2 is a zipped folder containing a GIF file with the caption:

Description - GIF showing the spatio-temporal distribution of cultural components during the ACR-YD period according to province. The distributions entered correspond to those in Supplementary Figures 1-8, as well as three start boundaries for Caverna da Pedra Pintada (see site report), which is the sole representative of the Amazonian Lowlands. Each circle is sized according to probability. Colours as in Figure 1.

Supplementary Data 3

Supplementary Data 3 is a zipped folder containing a GIF file with the caption:

Description - GIF showing the spatio-temporal distribution of cultural components during the ACR-YD period included in the modelling according to lithic technology. The distributions entered correspond to those in Supplementary Figures 12-21. Each circle is sized according to probability. Colours as in Figure 1.

Supplementary Data 4

Supplementary Data 4 is a zipped folder containing a GIF file with the caption:

Description - GIF showing the spatio-temporal distribution of cultural components during the ACR-YD period included in the modelling according to altitude (with high altitude ≥2,500 metres above sea level). The distributions entered correspond to those in Supplementary Figure 22. Each circle is sized according to probability. <2,5000 masl = black. ≥2,5000 masl = purple.

Supplementary Data 5

Supplementary Data 5 is a zipped folder containing a GIF file with the caption:

Description - GIF showing the spatio-temporal distribution of cultural components during the ACR-YD period included in the modelling according to direct evidence for megafauna killing/ scavenging by humans. The distributions entered correspond to those in Supplementary Figure 23. Each circle is sized according to probability. MF kill/scavenge evidence; no = black, yes = purple.
