# Supplementary figures and images for: Climate influence on the early human occupation of South America during the late Pleistocene
Source: Nat Commun. 2025 Mar 21;16:2780. doi: 10.1038/s41467-025-58134-5 (PMC11928665; doi:10.1038/s41467-025-58134-5)

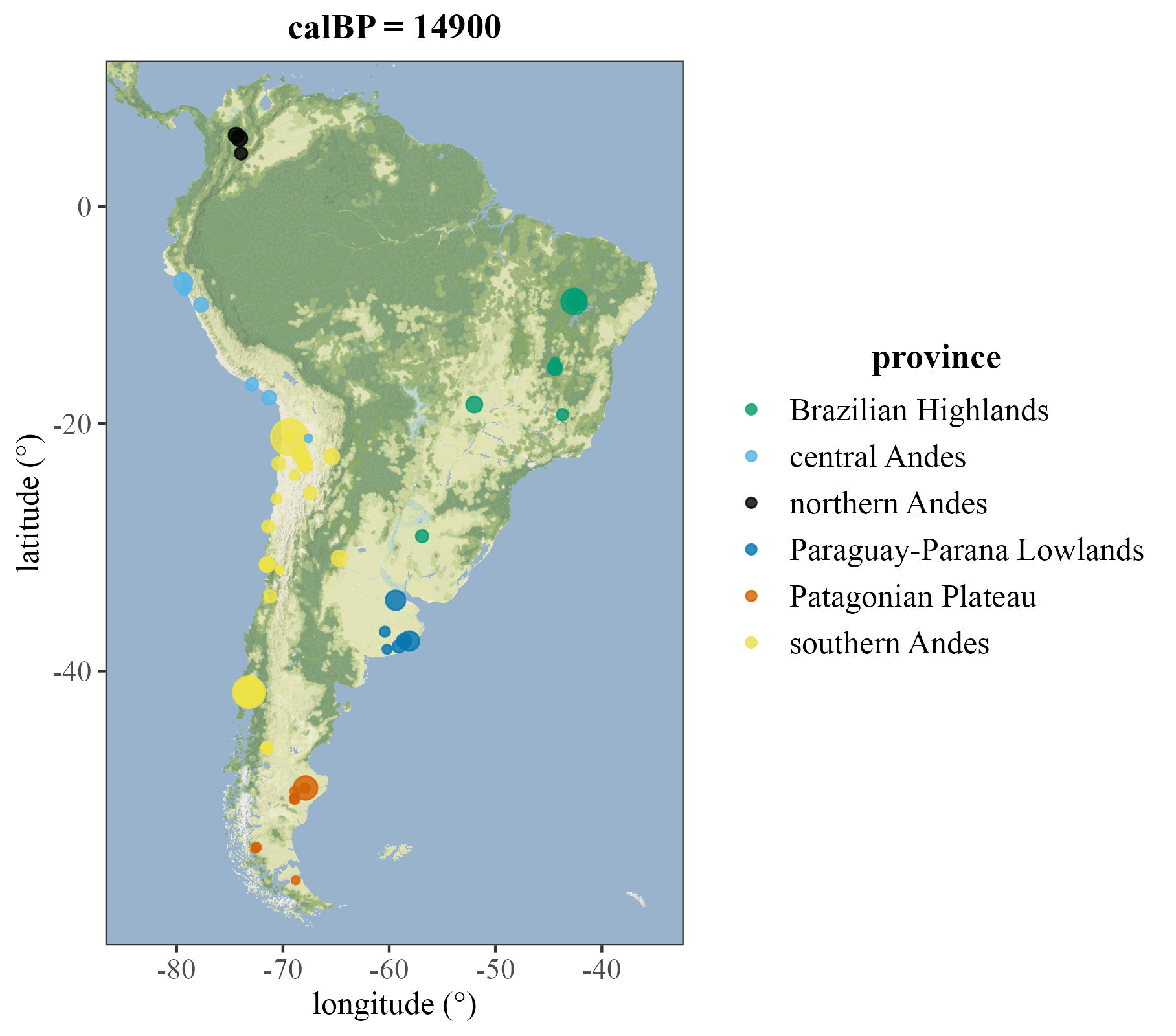

Supplement: Supplementary file 4 — Supplementary Data 2 [file 41467_2025_58134_MOESM4_ESM.zip › Supplementary Data 2/Supplementary Data 2.gif]

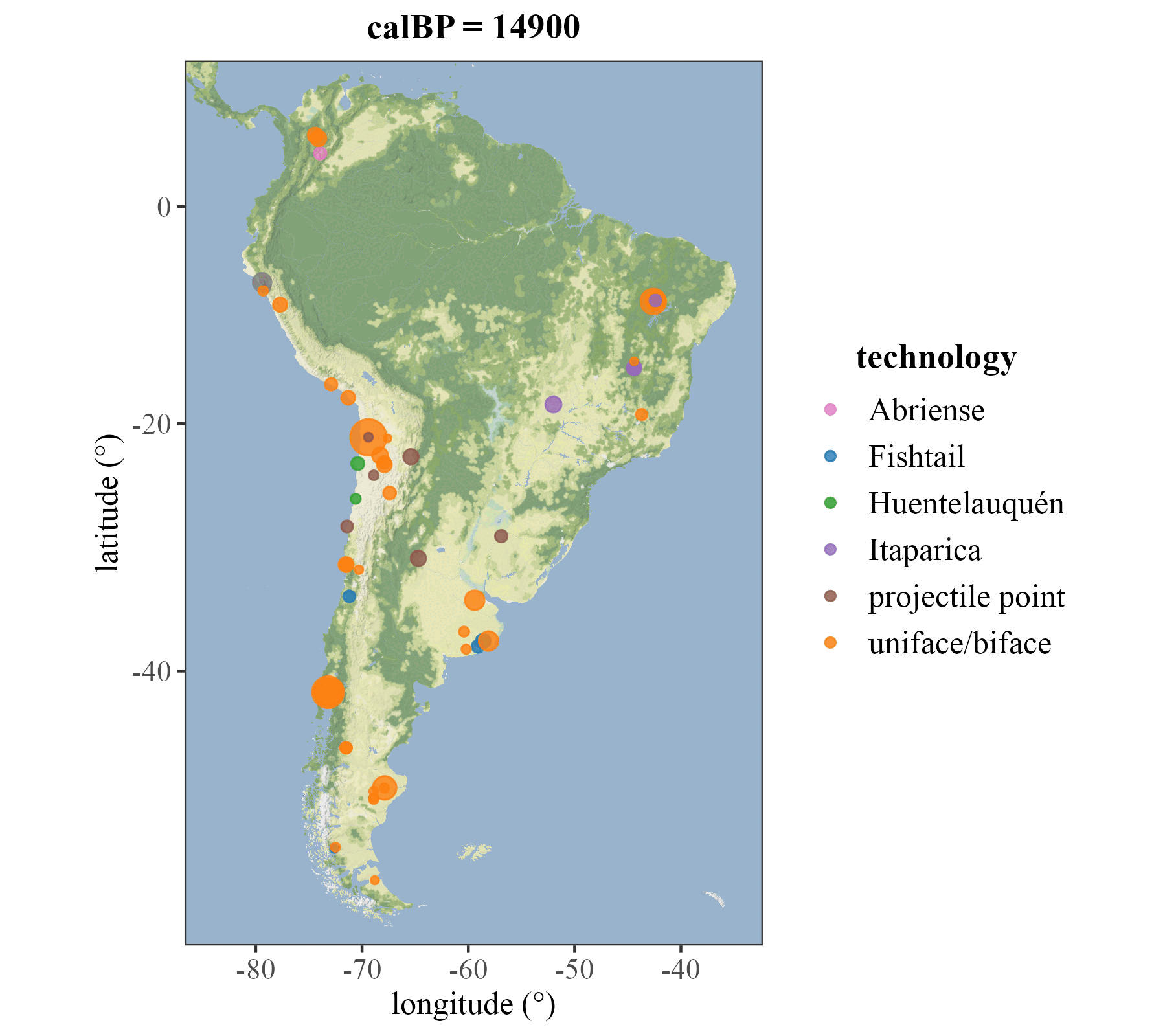

Supplement: Supplementary file 5 — Supplementary Data 3 [file 41467_2025_58134_MOESM5_ESM.zip › Supplementary Data 3/Supplementary Data 3.gif]

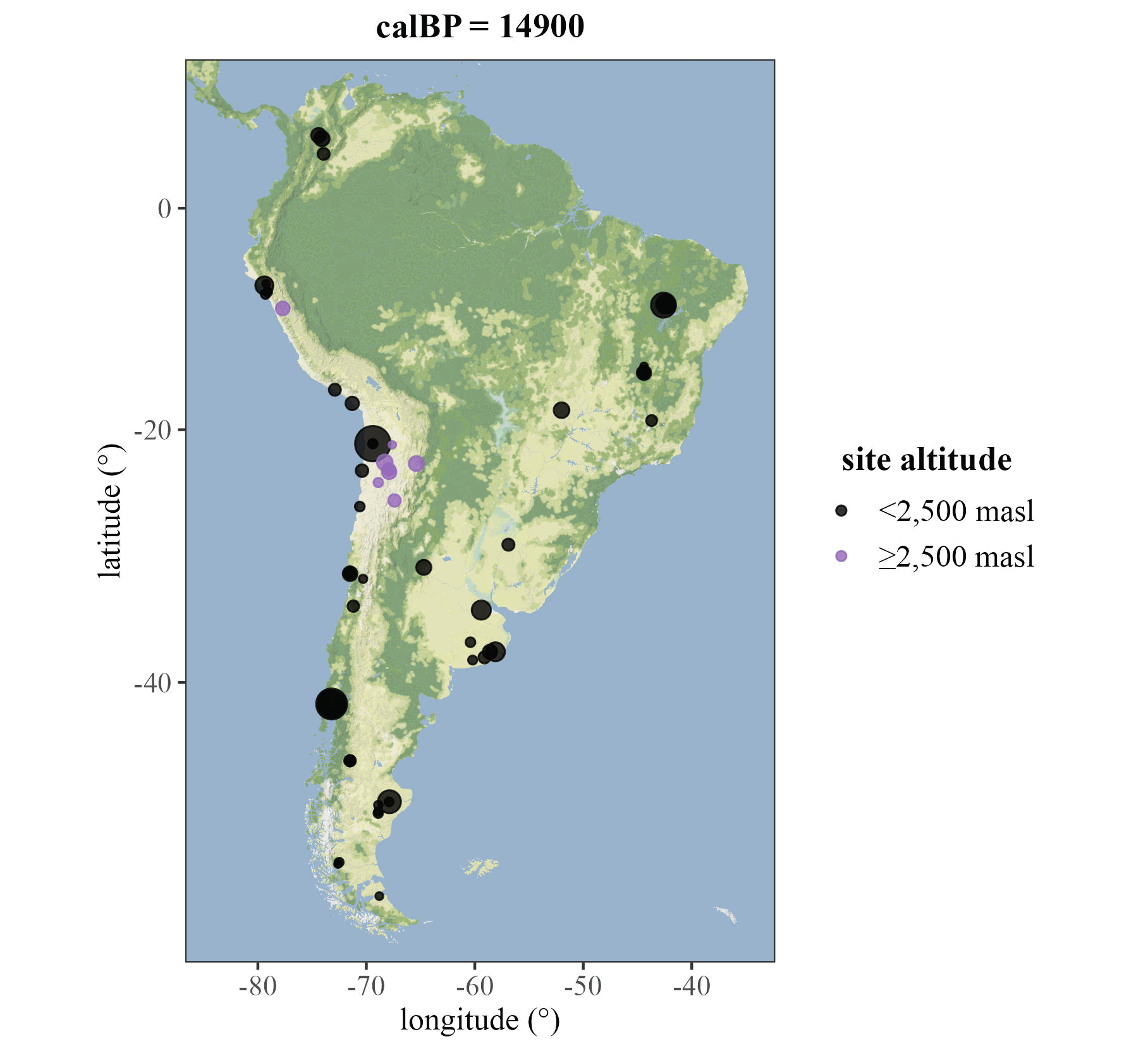

Supplement: Supplementary file 6 — Supplementary Data 4 [file 41467_2025_58134_MOESM6_ESM.zip › Supplementary Data 4/Supplementary Data 4.gif]

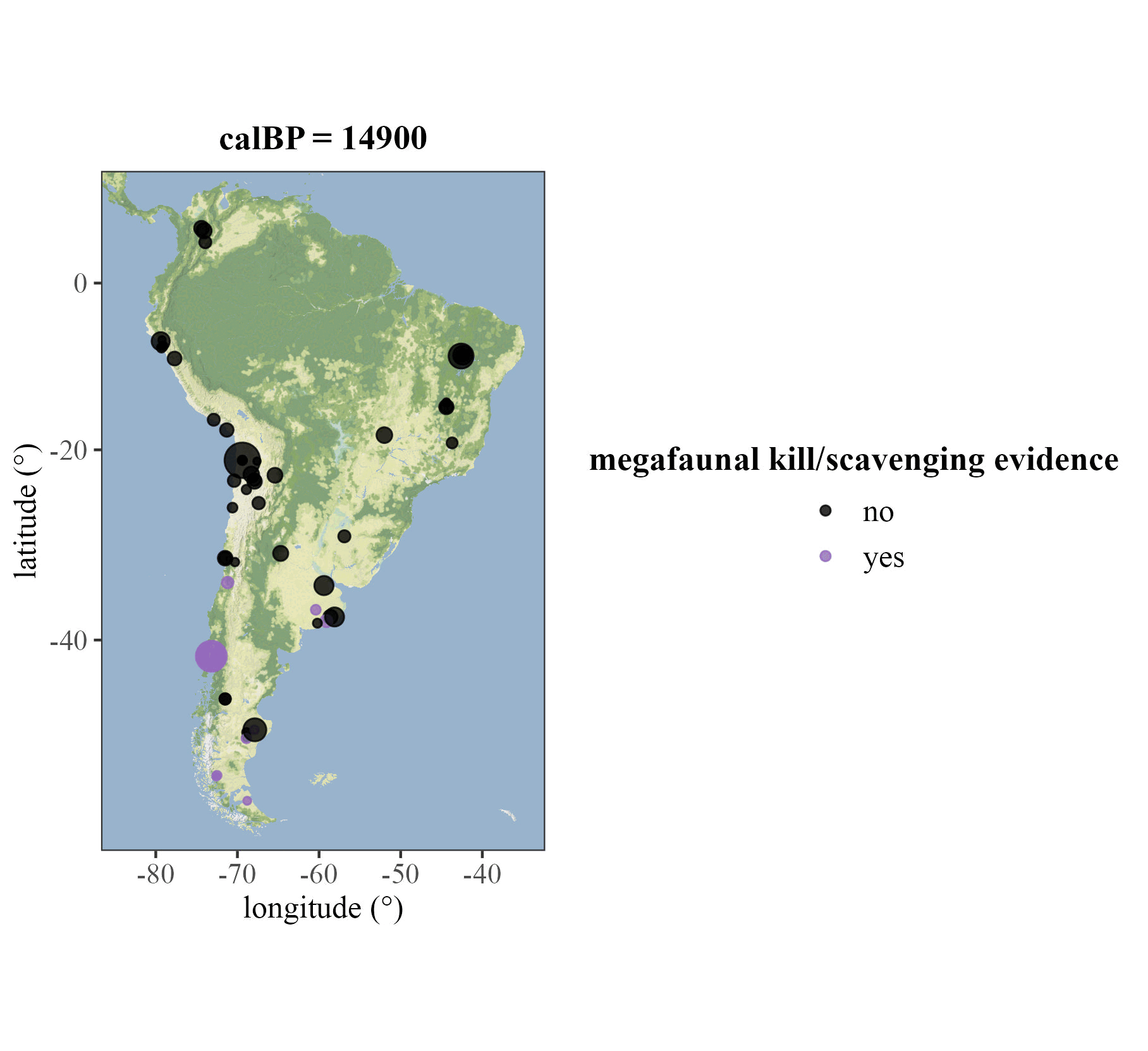

Supplement: Supplementary file 7 — Supplementary Data 5 [file 41467_2025_58134_MOESM7_ESM.zip › Supplementary Data 5/Supplementary Data 5.gif]
